# Variability in non-invasive tests: Challenges and implications for monitoring liver disease

**DOI:** 10.1097/HC9.0000000000001077

**Published:** 2026-11-11

**Authors:** Georg Semmler, Katrine Prier Lindvig, Lucie Simonis, Jan Embacher, Philipp Hruska, Peter Andersen, Yannick Marcour, Benedikt Mehl, Lorenz Balcar, Mathias Jachs, Katrine Holtz Thorhauge, Benedikt Simbrunner, Johanne Kragh Hansen, Benedikt S. Hofer, Mads Israelsen, Lukas Hartl, Patrick Haselwanter, Adrian Wunderbaldinger, Paula Höfinger, Ida Falk Villesen, Robert Winker, Michael Trauner, Aleksander Krag, Thomas Reiberger, Thomas-Matthias Scherzer, Mattias Mandorfer, Maja Thiele

**Affiliations:** 1 Division of Gastroenterology and Hepatology, Department of Medicine III, Medical University of Vienna, Vienna, Austria; 2 Vienna Hepatic Hemodynamic Lab, Division of Gastroenterology and Hepatology, Department of Medicine III, Medical University of Vienna, Vienna, Austria; 3 FLASH Centre for Liver Research, Department of Gastroenterology and Hepatology, Odense University Hospital, Odense, Denmark; 4 Institute of Clinical Research, Faculty of Health Sciences, University of Southern Denmark, Odense, Denmark; 5 Health and Prevention Center, Sanatorium Hera, Vienna, Austria; 6 Department of Medicine I—Gastroenterology and Hepatology, University Hospital Wiener Neustadt, Wiener Neustadt, Austria

**Keywords:** liver fibrosis, minimal detectable change (MDC), non-invasive tests, regression to the mean, vibration-controlled transient elastography (VCTE)

## Abstract

**Background:**

Diagnostic and prognostic non-invasive fibrosis tests (NITs) are increasingly repurposed to monitor liver disease. However, their reproducibility and the minimal detectable change (MDC, ie, the smallest change that exceeds expected measurement variability) are poorly characterized.

**Methods:**

We retrospectively assessed variability of blood-based NITs and liver stiffness measurement (LSM) by vibration-controlled transient elastography on the same day (repeatability) and across use-cases when metabolic dysfunction–associated steatotic liver disease, fibrosis, or portal hypertension is suspected. Measurements were done within 90 days. Agreement and variability were quantified using intraclass correlation coefficients (ICC), Bland–Altman analyses, and MDC on the original (absolute) and relative (MDC%) scales.

**Results:**

Across 6 cohorts, ICCs were generally good to excellent, but within-subject variability remained substantial. Same-day LSM had a pooled MDC% of 24% on the m/s scale, corresponding to an asymmetric kPa-equivalent window of −43% to +54%. For longitudinal lab-based NITs, pooled MDC% was 47% for FIB-4 and 56% for APRI, while absolute MDC was 13–17 percentage points for LiverPRO and 3 percentage points for CORE. Optimistic estimates remained substantial after excluding the 10% most extreme paired differences. Variability increased with biomarker level for LSM, FIB-4, and APRI, whereas MDC windows for probability-based scores were strongly baseline-dependent and followed an inverse U-shaped pattern. When repeat testing was triggered by an elevated initial LSM, regression to the mean accounted for most or all of the observed decrease.

**Conclusions:**

Good agreement in NITs does not guarantee high precision for disease monitoring. MDC depends on biomarker level, clinical setting, measurement scale, and selection for repeat testing. Universal thresholds should not be applied uncritically, and repeated measurements, baseline-dependent interpretation, and regression to the mean should guide monitoring decisions.

## INTRODUCTION

Non-invasive tests (NITs) are widely used in hepatology to screen for, diagnose, prognosticate, and monitor liver fibrosis.^1^,^2^ Because of their low cost, wide availability, and high patient acceptance, they are applied in multiple settings beyond cross-sectional fibrosis assessment, for which most of them were originally designed. Specifically, they are regularly used to provide updated risk estimates for disease progression or regression, the development of clinically significant portal hypertension (CSPH), or liver-related events.^3^,^4^,^5^,^6^,^7^,^8^ However, interpreting longitudinal changes comes with 2 key challenges: First, all of them have been developed as one-time diagnostic or prognostic tests, and little attention is paid to what happens when they are used for monitoring. Second, the precision of NITs (ie, measurement variability) and their reproducibility (ie, variation when being repeated) have received surprisingly little attention. Instead, obtained results are often considered the ground truth, as acknowledging their random variation complicates interpretation in clinical practice.^9^ Here, the minimal detectable change (MDC), corresponding to the degree of change that can be considered not random with 95% confidence, is barely discussed or acknowledged.^10^ However, understanding this variability becomes particularly important when monitoring liver-specific treatments such as resmetirom or semaglutide for metabolic dysfunction–associated steatotic liver disease (MASLD), where recommendations are necessary despite limited supporting evidence.^11^,^12^ While data on the reliability of blood-based NITs are nearly absent,^9^,^13^ evidence on the reliability and reproducibility of liver stiffness measurement (LSM) by vibration-controlled transient elastography (VCTE) as the most prominent imaging-based NIT is increasing, yet goes largely unrecognized. Thus, the aim of this study was to explore and characterize the measurement variability of commonly used NITs across clinical scenarios where they are applied.

## METHODS

### Objectives and rationale

The objective of this study was to assess the measurement variability (ie, precision) of VCTE and lab-based NITs that were designed as diagnostic tests (for liver fibrosis) or prognostic tests (for associated risk) measured at a single time point. Variability was studied both on the same day in the same setting to assess repeatability, and when repeated longitudinally in the short term to assess reproducibility, assuming no meaningful change in the underlying disease state. These aspects become essential when the respective biomarkers get repurposed for monitoring of liver disease. The current study was designed to highlight key aspects that are subsequently put into context in the discussion section. Notably, we have previously reported on the repeatability and reproducibility of the controlled attenuation parameter and FAST score in a separate manuscript.^14^

### Cohort overview and analyses

All cohorts include patients with established or suspected steatotic liver disease (SLD) who underwent repeated LSM or repeated blood draw on the same day (repeatability) or <90 days apart (reproducibility), with subgroup analyses within 28 days. Patients with chronic liver diseases other than SLD were excluded. If not otherwise specified, LSM and laboratory parameters were obtained on the same day. An extended description of the cohorts as well as ethics can be found in the Supplement, https://links.lww.com/HC9/C512.

#### Part A: Same-day study on repeatability

We assessed same-day repeatability of NITs when being repeated on the same day in the following settings:

1. *Repeatability cohort I—LSM*: Subjects with suspected MASLD from secondary care.
2. *Repeatability cohort II* *—LSM*: Subjects with suspected SLD from tertiary care.
3. *Repeatability cohort III—Lab-based NITs and standard laboratory tests*: Subjects with suspected SLD from tertiary care.

This informs on the smallest MDC that is to be expected under highly controlled conditions on the same day, in the same setting and by the same operator (as for LSM).

#### Part B: Longitudinal study on reproducibility

We assessed reproducibility of NITs across different scenarios in which they are applied:

1. *Reproducibility cohort I—suspected MASLD*: Subjects with suspected MASLD from secondary care.
2. *Reproducibility cohort II—suspected fibrosis*: Subjects with suspected fibrosis from secondary care referred to liver biopsy.
3. *Reproducibility cohort III—suspected portal hypertension*: Subjects with suspected portal hypertension from tertiary care referred for HVPG measurement.

These data inform the MDC captured by respective NITs in clinical application in a longitudinal setting (when being applied for monitoring of fibrosis dynamics).

#### Part C: Exploring the impact of scale on variability

LSM is expressed in kPa but is derived from shear wave speed *V* (m/s) using the elastic modulus approximation *E* = 3ρ*V* ^2^, where ρ represents tissue density, assumed to be approximately 1000 kg/m^3^. This influences measurement variability if interpreted on the kPa scale. To showcase this phenomenon, we compare variability as obtained from kPa and m/s and simulate how this influences the MDC.

For NITs expressed as probabilities, interpretation of change is complicated by their bounded 0%–100% scale and by the nonlinear relationship between the probability scale and the underlying linear predictor, approaching 0% and 100% asymptotically. Therefore, we compared MDC estimates obtained on the original probability scale with MDC estimates obtained on the logit-transformed scale, and back-transformed logit-based reliable-change limits to illustrate how the required change depends on the baseline probability.

#### Part D: Confirming elevated LSM in clinical practice

The availability of a second measurement in Reproducibility cohorts II and III was often conditional on an elevated first LSM leading to further referral and repeated measurement (≥6 and ≥10 kPa, respectively). Consequently, paired changes (and derived variability) were inherently affected by regression-to-the-mean (RTM), precluding unbiased direct comparison with other NITs. However, this scenario is highly prevalent and relevant in clinical practice, and quantifying this phenomenon is of high importance. Thus, we quantified RTM in these cohorts separately and assessed variability with regard to the level of the first measurement.

### Non-invasive tests

The following lab-based NITs were assessed: FIB-4 (age, AST, ALT, and platelet count), APRI (AST and platelet count), LiverPRO (age, alkaline phosphatase, AST, and gamma-glutamyl transferase [GGT], platelet count, INR, bilirubin, albumin, sodium, and cholesterol),^15^ and CORE (sex, age, AST, ALT, and GGT).^16^ Specifically, all NITs except LiverPRO required the complete availability of all input parameters, while LiverPRO employs a platform providing different algorithms depending on the lab parameters available. Also, this platform provides estimates for the probability of having F2 (significant fibrosis), F3 (advanced fibrosis), and F4 (cirrhosis), which were analyzed separately. We also studied VCTE-based LSM as well as its extensions, Agile 3+ and Agile 4 score (sex, age, LSM, platelet count, AST, ALT, and diabetes).^17^ Further details on respective formulas are provided in the supplement, Supplemental Digital Content 1, https://links.lww.com/HC9/C512. As the LiverRisk score is only intended for general population screening, it should not be used outside this setting, and its analysis was discouraged by the developers. As such, it was not included in the current manuscript.

Importantly, all laboratory measurements and LSM were performed in fasting conditions. Reliability criteria for LSM (interquartile range [IQR]/median <30%) were applied in sensitivity analyses.

### Statistics

Key metrics of agreement included the Intraclass Correlation Coefficient (ICC), Bland–Altman analyses, and the MDC on the original scale (ie, absolute MDC) and relative scale (MDC%).^18^ A detailed explanation of how these parameters relate to each other and how they are computed is provided in the Supplement, https://links.lww.com/HC9/C512, together with a full description of statistical methods.

## RESULTS

### Patient characteristics

Across the 6 cohorts, mean age ranged from 45 to 59 years, and patients were predominantly male (Table 1). The prevalence of obesity was ~60% in secondary care cohorts and 33%–47% in tertiary care. The proportion of patients with MetALD and alcohol-associated liver disease increased in tertiary care cohorts. Median LSM in Repeatability cohort I was 5.3 [4.4–6.1] kPa and 7.5 [5.9–10.5] kPa in Repeatability cohort II. Within the Reproducibility cohorts (longitudinal study, part B), LSM and other NITs reflecting fibrosis severity increased from 5.3 kPa in Reproducibility cohort I (suspected MASLD) to 9.2 kPa in Reproducibility cohort II (suspected fibrosis) and 27.9 kPa in Reproducibility cohort III (suspected portal hypertension), corresponding to more advanced patient populations.

**TABLE 1:** Patient characteristics of the 3 repeatability and 3 reproducibility cohorts at the time point of first measurement

	Repeatability cohort I, n=54	Repeatability cohort II, n=58	Repeatability cohort III, n=89	Reproducibility cohort I, n=61	Reproducibility cohort II, n=387	Reproducibility cohort III, n=88
Setting	Secondary care	Tertiary care	Tertiary care	Suspected MASLD	Suspected fibrosis	Suspected portal hypertension
Age, years	46±9	59±15	52±15	45±9	59±9	58±11
Male sex	34 (63%)	33 (57%)	44 (49%)	40 (66%)	250 (65%)	61 (69%)
BMI, kg/m^2^	31.5±3.4	31.4±6.6	28.1±6.7	31.5±3.6	33.8±7.4	28.2±5.9
≥30 kg/m^2^	33 (61%)	27 (47%)	29 (33%)	37 (61%)	241 (62%)	33 (38%)
WC, cm	109±8	107±17^a^	—	109±8	112±16	—
Abdominal obesity	54 (100%)	47 (85%)^a^	—	60 (98%)	348 (90%)	—
Diabetes	0 (0%)	25 (43%)	28 (32%)	0 (0%)	99 (26%)	29 (33%)
Etiology						
MASLD	54 (100%)	49 (85%)	50 (56%)	61 (100%)	239 (62%)	33 (38%)
MetALD	0 (0%)	1 (1.7%)	2 (2.2%)	0 (0%)	46 (12%)	5 (5.7%)
ALD	0 (0%)	7 (12%)	19 (21%)	0 (0%)	32 (8.3%)	45 (51%)
Cryptogenic	0 (0%)	1 (1.7%)	3 (3.4%)	0 (0%)	0 (0%)	5 (5.7%)
No SLD	—	—	15 (17%)	—	70 (18%)	—
LSM, kPa	5.3 [4.4–6.1]	7.5 [5.9–10.5]	10.0 [5.9–28.4]	5.3 [4.4–6.0]	9.2 [7.9–11.1]	27.9 [18.0–51.9]
≥8 kPa	3 (5.6%)	25 (43%)	51 (57%)	3 (4.9%)	289 (75%)	88 (100%)
≥12 kPa	0 (0%)	9 (16%)	42 (47%)	0 (0%)	70 (18%)	80 (91%)
≥25 kPa	0 (0%)	1 (1.7%)	26 (29%)	0 (0%)	17 (4.4%)	52 (59%)
Agile 3+, %	—	—	—	6.8 [3.3–12.0]	40.1 [24.9–62.1]	98.4 [88.4–99.4]
Agile 4, %	—	—	—	0.8 [0.3–1.6]	5.8 [2.7–13.1]	77.2 [48.3–91.6]
FIB-4, points	—	—	1.56 [0.76–4.48]	0.86 [0.66–1.19]	1.30 [0.97–1.73]	3.78 [2.02–5.39]
APRI, points	—	—	0.55 [0.26–0.99]	0.37 [0.29–0.43]	0.31 [0.23–0.46]	0.88 [0.55–1.49]
LiverPRO-F2, %	—	—	58 [24–81]	15 [9–25]	37 [23–57]	80 [72–91]
LiverPRO-F3, %	—	—	17 [2–82]	2 [1–4]	5 [1–12]	65 [36–83]
LiverPRO-F4, %	—	—	9 [2–59]	1 [1–3]	4 [1–10]	39 [18–63]
CORE, %	—	NA	NA	0.24 [0.14–0.50]	0.58 [0.24–2.2]	NA
ELF, points	—	—	—	—	9.3 [8.8–9.9]^b^	—
Time between time point 1 and 2, days	Same day	Same day	Same day	24 [17–38]	34 [19–47]	34 [22–50]

^a^ Missing in n=3 (5.2%).

^b^ Missing in 161 (42%).

Abbreviations: ALD, alcohol-associated liver disease; APRI, AST-to-platelet ratio index; BMI, body mass index; AST, aspartate aminotransferase; ELF, Enhanced Liver Fibrosis test; FIB-4, Fibrosis-4 score; LSM, liver stiffness measurement; MASLD, metabolic dysfunction–associated steatotic liver disease; MetALD, metabolic dysfunction and alcohol-associated liver disease; NA, not applicable; NIT, non-invasive test; SLD, steatotic liver disease; WC, waist circumference.

### Part A: Same-day study on repeatability

First, we studied same-day repeatability of LSM in one cohort from secondary and one from tertiary care on the m/s scale (Table 2). Agreement between measurements was good (ICC 0.79–0.82), and the MDC in Repeatability cohort I (secondary care) was 0.2 m/s, corresponding to a relative MDC% of 15% (95% CI: 12%–17%). This translated into an MDC% of −28 to +32% on the kPa scale, and a back-transformed MDC of −1.8 to +2.1 kPa at an 8 kPa reference value. In Repeatability cohort II (tertiary care), variability increased with an MDC of 0.5 m/s, corresponding to −4.1 to +5.6 kPa at an 8 kPa reference value, with MDC% of 30% on the m/s scale and −52% to +70% on the kPa scale, respectively. Of note, results were similar when limiting analyses to patients with IQR/median <0.3 at both measurements. Optimistic estimates for MDC and MDC% when excluding 10% of patients with the most extreme differences between measurements reduced estimates, yet MDC% for LSM remained above 20% change in any direction. Supporting Bland–Altman plots and scatterplots are provided in Attachment I.

**TABLE 2:** Measures of same-day variability in the 3 repeatability cohorts I–III: Repeatability cohort I—LSM including n=54 subjects with suspected MASLD (LSM: same session), Repeatability cohort II—LSM including n=58 subjects with suspected SLD (LSM: same consultation), and Repeatability cohort III—Lab-based NITs and standard laboratory tests including n=89 patients with suspected SLD

	Overall cohort			Excluding 10% extremes (optimistic estimates)		
Parameter	ICC (95% CI)	MDC (95% CI), original scale	MDC% (95% CI), %	ICC (95% CI)	MDC (95% CI), original scale	MDC% (95% CI), %
Repeatability cohort I						
LSM, m/s	0.79 (0.67–0.87)	0.20 (0.16–0.24)	15.0 (12.4–17.7)	0.88 (0.78–0.93)	0.15 (0.13–0.17)	11.6 (9.8–13.3)
*in kPa*	*—*	*−1.8 to +2.1 kPa* ^a^	*−27.8 to +32.3%*	*—*	*−1.4 to + 1.5 kPa* ^a^	*−21.9 to +24.6%*
LSM, m/s (IQR/median <0.3)^b^	0.79 (0.67–0.88)	0.20 (0.16–0.24)	15.2 (12.5–18.0)	0.88 (0.78–0.94)	0.15 (0.13–0.17)	11.7 (9.9–13.5)
Repeatability cohort II						
LSM, m/s	0.82 (0.72–0.89)	0.50 (0.38–0.61)	30.4 (22.4–38.7)	0.91 (0.85–0.95)	0.35 (0.28–0.42)	19.7 (16.7–22.8)
*in kPa*	*—*	*−4.1 to +5.6 kPa* ^*a*^	*−51.6 to +70.1%*	*—*	*−3.1 to +3.8 kPa* ^*a*^	*−35.6 to +43.4%*
LSM, m/s (IQR/median <0.3)^c^	0.85 (0.76–0.91)	0.46 (0.35–0.58)	27.8 (20.0–36.5)	0.92 (0.86–0.95)	0.34 (0.27–0.42)	18.6 (15.5–21.4)
Repeatability cohort III						
FIB-4, points	0.96 (0.94–0.97)	2.7 (1.1–4.0)	40.9 (31.6–49.5)	0.99 (0.99–1.00)	1.0 (0.7–1.3)	26.3 (22.8–29.4)
APRI, points	0.92 (0.88–0.94)	1.9 (0.4–3.1)	53.0 (38.3–66.5)	0.99 (0.98–0.99)	0.4 (0.2–0.6)	29.0 (24.2–33.5)
LiverPRO-F2, %	0.97 (0.95–0.98)	15.5 (6.8–24.3)	NA	0.99 (0.99–1.00)	7.2 (5.8–8.5)	NA
LiverPRO-F3, %	0.97 (0.95–0.98)	19.6 (12.6–26.8)	NA	0.99 (0.98–0.99)	11.7 (8.6–14.6)	NA
LiverPRO-F4, %	0.98 (0.97–0.99)	13.0 (10.0–15.8)	NA	0.98 (0.98–0.99)	11.5 (8.3–14.4)	NA
Platelet count, G/L	0.98 (0.97–0.99)	45 (34–56)	29.6 (20.4–38.0)	0.99 (0.99–0.99)	35 (26–43)	15.9 (13.4–18.1)
AST, U/L	0.95 (0.93–0.97)	22 (15–28)	49.4 (31.1–65.5)	0.99 (0.98–0.99)	11 (9–13)	23.6 (20.0–26.9)
ALT, U/L	0.98 (0.98–0.99)	22 (11–32)	45.3 (20.5–66.4)	0.99 (0.99–1.00)	14 (7–20)	16.8 (14.3–19.0)
GGT, U/L	0.99 (0.98–0.99)	52 (25–73)	40.2 (21.7–55.5)	1.00 (1.00–1.00)	21 (13–29)	14.1 (11.7–16.3)
ALP, U/L	0.96 (0.93–0.97)	41 (14–64)	26.9 (13.5–41.4)	0.99 (0.99–0.99)	12 (9–15)	11.3 (9.8–12.6)
INR	0.96 (0.94–0.98)	0.12 (0.10–0.15)	10.5 (8.6–12.1)	0.97 (0.96–0.98)	0.10 (0.08–0.12)	8.3 (6.6–9.8)
Bilirubin, mg/dL	0.91 (0.86–0.94)	0.48 (0.25–0.73)	47.8 (33.5–60.7)	0.98 (0.97–0.99)	0.22 (0.17–0.28)	24.4 (20.1–28.3)
Albumin, g/L	0.86 (0.80–0.91)	5.3 (4.5–6.0)	12.9 (10.9–14.6)	0.90 (0.85–0.94)	4.3 (3.8–4.9)	10.2 (8.9–11.4)

*Note:* To mitigate the influence of outliers, an additional analysis is provided in which 10% of the cohort with the most extreme differences between measurements 1 and 2 were excluded. For LSM, variability was assessed on the shear-wave speed scale (m/s), since kPa represents a nonlinear transformation of m/s according to kPa = 3 × (m/s)^2^. Therefore, m/s-derived MDC and MDC% estimates were back-transformed to kPa-equivalent values, for absolute MDC at an 8 kPa reference anchor.

^a^ At 8 kPa.

^b^ Including n=52 subjects (96%) with IQR/median <0.3  at both measurements according to established recommendations.

^c^ Including n=50 subjects (86%) with IQR/median <0.3  at both measurements according to established recommendations;

Abbreviations: ALP, alkaline phosphatase; ALT, alanine aminotransferase; APRI, AST-to-platelet ratio index; ELF, Enhanced Liver Fibrosis test; FIB-4, Fibrosis-4 score; GGT, gamma-glutamyl transferase; ICC, intraclass correlation coefficient; INR, international normalized ratio; IQR, interquartile range; LSM, liver stiffness measurement; MASLD, metabolic dysfunction–associated steatotic liver disease; MDC, minimal detectable change; MDC%, minimal detectable change (relative scale); NA, not applicable; SLD, steatotic liver disease; 95% CI, 95% confidence interval.

We then investigated proportional bias, heteroscedasticity, and the impact of absolute measurement value on MDC/MDC% in a pooled analysis and across strata of absolute LSM (<6 kPa, ≥6 kPa; Supplemental Table S1, https://links.lww.com/HC9/C512). Importantly, pooled MDC% was 24% on the m/s scale and −43 to +54% on the kPa scale, with corresponding MDC at clinically relevant cutoffs shown in Figure 1A. Even though relative agreement was substantially higher in the subjects ≥6 kPa (ICC 0.78 vs. 0.41), MDC increased in parallel with measurement level, from MDC% of 18% in those <6 kPa to 29% in those ≥6 kPa. This was consistent with no evidence for proportional bias (ie, no consistent direction of difference with increasing LSM), but significant heteroscedasticity (ie, the degree of variability increased with higher LSM; Figure 1). This was evident on the m/s scale but amplified when analyzed on the kPa scale. Further considerations regarding the impact of measurement scale (kPa vs. m/s) and alternative calculations of MDC are provided in part C.

**FIGURE 1:**
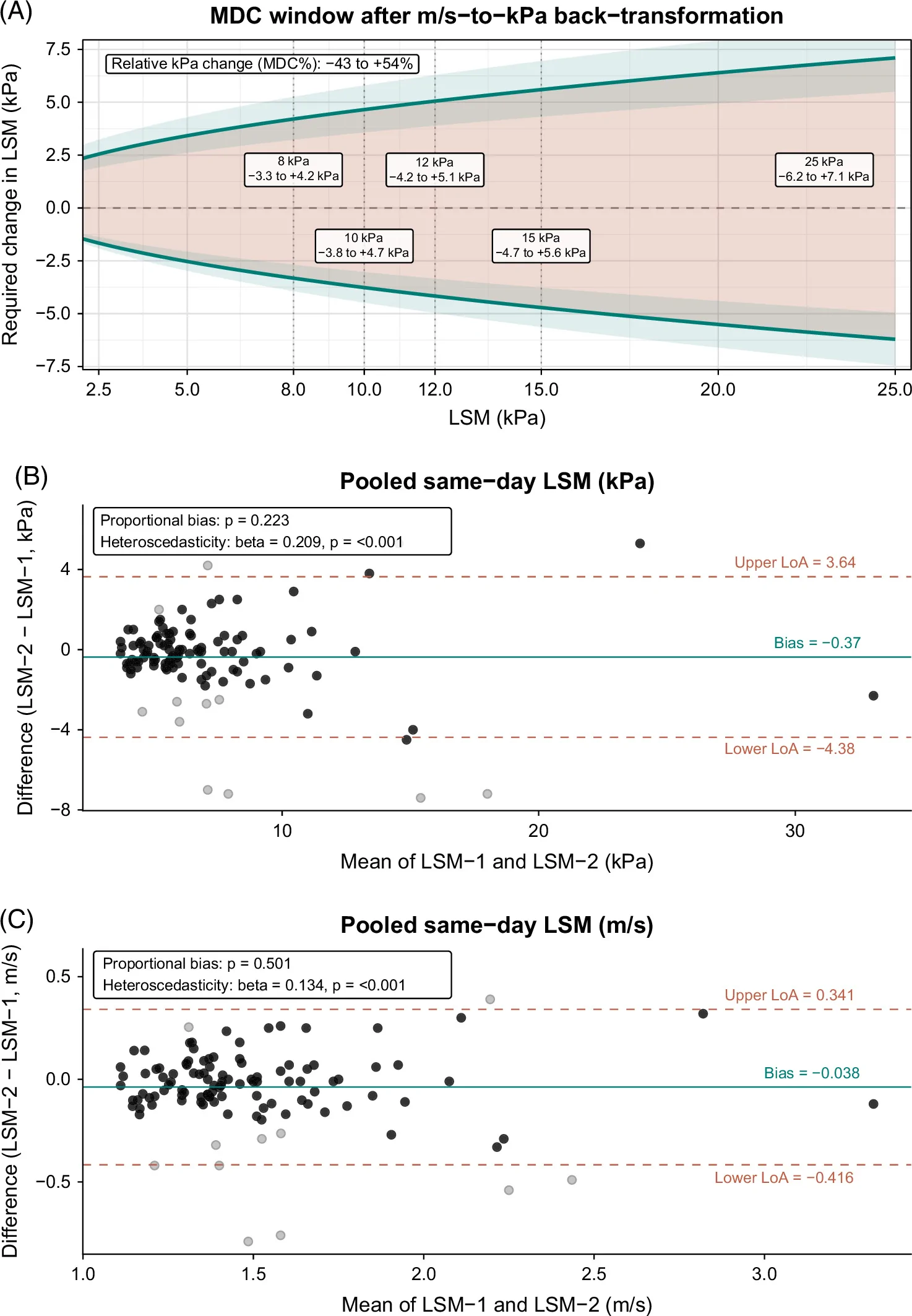
(A) MDC and MDC% estimated for LSM in the pooled cohorts from Repeatability cohort I+II when calculated on the m/s scale, and back-transformed into the corresponding MDC and MDC% in kPa. Due to the nonlinear transformation of shear-wave speed according to kPa = 3 × (m/s)^2^, the MDC in kPa depends on the reference value. (B, C) Bland–Altman plots of same-day repeatability of LSM in a pooled analysis when analyzed on the kPa (B) and m/s (C) scales. The solid horizontal line indicates the mean bias, defined as the average signed difference between repeated measurements. Thus, a bias close to zero indicates no systematic tendency for the second measurement to be higher or lower than the first measurement. The dashed horizontal lines indicate the 95% limits of agreement (LoA), representing the range within which ~95% of differences between repeated measurements are expected to lie. Gray points indicate the 10% of pairs with the largest relative paired differences and are shown to visually identify extreme differences. Proportional bias was assessed by linear regression of the signed paired difference on the paired mean. A significant slope indicates that the direction or magnitude of the signed difference varies systematically across the LSM range. Heteroscedasticity was assessed by linear regression of the absolute paired difference on the paired mean. A significant slope indicates that the magnitude of random measurement variability changes across the LSM range, irrespective of whether the second measurement is higher or lower than the first. Abbreviations: LSM, liver stiffness measurement; MDC, minimal detectable change; MDC%, minimal detectable change on the relative scale.

Next, we studied the same-day repeatability of lab-based NITs and standard laboratory tests. All lab-based NITs showed a strong agreement (ICC 0.92–0.98) with an MDC% of 41% for FIB-4 and an absolute MDC of 13–20 percentage points for LiverPRO versions. For standard laboratory tests, their agreement was similarly strong (ICC 0.86–0.99). Again, MDC% ranged from 11% (INR), 13% (albumin) to ~30% (platelet count, alkaline phosphatase), and ~40%–50% (AST, ALT, GGT, bilirubin), being reduced to 8%–24% in optimistic analyses. This supports the inherent variability of every biomarker measurement.

### Part B: Longitudinal study on reproducibility

We investigated the variability/reproducibility of NITs in 3 clinical scenarios where they are applied: Suspicion of SLD (Reproducibility cohort I), suspicion of fibrosis (Reproducibility cohort II), and suspicion of CSPH (Reproducibility cohort III). Importantly, reproducibility of LSM and derived scores (Agile 3+, Agile 4) was only studied in Reproducibility cohort I, while their dynamics when preselecting patients for liver biopsy (Reproducibility cohort II) or HVPG measurement (Reproducibility cohort III) based on elevated LSM (as done in current practice) is provided in Part D. In Reproducibility cohort I, MDC of LSM was >2.0 kPa (−32% to +38% on the relative scale) while it was 0.6 points for FIB-4 (38%), 7.2 percentage points for LiverPRO-F2, 0.5 percentage points for CORE, 9.3 percentage points for Agile 3+ and 2.2 percentage points for Agile 4 (Supplemental Table S2, https://links.lww.com/HC9/C512). For the lab-based NITs (FIB-4, APRI, LiverPRO-F2/3/4, and CORE), MDC on the original scale increased across Reproducibility cohort II—suspected fibrosis and Reproducibility cohort III—suspected portal hypertension (excluding CORE), in parallel with increasing median values. Taking FIB-4 as an example, this increased from 0.6 points (38%) to 1.1 points (45%) to 3.2 points (59%), highlighting challenges in defining a universal (pooled) MDC or MDC% for any biomarker across different use settings.

Paired ELF measurements were only available in a subgroup of Reproducibility cohort II, with an MDC of 1.2 points, translating into a relative MDC% of 13%.

To further investigate proportional bias and heteroscedasticity and propose level-dependent cutoffs for MDC and MDC% across the range of FIB-4, APRI, and LiverPRO versions and CORE, we pooled data from all 3 reproducibility cohorts (n=536; Table 3). Again, pooled MDC% of FIB-4 and APRI clustered around 40%–50%, while MDC (absolute scale) was 13–17 percentage points for LiverPRO versions and 3 percentage points for CORE. Notably, there was evidence for both proportional bias and heteroscedasticity across all NITs (except LiverPRO-F2), again indicating that pooled MDC/MDC% estimates across the whole biomarker ranges are only partially reflective of the respective variability within certain strata of the biomarkers (Supplemental Figure S1, https://links.lww.com/HC9/C512). Thus, complementary analyses stratified by clinically relevant biomarker ranges (Table 3), as well as locally estimated MDC/MDC%, are provided (Figure 2 and Supplemental Figure S2, https://links.lww.com/HC9/C512). Interestingly, both the MDC and MDC% of FIB-4 and APRI increased approximately linearly before reaching a plateau, while the absolute MDC of LiverPRO versions showed an inverse U-shaped pattern consistent with higher variability around ~50 percentage points while the corresponding MDC% decreased. A similar pattern was observed for CORE, although observations at higher probabilities were sparse in the studied datasets.

**TABLE 3:** Measures of variability in a pooled analysis from Reproducibility cohorts I–III (n=536) including intraclass correlation coefficient (ICC), the minimal detectable change (MDC) on the original (absolute) scale as well as on the relative scale (MDC%)

	Overall cohort			Excluding 10% extremes (optimistic estimates)		
Parameter	ICC (95% CI)	MDC (95% CI), original scale	MDC% (95% CI), %	ICC (95% CI)	MDC (95% CI), original scale	MDC% (95% CI), %
Pooled cohort (n=536)						
FIB-4, points	0.93 (0.91–0.94)	1.59 (1.22–2.00)	46.7 (42.9–51.7)	0.98 (0.97–0.98)	0.77 (0.67–0.88)	32.0 (30.4–33.9)
APRI, points	0.91 (0.89–0.92)	0.54 (0.42–0.68)	55.9 (52.1–61.1)	0.95 (0.95–0.96)	0.38 (0.25–0.53)	40.0 (38.0–42.5)
LiverPRO-F2, %	0.97 (0.96–0.97)	13.4 (11.9–14.9)	NA	0.98 (0.97–0.98)	10.6 (9.4–11.6)	NA
LiverPRO-F3, %	0.94 (0.93–0.95)	16.9 (15.4–19.0)	NA	0.96 (0.95–0.96)	15.8 (14.0–17.5)	NA
LiverPRO-F4, %	0.91 (0.90–0.93)	17.0 (15.0–19.0)	NA	0.94 (0.92–0.95)	15.2 (13.7–17.1)	NA
CORE, %^a^	0.93 (0.91–0.94)	3.1 (2.6–3.8)	NA	0.95 (0.94–0.96)	2.6 (2.0–3.2)	NA
FIB-4 strata						
<1.3, n=245	0.71 (0.65–0.77)	0.37 (0.33–0.41)	39.2 (34.6–43.7)	0.85 (0.80–0.88)	0.26 (0.23–0.29)	27.5 (25.4–29.7)
1.3–2.67, n=204	0.48 (0.37–0.58)	0.83 (0.71–0.99)	46.1 (38.2–54.4)	0.71 (0.63–0.77)	0.57 (0.54–0.63)	32.0 (30.0–34.4)
>2.67, n=87	0.85 (0.78–0.90)	3.68 (2.68–4.88)	64.2 (51.1–73.9)	0.90 (0.85–0.94)	3.07 (1.91–4.28)	47.4 (39.3–52.0)
APRI strata						
<0.5, n=382	0.70 (0.64–0.75)	0.16 (0.14–0.17)	47.4 (44.2–52.3)	0.82 (0.79–0.85)	0.11 (0.10–0.12)	35.8 (34.1–38.3)
0.5–1.5, n=129	0.41 (0.26–0.55)	0.67 (0.52–0.80)	73.7 (61.5–85.4)	0.69 (0.58–0.78)	0.44 (0.38–0.48)	53.7 (48.1–59.3)
>1.5, n=25	0.79 (0.59–0.90)	1.88 (1.07–2.32)	68.1 (43.2–85.1)	0.85 (0.68–0.93)	1.65 (0.90–2.52)	45.7 (29.7–57.3)
LiverPRO-F2 strata						
<25%, n=155	0.84 (0.79–0.88)	7.3 (6.5–8.4)	55.4 (49.1–61.4)	0.87 (0.82–0.90)	6.3 (5.6–7.6)	41.8 (38.5–45.9)
25%–65%, n=244	0.76 (0.70–0.81)	16.3 (14.2–18.4)	42.6 (34.4–50.3)	0.88 (0.85–0.91)	11.0 (10.1–12.1)	26.6 (24.6–28.7)
>65%, n=137	0.76 (0.67–0.82)	12.9 (10.3–14.8)	17.3 (13.6–20.1)	0.89 (0.85–0.92)	8.3 (7.3–9.3)	10.5 (9.4–11.6)
LiverPRO-F3 strata						
<15%, n=372	0.65 (0.59–0.71)	6.8 (5.7–7.8)	101.6 (93.6–113.1)	0.81 (0.77–0.84)	4.9 (4.3–5.6)	74.7 (69.9–79.8)
15%–65%, n=109	0.59 (0.43–0.70)	30.2 (26.1–33.8)	96.8 (86.2–107.7)	0.65 (0.51–0.76)	27.7 (24.7–30.5)	78.7 (70.9–86.3)
>65%, n=55	0.39 (0.15–0.59)	25.9 (21.1–31.2)	35.2 (28.3–43.0)	0.57 (0.34–0.73)	19.7 (16.3–22.9)	25.5 (21.0–29.7)
LiverPRO-F4 strata						
<5%, n=264	0.36 (0.25–0.46)	3.0 (2.6–3.5)	100.5 (90.4–114.2)	0.65 (0.57–0.72)	1.9 (1.7–2.2)	71.1 (64.8–79.1)
5%–15%, n=136	0.10 (−0.07 to 0.26)	10.1 (8.9–11.8)	115.4 (103.8–128.5)	0.35 (0.18–0.49)	7.9 (6.8–8.9)	91.7 (82.3–100.9)
>15%, n=136	0.76 (0.67–0.83)	32.0 (28.2–36.5)	98.0 (82.6–109.9)	0.85 (0.77–0.90)	25.3 (23.0–26.9)	69.3 (61.5–76.6)
CORE strata^a^						
<0.4%, n=196	0.64 (0.55–0.72)	0.2 (0.1–0.2)	69.5 (61.4–76.5)	0.81 (0.75–0.85)	0.1 (0.1–0.1)	51.6 (47.5–56.3)
0.4%–5%, n=210	0.66 (0.56–0.73)	2.0 (1.7–2.4)	105.1 (93.8–115.1)	0.78 (0.71–0.83)	1.6 (1.4–1.8)	79.6 (74.1–85.9)
>5%, n=42	0.81 (0.68–0.89)	9.1 (6.7–10.7)	93.6 (68.2–111.4)	0.86 (0.75–0.93)	7.8 (5.9–9.5)	62.3 (51.3–72.1)

*Note:* Complementary Bland–Altman plots are provided in Supplemental Figure S1, https://links.lww.com/HC9/C512. To mitigate the influence of outliers, an additional analysis provides optimistic estimates in which 10% of the cohort with the most extreme relative differences between measurements 1 and 2 were excluded. Estimates are shown for the pooled cohort and within clinically relevant biomarker ranges. Only NITs that were available in at least 2 cohorts were included.

^a^ Only assessed n=448 patients in cohorts from secondary care.

Abbreviations: 95% CI, 95% confidence interval; APRI, AST-to-platelet ratio index; AST, aspartate aminotransferase; CI, confidence interval; FIB-4, Fibrosis-4 score; ICC, intraclass correlation coefficient; MDC, minimal detectable change; MDC%, minimal detectable change (relative scale).

**FIGURE 2:**
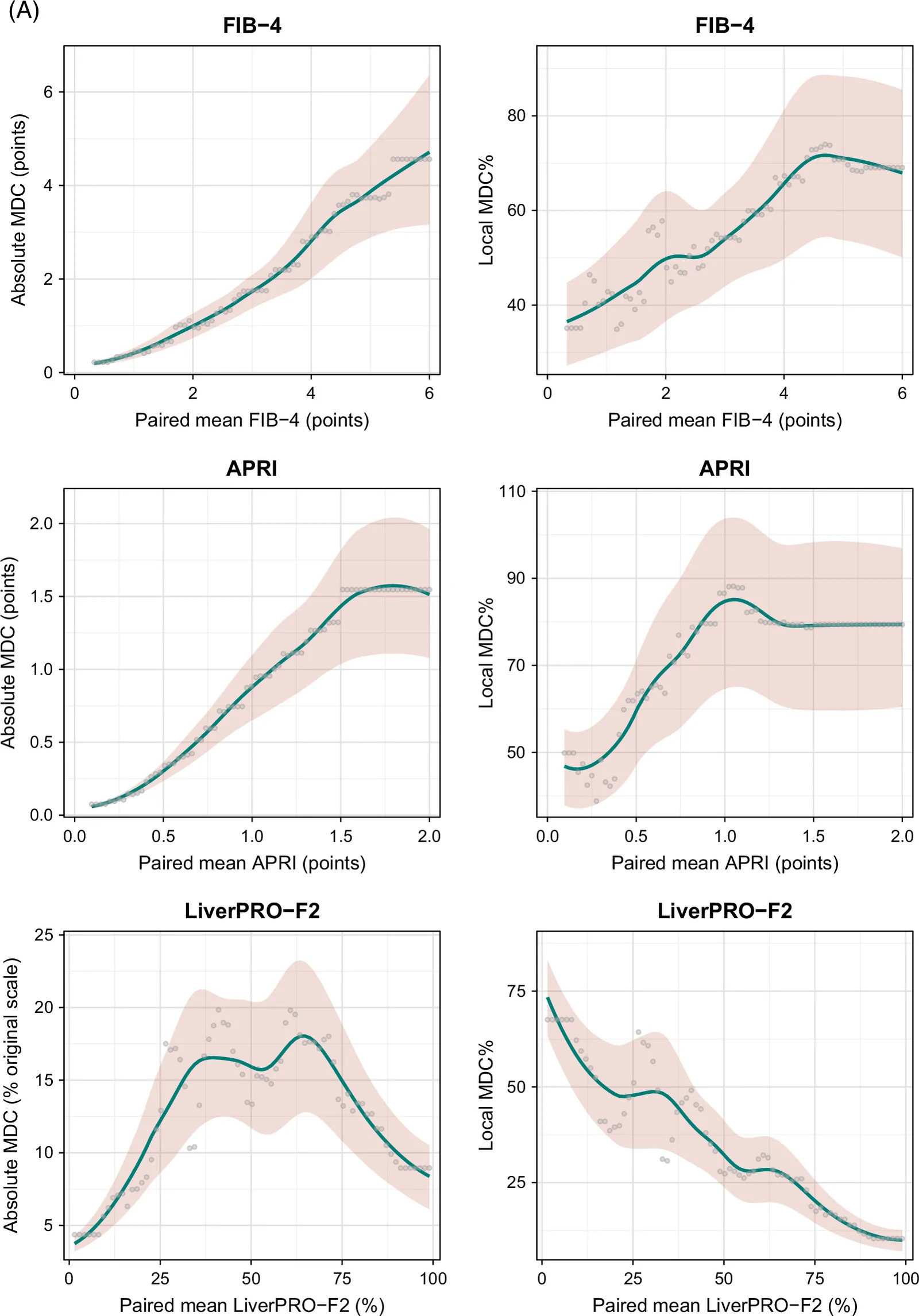
Local MDC and relative MDC% were estimated across the observed range of each NIT using a fixed-grid nearest-neighbor approach. For each biomarker, the left column shows the local absolute MDC on the original scale and the right column shows the corresponding local MDC%. The *x*-axis represents the paired mean of the 2 measurements. Gray points indicate the unsmoothed local MDC estimates at each grid point. The blue line indicates the smoothed local estimate, and the shaded ribbon indicates the smoothed bootstrap 95% confidence interval. FIB-4 and APRI are shown as points, whereas LiverPRO and CORE are shown on the original percentage scale. Individual high-resolution plots are provided as Supplemental Figure S2, https://links.lww.com/HC9/C512. Abbreviations: APRI, AST-to-platelet ratio index; AST, aspartate aminotransferase; FIB-4, Fibrosis-4 score; MDC, minimal detectable change; MDC%, minimal detectable change on the relative scale; NIT, non-invasive test.

**FIGURE 2:**
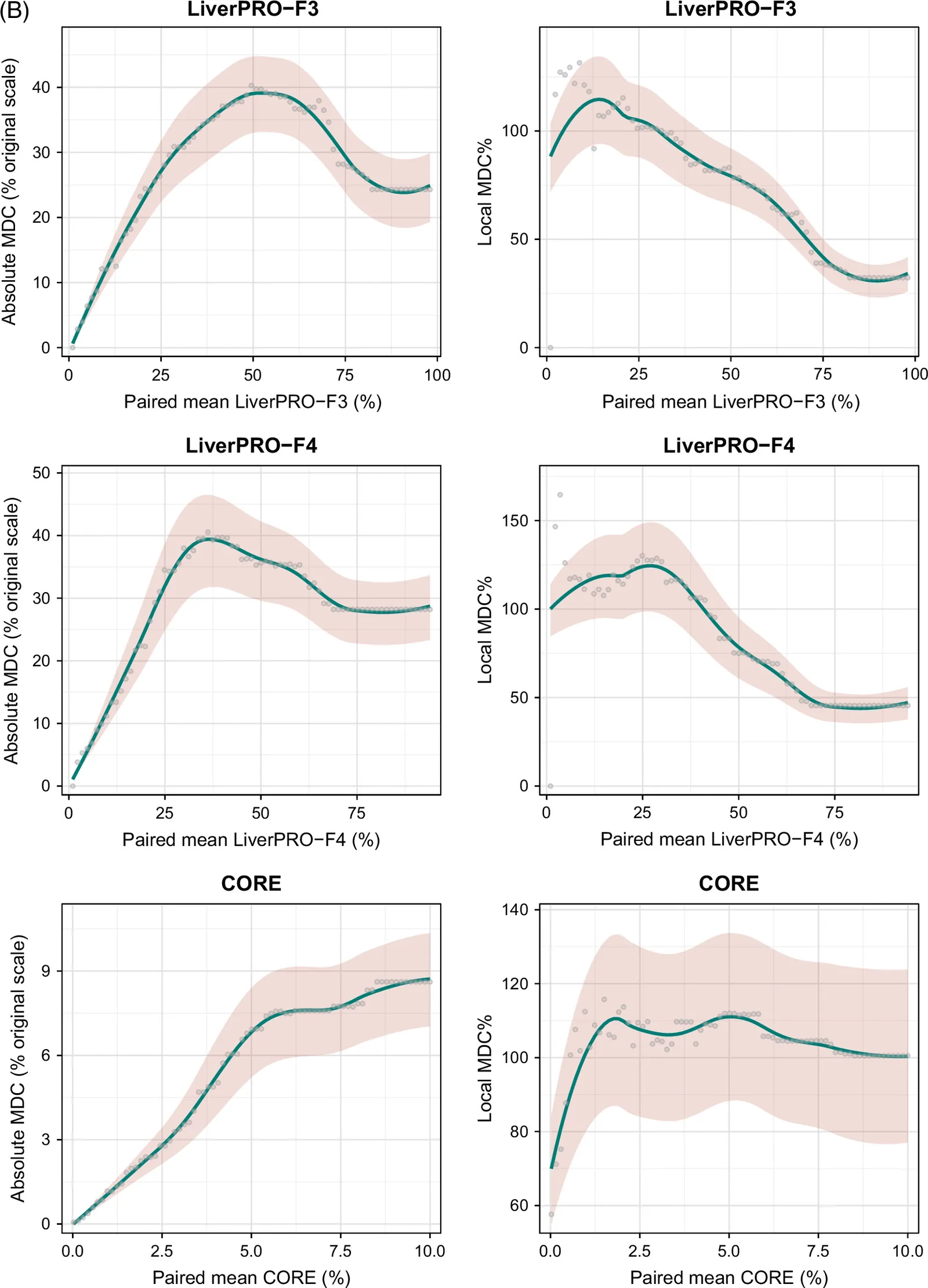
(Continued)

### Part C: Exploring the impact of scale on variability

LSM is interpreted in kPa, yet derived from shear-wave speed measured in m/s. To assess how the choice of scales influences apparent variability, we analyzed pooled data from Repeatability cohort I and Repeatability cohort II. Interpreting change on the original kPa scale not only amplifies heteroscedasticity of measurement differences (Figure 1) but would also result in a broader MDC/MDC% (Supplemental Table S1, https://links.lww.com/HC9/C512). Importantly, relative MDC% in m/s is always ~50% smaller, as kPa is proportional to the square of m/s (Supplemental Figure S3A, https://links.lww.com/HC9/C512).

In addition, symmetric variability on the m/s scale translates into asymmetric changes on the kPa scale, indicating that separate consideration of upward and downward MDC thresholds is required when interpreting LSM dynamics on the kPa scale. One method would be back-transformation according to Obuchowski (2018).^19^ As shown in Supplemental Figure S3B, https://links.lww.com/HC9/C512, this resulted in a logMDC of 0.491 (95% CI: 0.381–0.605), translating into a −39 to +63% window on the relative scale in which measurement variability cannot be excluded. At 8 kPa, any change outside −3.1 to +5.1 kPa would exceed the estimated measurement-variability window, while this would be −5.8 to +9.5 kPa at 15 kPa and −9.7 to +15.9 kPa at 25 kPa, respectively.

To further explore the impact of scale choice on MDC and variability for probability-based NITs, we calculated the MDC for LiverPRO-F2, LiverPRO-F3, LiverPRO-F4, and CORE after logit transformation and subsequently back-transformed these estimates to the original probability scale (Figure 3), and Supplemental Figure S4, https://links.lww.com/HC9/C512, Supplemental Table S3, https://links.lww.com/HC9/C512). This allowed us to express logit-scale measurement variability as baseline-dependent MDC windows on both absolute and relative scales. The resulting analyses demonstrate that the window of change in which measurement variability cannot be excluded is asymmetric and strongly dependent on the baseline probability. For example, a LiverPRO-F2 value of 10 percentage points corresponded to a back-transformed MDC window of ~5–19 percentage points, whereas the corresponding window was 32–68 percentage points at ~50 percentage points. Larger logit-scale MDCs for LiverPRO-F3 and LiverPRO-F4 translated into wider probability-scale windows, particularly at low and intermediate baseline probabilities. These analyses therefore support the concept that dynamics of probability-based NITs should not be interpreted as fixed absolute or fixed relative changes across the full probability range, but rather as scale-dependent, baseline-dependent changes.

**FIGURE 3:**
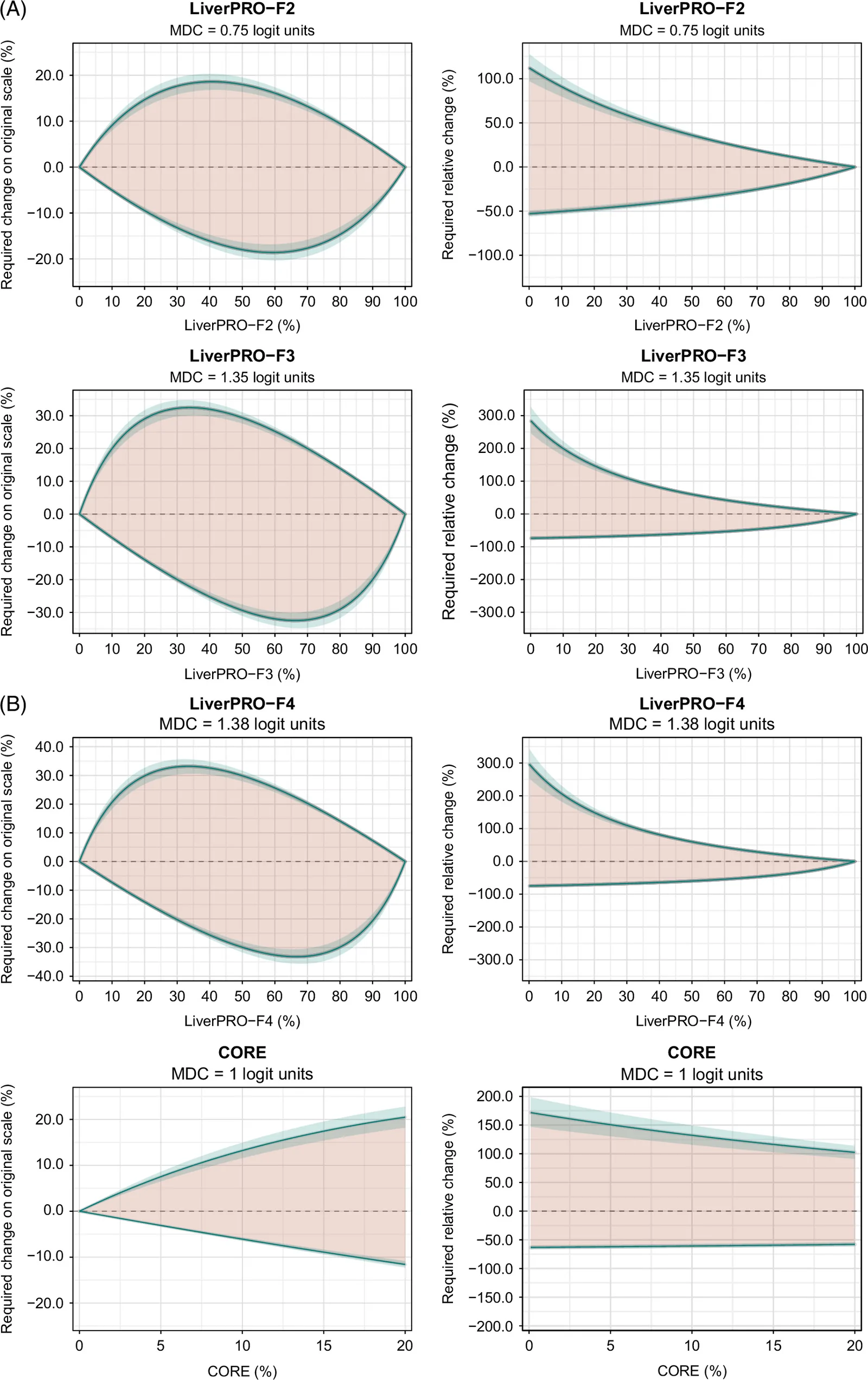
MDC estimates for LiverPRO-F2, LiverPRO-F3, LiverPRO-F4, and CORE were calculated on the logit scale and subsequently back-transformed to the original probability scale. The *x*-axis represents the baseline probability (%). The *y*-axis represents the signed change corresponding to the MDC, with positive values indicating the required increase and negative values indicating the required decrease. For each NIT, the left panel shows the MDC window on the absolute probability scale, whereas the right panel shows the corresponding relative change from baseline. The area between the upper and lower MDC thresholds represents the window of change in which measurement variability cannot be excluded. Dark blue lines indicate the MDC-based point estimate and shaded areas indicate the corresponding 95% confidence interval. For CORE, the *x*-axis was restricted to 0%–20%. Individual high-resolution plots are provided as Supplemental Figure S4, https://links.lww.com/HC9/C512, and associated data as Supplemental Table S3, https://links.lww.com/HC9/C512. Abbreviations: MDC, minimal detectable change; NIT, non-invasive test.

### Part D: Confirming elevated LSM in clinical practice

Selecting patients for repeated LSM based on an elevated first result is frequently done in clinical practice and research. However, this inherently introduces RTM, a phenomenon that has not received the attention it deserves. Thus, we set out to characterize the dynamics of LSM and derived scores (Agile 3+, Agile 4) and associated RTM within 2 settings: patients being referred to liver biopsy after fibrosis was suspected (Reproducibility cohort II), and patients being referred to HVPG measurement after portal hypertension was suspected (Reproducibility cohort III). In both cohorts, all 3 metrics decreased: LSM by a median of −1.8 kPa (−19%) and −2.1 kPa (−9.2%), Agile 3+ by −7.2 (−22%) and −0.1 percentage points (−0.1%), and Agile 4 by −1.5 (−41%) and −0.3 percentage points (−0.3%), in line with higher and plateauing Agile 3+ and Agile 4 values in Reproducibility cohort III (Supplemental Table S4, https://links.lww.com/HC9/C512). When stratified by quartiles, a more pronounced relative decrease in LSM was evident across higher LSM values/quartiles for Reproducibility cohort II (−12% to −26%), with heterogeneous results for Reproducibility cohort III, yet a low number of subjects within each quartile.

Next, we quantified RTM in these 2 specific settings, applying both a parametric approach and an empirical one based on the distribution of LSM, Agile 3+, and Agile 4 in the background populations of both cohorts (Supplemental Table S5, https://links.lww.com/HC9/C512). Importantly, analyses were performed on the m/s scale for LSM and the logit scale for Agile 3+ and Agile 4.

For LSM, both approaches indicated that a substantial proportion of the observed decrease after repeat testing was compatible with RTM (Figure 4, Table 4). In Reproducibility cohort II, parametric RTM accounted for 50%–74% of the observed LSM decrease across kPa-equivalent thresholds from ≥6 to ≥25 kPa, whereas empirical RTM accounted for 63%–107%. Importantly, values exceeding 100% indicate that the estimated decline attributable to RTM was larger than the observed decline. In Reproducibility cohort III, parametric RTM accounted for 62%–71% of the observed LSM decrease, whereas empirical RTM accounted for 91%–103%, respectively. Accordingly, residual changes after empirical RTM adjustment were close to zero in both cohorts, indicating that most of the observed decline after repeat testing could be attributed to RTM.

**FIGURE 4:**
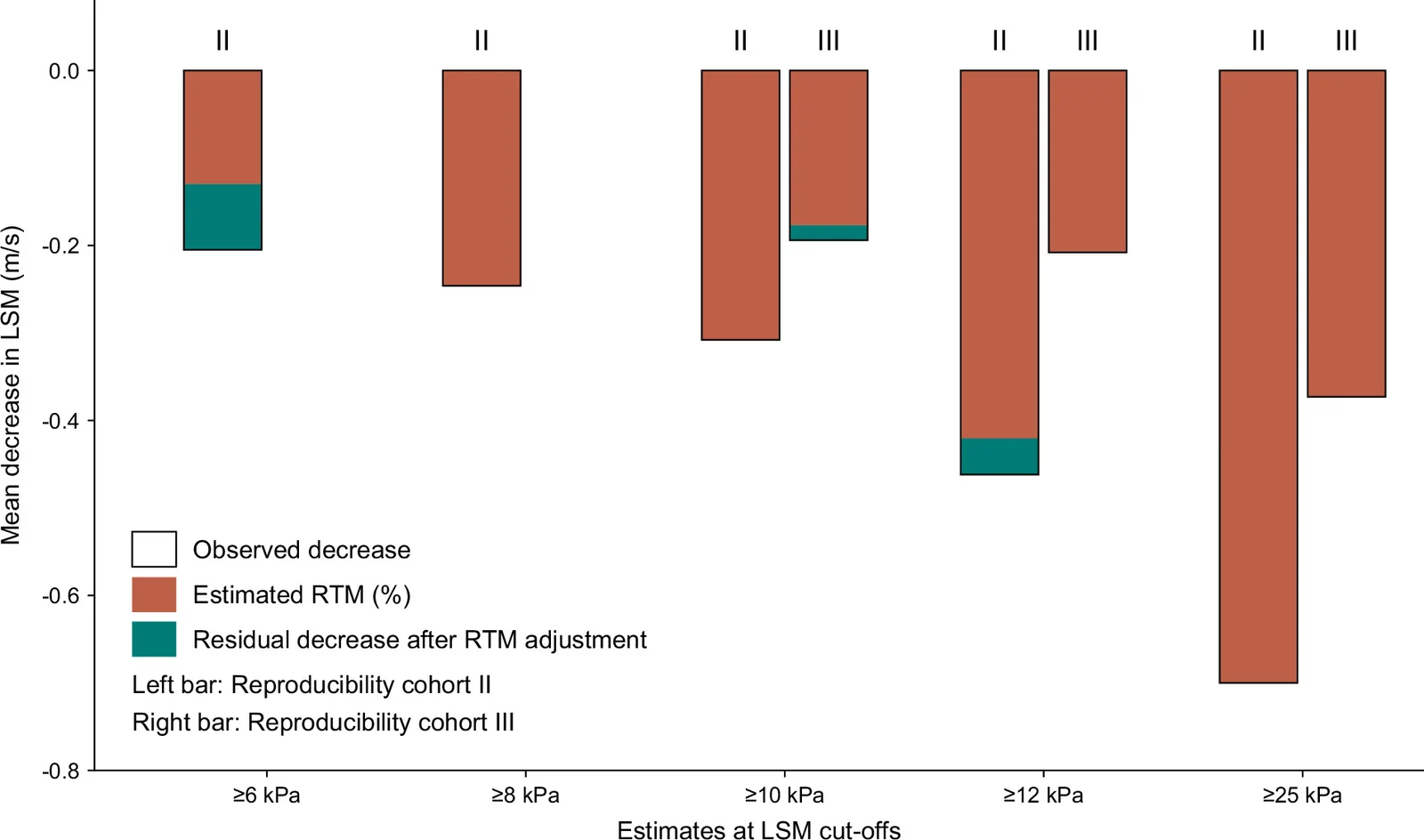
Estimated contribution of regression to the mean to the observed decline in LSM after repeat testing of patients referred to liver biopsy (Reproducibility cohort II) or HVPG measurement (Reproducibility cohort III). Bars show the mean observed change in LSM on the shear-wave speed scale at clinically relevant LSM thresholds. The gold segment shows the proportion of the observed decrease attributable to empirical RTM, while the dark blue segment shows the remaining residual decrease after RTM adjustment. For visualization, empirical RTM estimates exceeding the observed decrease were capped at the magnitude of the observed decrease. Exact estimates are provided in Table 4. Abbreviations: HVPG, hepatic venous pressure gradient; LSM, liver stiffness measurement; RTM, regression to the mean.

**TABLE 4:** Part A shows RTM estimates for LSM at clinically relevant kPa-equivalent thresholds while performing the RTM calculation on the shear-wave speed scale (m/s). Part B shows RTM estimates for Agile 3+ and Agile 4, calculated on the logit scale and subsequently back-transformed to the original probability scale for clinical interpretation

A—LSM	N above cutoff	Observed change (m/s)	Parametric		Empirical				
			RTM (m/s)	% explained	RTM (m/s)	% explained	Residual change after accounting for RTM (m/s)	N above cutoff (background population)	
Reproducibility cohort II									
≥6 kPa (1.41 m/s)	386	−0.21	−0.10	49.6	−0.13	63.2	−0.08	1179	
≥8 kPa (1.63 m/s)	289	−0.25	−0.16	62.8	−0.25	101.1	0.003	402	
≥10 kPa (1.83 m/s)	149	−0.31	−0.21	66.9	−0.33	107.0	0.02	234	
≥12 kPa (2.00 m/s)	70	−0.46	−0.25	55.0	−0.42	91.0	−0.04	140	
≥25 kPa (2.89 m/s)^a^	17	−0.70	−0.52	73.7	−0.71	101.6	0.01	37	
Reproducibility cohort III									
≥10 kPa (1.83 m/s)	88	−0.19	−0.12	61.4	−0.18	91.1	−0.02	572	
≥12 kPa (2.00 m/s)	80	−0.21	−0.14	67.7	−0.21	103.1	0.006	447	
≥25 kPa (2.89 m/s)	52	−0.37	−0.27	71.1	−0.38	101.1	0.004	160	
**B—Agile 3+/4**	**N above cutoff**	**Baseline prob. anchor (percentage points)**	**Observed change (percentage points)**	**Parametric**		**Empirical**			
				**RTM (percentage points)**	**% explained**	**RTM (percentage points)**	**% explained**	**Residual change after accounting for RTM (percentage points)**	**N above cutoff (background population)**
Reproducibility cohort II									
Agile 3+ ≥45.1%	173	74.0	−15.2	−9.3	63.8	−12.3	82.9	−2.3	302
Agile 3+ ≥67.9%	75	89.0	−9.1	−7.3	84.1	−10.2	109.7	0.7	127
Agile 4 ≥25.1%	44	54.6	−20.8	−24.6	120.8	−28.8	145.0	9.3	72
Agile 4 ≥56.5%^a^	17	79.8	−14.4	−28.9	181.2	−33.7	207.5	9.9	29
Reproducibility cohort III									
Agile 3+ ≥45.1%	85	97.6	−0.7	−1.1	150.8	−1.4	186.1	0.5	610
Agile 3+ ≥67.9%	82	97.8	−0.6	−1.3	185.6	−1.7	231.7	0.6	404
Agile 4 ≥25.1%	79	80.9	−1.5	−9.5	552.0	−11.4	642.9	6.8	324
Agile 4 ≥56.5%	61	87.9	−2.1	−9.2	365.3	−10.3	401.9	4.8	187

*Note:* For each threshold, the table reports the observed mean change between the first and second measurement, the expected RTM effect estimated using a parametric reference-distribution approach, and the expected RTM effect estimated directly from the empirical reference distribution. Specifically, the parametric approach estimates the expected mean of the cutoff-selected reference subgroup assuming a normal distribution on the analyzed scale, whereas the empirical approach estimates this mean directly from observed reference values above the respective cutoff. The RTM-adjusted change represents the observed change minus the expected RTM effect. The percentage of the observed change explained by RTM was calculated as the expected RTM divided by the observed change. Values above 100% indicate that the expected RTM effect exceeded the observed change. For Agile scores, logit-scale observed changes, expected RTM effects, and RTM-adjusted changes were back-transformed to probability-point changes at the selected subgroup’s mean baseline probability. Thus, Part B should be interpreted as the estimated change in Agile-predicted probability at the typical baseline probability of the cutoff-selected subgroup.

^a^ Estimates should be interpreted with caution due to the low number of patients in the retest subset and the background population

Abbreviations: Agile 3+, Agile 3+ score; Agile 4, Agile 4 score; LSM, liver stiffness measurement; N, number; NIT, non-invasive test; prob., probability; RTM, regression to the mean.

For Agile 3+ and Agile 4, RTM also explained a substantial proportion of the observed decrease. In Reproducibility cohort II, empirical RTM similarly explained 83%–110% of the observed Agile 3+ decrease, leaving little residual change after adjustment. For Agile 4, the expected empirical RTM exceeded the observed decrease at both cutoffs. In Reproducibility cohort III, Agile 3+ and Agile 4 were both highly saturated at baseline, observed probability-point changes were small, and expected empirical RTM effects exceeded the observed decreases for both scores.

In summary, when patients are remeasured because the first LSM was elevated, a decrease at the second measurement is expected even without true biological improvement. This expected decrease is substantial and explains most, if not all, of the observed decrease between paired LSM.

### Sensitivity analyses

Finally, we performed sensitivity analyses for key measures of variability (ICC, MDC, MDC%) within Reproducibility cohorts I–III in the subgroups of patients with repeated measurements within 28 days (Supplemental Table S6, https://links.lww.com/HC9/C512). In brief, ICC slightly improved and MDC/MDC% were narrower for lab-based NITs in Reproducibility cohorts I and III, while estimates excluding outliers remained nearly unchanged. Estimates in Reproducibility cohort II remained unchanged or even worsened. Estimates for LSM, Agile 3+, and Agile 4 were comparable in Reproducibility cohort I.

## DISCUSSION

NITs are broadly used by clinicians and scientists to infer disease progression, regression, or treatment response. However, when diagnostic and prognostic biomarkers are repurposed for monitoring, their random variation is barely considered, and data on precision remain limited, as acknowledging the inherent uncertainty in these surrogates complicates their interpretation. In this study, we show that the variability of NITs is not a statistical nuisance but must be considered when using them as monitoring tools. Across same-day repeatability and short-term reproducibility settings, we found good to excellent agreement, but considerable variability for all investigated biomarkers. We show that a pooled MDC is not a fixed property of a given NIT. Instead, it depended on biomarker level, clinical setting, and measurement scale. These findings challenge the common practice of applying universal change thresholds across different patient populations and biomarker ranges in both clinical and scientific practice. Key challenges are discussed below and in the Supplement, https://links.lww.com/HC9/C512.

### Pooled MDCs are often misleading and inaccurate

A key takeaway from the current analysis is the large variation in MDC and MDC% estimates between settings and cohorts. We started off with MDC estimates without further transformations, because this reflects how NIT dynamics are currently interpreted in clinical practice and research. However, our more granular analyses demonstrate why this approach is problematic, as variability estimates vary considerably between settings and biomarker ranges, with the absolute biomarker level appearing to be a major determinant. Consequently, a change threshold derived from a low-risk screening cohort should not be uncritically transferred to patients with more advanced disease managed in tertiary care, and vice versa. In contrast, the MDC should be interpreted as a level-dependent estimate of expected variability within a given setting,^18^ and applying one cutoff as a universal rule for monitoring should be avoided. For example, the MDC for FIB-4 increased from 0.6 points (38%) in the screening setting to 1.1 (45%) in those with suspected fibrosis and 3.2 (59%) in those with suspected portal hypertension.^20^ A relative threshold of 38% derived from a screening cohort should therefore not be generalized to patients with more advanced disease. Nevertheless, our findings consistently indicate that changes in FIB-4 below 30% cannot be distinguished confidently from measurement variability at the individual-patient level.

### Good agreement does not imply high precision

A second important observation is that good agreement does not equal negligible within-patient variability. ICCs were generally good to excellent, particularly for composite lab-based scores, but MDCs remained considerable. This apparent discrepancy can be explained: ICCs primarily reflect whether patients maintain their relative ranking within a cohort, whereas MDC quantifies how large an individual change must be to exceed expected variability. This also explains why a biomarker may retain diagnostic and prognostic discrimination at a population level: Maintaining rank order or remaining above or below a certain threshold is affected by measurement variability to a far lesser degree than monitoring changes.

### Nonlinear properties of biomarkers have greater consequences for monitoring than for diagnosis and prognostication

Along the same lines, the mathematical framework from which NITs are derived has major implications for their use as monitoring tools. LSM by VCTE is clinically interpreted in kPa but derived from shear-wave speed through a quadratic transformation. Therefore, changes in shear-wave speed do not translate linearly into changes in kPa. Probability-based NITs pose a similar challenge, as linear predictors from logistic regression or Cox models are non-linearly transformed into probabilities or absolute risks. This is supported by our stratified analyses demonstrating level-dependent MDC estimates for LSM and probability-based scores, which are discussed in detail below.

### Measurement variability affects all biomarkers

Our findings underscore that measurement variability is not limited to elastography or composite scores but is inherent to every biomarker measurement. Even standard laboratory parameters showed relevant short-term variation, reflecting both analytical and biological variability (compare^9^ and the respective online calculator). While this is not regularly considered in clinical practice and depends on the laboratory tests used, we demonstrate that an MDC% of 10% at minimum, but rather of 30%–50%, also applies to standard laboratory tests.^9^ Here, composite scores may show some degree of robustness against outliers in single parameters but may likewise be impacted by significant biological or analytical variation in one single component.

### The measurement scale of LSM needs specific attention

Although shear-wave speed in m/s and liver stiffness in kPa are mathematically related, this relationship is not linear: Since kPa is proportional to the square of shear wave speed following the formula kPa = 3 × (m/s)^2^, absolute MDC values become level-dependent after transformation, and symmetric relative changes on the m/s scale translate into asymmetric relative changes on the kPa scale.^21^ For example, a ±30% change in m/s corresponds to a −51% to +69% change in kPa. Applying a symmetric 30% threshold directly to the kPa scale therefore underestimates measurement variability and ignores the asymmetry between increases and decreases. This scale dependency was evident in our analysis. An MDC% of ~25% for LSM on the m/s scale corresponded to a back-transformed window of roughly −44% to +56% on the kPa scale, while the log-transformation-derived MDC yielded a comparable window of −39% to +63%, suggesting that these methods arrive at similar estimates. Awareness of this scale dependency is important when defining clinically meaningful change thresholds and when comparing variability across the spectrum of LSM.

### The MDC of LSM by VCTE

Compared with the number of studies applying VCTE in clinical and scientific practice, the data on variability falls surprisingly short, even though it is still the best investigated biomarker in hepatology. While the best evidence regarding test–retest reliability of elastography stems from magnetic resonance elastography (MRE) and showed an MDC% of >19%,^22^,^23^ recent studies on LSM by VCTE indicated an MDC% (same day, same operator) of 38% and an MDC% (different day, different operator) of 70% on the kPa scale.^21^ This is in line with our study, suggesting an MDC% of −43 to +54% (same-day, same operator), while our longitudinal study suggested a smaller difference (−32 to +38%), yet being restricted to early disease patients with low kPa values. Importantly, the inherent increase in variability when studying more advanced patients has been reported before (MDC% of 54% in patients with cirrhosis),^20^ again supported by current stratified analyses and elaborations on measurement scale. Taken together, our findings and the available literature indicate that changes of <30% between paired measurements often remain within the range of expected measurement variability. Such changes should therefore be interpreted cautiously and should not, in isolation, be assumed to represent clinically meaningful change.

### Biomarkers based on probabilities come with additional challenges

Probability-based scores require specific consideration when used for monitoring. Unlike unbounded continuous biomarkers, probabilities are restricted to values between 0% and 100% and result from a nonlinear transformation of an underlying linear predictor. Consequently, changes on the reported probability scale are not directly comparable across its full range. A one-percentage-point change from 1% to 2%, from 50% to 51%, or from 98% to 99% appears identical on the probability scale but represents different changes on the underlying scale. Near 0% and 100%, the probability scale is compressed, such that substantial changes in the underlying predictor may translate into only small absolute probability-point changes.

Relative changes are particularly difficult to interpret at low baseline probabilities. For example, an increase from 1% to 2% represents a 100% relative change, although the absolute change is only one percentage point and both measurements continue to indicate a very low probability. Accordingly, a high MDC% at the lower end of the probability range does not necessarily indicate poor measurement precision or a clinically relevant change. Instead, it may largely reflect the small denominator used to calculate relative change.

This issue also highlights the importance of applying biomarkers in populations that are appropriate for their intended use. For example, LiverPRO-F4 provides limited information for monitoring in a low-risk cohort in which most participants have predicted probabilities close to 1%. In this setting, relative changes may appear large despite negligible changes in absolute probability, while the score provides little differentiation between patients. Conversely, a score developed to detect less advanced fibrosis, such as LiverPRO-F2, may provide limited additional information in cohorts enriched for advanced disease, where probabilities are clustered near the upper boundary.

Our stratified and nonlinear analyses of LiverPRO and CORE further illustrate this baseline dependence. Absolute variability was greatest in the intermediate probability range, whereas clustering near 0% or 100% resulted in small probability-point changes. Neither fixed absolute percentage-point thresholds nor fixed relative MDC% thresholds should therefore be applied uniformly across the full probability range. Estimating variability on the logit scale and back-transforming it to the probability scale provides a more appropriate means of deriving baseline-dependent intervals within which measurement variability cannot be excluded.

### RTM is a common challenge in monitoring

The phenomenon of RTM has been known for a long time but is often overlooked in clinical research and practice. The strong reliance on VCTE provides an excellent example of this. Scientists and clinicians may use an elevated initial LSM to determine whether patients (i) remain in hepatology care, (ii) undergo additional diagnostic procedures, (iii) receive a repeated LSM, or (iv) start a treatment. Repeating LSM in these scenarios needs attention, as they are prone to RTM. When repeat testing is conditional on an elevated initial result, the second measurement is expected to be lower on average, even in the absence of any true biological improvement. This occurs because the initial extreme value may partly reflect random measurement variation, which is less likely to recur to the same extent at the subsequent assessment. This expected decline is particularly relevant when treatment efficacy is evaluated using paired LSM. If treatment is initiated based on an elevated baseline LSM, a lower follow-up value may be attributed to treatment response even though part, or potentially most, of the decline is explained by RTM.

Nevertheless, decreases in LSM are frequently reported in the medical literature and are often associated with a lower subsequent risk of liver-related events. However, it remains important to determine the extent to which an observed decrease reflects true improvement in fibrosis rather than regression of an initially extreme measurement toward its expected value. Assuming some degree of RTM, this could include patients with profound decline who had neither any actual risk at first or second measurement and overestimate the “protective” effect of decreases in LSM. RTM may also affect interventional trials, in which participants are selected based on an elevated first LSM. In such settings, apparent treatment and placebo responses may be (unsurprisingly) greater for LSM than for other fibrosis biomarkers. Similarly, high screening failure rates may arise when an elevated initial LSM cannot be confirmed by repeat measurement.

Barnett et al^24^ suggest a simple approach to mitigate RTM: selecting subjects based on 2 or more baseline measurements, and either selecting them based on the paired mean or the later measurement. In general, repeating measurements and interpreting the subject’s mean mitigates the RTM effect, with the strongest reduction of this effect by performing a second measurement.^24^

By highlighting the decrease in LSM and the magnitude of RTM in the settings of referral to liver biopsy or HVPG measurement, we want to emphasize that physicians need to be made aware of this phenomenon when decisions regarding monitoring, a medical intervention (eg, biopsy, HVPG measurement), or treatment are based on a single elevated index test that is subsequently used to judge disease dynamics and treatment effects.

### What this study does not show

This study does not assess the diagnostic accuracy of NITs, determine how frequently changes exceeding MDC occur, or establish which magnitude of change is clinically meaningful. Because only 2 measurements were analyzed per patient, we did not evaluate whether additional measurements or longitudinal trajectories provide incremental diagnostic or prognostic information.

### Limitations

Between-day estimates reflect reproducibility because testing conditions, the underlying disease state, and time-varying factors affecting NITs may have changed between measurements. While this cannot be fully accounted for even in biopsy-controlled studies, fibrosis is very unlikely to change within 90 days, and subgroup analysis limiting data to repeated measurements obtained within 28 days showed comparable results. However, all analyses in this manuscript assume that the ground truth has not changed in between. Second, the low number of paired measurements within certain strata of investigated biomarkers in stratified analyses needs to be considered. The same applies to same-day or different-day LSM analyses that were insufficient to estimate local MDC and MDC%.

## CONCLUSIONS

We aimed to highlight key challenges for monitoring liver disease within the current NIT framework and to support appropriate interpretation in clinical practice and research. Until further evidence arises, the following principles may be applied:

1. Repeated measurements increase confidence in the direction and magnitude of change. However, changes between paired measurements are prone to overinterpretation if inherent measurement variability is not considered.
2. Confirming a biomarker result at a second time point informs on within-subject variation and reduced bias from RTM. Before important clinical decisions are made, the repeated value or the mean of repeated baseline measurements should be considered rather than relying solely on a single result.
3. The measurement scale of LSM (kPa vs. m/s) affects the MDC and is highly relevant for monitoring purposes. When looking at paired measurements, changes below 30% should not be interpreted confidently as true change. Depending on the clinical setting and measurement scale, even larger changes remain within expected measurement variability.
4. The relative minimal detectable change for any biomarker is expected to be at least 10%, but is often higher.
5. Biomarkers based on probabilities provide easily interpretable estimates but need specific attention when applied for monitoring. Their nonlinear nature needs to be considered. Changes must be interpreted in relation to the absolute probability across the measurement scale.

## Supplementary Material
