# Supplementary material for: Variability in non-invasive tests: Challenges and implications for monitoring liver disease
Source: Hepatol Commun. 2026 Nov 11;10(12):e01077. doi: 10.1097/HC9.0000000000001077 (PMC13766574; doi:10.1097/HC9.0000000000001077)
Supplement: Supplementary file 1 [file hc9-10-e01077-s001.pdf]

SUPPLEMENT

SUPPLEMENTARY METHODS

Extended cohort descriptions of repeatability cohorts (same day)

Repeatability cohort I - LSM: 54 individuals with metabolic dysfunction and suspicion of hepatic steatosis presenting at the outpatient clinic of the Sanatorium Hera, a municipal healthcare and prevention center for state employees in Vienna (Austria) were included. Notably, all patients were screened for participation in a prospective lifestyle intervention, and patients with moderate or severe alcohol consumption were excluded as per the study protocol (1). All patients underwent repeated VCTE on the same day within the same examination.

All VCTE measurements were done on the same machine (FibroScan® 502 Touch) by two operators with extensive experience in VCTE (>100 measurements before study initiation) and having received certified training by Echosens.

Repeatability cohort II - LSM: 58 patients with suspected SLD undergoing same-day LSM in the SLD outpatient clinic of the General Hospital Vienna (Austria). This sample included all consecutive patients presenting between July and September 2025. LSM was performed at the beginning of the consultation and repeated at the very end by the same operator.

All VCTE measurements were done on the same machine (FibroScan® Expert 630) by the same operator with extensive experience in VCTE (>100 measurements before study initiation) and having received certified training by Echosens.

Repeatability cohort III - Lab-based NITs and standard laboratory tests: 89 patients with suspected SLD presenting at the outpatient clinic of the General Hospital Vienna (Austria) were included. Patients received two laboratory measurements on the same day due to either (i) organizational aspects (i.e., labs drawn in both the outpatient clinic and the inpatient ward), or (ii) control of hemoglobin levels after invasive procedures (e.g., HVPG measurement, transjugular liver biopsy or percutaneous liver biopsy). Only timepoints within 1 year from first reliable LSM were considered if they did not have any missing values in platelet count, ALT, AST, or INR. Measurements with ALT or AST >5x upper limit of normal, bilirubin >3mg/dL, or a C-reactive protein ≥2mg/dL were excluded.

Extended cohort descriptions of reproducibility cohorts (longitudinal setting)

Reproducibility cohort I - suspected MASLD: 61 individuals with metabolic dysfunction and suspicion of hepatic steatosis presenting at the outpatient clinic of the Sanatorium Hera, a municipal healthcare and prevention center for state employees in Vienna (Austria), were included. Timepoint 1 was the appointment in the outpatient clinic, at which patients were screened for participation in a prospective lifestyle intervention (1). Timepoint 2 was the study initiation visit within 90 days (37 subjects within 28 days). No intervention happened in between. Patients were included between April 2016 and April 2017. According to the original study protocol, patients with moderate or severe alcohol consumption were excluded (1).

All VCTE measurements were done on the same machine (FibroScan® 502 Touch) by two operators with extensive experience in VCTE (>100 measurements before study initiation) and having received certified training by Echosens.

Reproducibility cohort II - suspected fibrosis: 387 participants screened for the presence of SLD within prospective studies at Odense University Hospital (Denmark) were included (2, 3). Timepoint 1 was the first appointment in the outpatient clinic (screening visit), and timepoint 2 was the subsequent referral to liver biopsy when fibrosis was suspected within 90 days (167 subjects within 28 days). Patients were included between 2017 and 2024. The general prerequisite for a second visit was an initial LSM ≥6kPa. Therefore, results on LSM and LSM-derived scores are subject to selection bias due to conditioning on the index test and were therefore not compared to other NITs but used to quantify the degree of 'regression to the mean' that is expected when applying LSM in this setting. Laboratory tests and LSM were performed on the same day or within 7 days from each other.

All VCTE measurements were done on two different machines (FibroScan® 502 Touch, FibroScan® Compact 530) by different operators with extensive experience in VCTE (>100 measurements before study initiation).

A repeated ELF test was available in 118 patients (69 subjects within 28 days).

Reproducibility cohort III - suspected portal hypertension: 88 patients with SLD and suspected portal hypertension presenting to the cirrhosis outpatient clinic of the General Hospital Vienna (Austria) were included (4). Timepoint 1 was the first LSM in the outpatient clinic and timepoint 2 was the subsequent LSM at the HVPG

measurement within 90 days (32 subjects within 28 days). Patients were included between 2014 and 2024. Importantly, only patients with both complete lab and reliable LSM before HVPG obtained at the hospital were included, and n=4 patients with LSM <10kPa were excluded to obtain a homogeneous cohort. Other specific exclusion criteria for this cohort were (i) prior or current hepatic decompensation, (ii) hepatocellular carcinoma, (iii) bilirubin ≥3 mg/dL at any timepoint, (iv) AST/ALT >5x upper limit of normal (ULN) at any timepoint, or (v) C-reactive protein ≥2x ULN at any timepoint. Notably, excluded compensated SLD patients were often referred to with external labs, or did not receive a full lab draw prior to HVPG measurement. Again, the indication for an HVPG measurement was essentially based on the VCTE result, making comparison to other NITs biased.

All VCTE measurements were done on three different machines (FibroScan® 502 Touch, FibroScan® Mini+ 430, FibroScan® Expert 630) by different operators with extensive experience in VCTE (>100 measurements before study initiation).

Ethics

The study received approval by the local Ethics Committee of the Medical University of Vienna (1735/2015, 1917/2024, 1821/2024, 1055/2025) and Odense (S-20170087) and was conducted in accordance with the Declaration of Helsinki. Given the retrospective design of the study, the requirement for informed consent was waived by the ethics committee. However, all patients from repeatability cohort I, repeatability cohort II, reproducibility cohort I, and reproducibility cohort II provided written informed consent to the use of their data.

Non-invasive tests and formulas

<u>FIB-4 and APRI</u>:

$$FIB-4 = \frac{Age \times AST}{PLT \times \sqrt{ALT}}$$

$$APRI = \frac{\frac{AST}{ULN} \times 100}{PLT}$$

For APRI, the ULN of AST was set to 50 U/L for males and 35 U/L for females, respectively.

Agile 3+ and Agile 4:

$$Agile\ 3+ = \frac{e^{-3.92368 + 2.29714 \times \ln(LSM) - 0.00902 \times PLT - 0.98633 \times \frac{ALT}{AST} + 1.08636 \times Diabetes - 0.38581 \times Sex + 0.03018 \times Age}}{1 + e^{-3.92368 + 2.29714 \times \ln(LSM) - 0.00902 \times PLT - 0.98633 \times \frac{ALT}{AST} + 1.08636 \times Diabetes - 0.38581 \times Sex + 0.03018 \times Age}}$$

$$Agile\ 4 = \frac{e^{7.50139 - 15.42498 \times \frac{1}{\sqrt{LSM}} - 0.01378 \times PLT - 1.41149 \times \frac{ALT}{AST} - 0.53281 \times Sex + 0.41741 \times Diabetes}}{1 + e^{7.50139 - 15.42498 \times \frac{1}{\sqrt{LSM}} - 0.01378 \times PLT - 1.41149 \times \frac{ALT}{AST} - 0.53281 \times Sex + 0.41741 \times Diabetes}}$$

Sex: male = 1, female = 0; Diabetes: yes = 1, no = 0; LSM in kPa; PLT in G/L

CORE: As obtained from https://rickstra.shinyapps.io/CORE/ per 01/10/2025.

LiverPRO: Patented score based on at least 3 of the following variables and age: platelet count, INR, AST, ALT, GGT, ALP, bilirubin, cholesterol, sodium, and albumin.

Importantly, the LiverPRO platform provides simultaneous probability estimates ranging from 1 to 99% for the presence of significant fibrosis (≥F2), advanced fibrosis (≥F3) or cirrhosis (F4). For the current analysis, it was required that the same version of LiverPRO was used at timepoints 1 and 2.

For analyses on the logit scale, probabilities were transformed according to:

$$LinearPredictor(\eta) = \log\left(\frac{Probability}{1 - Probability}\right)$$

Statistical methods

Statistical analyses were performed using R 4.6.0 (R Core Team, R Foundation for Statistical Computing, Vienna, Austria). Key metric of agreement included the Intraclass Correlation Coefficient (ICC), Bland-Altman analyses, the Minimal Detectable Change (MDC) on the original scale (i.e., absolute MDC) and relative scale (MDC%). Importantly, the MDC is equivalent to the Repeatability Coefficient (RC or CR) and the Smallest Real Difference (SRD) which are used heterogeneously in medical literature. To maintain clarity and avoid confusion stemming from the interchangeable use of various terms, we consistently used MDC and MDC%

throughout the manuscript. Numbers >10% were rounded to full integer within the manuscript to increase readability. As previous work suggested that sample sizes of approximately 35-65 participants provide reasonably precise estimates in test-retest studies, we considered our cohorts sufficiently large (5). 95% confidence intervals are provided for all estimates using bootstrapping to quantify uncertainty. A p-value <0.05 was considered statistically significant.

Intraclass correlation coefficient (ICC): The ICC represents a relative reliability index corresponding to the ratio of the variance between subjects (i.e. the variance of interest) and the total variance. We calculated $ICC_{agreement}$ using the R "irr" package, as our main interest was absolute agreement between the 2 measurements although we acknowledge that LSM measurements were performed by different operators (6). Values of 0.4-0.5 indicate poor, 0.5-0.75 moderate, 0.75-0.9 good and values >0.9 indicate excellent agreement (6).

$$ICC = \frac{\sigma^2_{between}}{\sigma^2_{between} + \sigma^2_{within}}$$

Bland-Altman analysis: Agreement was assessed according to the Bland-Altman method and respective plots (7). Specifically, bias hereby refers to the central tendency of values being different (mean difference) while the upper and lower limits of agreement define the range in which 95% of all observed differences lie. Additionally, Bland-Altman plots should be visually inspected for systematic patterns in the difference, as these measures are most valid when variability is uniform throughout the range of measurements (7).

Scatterplots: For graphical assessment of agreement, paired measurements were displayed in scatterplots with the first measurement on the x-axis and the second measurement on the y-axis. The line of identity was added to indicate perfect agreement. Reduced major-axis regression lines were fitted using all paired measurements and after exclusion of the 10% with the largest absolute relative differences. Reduced major-axis regression was chosen instead of ordinary least-squares regression because both repeated measurements are subject to measurement variability, and neither time point can be considered an error-free independent variable. The 10% of paired measurements with the largest absolute relative differences were shown in grey to illustrate their influence on the fitted relationship.

Proportional bias and heteroscedasticity were evaluated within the Bland-Altman framework. Proportional bias was assessed by regressing the paired difference on the paired mean. A non-zero slope was interpreted as evidence that the direction or magnitude of the signed measurement difference varied across the measurement range. Heteroscedasticity was assessed by regressing the absolute paired difference on the paired mean, thereby evaluating whether the magnitude of measurement variability increased or decreased with the biomarker level.

Minimal detectable change (MDC): The MDC reflects the degree of change that can be considered not random with 95% confidence. The MDC on the original scale was calculated as follows:

$$MDC = 1.96 \times \sqrt{\frac{\sum(x_{i2} - x_{i1})^2}{N}}$$

where x_1 and x_2 are the two repeated measurements for each subject, and n is the number of subjects. Importantly, this is equivalent to

$$MDC = 1.96 \times \sqrt{2} \times wSD$$

where

$$wSD = \sigma_{within} = \sqrt{\frac{1}{2N}\sum_{i=1}^{N}(x_{i1} - x_{i2})^2}$$

where x_1 and x_2 are the two repeated measurements for each subject, and N is the number of subjects.

To define the relative MDC (expressed as a percentage), the MDC% was derived from the squared within-subject coefficient of variation (wCV²):

$$MDC\% = 1.96 \times \sqrt{2} \times \sqrt{wCV^2} \times 100 = 2.77 \times wCV \times 100$$

where

$$wCV^2 = \frac{1}{N}\sum_{i=1}^{N}\left(\frac{(x_{i1} - x_{i2})^2}{2\bar{x}^2{}_i}\right)$$

and

$$\bar{x}_i = \frac{x_{i1} + x_{i2}}{2}$$

For each pair of repeated measurements, the squared difference was divided by twice the squared mean of the two measurements. These standardized squared differences were averaged across all subjects to obtain the squared within-subject coefficient of variation (wCV^2). Bootstrap resampling of the cohort was used to obtain 95% confidence intervals.

Scale transformations for LSM and probability-based scores: For LSM, MDC and MDC% were primarily estimated on the shear-wave speed scale (m/s), because kPa represents a nonlinear transformation of shear-wave speed according to kPa = 3 × $(m/s)^2$. Absolute m/s-derived MDC values were additionally expressed as kPa-equivalent changes at 8 kPa (Table 2, Supplementary table 2) as well as additional clinically relevant LSM thresholds (Figure 1A), resulting in different thresholds for increases and decreases. Likewise, relative m/s-derived MDC% values were transformed to asymmetric kPa-equivalent percentage changes. Additional analyses included log-transformation prior to calculation of MDC/MDC% to reduce the skewness. For probability-based scores bounded between 0 and 1 (LiverPRO, CORE), MDC analyses were additionally performed on the logit scale and then back-transformed to the original probability scale at the corresponding baseline probabilities. Pooled MDC% was not reported for probability-based scores because relative changes are strongly baseline-dependent and become disproportionately inflated near 0%, making a single summary percentage poorly interpretable across the full probability range.

Locally estimated MDC and MDC%: To explore whether measurement variability differed across the observed biomarker range, local MDC curves were calculated for FIB-4, APRI, LiverPRO-F2, LiverPRO-F3, LiverPRO-F4, and CORE. For each biomarker, a fixed grid of 75 equally spaced values was defined across the observed range of the paired mean. At each grid point, the 50 paired observations with the closest paired mean values were selected, and MDC and MDC% were calculated once within this local window. Bootstrap resampling with 1000 replicates was used only to estimate 95% confidence intervals for each local estimate. For visualization, the local point estimates and confidence limits were smoothed using LOESS.

Regression-to-the-mean (RTM) estimations: RTM describes the tendency of values selected because they are high or low at baseline to be closer to the population mean upon repeated measurement, even in the absence of true biological change (8, 9). RTM was estimated for clinically relevant LSM, Agile 3+, and Agile 4 thresholds. For LSM, calculations were performed on the shear-wave speed scale (m/s) and interpreted at the corresponding kPa-equivalent threshold. For Agile 3+ and Agile 4, calculations were performed on the logit scale and subsequently back transformed to the original probability scale. Two approaches were applied: a parametric approach assuming a normal reference distribution on the analyzed scale (as previously described (8, 9)), and an empirical approach using the observed mean of the reference-distribution tail above the respective cutoff. The RTM-adjusted change was calculated as the observed mean change minus the expected RTM effect. Data for the background population were obtained from the cohorts where these samples were taken from, as previously described (3, 4) (Supplementary table 5).

SUPPLEMENTARY FIGURES

Supplementary figure 1. Bland-Altman plots of different day reproducibility of lab-based NITs in a pooled analysis of Reproducibility cohorts I-III (n=536). The solid horizontal line indicates the mean bias, defined as the average signed difference between repeated measurements. Thus, a bias close to zero indicates no systematic tendency for the second measurement to be higher or lower than the first measurement. The dashed horizontal lines indicate the 95% limits of agreement (LoA), representing the range within which approximately 95% of differences between repeated measurements are expected to lie. Grey points indicate the 10% of pairs with the largest absolute relative differences and are shown to visually identify extreme differences. Proportional bias was assessed by linear regression of the signed paired difference on the paired mean. A significant slope indicates that the direction or magnitude of the signed difference varies systematically across the biomarker's range, with negative beta indicating systematically lower values at second measurement, and positive beta indicating higher values. Heteroscedasticity was assessed by linear regression of the absolute paired difference on the paired mean. A significant slope indicates that the magnitude of random measurement variability changes across the biomarker's range, irrespective of whether the second measurement is higher or lower than the first.

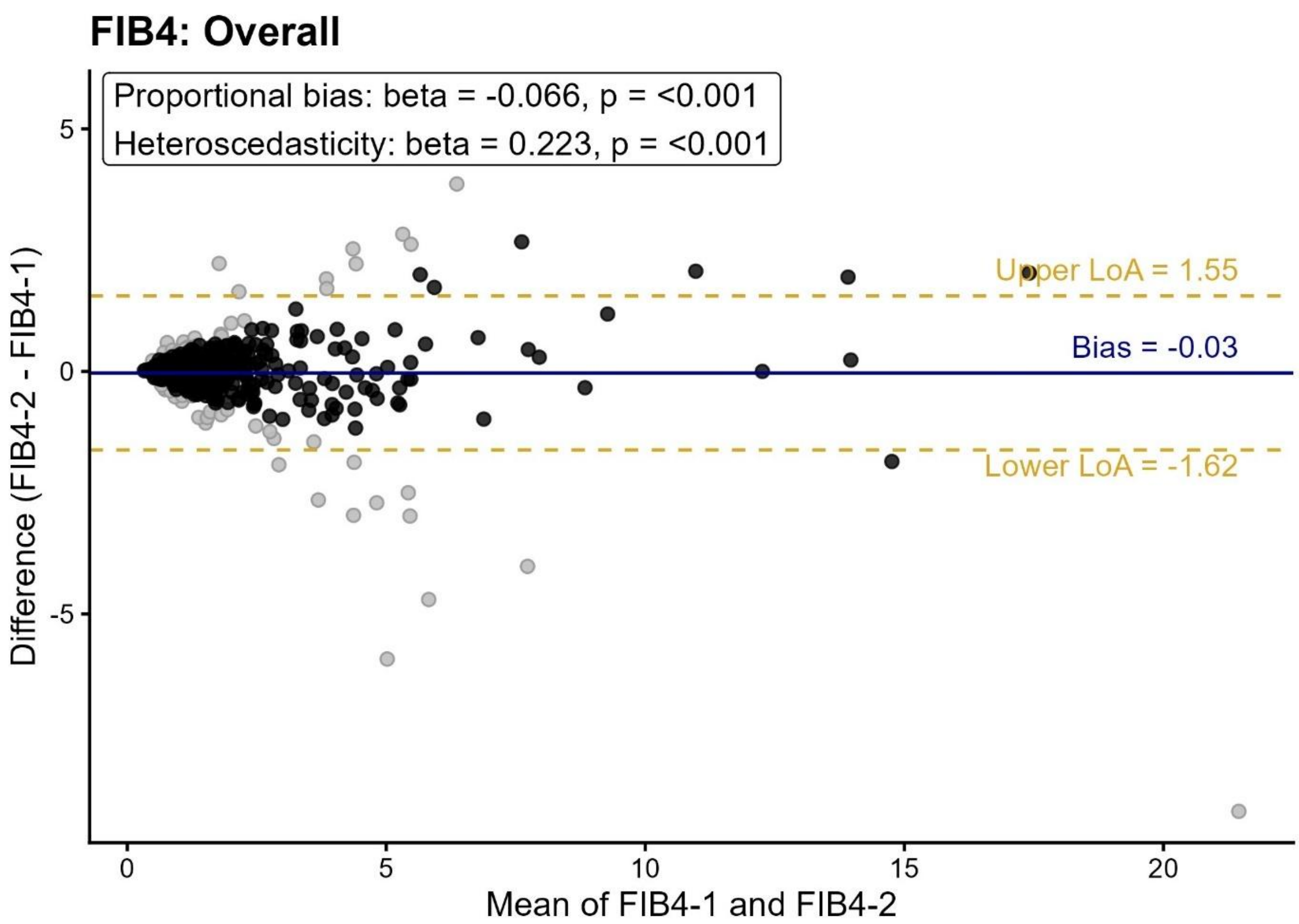
FIB4: Overall
Proportional bias: beta = -0.066, p = <0.001
Heteroscedasticity: beta = 0.223, p = <0.001
Upper LoA = 1.55
Bias = -0.03
Lower LoA = -1.62
Difference (FIB4-2 - FIB4-1)
Mean of FIB4-1 and FIB4-2
5
0
-5
0
5
10
15
20

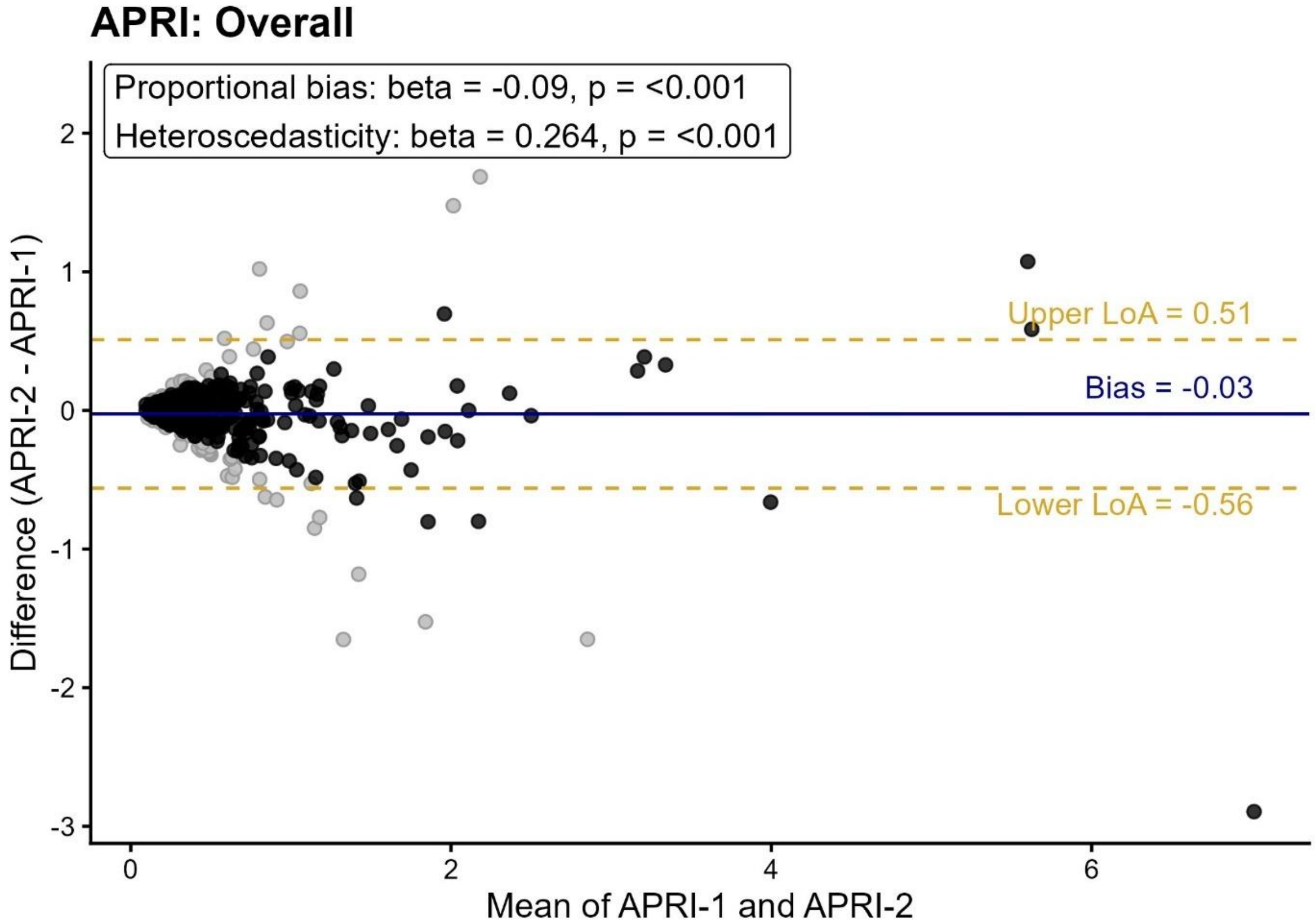
APRI: Overall
Proportional bias: beta = -0.09, p = <0.001
Heteroscedasticity: beta = 0.264, p = <0.001
Upper LoA = 0.51
Bias = -0.03
Lower LoA = -0.56
Difference (APRI-2 - APRI-1)
Mean of APRI-1 and APRI-2
2
1
0
-1
-2
-3
0
2
4
6

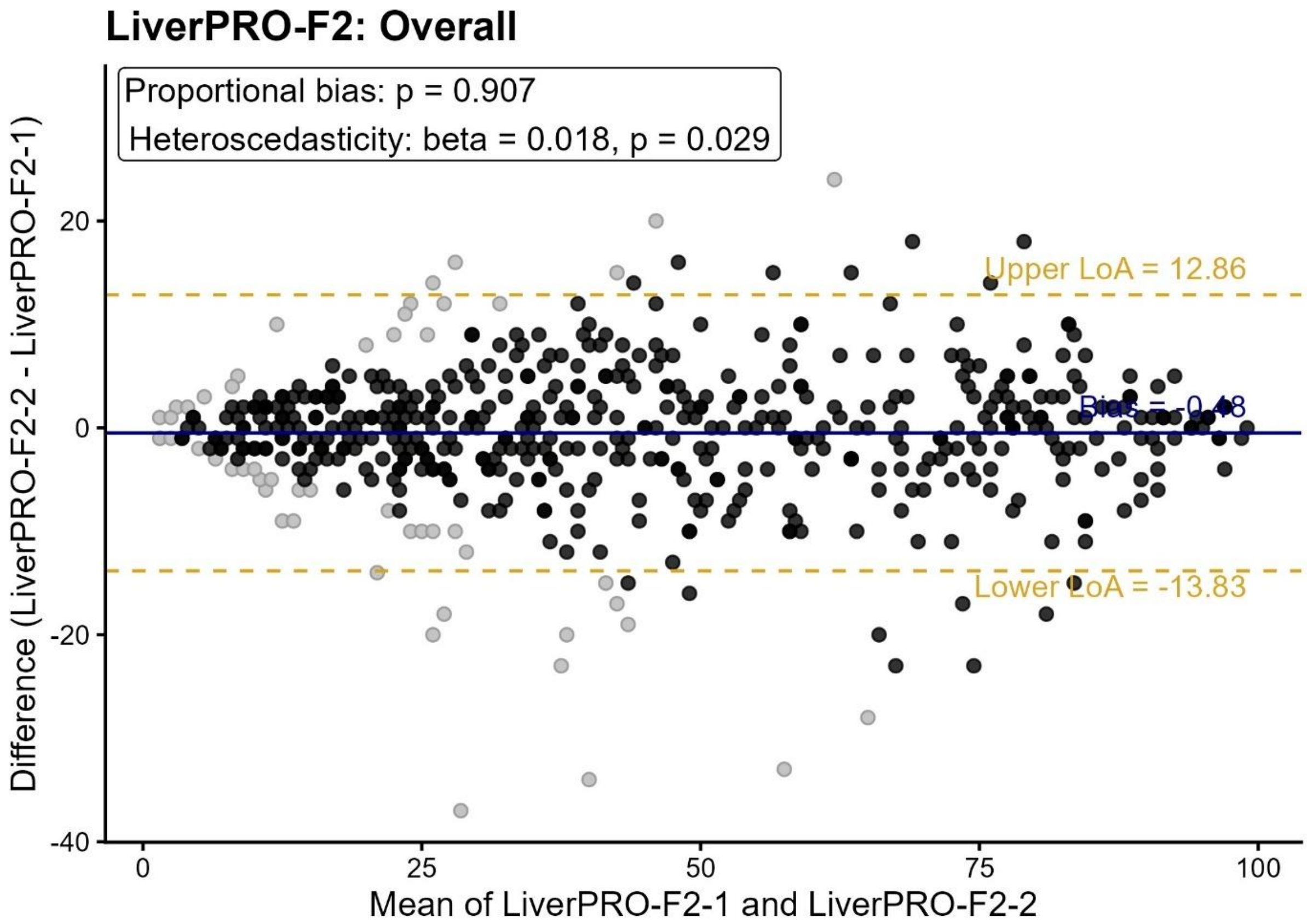
LiverPRO-F2: Overall
Proportional bias: p = 0.907
Heteroscedasticity: beta = 0.018, p = 0.029
Upper LoA = 12.86
Bias = -0.48
Lower LoA = -13.83
Difference (LiverPRO-F2-2 - LiverPRO-F2-1)
Mean of LiverPRO-F2-1 and LiverPRO-F2-2
20
0
-20
-40
0
25
50
75
100

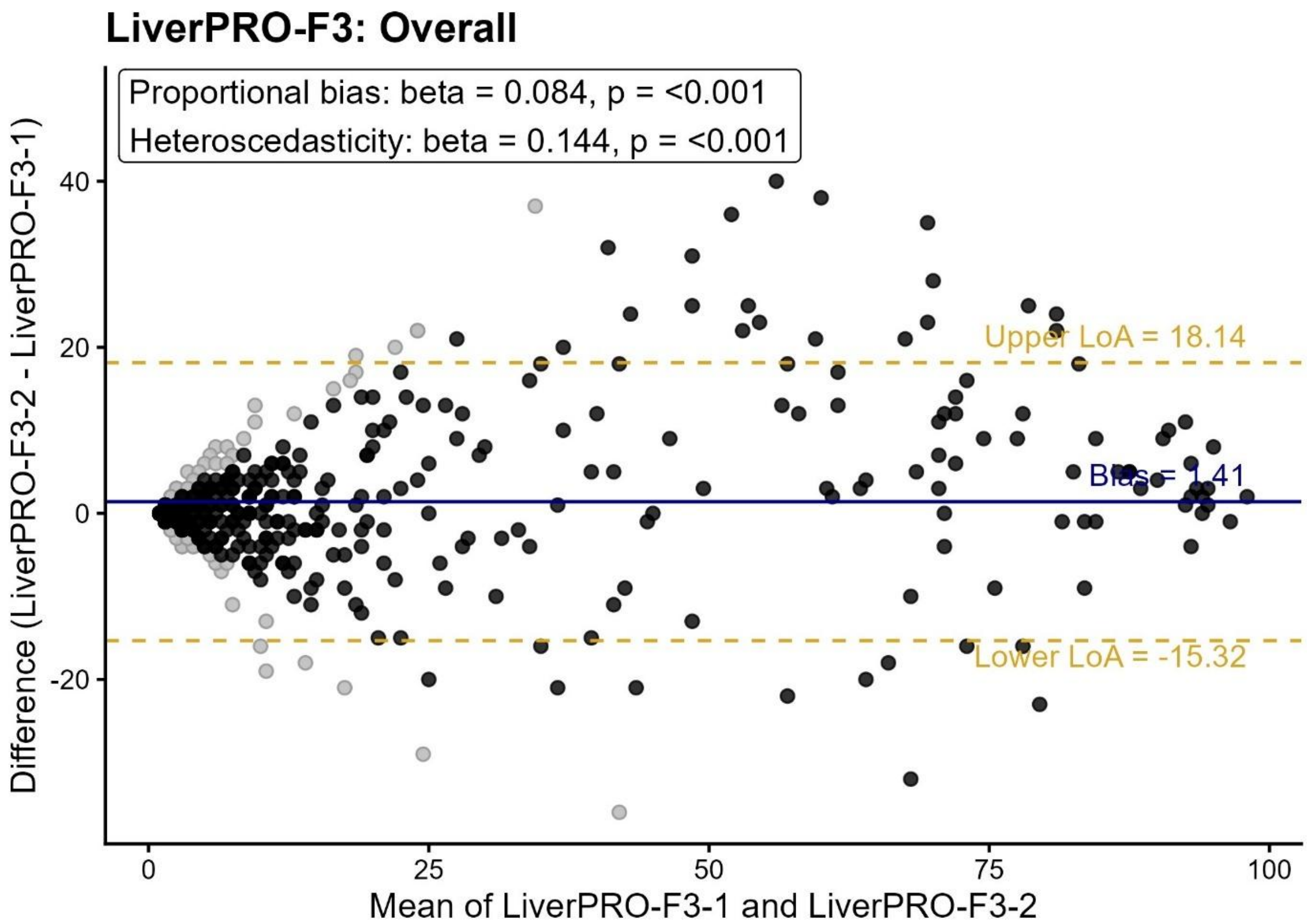
LiverPRO-F3: Overall
Proportional bias: beta = 0.084, p = <0.001
Heteroscedasticity: beta = 0.144, p = <0.001
Upper LoA = 18.14
Bias = 1.41
Lower LoA = -15.32
Difference (LiverPRO-F3-2 - LiverPRO-F3-1)
Mean of LiverPRO-F3-1 and LiverPRO-F3-2
40
20
0
-20
0
25
50
75
100

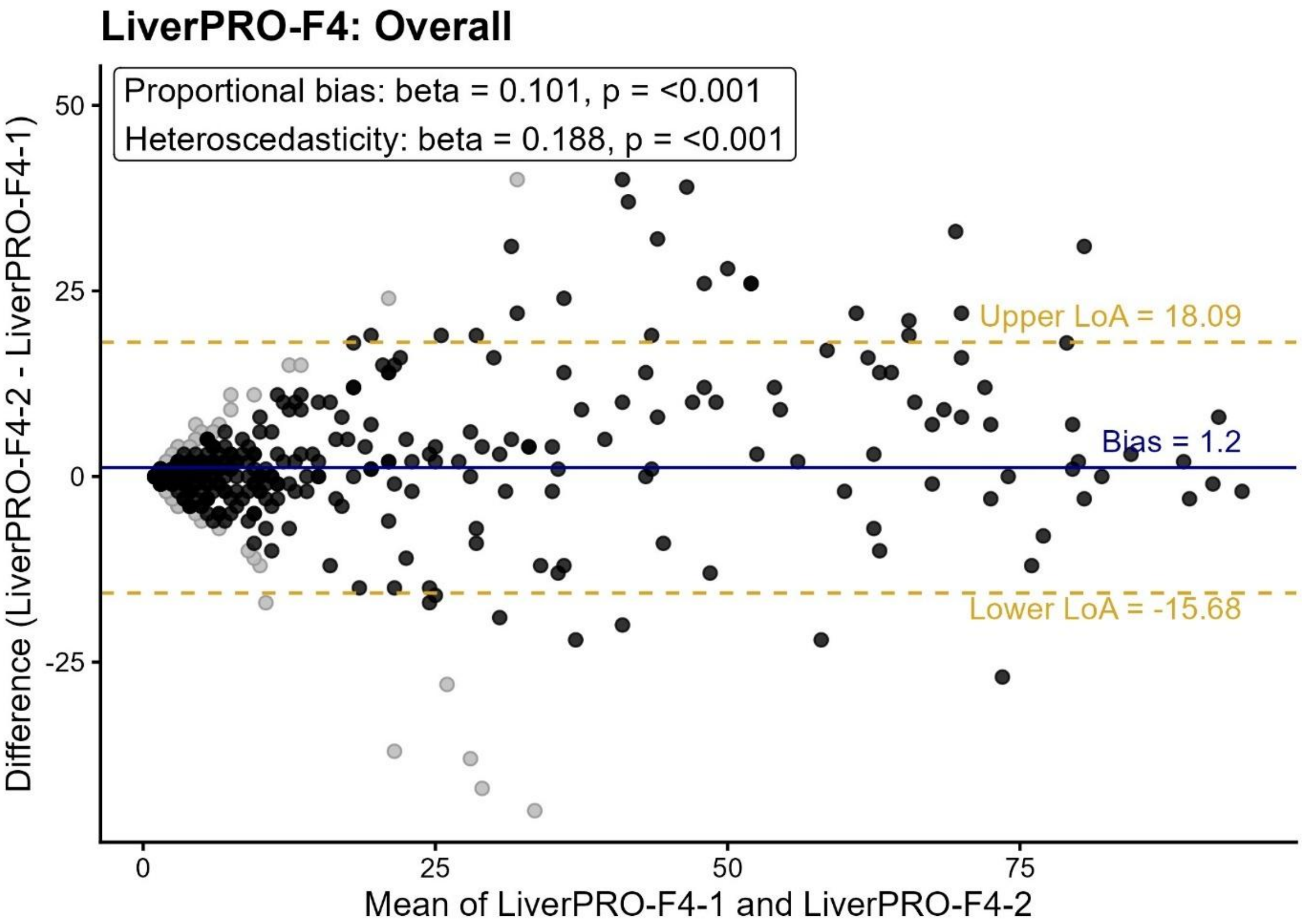
LiverPRO-F4: Overall
Proportional bias: beta = 0.101, p = <0.001
Heteroscedasticity: beta = 0.188, p = <0.001
Upper LoA = 18.09
Bias = 1.2
Lower LoA = -15.68
Difference (LiverPRO-F4-2 - LiverPRO-F4-1)
Mean of LiverPRO-F4-1 and LiverPRO-F4-2

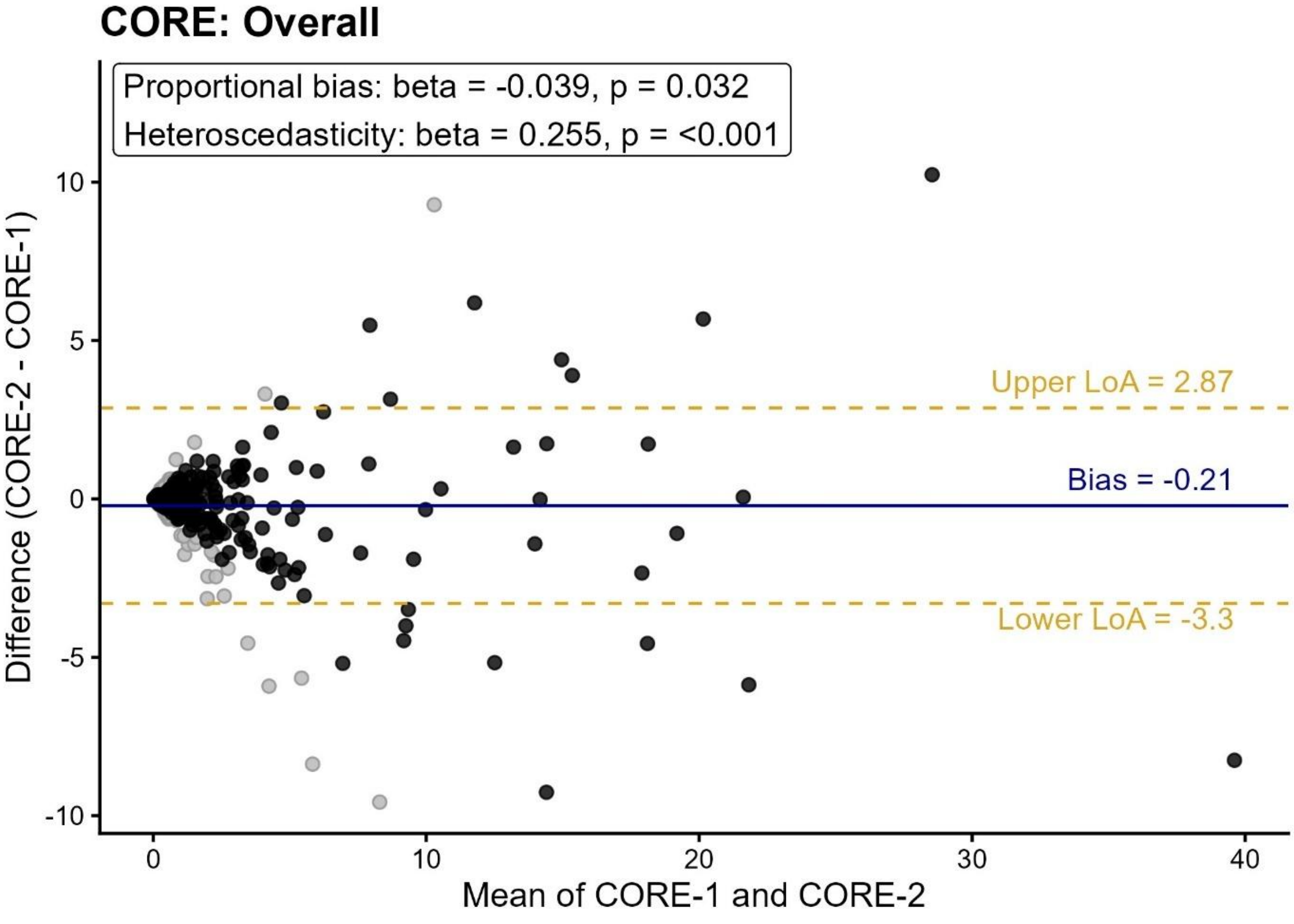
CORE: Overall
Proportional bias: beta = -0.039, p = 0.032
Heteroscedasticity: beta = 0.255, p = <0.001
Upper LoA = 2.87
Bias = -0.21
Lower LoA = -3.3
Difference (CORE-2 - CORE-1)
Mean of CORE-1 and CORE-2

Supplementary figure 2. High-resolution figures as provided in Figure 2. Local minimal detectable change (MDC) and relative MDC% were estimated across the observed range of each NIT using a fixed-grid nearest-neighbor approach. For each biomarker, the left column shows the local absolute MDC on the original scale and the right column shows the corresponding local MDC%. The x-axis represents the paired mean of the two measurements. Grey points indicate the unsmoothed local MDC estimates at each grid point, the blue line indicates the smoothed local estimate, and the shaded ribbon indicates the smoothed bootstrap 95% confidence interval. FIB-4 and APRI are shown in points, whereas LiverPRO and CORE are shown on the original percentage scale.

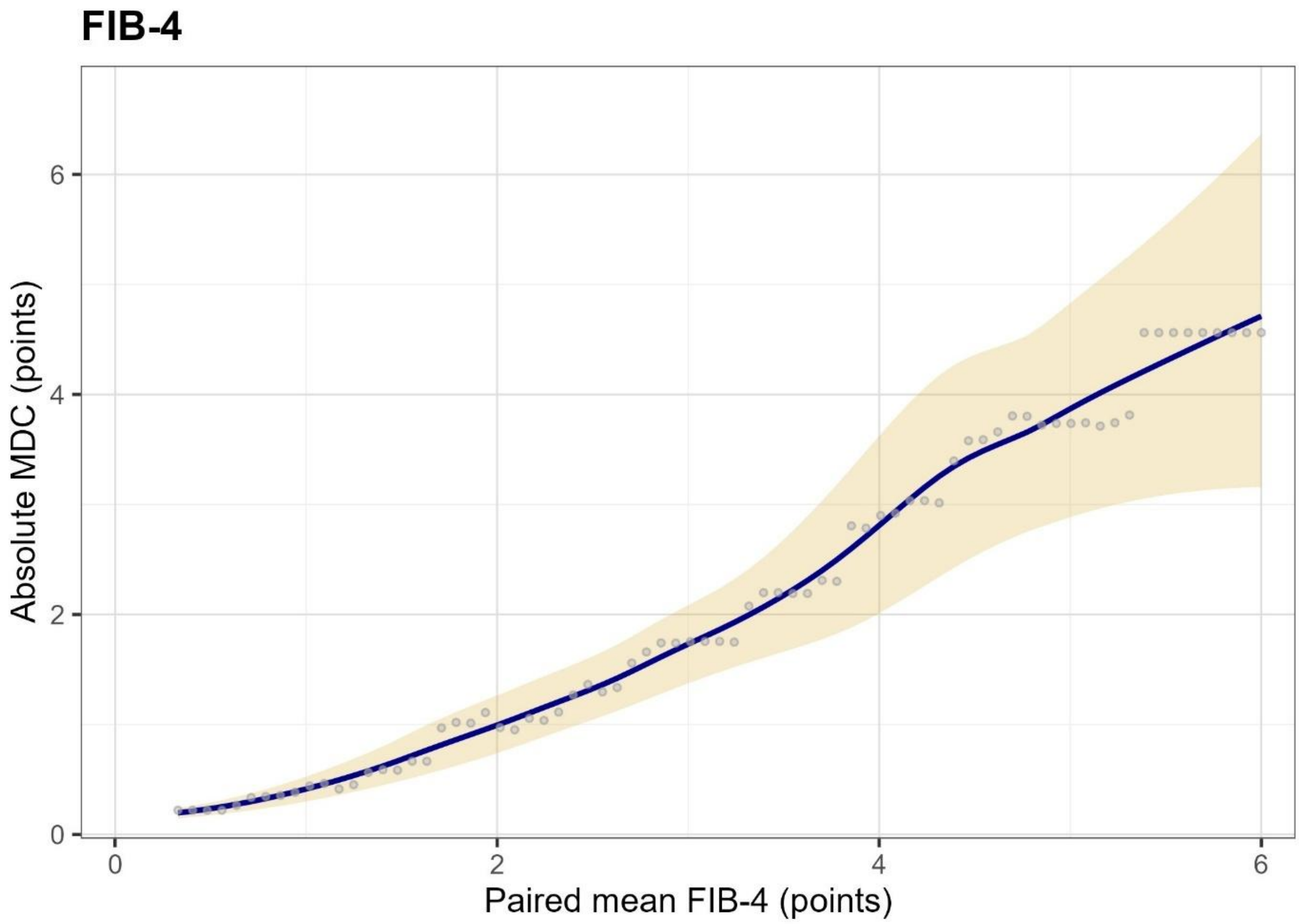
FIB-4
Absolute MDC (points)
Paired mean FIB-4 (points)
0
2
4
6

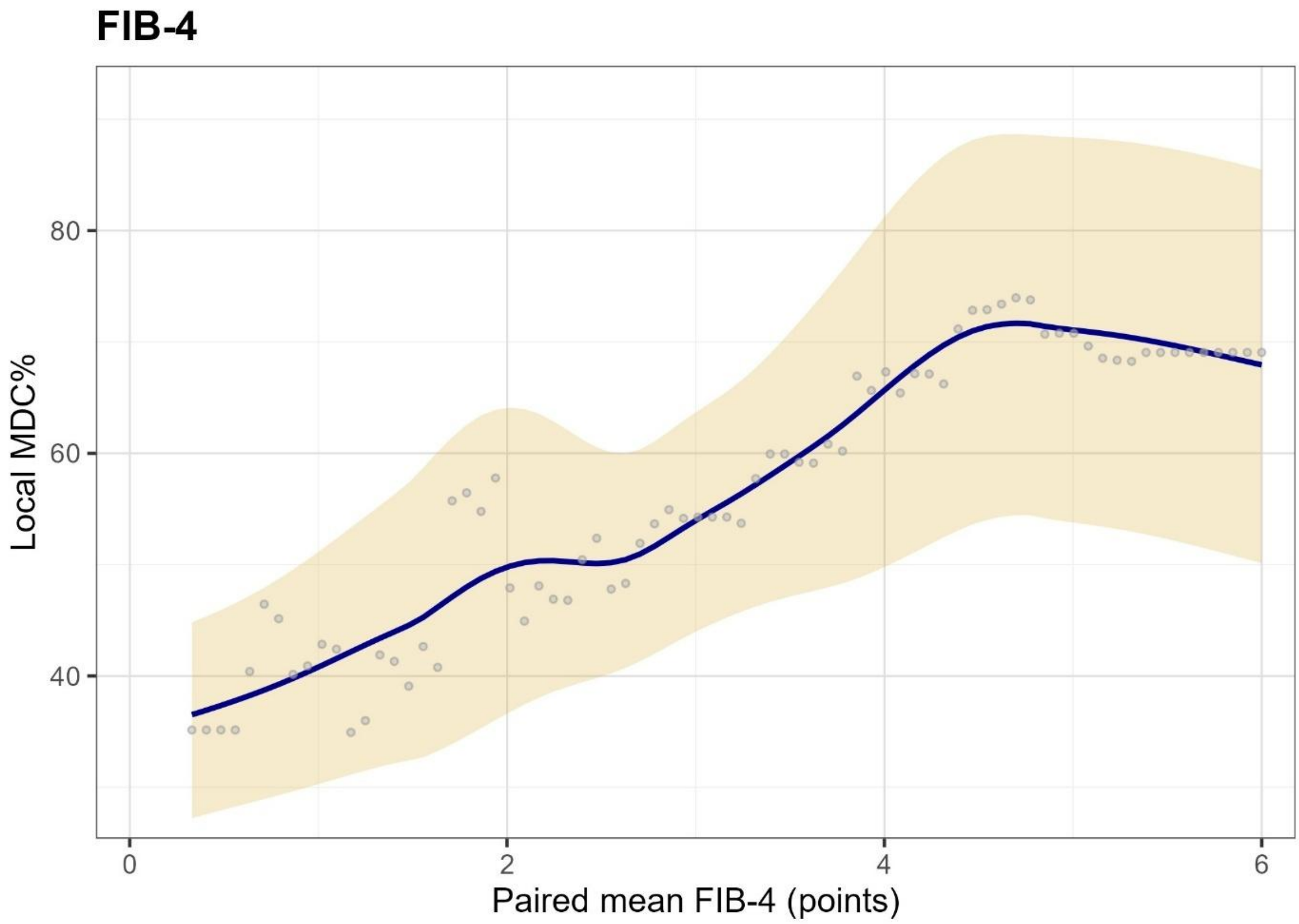
FIB-4
Local MDC%
Paired mean FIB-4 (points)
40
60
80
0
2
4
6

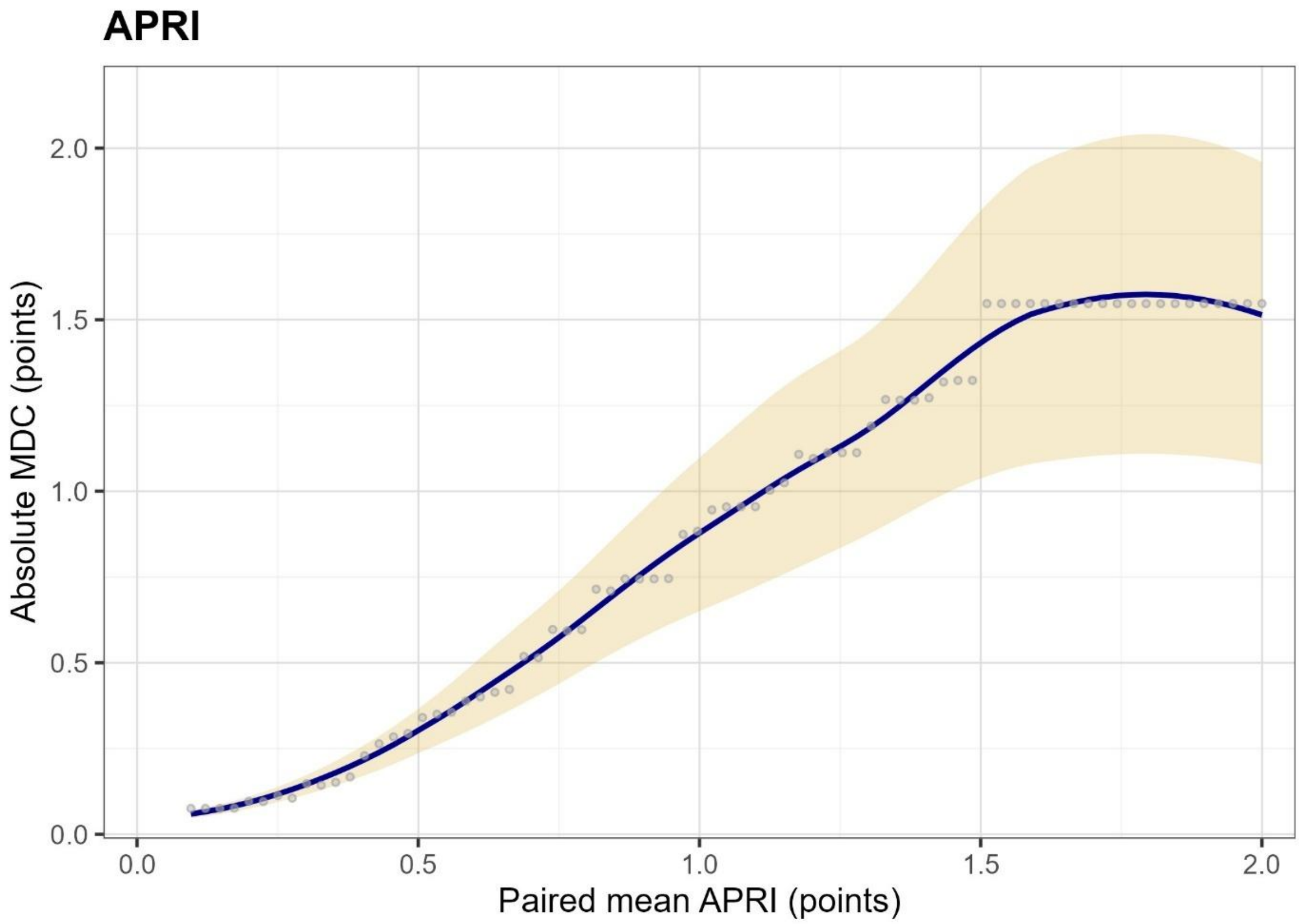
APRI
Absolute MDC (points)
2.0
1.5
1.0
0.5
0.0
0.0
0.5
1.0
1.5
2.0
Paired mean APRI (points)

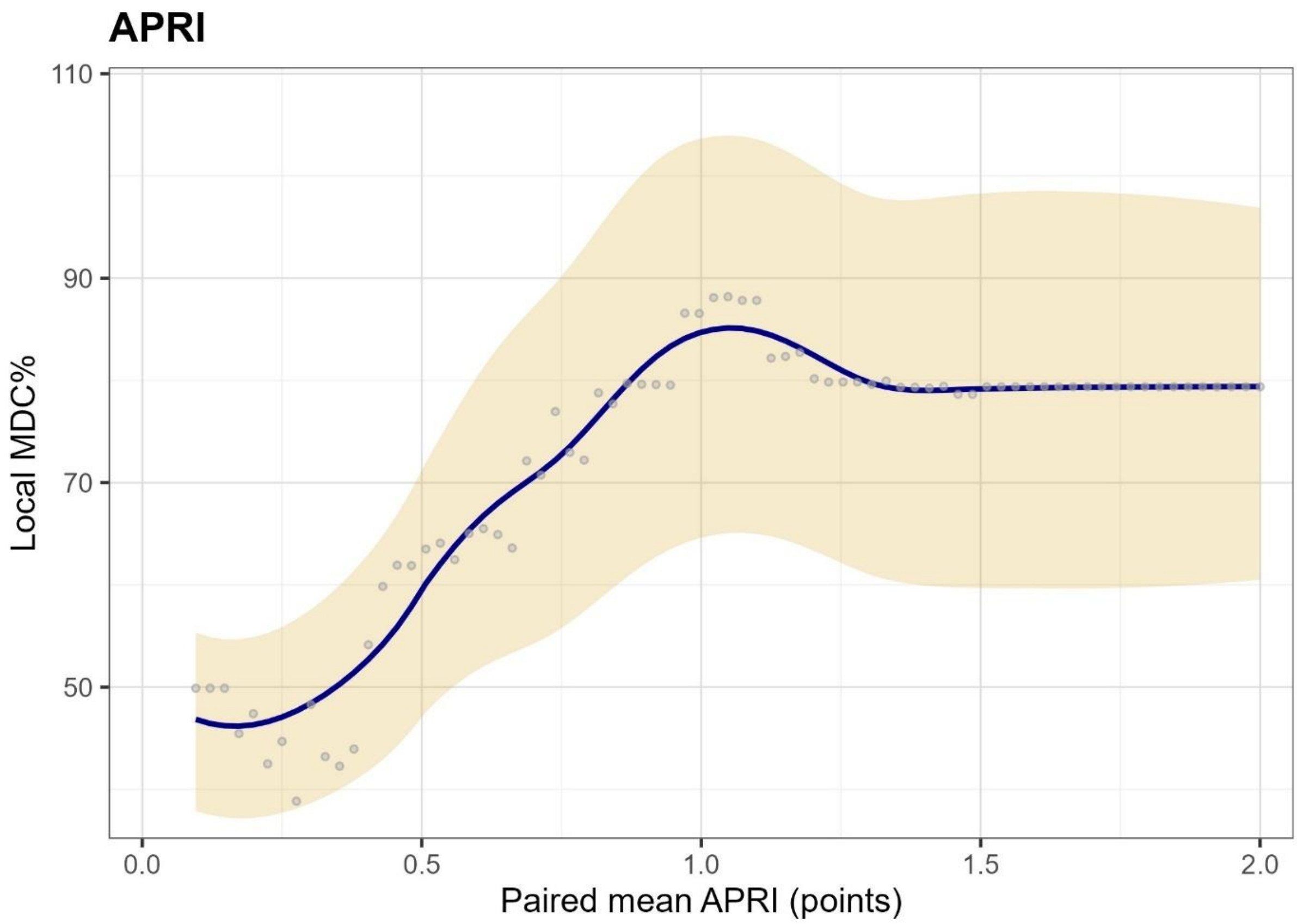
APRI
Local MDC%
110
90
70
50
0.0
0.5
1.0
1.5
2.0
Paired mean APRI (points)

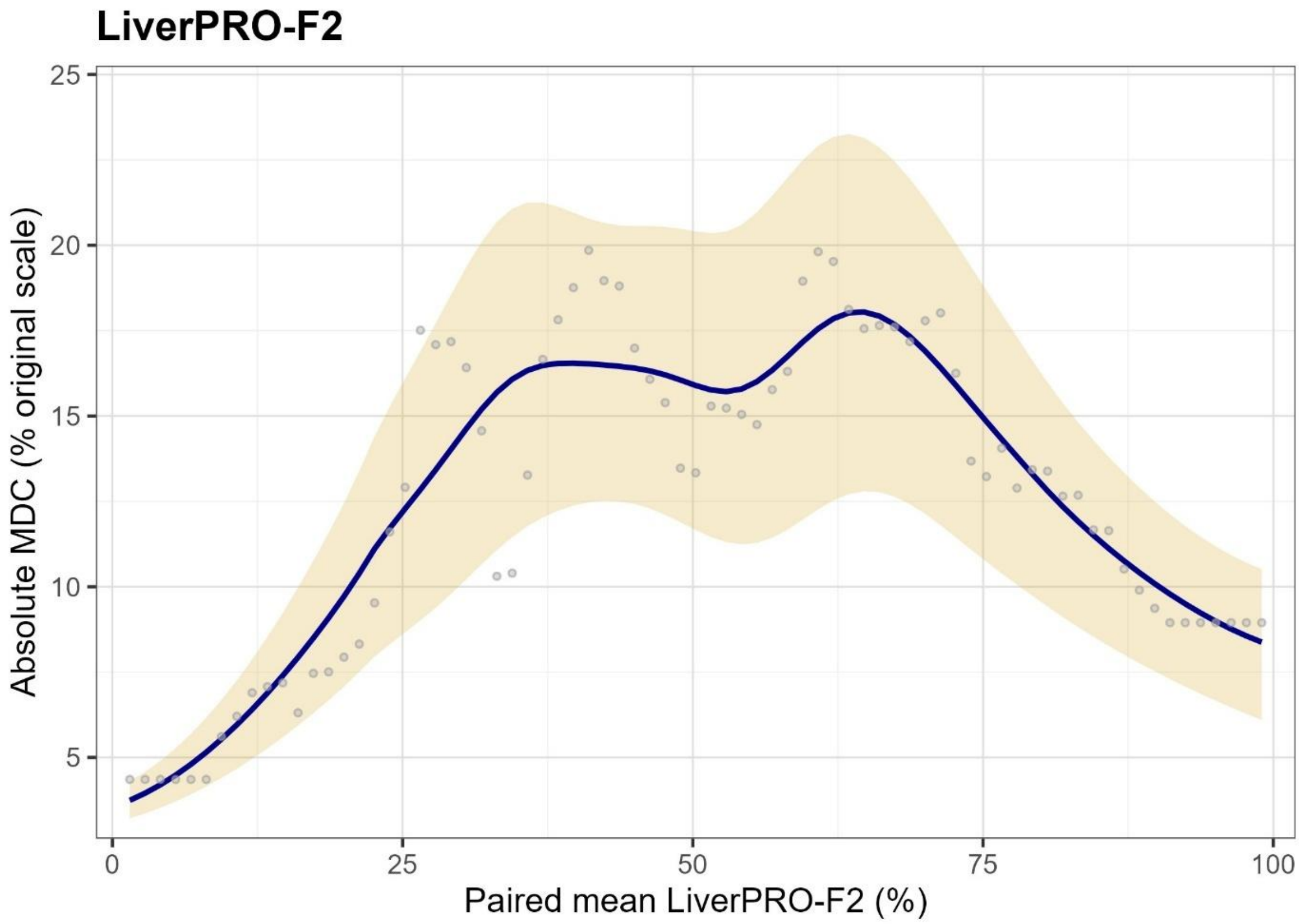
LiverPRO-F2
Absolute MDC (% original scale)
25
20
15
10
5
0
25
50
75
100
Paired mean LiverPRO-F2 (%)

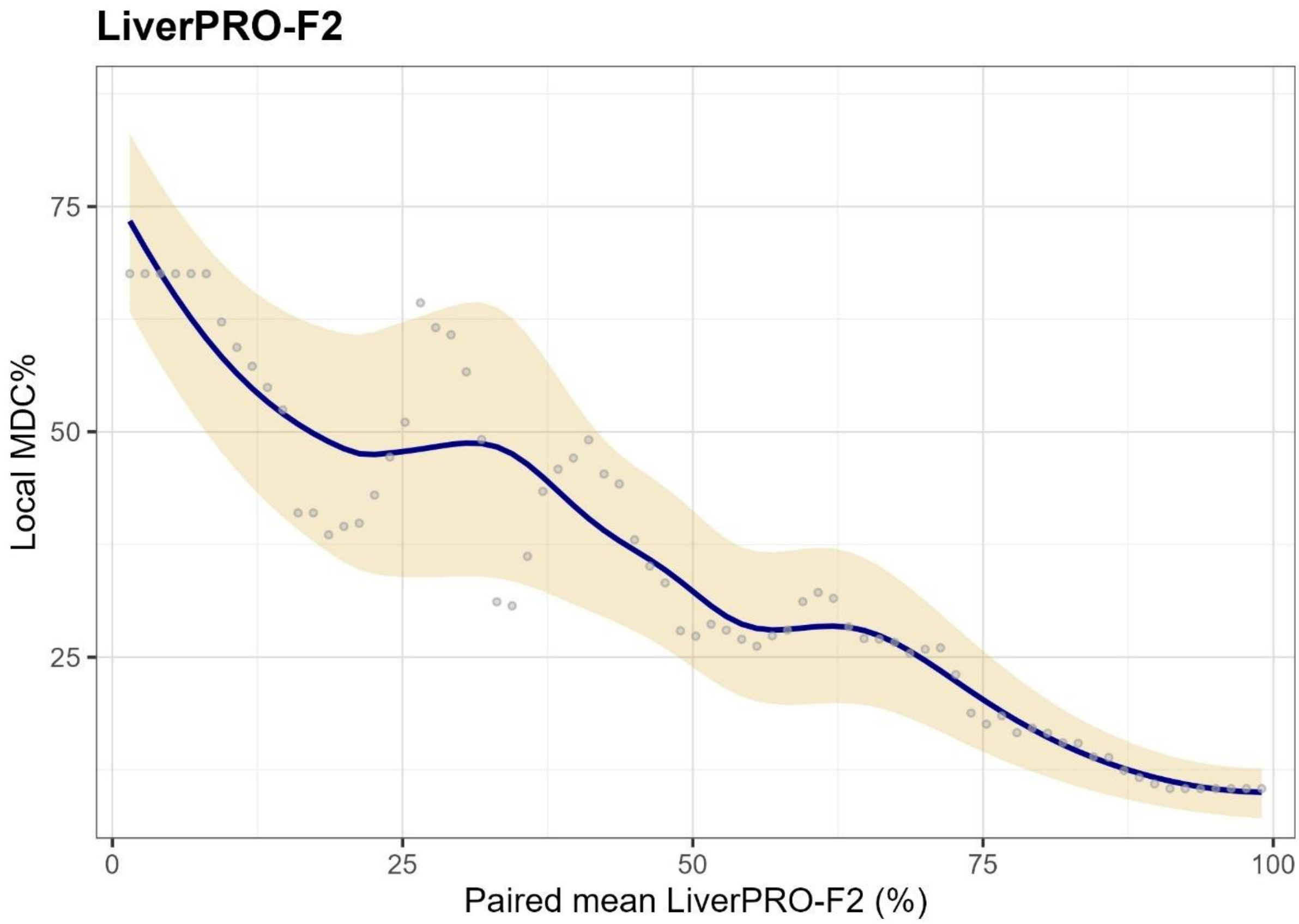
LiverPRO-F2
Local MDC%
75
50
25
0
25
50
75
100
Paired mean LiverPRO-F2 (%)

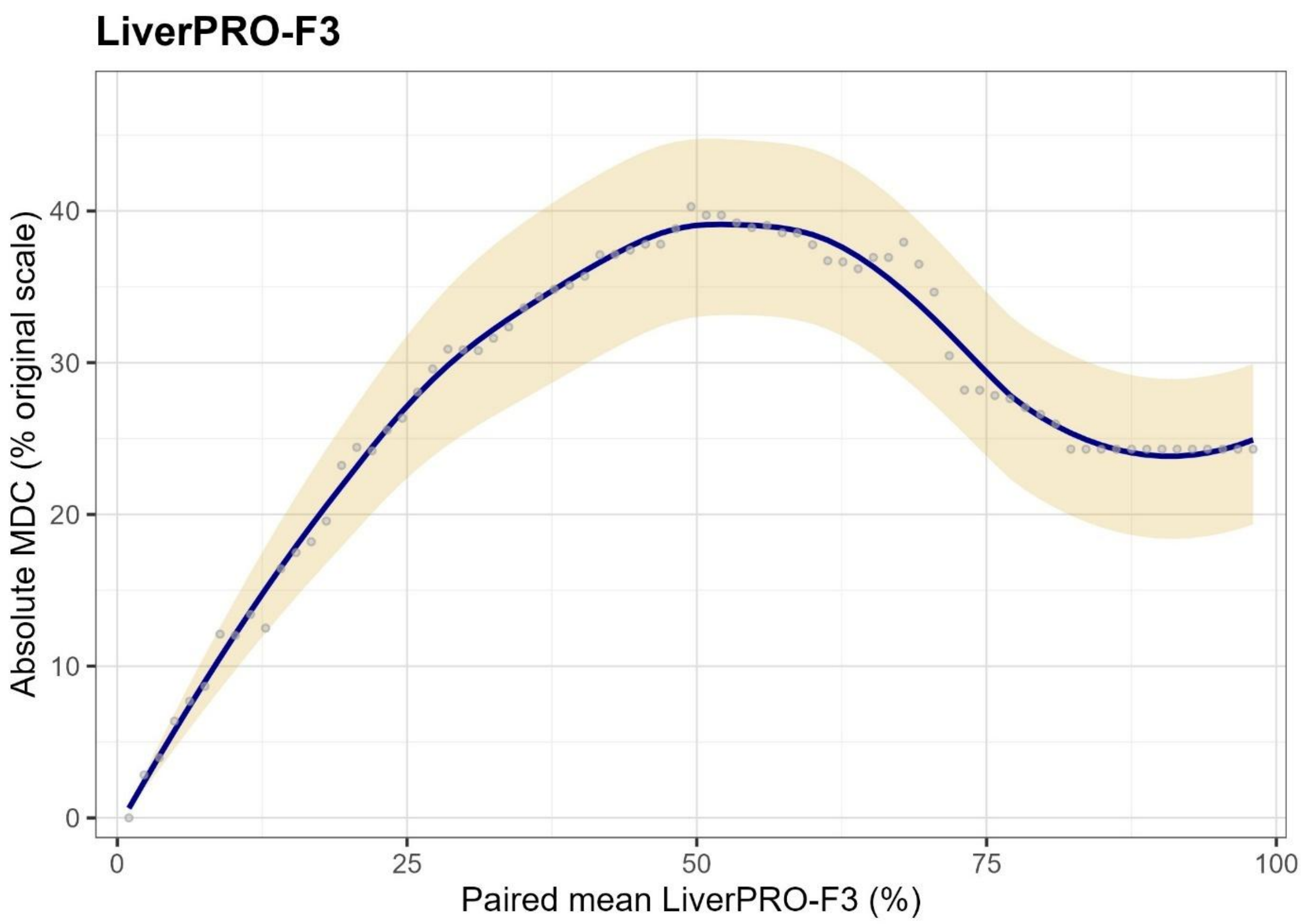
LiverPRO-F3
Absolute MDC (% original scale)
0
10
20
30
40
0
25
50
75
100
Paired mean LiverPRO-F3 (%)

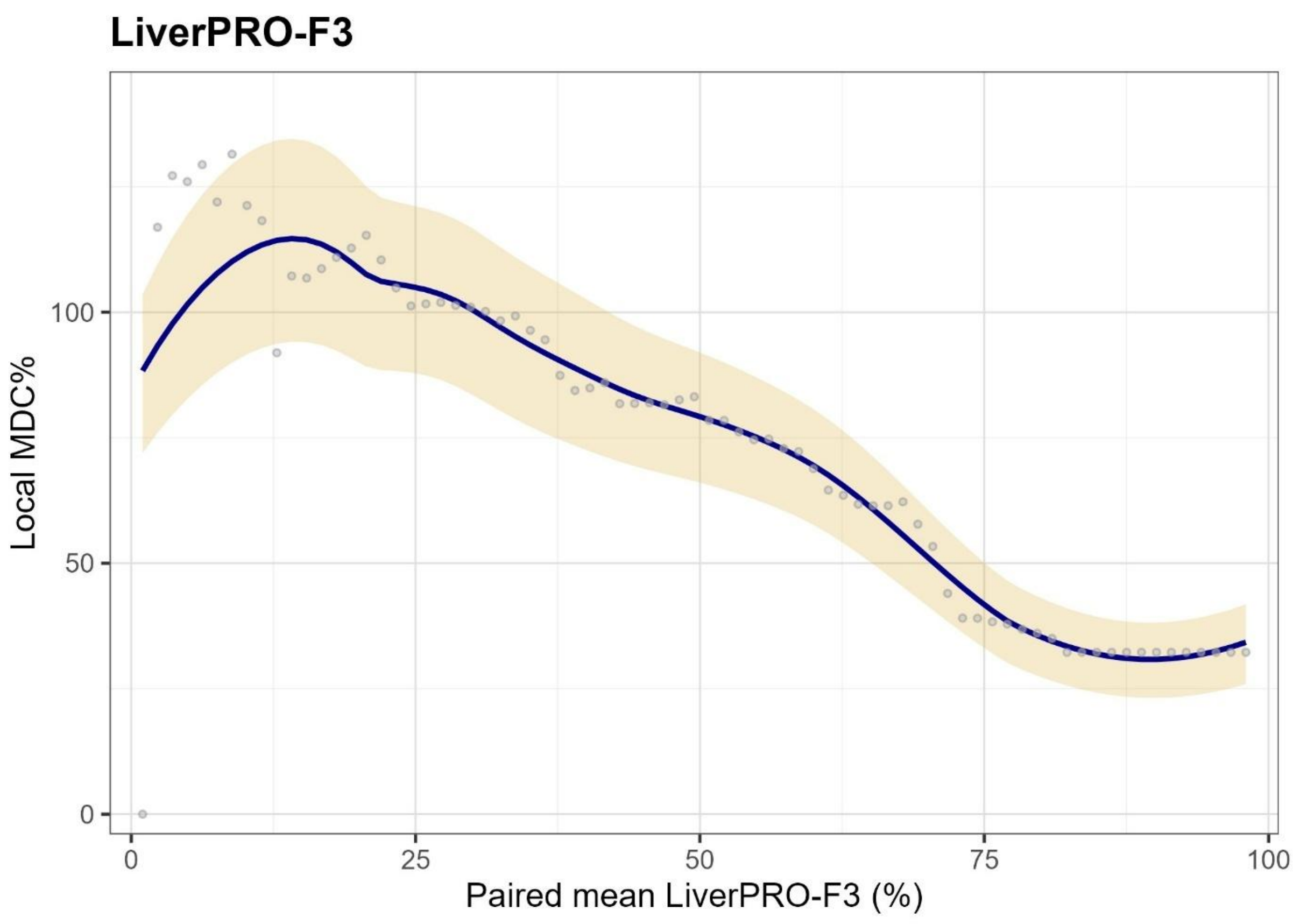
LiverPRO-F3
Local MDC%
0
50
100
0
25
50
75
100
Paired mean LiverPRO-F3 (%)

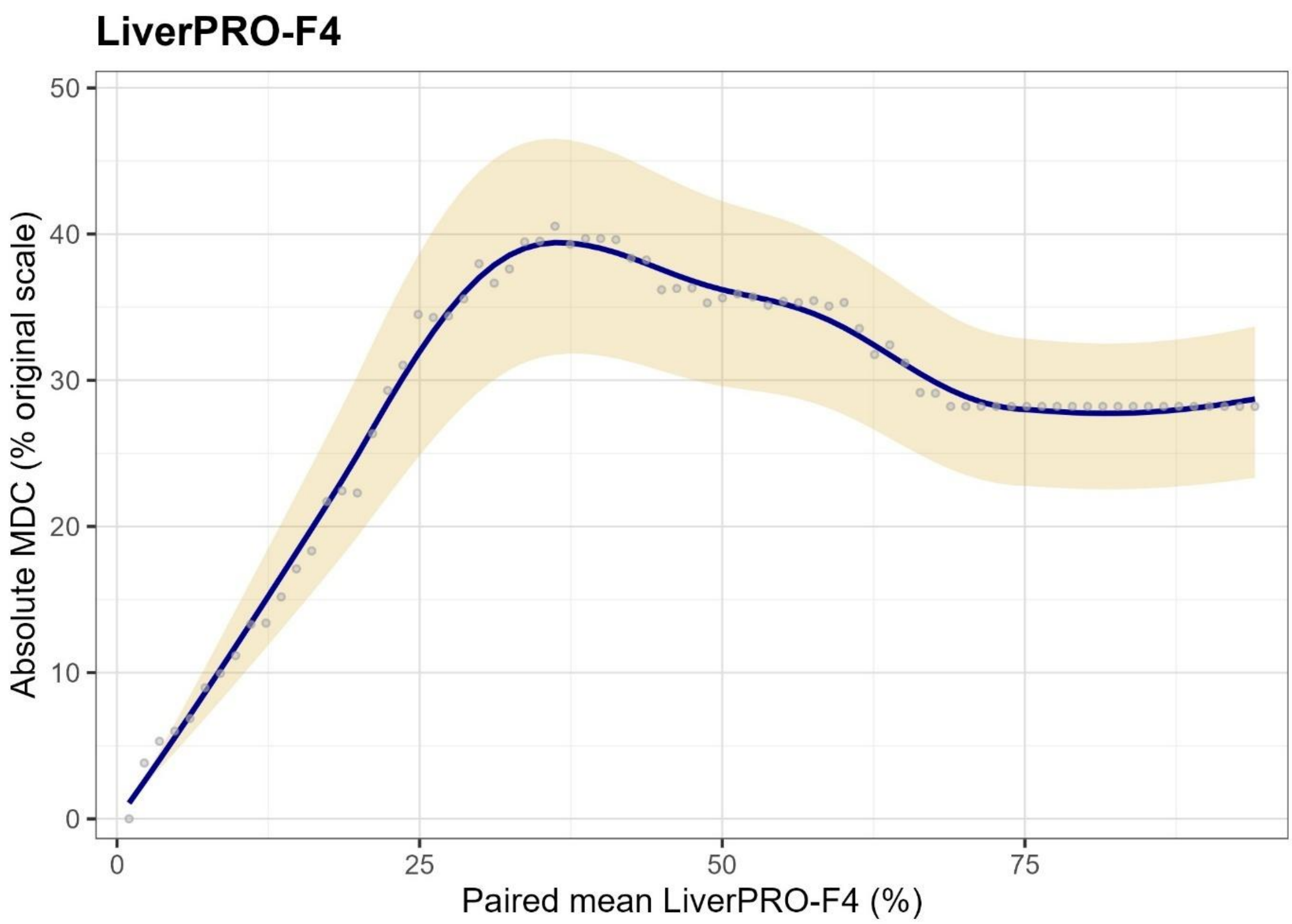
LiverPRO-F4
Absolute MDC (% original scale)
50
40
30
20
10
0
0
25
50
75
Paired mean LiverPRO-F4 (%)

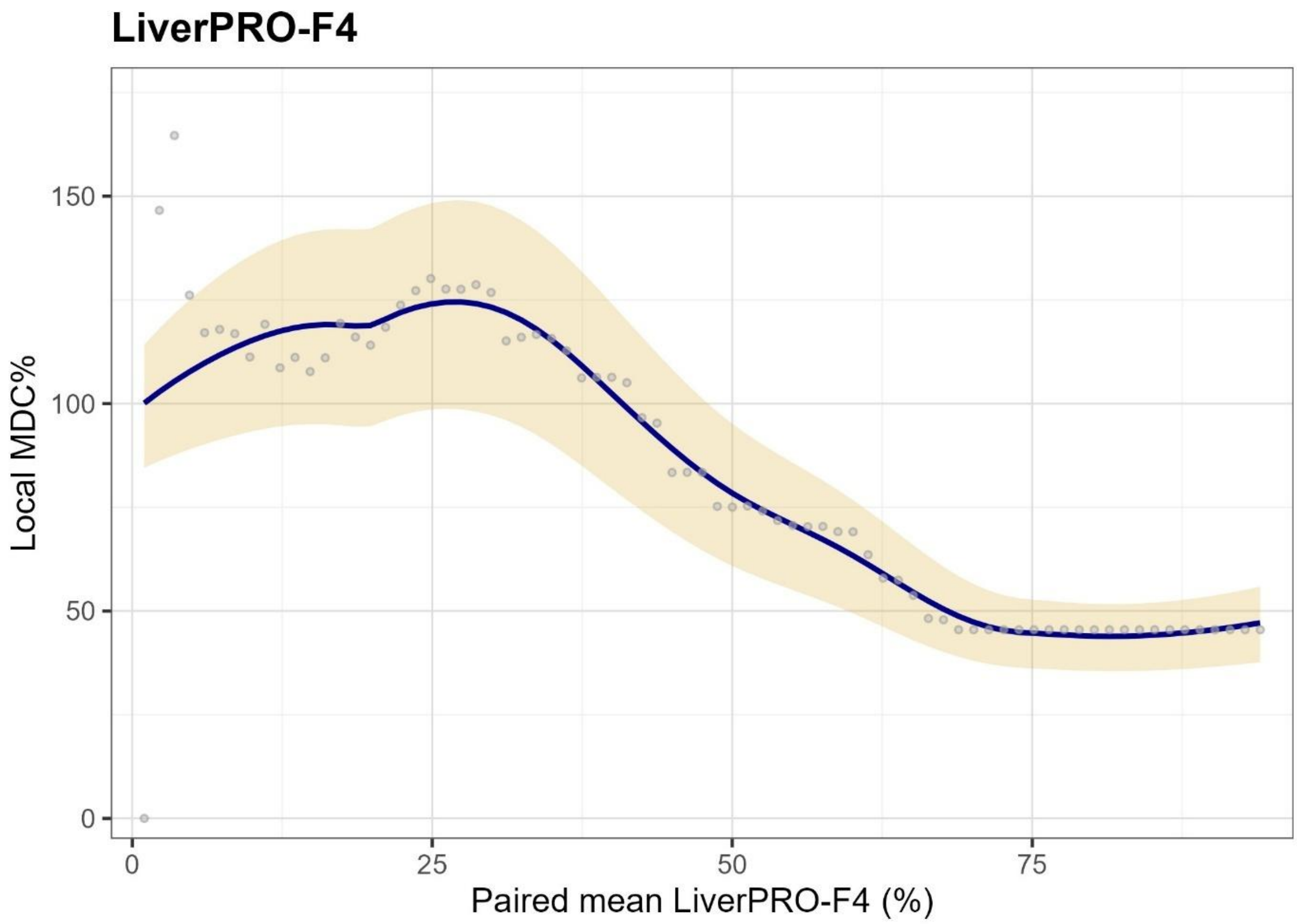
LiverPRO-F4
Local MDC%
150
100
50
0
0
25
50
75
Paired mean LiverPRO-F4 (%)

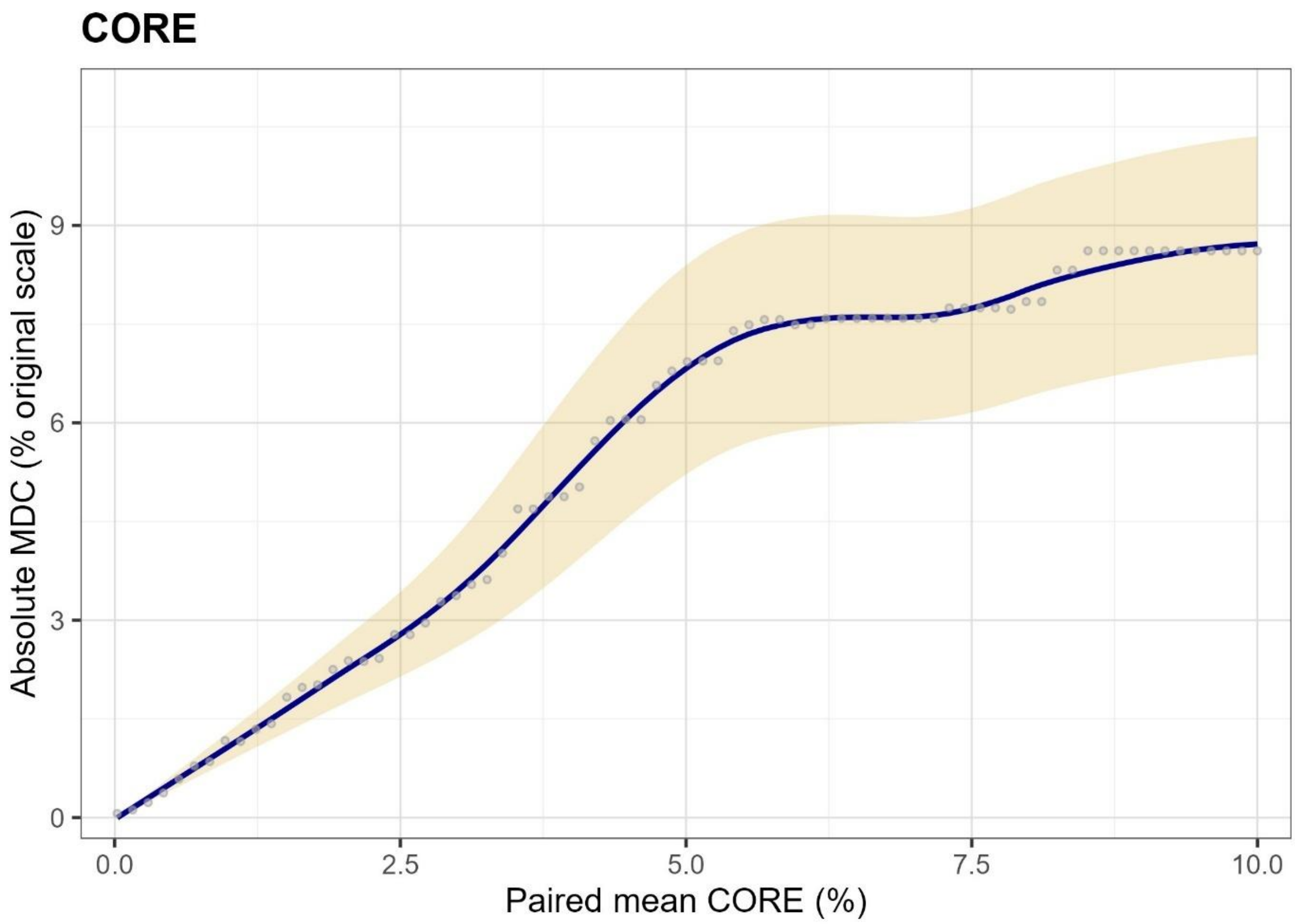
CORE
Absolute MDC (% original scale)
0
3
6
9
0.0
2.5
5.0
7.5
10.0
Paired mean CORE (%)

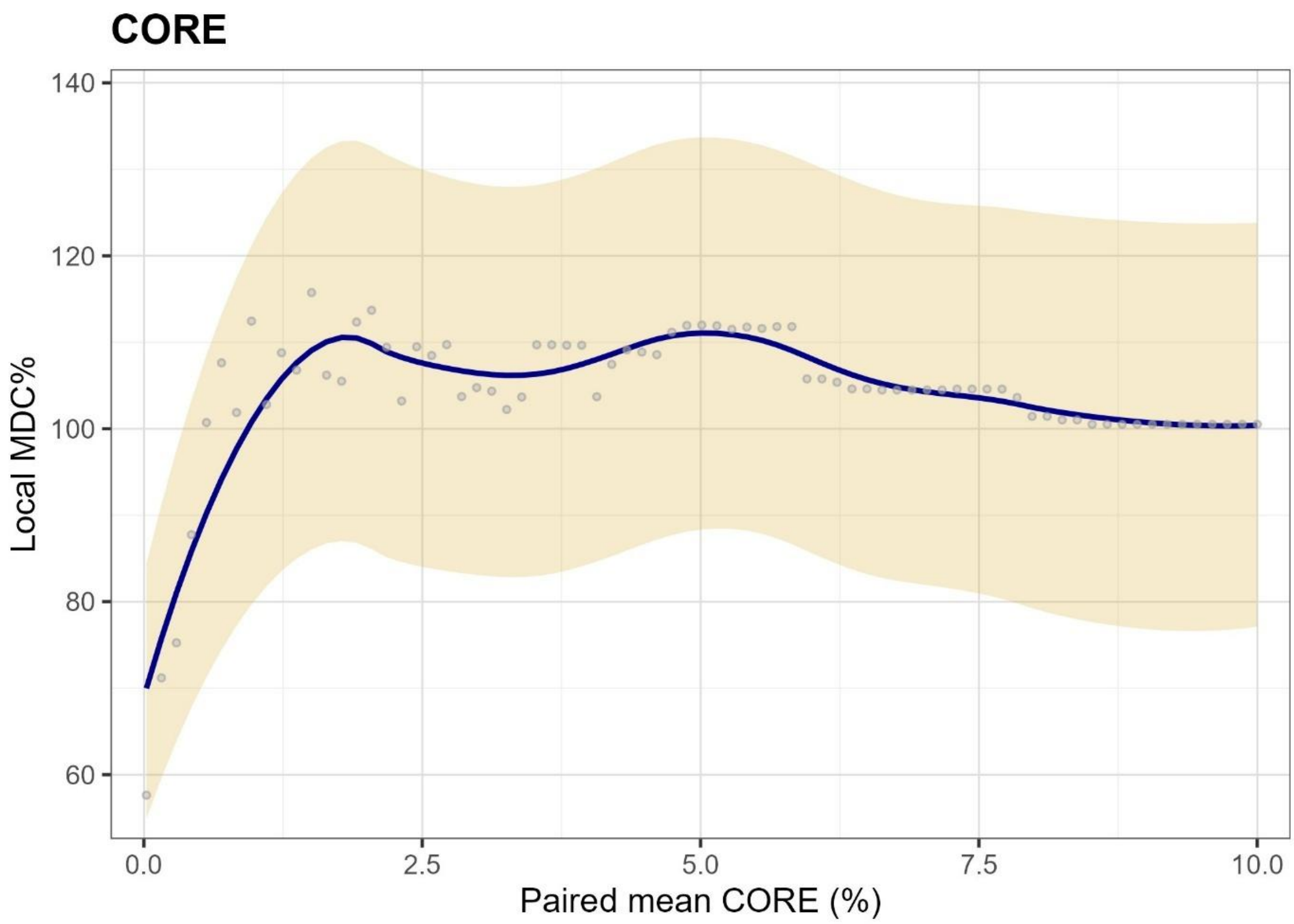
CORE
Local MDC%
60
80
100
120
140
0.0
2.5
5.0
7.5
10.0
Paired mean CORE (%)

Supplementary figure 3. (**A**) Relationship between LSM expressed as shear-wave speed in meters per second (m/s) and Young's modulus in kilopascal (kPa). LSM in kPa is derived from shear-wave speed according to the elastic modulus approximation: $kPa = 3 \times (m/s)^2$. Conversely, m/s can be obtained from kPa as $m/s = \sqrt{(kPa/3)}$, resulting in a square-root relationship. This demonstrates that the two reporting scales are mathematically linked but not linearly interchangeable. Equal absolute changes in kPa correspond to progressively smaller changes in m/s at higher stiffness values, whereas equal changes in m/s translate into progressively larger changes in kPa. (**B**) Back-transformed asymmetric MDC window for LSM after calculation on the natural log-transformed kPa scale. The x-axis represents LSM in kPa, and the y-axis shows the signed absolute change required to exceed the MDC, with positive values indicating an increase and negative values indicating a decrease. Because the MDC was calculated on the log scale and subsequently back-transformed to the original kPa scale, the resulting no-change window is asymmetric on the original scale. The golden area indicates the range of change in which measurement variability cannot be excluded, dark blue lines indicate the upper and lower MDC thresholds, and blue shaded areas indicate the corresponding 95% confidence intervals. Relative changes are constant across the kPa range after log transformation and correspond to −39% to +63%.

A

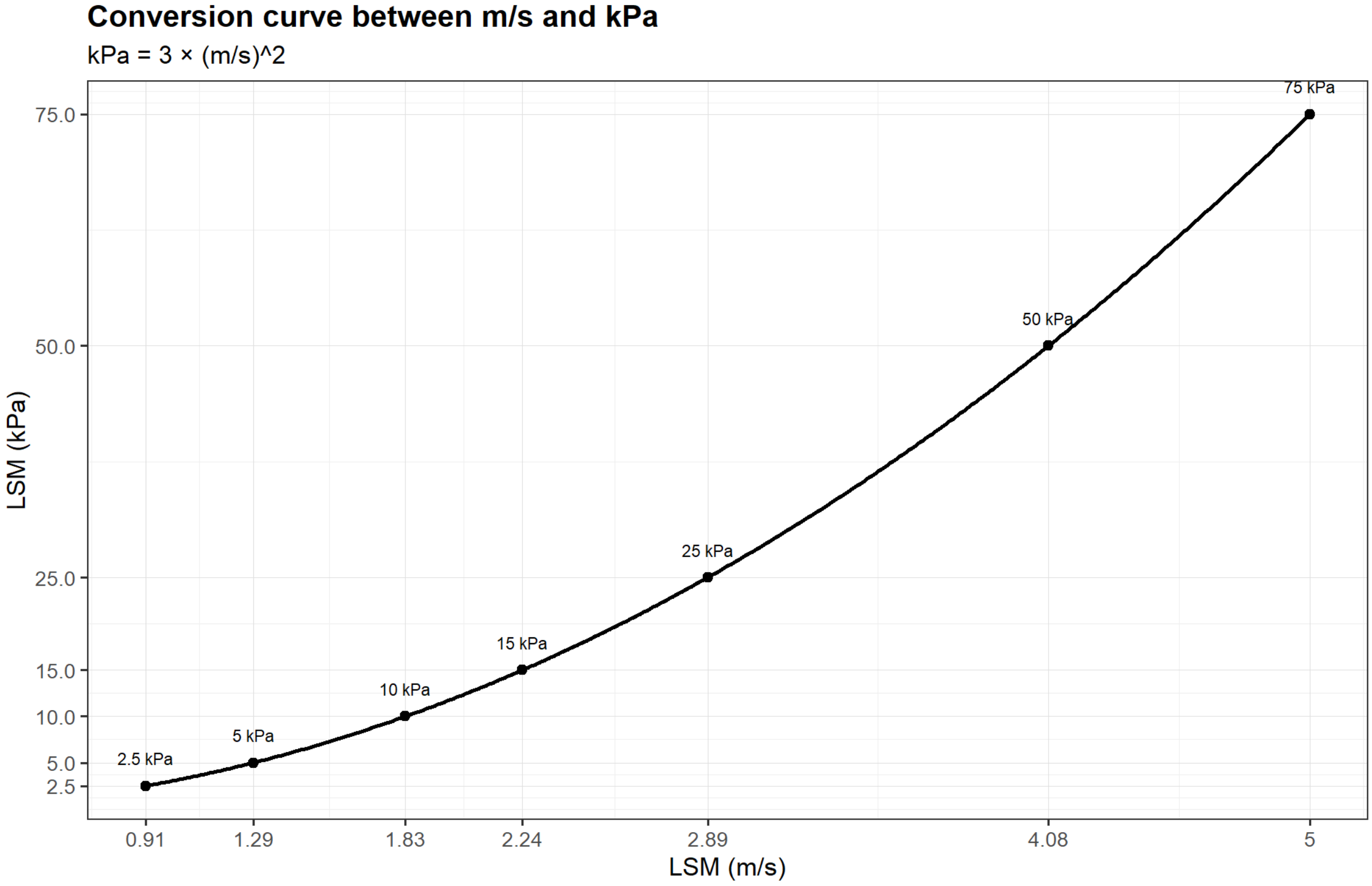

Conversion curve between m/s and kPa
kPa = 3 × (m/s)^2
75 kPa
50 kPa
25 kPa
15 kPa
10 kPa
5 kPa
2.5 kPa
75.0
50.0
25.0
15.0
10.0
5.0
2.5
LSM (kPa)
0.91
1.29
1.83
2.24
2.89
4.08
5
LSM (m/s)

B

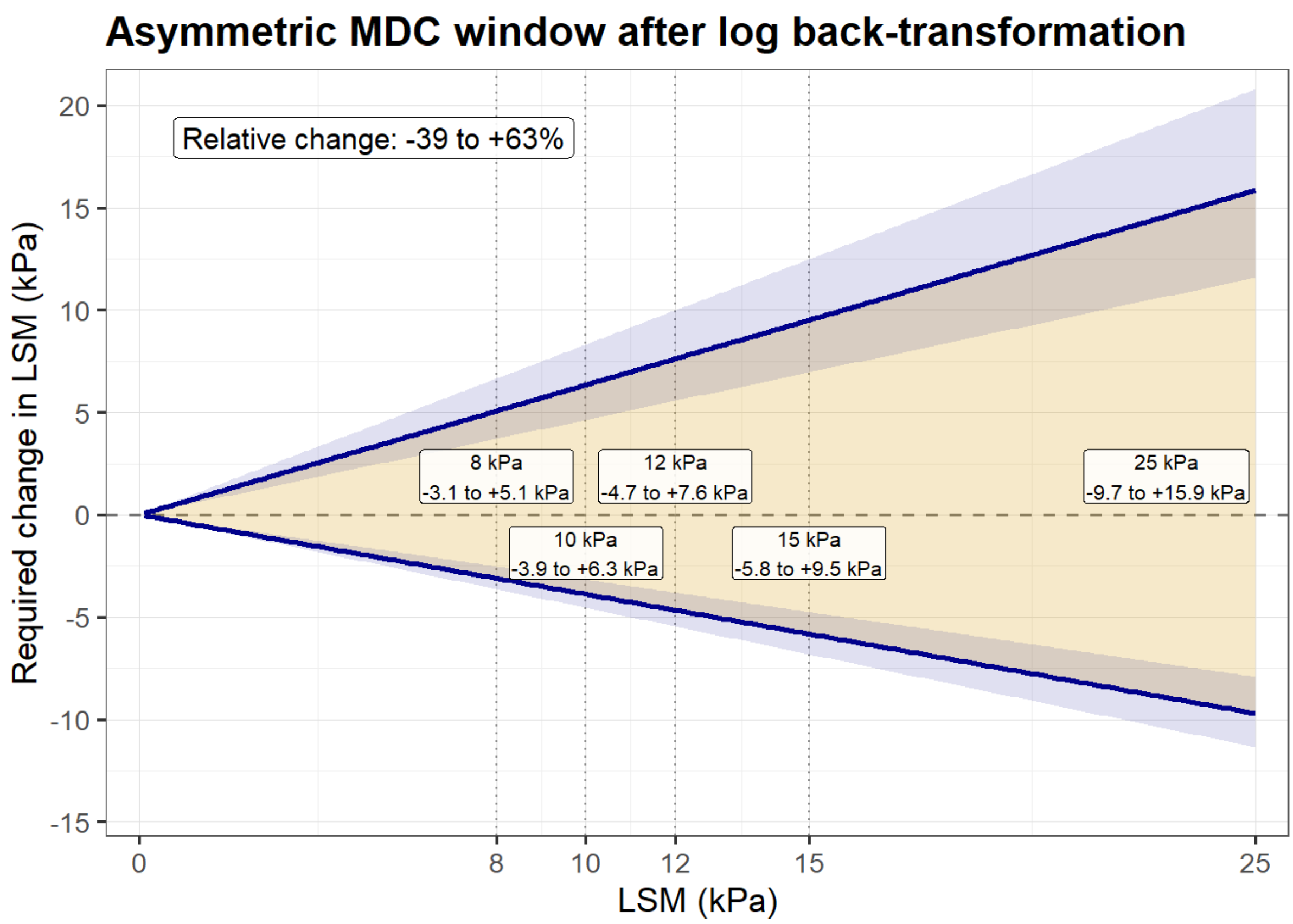

Asymmetric MDC window after log back-transformation
Relative change: -39 to +63%
8 kPa
-3.1 to +5.1 kPa
12 kPa
-4.7 to +7.6 kPa
25 kPa
-9.7 to +15.9 kPa
10 kPa
-3.9 to +6.3 kPa
15 kPa
-5.8 to +9.5 kPa
20
15
10
5
0
-5
-10
-15
Required change in LSM (kPa)
0
8
10
12
15
25
LSM (kPa)

Supplementary figure 4. High-resolution figures as provided in Figure 3. MDC estimates for LiverPRO-F2, LiverPRO-F3, LiverPRO-F4, and CORE were calculated on the logit scale and subsequently back-transformed to the original probability scale. The x-axis represents the baseline probability score in percent. The y-axis represents the signed change corresponding to the MDC, with positive values indicating the required increase and negative values indicating the required decrease. For each NIT, the upper panel shows the MDC window on the absolute probability scale, whereas the lower panel shows the corresponding relative change from baseline. The area between the upper and lower MDC thresholds represents the window of change in which measurement variability cannot be excluded. Dark blue lines indicate the MDC-based point estimate, and shaded areas indicate the corresponding 95% confidence interval. For CORE, the x-axis was restricted to 0-20%.

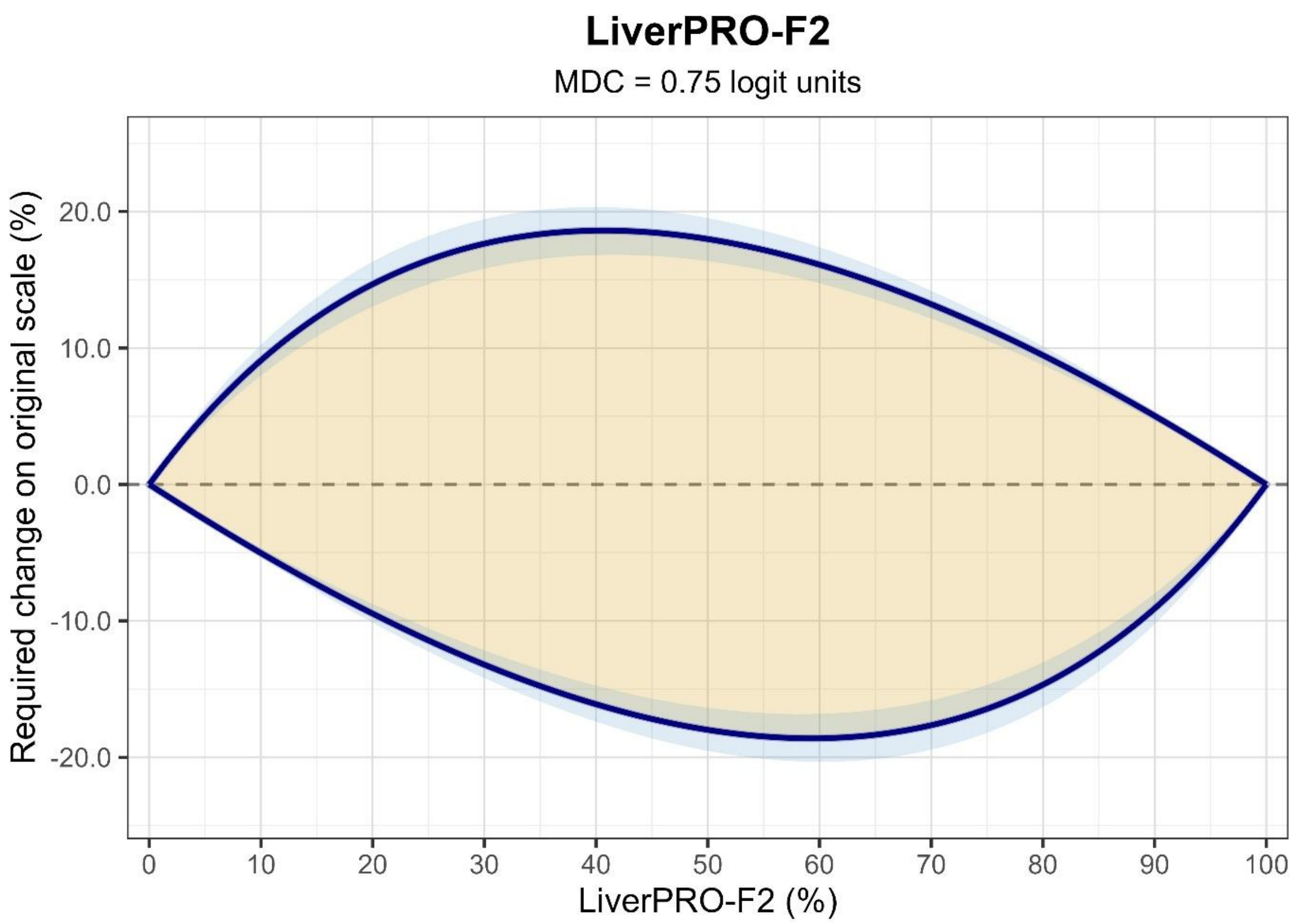
LiverPRO-F2
MDC = 0.75 logit units
Required change on original scale (%)
20.0
10.0
0.0
-10.0
-20.0
0
10
20
30
40
50
60
70
80
90
100
LiverPRO-F2 (%)

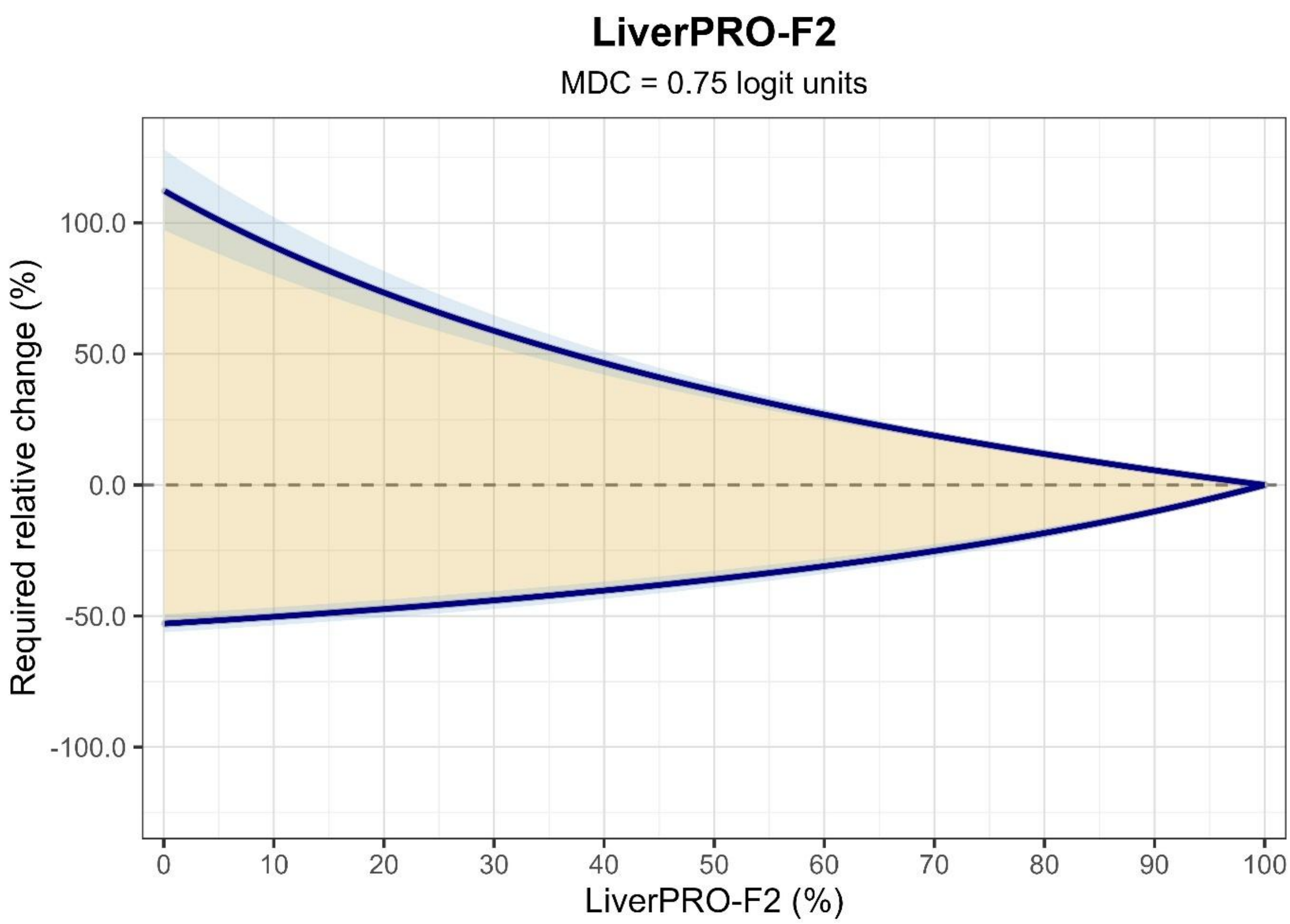
LiverPRO-F2
MDC = 0.75 logit units
Required relative change (%)
100.0
50.0
0.0
-50.0
-100.0
0
10
20
30
40
50
60
70
80
90
100
LiverPRO-F2 (%)

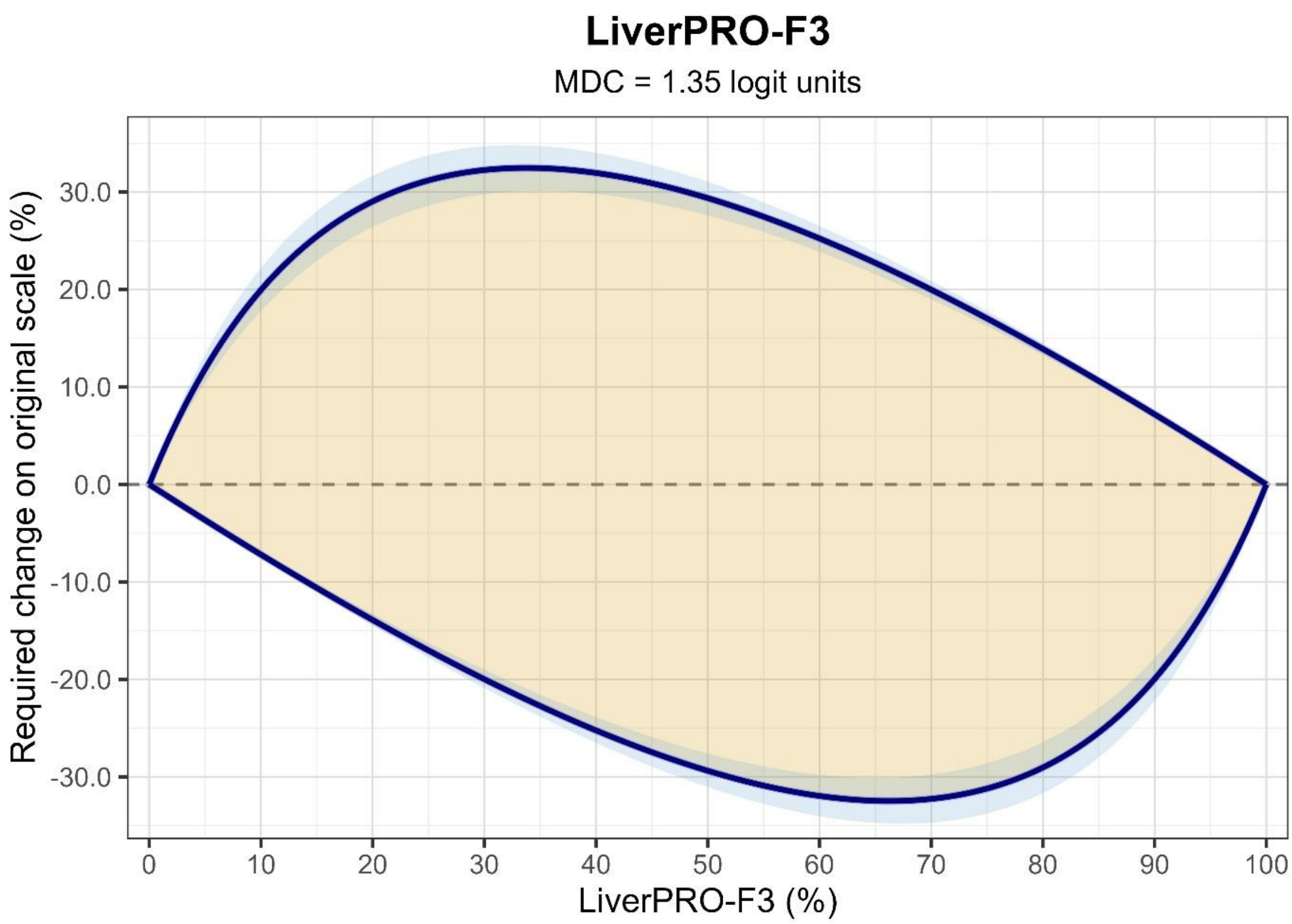
LiverPRO-F3
MDC = 1.35 logit units
Required change on original scale (%)
30.0
20.0
10.0
0.0
-10.0
-20.0
-30.0
0
10
20
30
40
50
60
70
80
90
100
LiverPRO-F3 (%)

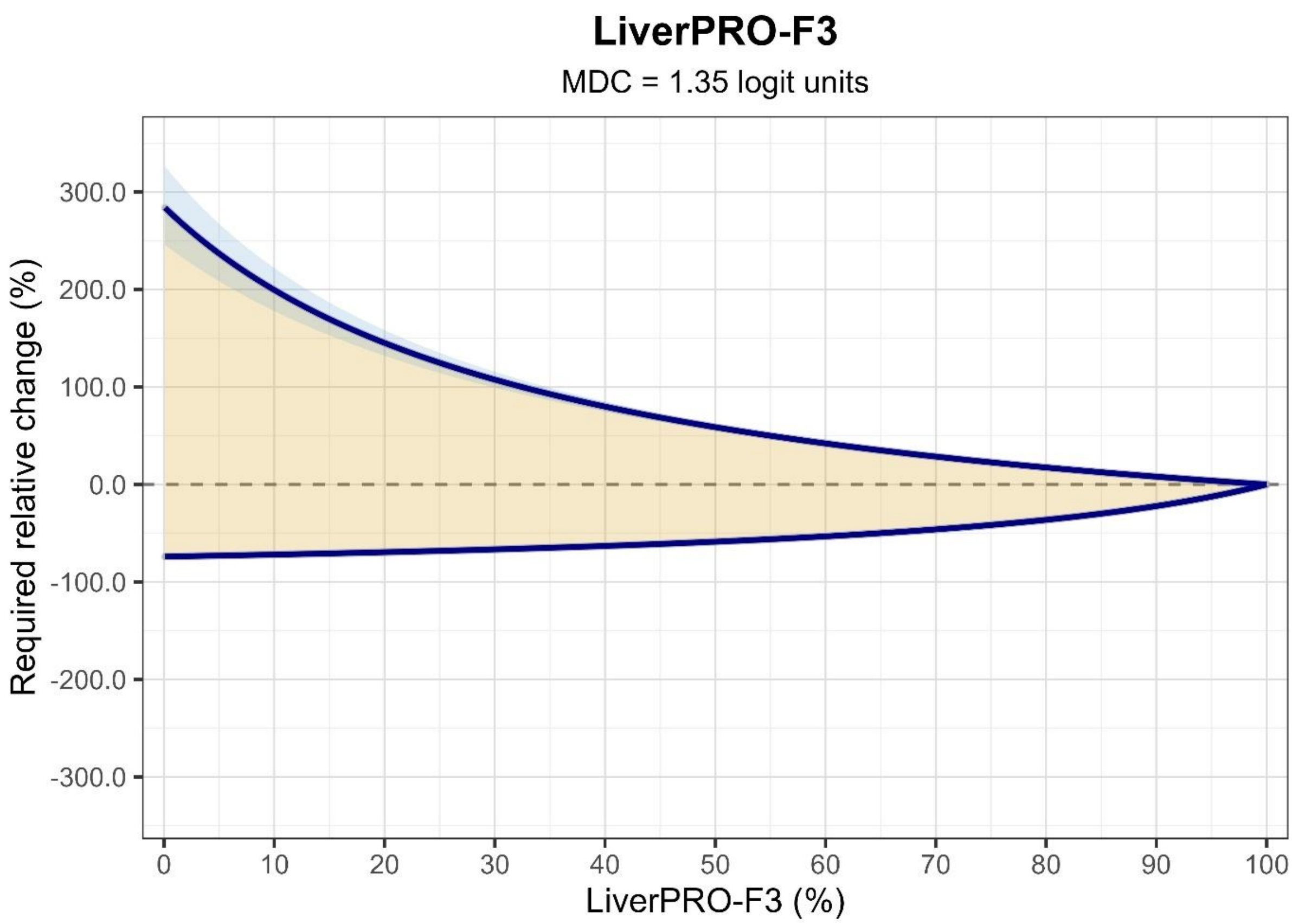
LiverPRO-F3
MDC = 1.35 logit units
Required relative change (%)
300.0
200.0
100.0
0.0
-100.0
-200.0
-300.0
0
10
20
30
40
50
60
70
80
90
100
LiverPRO-F3 (%)

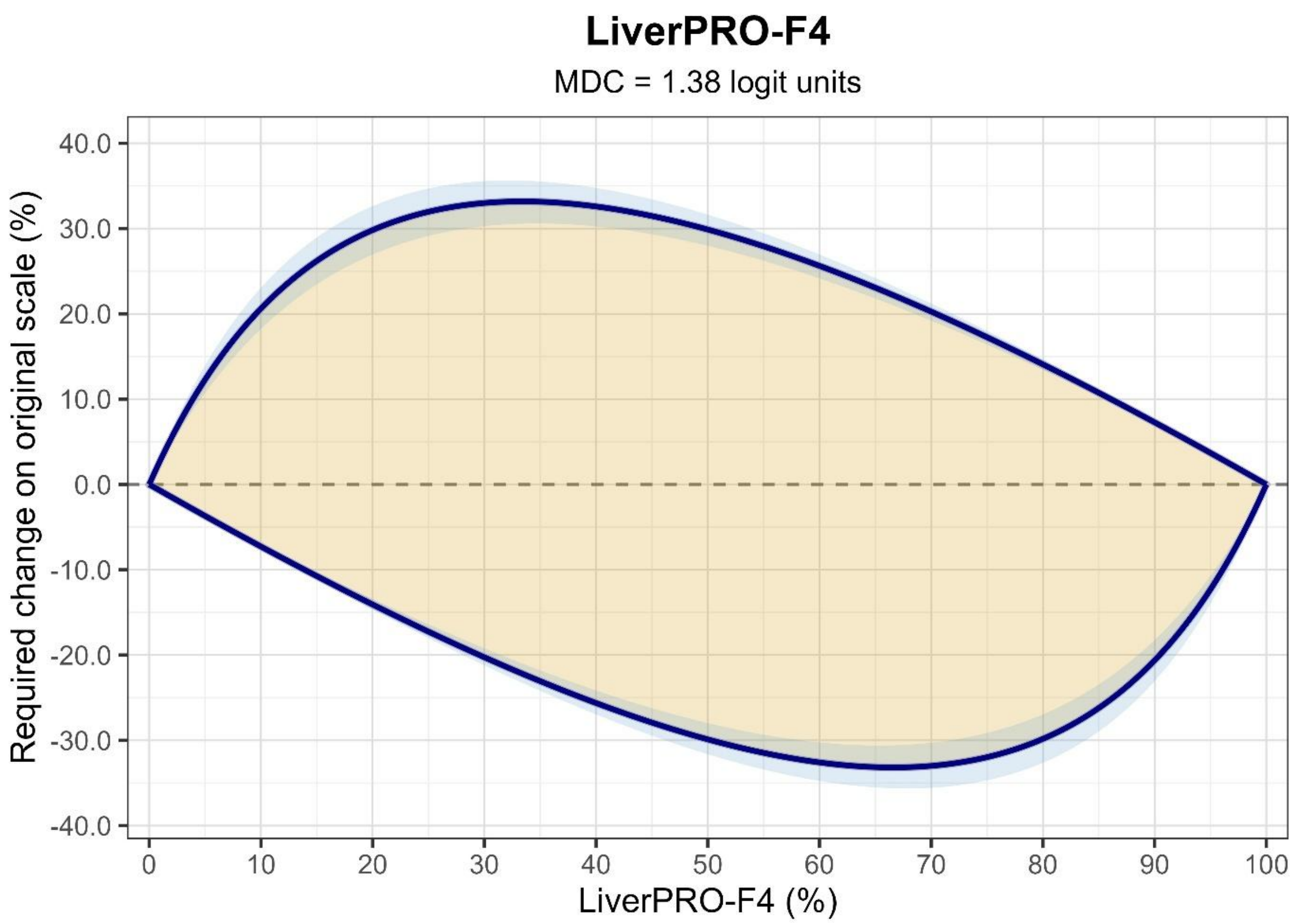
LiverPRO-F4
MDC = 1.38 logit units
Required change on original scale (%)
40.0
30.0
20.0
10.0
0.0
-10.0
-20.0
-30.0
-40.0
0
10
20
30
40
50
60
70
80
90
100
LiverPRO-F4 (%)

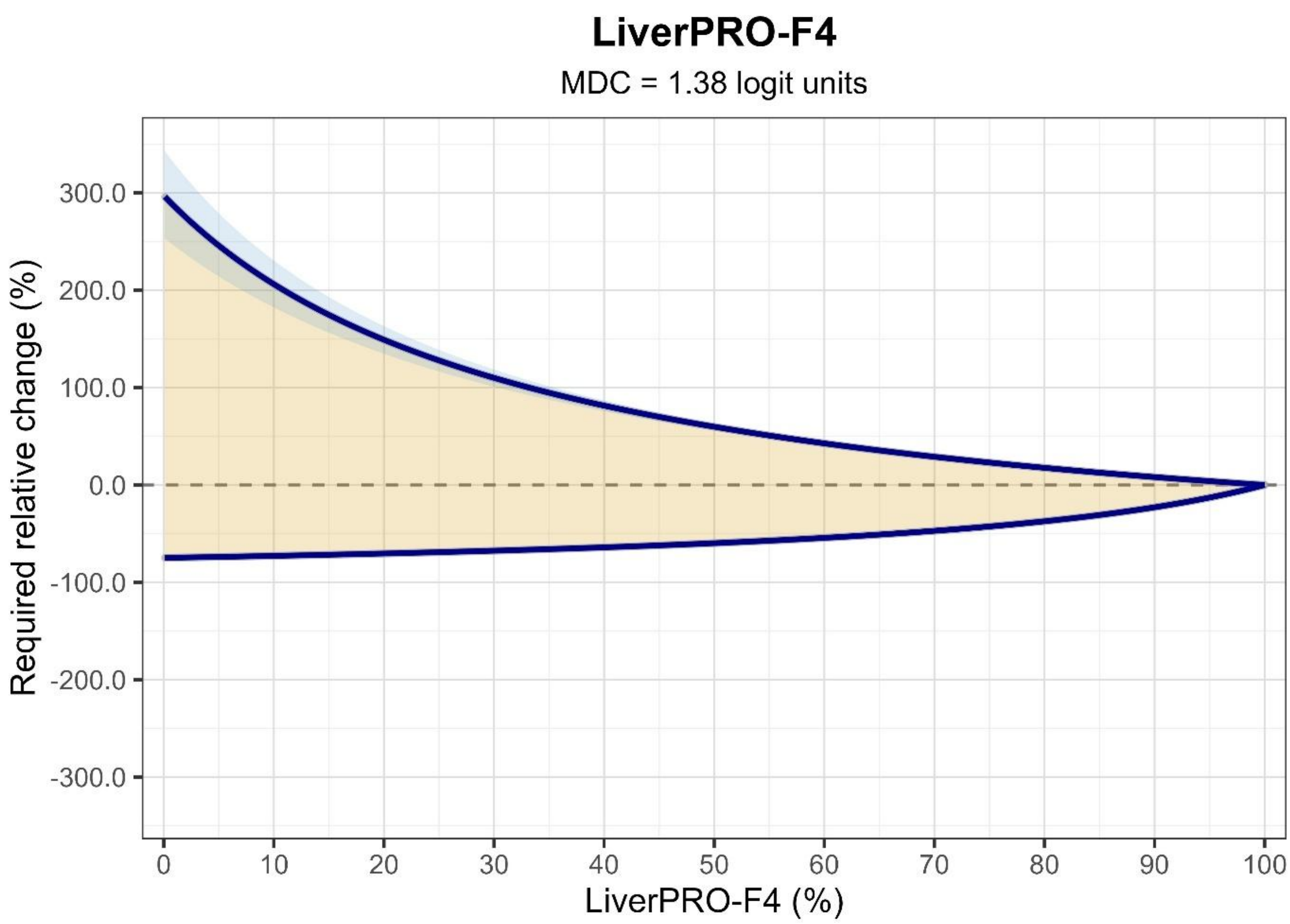
LiverPRO-F4
MDC = 1.38 logit units
Required relative change (%)
300.0
200.0
100.0
0.0
-100.0
-200.0
-300.0
0
10
20
30
40
50
60
70
80
90
100
LiverPRO-F4 (%)

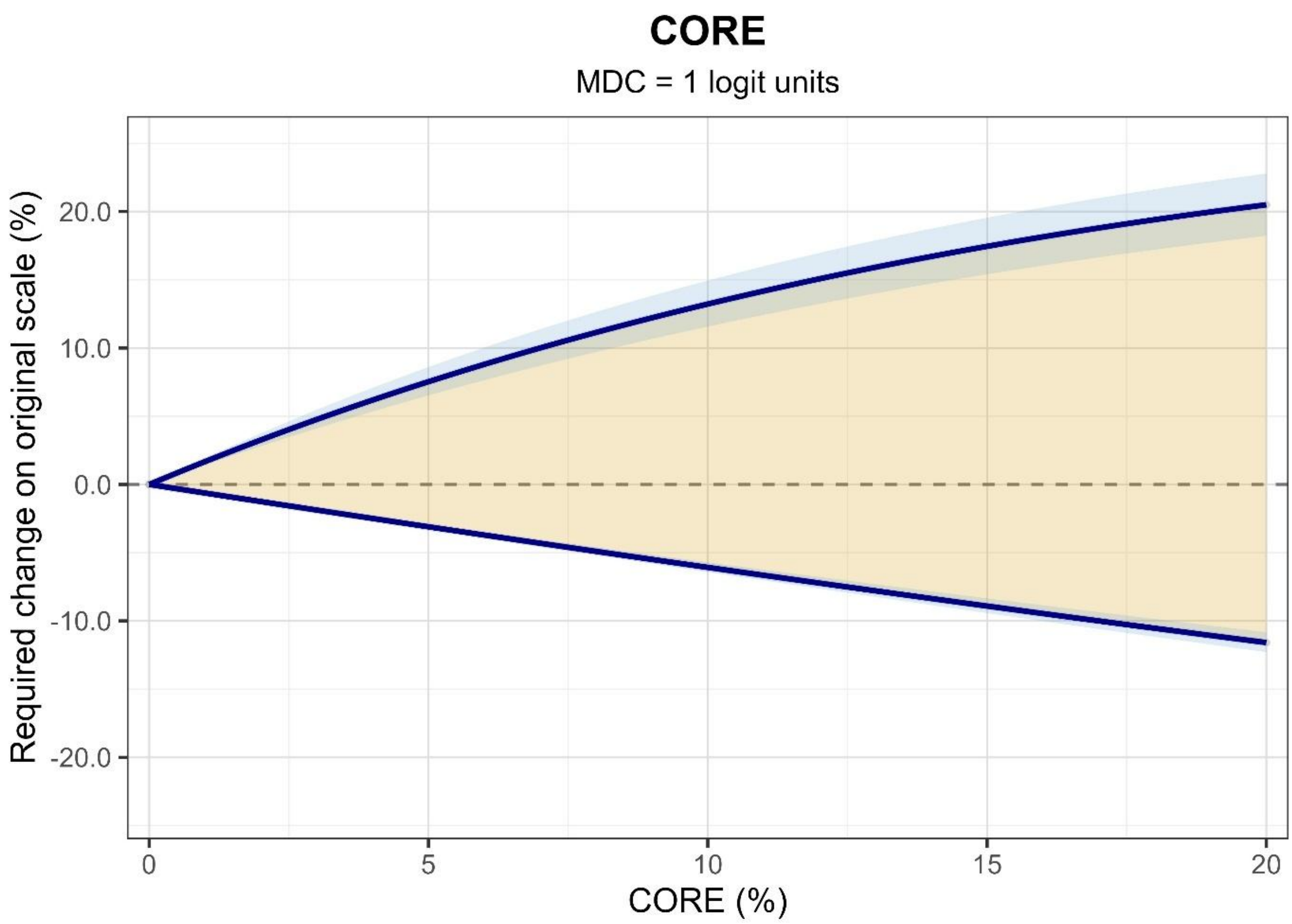
CORE
MDC = 1 logit units
Required change on original scale (%)
20.0
10.0
0.0
-10.0
-20.0
0
5
10
15
20
CORE (%)

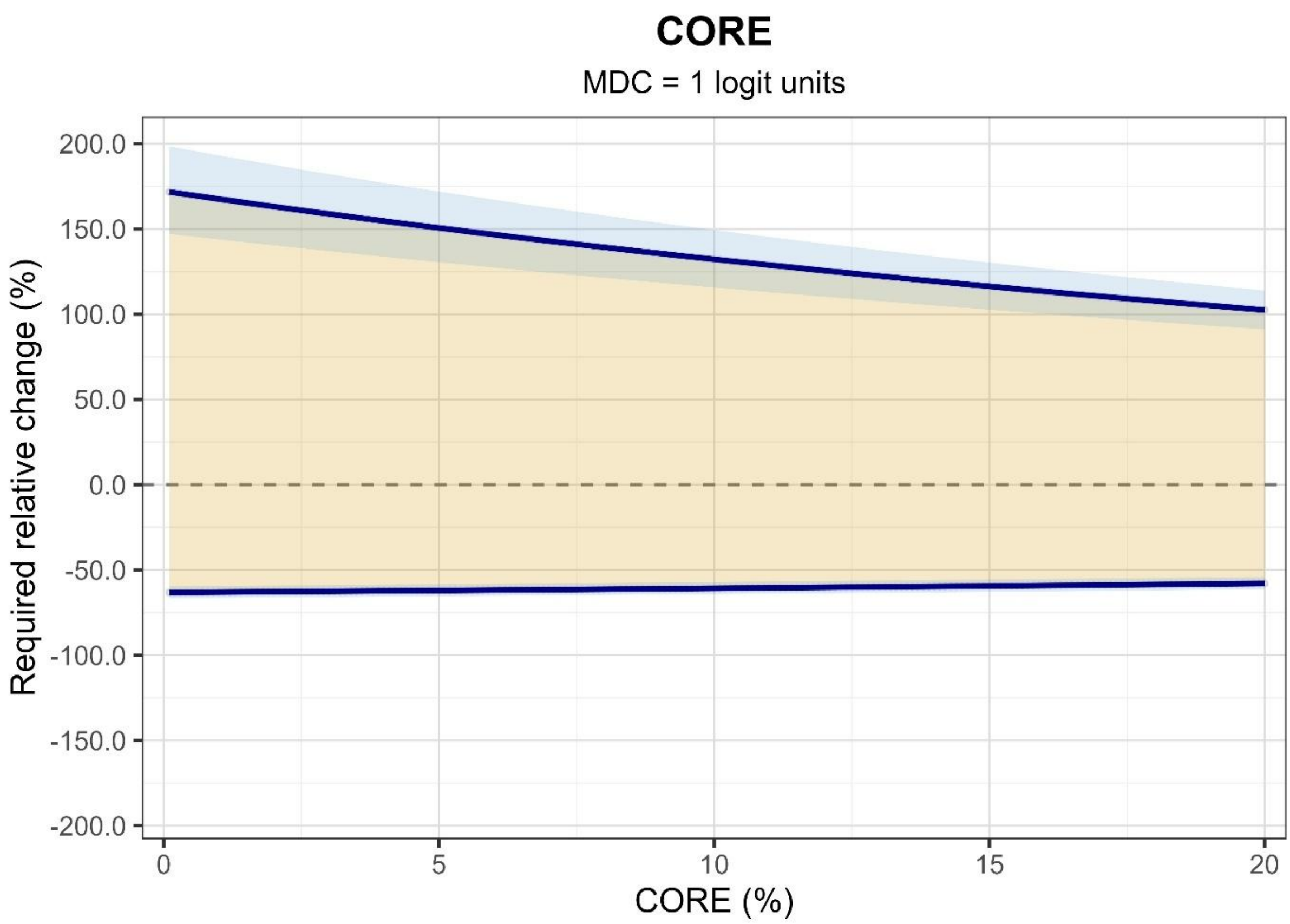
CORE
MDC = 1 logit units
Required relative change (%)
200.0
150.0
100.0
50.0
0.0
-50.0
-100.0
-150.0
-200.0
0
5
10
15
20
CORE (%)

Supplementary table 1. Measures of same-day variability in the pooled cohort from Repeatability cohort I and Repeatability cohort II (n=112) as well as stratified by <6 kPa and ≥6 kPa due to sample distribution. To mitigate the influence of outliers, an additional analysis is provided in which 10% of the cohort with the most extreme differences between measurement 1 and 2 were excluded. M/s-derived MDC and MDC% estimates were back-transformed to kPa-equivalent values, for MDC at an 8 kPa anchor.

	**Overall cohort**			**Excluding 10% extremes (optimistic estimates)**		
**Parameter**	**ICC (95%CI)**	**MDC (95%CI), original scale**	**MDC% (95%CI), %**	**ICC (95%CI)**	**MDC (95%CI), original scale**	**MDC% (95%CI), %**
**Pooled cohort, n=112**						
LSM, m/s	0.85 (0.79-0.90)	0.38 (0.30-0.47)	24.3 (18.8-29.9)	0.93 (0.90-0.96)	0.24 (0.21-0.28)	15.4 (13.6-17.1)
in kPa	-	-3.3 to +4.2 kPa[1]	-42.6 to +54.4%	-	-2.2 to +2.6 kPa[1]	-28.5 to +33.2%
LSM, kPa	0.88 (0.82-0.91)	4.1 (3.1-4.9)	47.4 (37.2-57.7)	0.94 (0.92-0.96)	2.6 (2.0-3.2)	30.7 (27.0-34.0)
**Pooled cohort <6 kPa, n=57**						
LSM, m/s	0.41 (0.18-0.61)	0.23 (0.17-0.29)	18.0 (13.6-23.0)	0.67 (0.49-0.80)	0.16 (0.14-0.18)	12.6 (10.9-14.3)
LSM, kPa	0.40 (0.17-0.60)	1.8 (1.3-2.2)	35.6 (27.1-45.3)	0.67 (0.49-0.80)	1.2 (1.1-1.4)	25.1 (21.8-28.4)
**Pooled cohort ≥6 kPa, n=55**						
LSM, m/s	0.78 (0.66-0.87)	0.50 (0.38-0.62)	29.4 (21.0-38.0)	0.89 (0.82-0.94)	0.33 (0.27-0.39)	18.7 (15.6-21.5)
LSM, kPa	0.84 (0.74-0.90)	5.5 (4.2-6.8)	57.1 (41.9-73.0)	0.91 (0.85-0.95)	3.9 (3.0-4.8)	37.3 (31.0-43.0)

[1] At 8 kPa

Supplementary table 2. Measures of variability in reproducibility cohorts I-III including Intraclass Correlation Coefficient (ICC), the minimal detectable change (MDC) on the original (absolute) scale as well as on the relative scale (MDC%). Complementary Bland-Altman analyses and Scatterplots of the respective cohorts are provided as Attachment I. To mitigate the influence of outliers, an additional analysis provides optimistic estimates in which 10% of the cohort with the largest absolute relative differences between measurement 1 and 2 were excluded. For LSM, variability was assessed on the shear-wave speed scale (m/s), since kPa represents a nonlinear transformation of m/s according to kPa = 3 × $(m/s)^2$. Therefore, m/s-derived MDC and MDC% estimates were back-transformed to kPa-equivalent values, for MDC at an 8 kPa anchor.

	**Overall cohort**			**Excluding 10% extremes (optimistic estimates)**		
**Parameter**	**ICC (95%CI)**	**MDC (95%CI), original scale**	**MDC% (95%CI), %**	**ICC (95%CI)**	**MDC (95%CI), original scale**	**MDC% (95%CI), %**
**Reproducibility cohort I - suspected MASLD (n=61)**						
FIB-4, points	0.70 (0.54-0.81)	0.6 (0.2-1.0)	38.2 (18.3-59.8)	0.97 (0.96-0.99)	0.2 (0.1-0.2)	15.8 (13.8-17.9)
APRI, points	0.59 (0.40-0.73)	0.3 (0.1-0.5)	46.9 (28.6-68.0)	0.93 (0.89-0.96)	0.1 (0.1-0.1)	23.4 (19.2-27.5)
LiverPRO-F2, %	0.96 (0.93-0.98)	7.2 (4.9-9.6)	NA	0.98 (0.96-0.99)	5.2 (4.1-6.4)	NA
LiverPRO-F3, %	0.83 (0.73-0.89)	4.2 (2.5-5.7)	NA	0.89 (0.82-0.94)	3.4 (1.8-5.1)	NA
LiverPRO-F4, %	0.78 (0.66-0.86)	3.1 (2.2-4.0)	NA	0.89 (0.83-0.94)	2.2 (1.5-2.8)	NA
CORE, %	0.85 (0.76-0.90)	0.5 (0.3-0.8)	NA	0.90 (0.83-0.94)	0.4 (0.2-0.6)	NA
LSM, m/s	0.66 (0.49-0.78)	0.24 (0.19-0.28)	17.6 (14.2-21.0)	0.81 (0.70-0.89)	0.18 (0.14-0.21)	12.9 (10.4-15.4)
in kPa	-	*-2.2 to +2.5 kPa*[1]	*-32.1 to +38.4%*	-	*-1.7 to +1.8 kPa*[1]	*-24.1 to +27.5%*
Agile 3+, %	0.74 (0.60-0.84)	9.3 (6.9-11.8)	NA	0.80 (0.69-0.88)	8.3 (5.7-11.2)	NA
Agile 4, %	0.58 (0.39-0.73)	2.2 (1.3-3.0)	NA	0.72 (0.57-0.83)	1.6 (1.0-2.2)	NA
**Reproducibility cohort II - suspected fibrosis (n=387)**						
FIB-4, points	0.85 (0.82-0.87)	1.1 (0.7-1.4)	44.8 (40.5-49.4)	0.95 (0.94-0.96)	0.6 (0.5-0.6)	32.1 (30.1-34.0)
APRI, points	0.84 (0.80-0.86)	0.4 (0.3-0.5)	53.7 (49.1-58.5)	0.95 (0.94-0.96)	0.2 (0.2-0.2)	39.7 (37.2-42.2)
LiverPRO-F2, %	0.95 (0.94-0.96)	13.7 (11.9-15.5)	NA	0.97 (0.97-0.98)	10.2 (9.3-11.1)	NA
LiverPRO-F3, %	0.91 (0.89-0.92)	12.5 (10.7-14.4)	NA	0.93 (0.92-0.95)	11.0 (9.2-13.0)	NA
LiverPRO-F4, %	0.87 (0.84-0.89)	13.0 (10.3-15.7)	NA	0.93 (0.92-0.95)	9.3 (7.8-10.7)	NA
CORE, %	0.92 (0.91-0.94)	3.3 (2.7-4.0)	NA	0.95 (0.94-0.96)	2.8 (2.1-3.5)	NA
ELF, points	0.85 (0.79-0.89)	1.2 (1.1-1.4)	13.0 (11.2-14.5)	0.90 (0.86-0.93)	1.0 (0.8-1.1)	10.1 (8.9-11.3)
**Reproducibility cohort III - suspected portal hypertension (n=88)**						
FIB-4, points	0.91 (0.86-0.94)	3.2 (2.1-4.3)	58.5 (48.8-67.1)	0.93 (0.90-0.96)	2.8 (1.6-4.0)	44.5 (37.5-50.6)

APRI, points	0.91 (0.87-0.94)	1.0 (0.6-1.4)	69.5 (58.5-79.4)	0.94 (0.90-0.96)	0.9 (0.5-1.3)	53.2 (45.1-60.5)
LiverPRO-F2, %	0.91 (0.87-0.94)	15.1 (11.8-18.1)	NA	0.95 (0.92-0.97)	10.3 (8.4-12.0)	NA
LiverPRO-F3, %	0.81 (0.54-0.91)	32.4 (27.9-36.7)	NA	0.80 (0.59-0.89)	30.2 (25.5-34.7)	NA
LiverPRO-F4, %	0.81 (0.52-0.91)	31.8 (27.0-36.5)	NA	0.83 (0.59-0.91)	29.3 (24.5-33.9)	NA

[1] At 8 kPa

<u>Abbreviations</u>: APRI - AST to platelet ratio index; ELF - Enhanced Liver Fibrosis test; FIB-4 - Fibrosis-4 score; ICC - intraclass correlation coefficient; LSM - liver stiffness measurement; MDC - minimal detectable change; MDC% - minimal detectable change (relative scale); NA - not applicable; SLD - steatotic liver disease; 95%CI - 95% confidence interval;

Supplementary table 3. MDC of logit-transformed probability-based NITs LiverPRO-F2, LiverPRO-F3, LiverPRO-F4 and CORE in a pooled cohort from Reproducibility cohorts I-III (n=536). Probability scores were transformed to the logit scale before analysis, and MDC is therefore expressed in logit units. For clinical interpretation, logit-scale MDC estimates were subsequently back-transformed to the original probability scale and displayed as the window of no change on the absolute and relative scale in which measurement variability cannot be excluded.

	Overall cohort		
**Parameter**	**MDC (95%CI), logit scale**	**No-change window (original percent probability)**	**No-change window (relative %)**
LiverPRO-F2, %	0.75 (0.68-0.82)		
5%		2.4-10.1%	-52-101%
10%		5.0-19.1%	-50-91%
25%		13.6-41.4%	-46-66%
50%		32.0-68.0%	-36-36%
75%		58.6-86.4%	-22-15%
90%		80.9-95.0%	-10-6%
95%		90.0-97.6%	-5-3%
LiverPRO-F3, %	1.35 (1.24-1.45)		
5%		1.4-16.8%	-73-237%
10%		2.8-29.9%	-72-200%
25%		8.0-56.2%	-68-125%
50%		20.6-79.4%	-59-59%
75%		43.8-92.0%	-42-23%
90%		70.0-97.2%	-22-8%
95%		83.2-98.7%	-12-4%
LiverPRO-F4, %	1.38 (1.26-1.49)		
5%		1.3-17.3%	-74-246%
10%		2.7-30.6%	-73-206%
25%		7.7-57.0%	-69-128%
50%		20.1-79.9%	-60-60%
75%		43.0-92.3%	-43-23%
90%		69.4-97.3%	-23-8%
95%		82.7-98.7%	-13-4%
CORE, %[1]	1.00 (0.91-1.10)		
0.1%		0.04-0.3%	-63-172%
0.4%		0.1-1.1%	-63-170%
1%		0.4-2.7%	-63-168%
2.5%		0.9-6.5%	-63-161%
5%		1.9-12.5%	-62-151%
10%		3.9-23.2%	-61-132%
15%		6.1-32.5%	-59-116%

[1] Only assessed n=448 patients in cohorts from secondary care;

Abbreviations: MDC - minimal detectable change; MDC% - minimal detectable change (relative scale); 95%CI - 95% confidence interval;

Supplementary table 4. Median values of LSM and derived scores Agile 3+ and Agile 4 at timepoint 1 and 2 and their respective median absolute and relative change with interquartile ranges (IQR).

Parameter	Timepoint 1	Timepoint 2	Change (absolute)	Change (relative, %)
**Reproducibility cohort II - suspected fibrosis**				
LSM, kPa	9.2 [7.9-11.1]	7.3 [5.7-9.7]	-1.8 [-3.5-(-0.3)]	-19.1 [-34.7-(-4.0)]%
Quartile 1 (n=97)	6.9 [6.6-7.4]	5.9 [4.9-7.2]	-0.8 [-1.8-0.0]	-12.3 [-25.8-0.0]%
Quartile 2 (n=97)	8.6 [8.30-8.9]	7.1 [5.8-8.1]	-1.7 [-2.8-(-0.5)]	-20.0 [-32.6-(-6.0)]%
Quartile 3 (n=97)	10.1 [9.6-10.4]	7.8 [6.4-9.6]	-2.1 [-3.8-(-0.5)]	-20.0 [-37.5-(-4.9)]%
Quartile 4 (n=96)	14.1 [11.9-21.2]	10.7 [7.4-16.4]	-4.2 [-7.1-(-1.3)]	-26.3 [-46.4-(-8.2)]%
Agile 3+, %	40.1 [24.9-62.1]	28.1 [14.3-49.8]	-7.2 [-18.4-(-0.2)]	-22.3 [-49.3-(-0.2)]%
Quartile 1 (n=97)	16.9 [11.1-21.4]	12.0 [7.7-16.8]	-3.2 [-7.8-1.3]	-27.0 [-49.7-10.3]%
Quartile 2 (n=97)	33.1 [29.0-37.2]	21.8 [15.8-32.7]	-8.7 [-18.0-(-0.8)]	-27.3 [-54.0-(-2.7)]%
Quartile 3 (n=97)	51.6 [46.3-56.2]	36.8 [22.9-50.2]	-14.7 [-26.2-(-2.0)]	-28.6 [-52.9-(-3.4)]%
Quartile 4 (n=96)	78.5 [68.6-91.3]	73.8 [45.1-87.6]	-8.0 [-23.0-0.6]	-9.6 [-34.7-0.6]%
Agile 4, %	5.8 [2.7-13.1]	3.0 [1.1-9.0]	-1.5 [-5.0-0.2]	-40.5 [-69.8-3.9]%
Quartile 1 (n=97)	1.4 [0.9-1.9]	0.9 [0.45-1.8]	-0.3 [-0.9-0.6]	-31.5 [-67.9-43.4]%
Quartile 2 (n=97)	4.0 [3.3-4.8]	2.8 [1.4-4.4]	-1.3 [-2.7-0.3]	-31.3 [-66.4-7.0]%
Quartile 3 (n=97)	8.5 [7.2-10.2]	3.7 [1.8-7.0]	-4.5 [-6.9-(-2.4)]	-60.5 [-81.2-(-21.2)]%
Quartile 4 (n=96)	23.5 [16.8-42.9]	17.0 [6.7-34.3]	-7.0 [-16.8-1.8]	-30.5 [-66.0-3.8]%
**Reproducibility cohort III - suspected portal hypertension**				
LSM, kPa	27.9 [18.0-51.9]	26.5 [16.8-46.9]	-2.1 [-9.2-2.4]	-9.2 [-26.4-10.2]%
Quartile 1 (n=22)	12.7 [11.6-14.4]	12.0 [9.4-15.4]	-1.4 [-2.7-1.5]	-9.0 [-24.9-10.3]%
Quartile 2 (n=22)	23.9 [21.5-26.0]	25.2 [21.1-28.9]	1.7 [-2.8-4.6]	7.0 [-10.8-21.8]%
Quartile 3 (n=22)	38.9 [33.9-43.3]	27.4 [21.7-39.7]	-10.2 [-17.5-4.3]	-25.2 [-44.3-10.5]%
Quartile 4 (n=22)	72.0 [65.6-75.0]	61.7 [49.0-74.1]	-8.9 [-14.5-0.0]	-14.0 [-20.8-0.0]%
Agile 3+, %	98.4 [88.4-99.4]	96.2 [89.6-99.2]	-0.1 [-2.6-1.1]	-0.1 [-2.7-1.1]%
Quartile 1 (n=22)	76.5 [66.3-81.5]	75.5 [52.9-88.0]	3.9 [-9.7-9.2]	4.8 [-15.0-11.0]%
Quartile 2 (n=22)	95.4 [93.2-96.7]	94.9 [92.0-97.3]	-0.4 [-2.1-2.1]	-0.5 [-2.3-2.2]%
Quartile 3 (n=22)	98.8 [98.6-99.1]	98.4 [94.4-99.2]	-0.4 [-4.2-(-0.1)]	-0.4 [-4.2-(-0.1)]%
Quartile 4 (n=22)	99.7 [99.5-99.7]	99.6 [99.1-99.7]	0.0 [-0.4-0.0]	0.0 [-0.4-0.0]%
Agile 4, %	77.2 [48.3-91.6]	73.5 [49.4-91.3]	-0.3 [-8.1-5.8]	-0.3 [-15.2-8.9]%
Quartile 1 (n=22)	27.4 [14.6-38.0]	33.6 [8.3-45.5]	0.8 [-8.3-12.8]	-3.0 [-37.1-61.1]%
Quartile 2 (n=22)	69.7 [58.0-73.0]	69.8 [56.2-72.8]	-1.0 [-8.5-10.4]	-1.6 [-16.1-15.7]%
Quartile 3 (n=22)	86.0 [82.1-89.2]	85.3 [80.3-90.5]	-1.2 [-7.8-5.5]	-1.5 [-8.7-6.3]%
Quartile 4 (n=22)	94.4 [92.7-96.0]	94.7 [92.1-96.6]	-0.2 [-3.0-0.3]	-0.2 [-3.1-0.3]%

Abbreviations: IQR - interquartile range; LSM - liver stiffness measurement;

Supplementary table 5. Median and mean values of LSM and derived scores Agile 3+ and Agile 4 within the background populations for Reproducibility Cohort II and Reproducibility Cohort III, respectively.

Parameter	Mean (SD)	Median (IQR)
**Background population for Reproducibility cohort II - suspected fibrosis**		
LSM, kPa	5.1 (3.4)	4.5 [3.7-5.5]
LSM, m/s	1.28 (0.29)	1.23 [1.11-1.35]
LSM, log(kPa)	1.55 (0.37)	1.50 [1.31-1.71]
Agile 3+, %	15.1 (15.4)	10.2 [5.8-18.1]
Agile 3+, logit	-2.07 (1.17)	-2.18 [-2.79-(-1.51)]
Agile 4, %	2.2 (6.7)	0.7 [0.3-1.6]
Agile 4, logit	-4.92 (1.46)	-4.96 [-5.83-(-4.10)]
**Background population for Reproducibility cohort III - suspected portal hypertension**		
LSM, kPa	10.0 (11.3)	6.1 [4.7-9.6]
LSM, m/s	1.67 (0.72)	1.43 [1.25-1.79]
LSM, log(kPa)	2.00 (0.68)	1.81 [1.55-2.26]
Agile 3+, %	29.2 (31.8)	13.9 [4.7-46.3]
Agile 3+, logit	-1.32 (2.48)	-1.83 [-3.00-(-0.15)]
Agile 4, %	11.4 (23.3)	1.3 [0.3-6.9]
Agile 4, logit	-3.94 (2.60)	-4.32 [-5.70-(-2.60)]

Abbreviations: IQR - interquartile range; LSM - liver stiffness measurement; SD - standard deviation;

Supplementary table 6. Measures of variability in reproducibility cohorts I-III when restricting the cohorts to measurement taken within 28 days. To mitigate the influence of outliers, an additional analysis provides optimistic estimates in which 10% of the cohort with the largest absolute relative differences between measurement 1 and 2 were excluded. For LSM, variability was assessed on the shear-wave speed scale (m/s), since kPa represents a nonlinear transformation of m/s according to kPa = 3 × (m/s)². Therefore, m/s-derived MDC and MDC% estimates were back-transformed to kPa-equivalent values, for MDC at an 8 kPa anchor.

	Overall cohort			Excluding 10% extremes (optimistic estimates)		
**Parameter**	**ICC (95%CI)**	**MDC (95%CI), original scale**	**MDC% (95%CI), %**	**ICC (95%CI)**	**MDC (95%CI), original scale**	**MDC% (95%CI), %**
**Reproducibility cohort I - suspected MASLD (n=37)**						
FIB-4, points	0.94 (0.84-0.97)	0.2 (0.2-0.3)	23.7 (17.3-30.4)	0.98 (0.94-0.99)	0.2 (0.1-0.2)	16.4 (13.7-19.3)
APRI, points	0.89 (0.73-0.95)	0.1 (0.1-0.2)	32.7 (21.8-44.1)	0.94 (0.86-0.97)	0.1 (0.1-0.1)	21.5 (17.4-25.4)
LiverPRO-F2, %	0.97 (0.95-0.99)	6.1 (4.2-7.9)	NA	0.98 (0.95-0.99)	5.4 (3.9-7.2)	NA
LiverPRO-F3, %	0.94 (0.88-0.97)	2.3 (1.4-3.1)	NA	0.95 (0.91-0.98)	2.1 (1.1-3.1)	NA
LiverPRO-F4, %	0.86 (0.75-0.93)	2.4 (1.5-3.3)	NA	0.91 (0.82-0.96)	2.0 (1.2-2.9)	NA
CORE, %	0.89 (0.79-0.94)	0.5 (0.2-0.8)	NA	0.89 (0.79-0.95)	0.5 (0.2-0.8)	NA
LSM, m/s	0.65 (0.41-0.80)	0.25 (0.18-0.31)	17.6 (12.9-22.2)	0.81 (0.65-0.90)	0.18 (0.13-0.23)	12.8 (9.3-16.0)
in kPa	-	*-2.2 to +2.6 kPa*[1]	*-32.2 to +38.4%*	-	*-1.7 to +1.9 kPa*[1]	*-23.9 to +27.2%*
Agile 3+, %	0.73 (0.53-0.85)	9.5 (6.1-13.0)	NA	0.84 (0.69-0.92)	6.7 (4.4-8.9)	NA
Agile 4, %	0.51 (0.23-0.72)	2.3 (1.0-3.5)	NA	0.69 (0.46-0.84)	1.5 (0.7-2.3)	NA
**Reproducibility cohort II - suspected fibrosis (n=167)**						
FIB-4, points	0.74 (0.67-0.80)	1.3 (0.7-1.9)	48.1 (40.3-56.7)	0.94 (0.92-0.95)	0.5 (0.4-0.6)	32.9 (29.5-36.2)
APRI, points	0.78 (0.71-0.83)	0.4 (0.2-0.6)	57.4 (49.3-66.1)	0.95 (0.93-0.96)	0.2 (0.1-0.2)	41.3 (37.0-45.3)
LiverPRO-F2, %	0.96 (0.95-0.97)	12.2 (10.1-14.3)	NA	0.98 (0.97-0.98)	9.2 (8.1-10.4)	NA
LiverPRO-F3, %	0.88 (0.84-0.91)	14.1 (10.9-17.3)	NA	0.92 (0.90-0.94)	11.5 (8.7-14.3)	NA
LiverPRO-F4, %	0.83 (0.78-0.87)	13.7 (9.7-17.4)	NA	0.91 (0.87-0.93)	10.3 (7.8-12.6)	NA
CORE, %	0.89 (0.86-0.92)	3.6 (2.5-4.7)	NA	0.94 (0.92-0.96)	2.7 (1.7-3.6)	NA
ELF, points	0.86 (0.78-0.91)	1.2 (1.0-1.4)	13.2 (11.0-15.3)	0.90 (0.85-0.94)	1.0 (0.8-1.2)	10.5 (8.9-12.2)
**Reproducibility cohort III - suspected portal hypertension (n=32)**						
FIB-4, points	0.95 (0.91-0.98)	2.0 (1.2-2.7)	47.3 (35.9-58.1)	0.98 (0.96-0.99)	1.2 (0.9-1.6)	34.3 (27.7-41.0)
APRI, points	0.95 (0.90-0.98)	0.5 (0.4-0.7)	58.8 (43.5-73.0)	0.97 (0.94-0.99)	0.4 (0.3-0.6)	42.6 (33.0-51.1)
LiverPRO-F2, %	0.83 (0.68-0.91)	15.1 (9.5-20.5)	NA	0.91 (0.81-0.96)	9.7 (7.1-12.0)	NA
LiverPRO-F3, %	0.90 (0.74-0.96)	23.4 (18.0-28.6)	NA	0.90 (0.77-0.95)	20.8 (14.9-26.1)	NA

LiverPRO-F4, %	0.87 (0.68-0.94)	26.6 (21.1-31.8)	NA	0.87 (0.71-0.94)	24.9 (18.8-30.5)	NA

[1] At 8 kPa

Abbreviations: APRI - AST to platelet ratio index; ELF - Enhanced Liver Fibrosis test; FIB-4 - Fibrosis-4 score; ICC - intraclass correlation coefficient; LSM - liver stiffness measurement; MDC - minimal detectable change; MDC% - minimal detectable change (relative scale); NA - not applicable; SLD - steatotic liver disease; 95%CI - 95% confidence interval;

SUPPLEMENTARY REFERENCES

1. Hruska P, Semmler G, Teufelhart M, Hofer H, Winker R, Mehl B, Rabitsch C, et al. Applicability and effectiveness of a formula-based low-carb diet in metabolic dysfunction-associated steatotic liver disease—a single-arm interventional study. Hepatobiliary Surg Nutr 2026.
2. Kjaergaard M, Lindvig KP, Thorhauge KH, Andersen P, Hansen JK, Kastrup N, Jensen JM, et al. Using the ELF test, FIB-4 and NAFLD fibrosis score to screen the population for liver disease. Journal of Hepatology 2023;79:277-286.
3. Hansen CD, Hansen JK, Israelsen M, Andersen P, Pikkupeura LM, Lindvig KP, Stinson SE, et al. Prevalence, severity and determinants of steatotic liver disease among individuals with metabolic and alcohol risk from the community. J Hepatol 2025;83:1278-1291.
4. Semmler G, Thöne P, Embacher J, Hruska P, Simonis L, Stopfer K, Fritz L, et al. Prevalence of Fibrosis and Applicability of Lab-based Noninvasive Tests From Primary to Tertiary Care. Clin Gastroenterol Hepatol 2025.
5. Obuchowski NA, Bullen J. Quantitative imaging biomarkers: Effect of sample size and bias on confidence interval coverage. Statistical Methods in Medical Research 2018;27:3139-3150.
6. Koo TK, Li MY. A Guideline of Selecting and Reporting Intraclass Correlation Coefficients for Reliability Research. J Chiropr Med 2016;15:155-163.
7. Bland JM, Altman DG. Measuring agreement in method comparison studies. Stat Methods Med Res 1999;8:135-160.
8. Barnett AG, van der Pols JC, Dobson AJ. Regression to the mean: what it is and how to deal with it. International Journal of Epidemiology 2005;34:215-220.
9. Linden A. Assessing regression to the mean effects in health care initiatives. BMC Medical Research Methodology 2013;13:119.

ATTACHMENT I: Full list of scatterplots and Bland-Altman plots of all investigated NITs within the 3 Repeatability and 3 Reproducibility cohorts

Scatterplots show the association between the first and second measurement for each NIT. The dashed black line represents the line of identity, indicating perfect agreement between both measurements. Black points indicate paired measurements included in the main analysis, while grey points represent the 10% largest absolute relative differences that were excluded in the sensitivity analysis on optimistic MDC/MDC%. The dark-blue line shows the reduced major-axis fit using all paired measurements, and the orange line shows the reduced major-axis fit after exclusion of the 10% with largest absolute relative differences. Where the orange line moves closer to the identity line, this indicates that the apparent disagreement or slope distortion is partly driven by the individuals with extreme test-retest differences. Slopes, correlation coefficients, and sample sizes are displayed as all pairs | pairs after exclusion.

For the Bland-Altman plots, the x axis shows the mean between the 2 measurements (measurement 1 + measurement 2) / 2, the y axis the difference for the given pair (measurement 2 - measurement 1). Important metrics include the “bias” (dark-blue line) corresponding to the average mean difference between measurement 2 and measurement 1, and the upper and lower limit of agreement (LoA, orange line), corresponding to the range in which 95% of the differences between two measurements are expected to lie. Again, grey points represent the 10% most extreme paired differences that were excluded in the sensitivity analysis

Repeatability cohort I

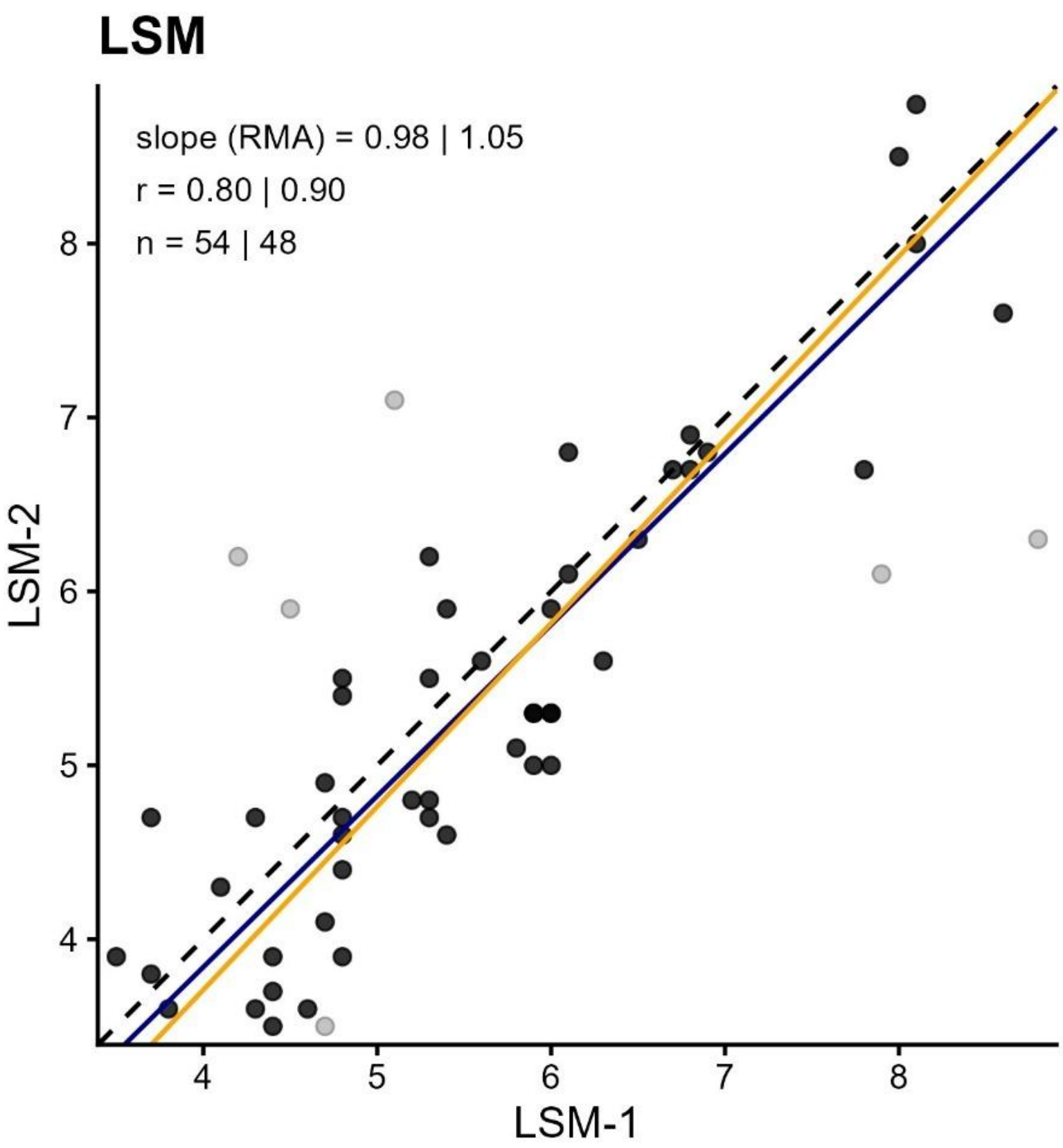

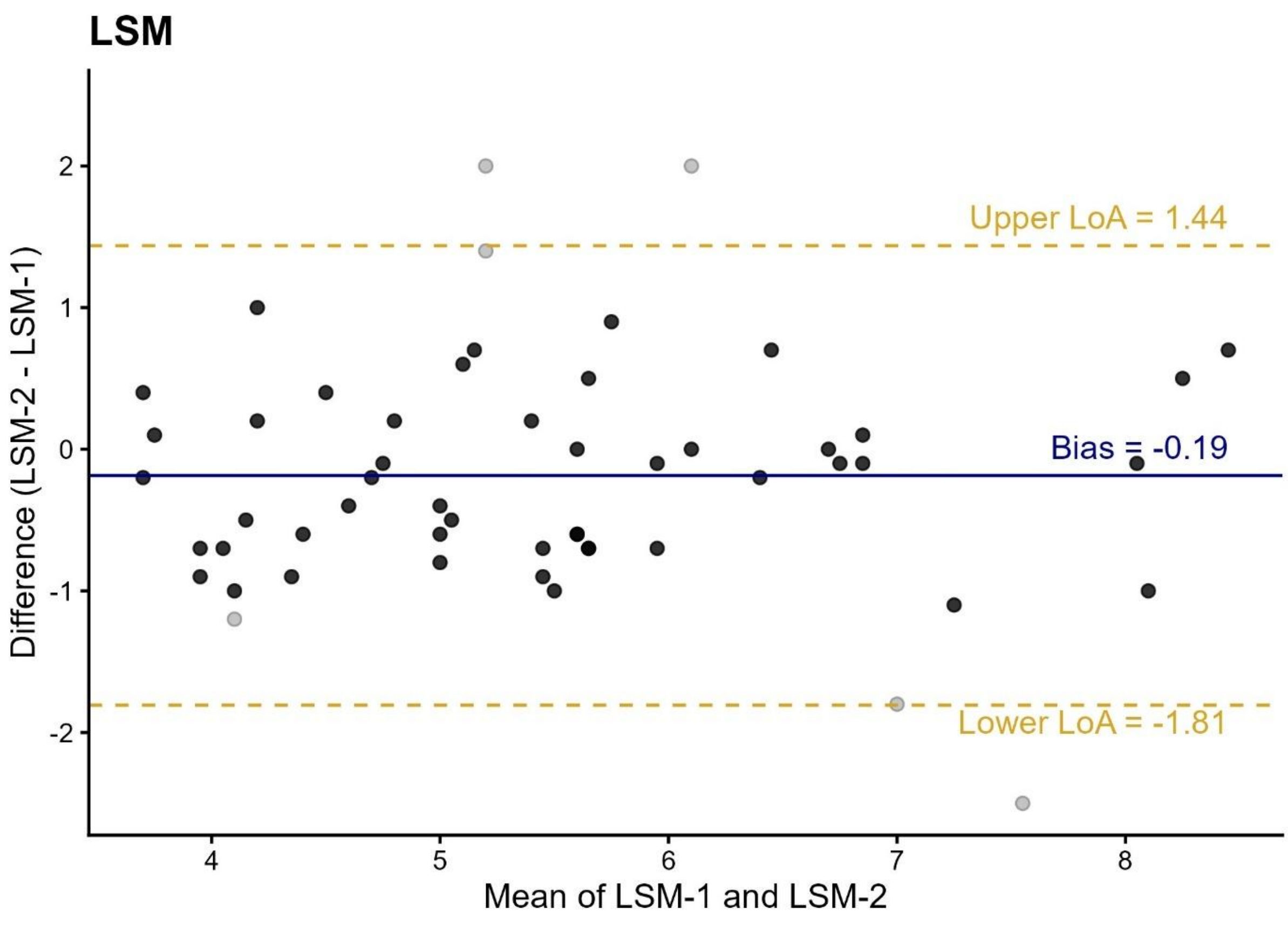

Repeatability cohort II

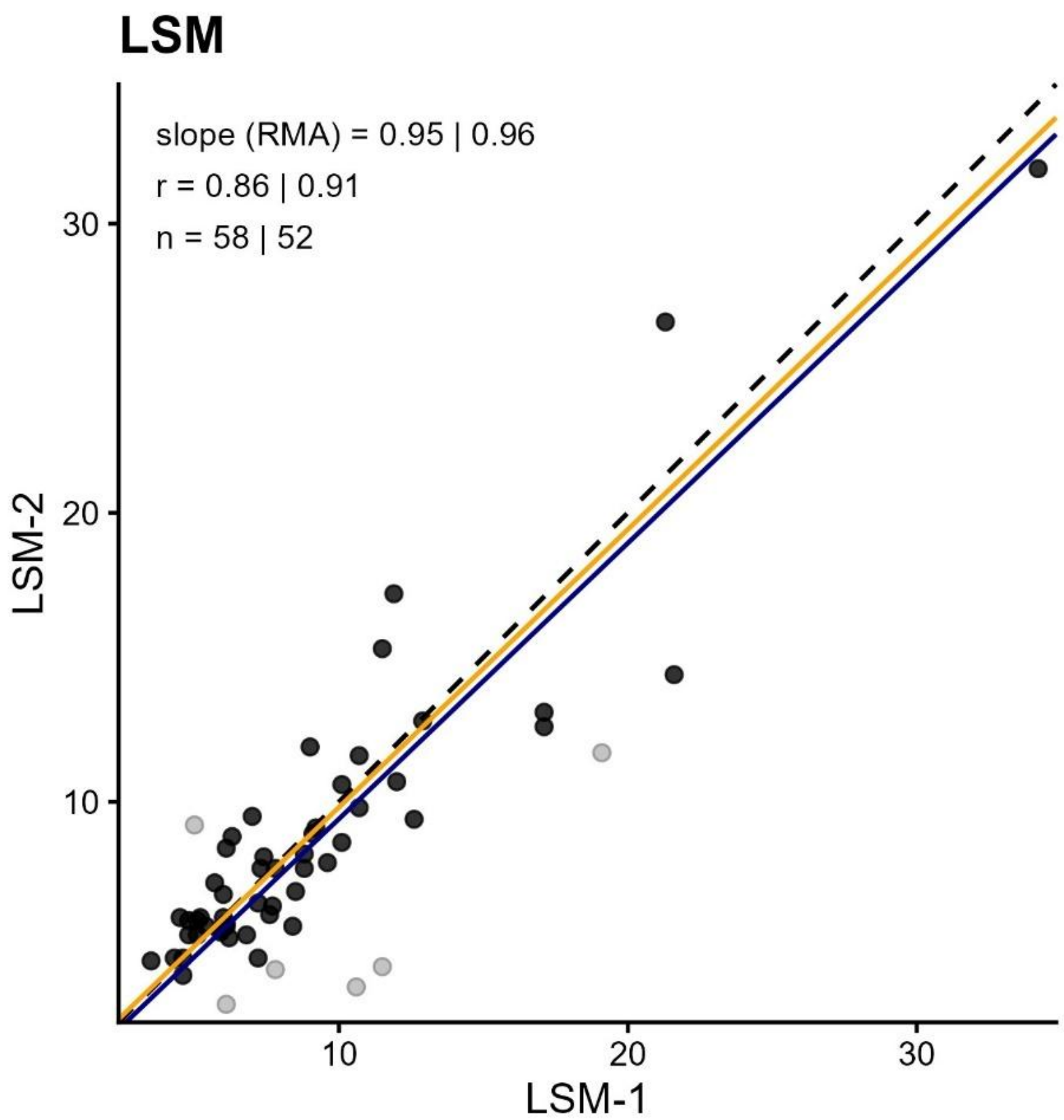

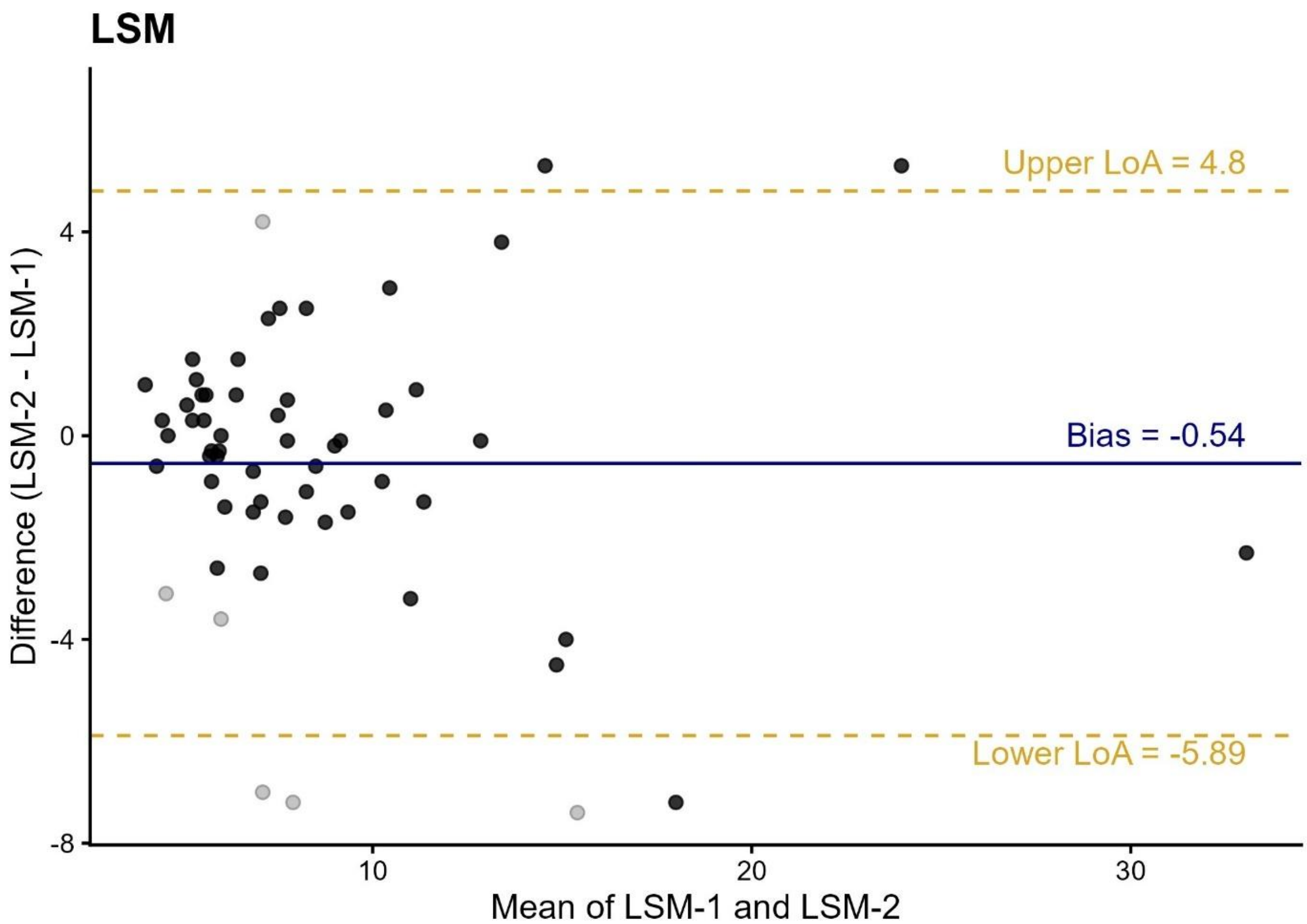

Repeatability cohort III

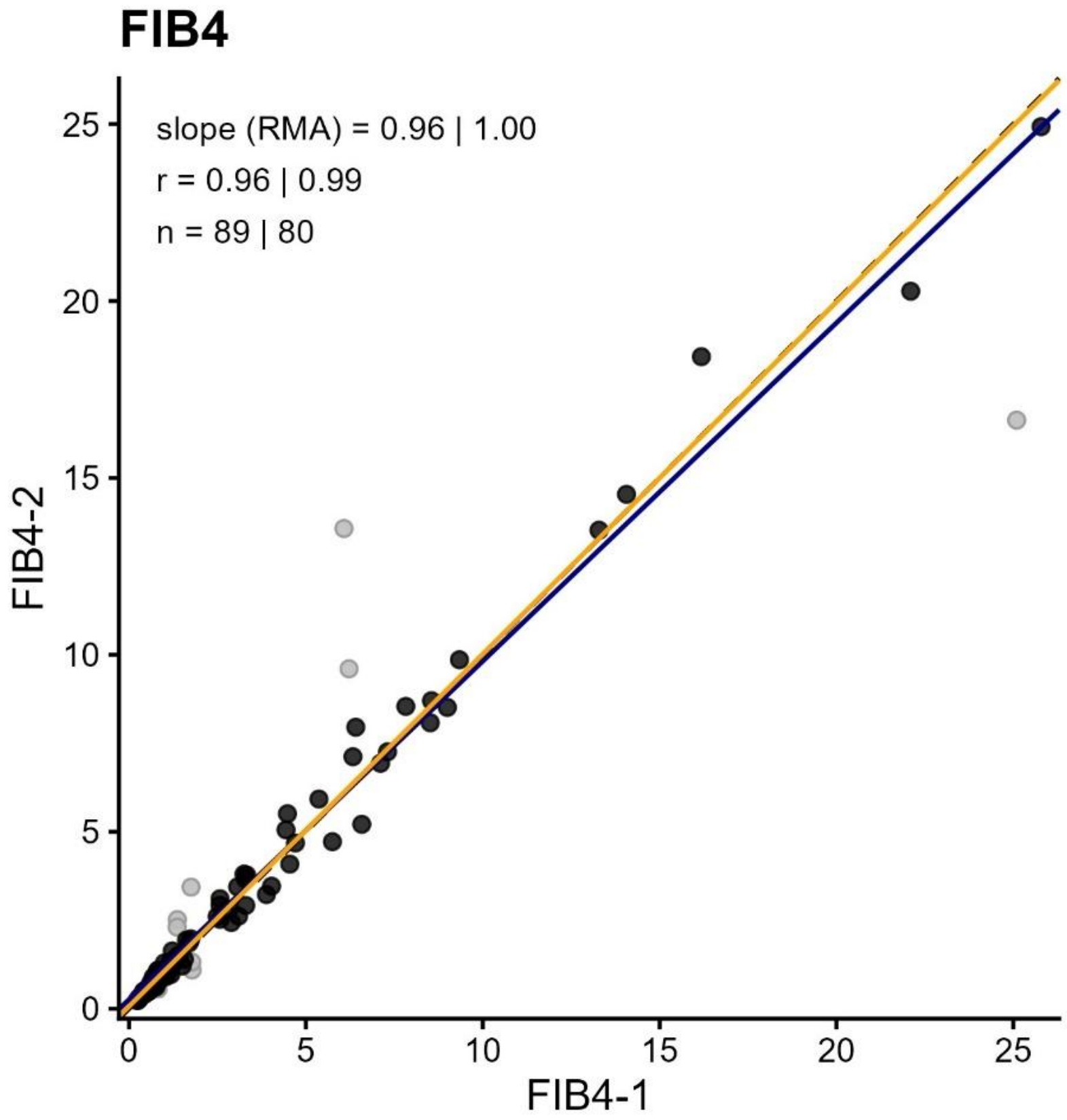

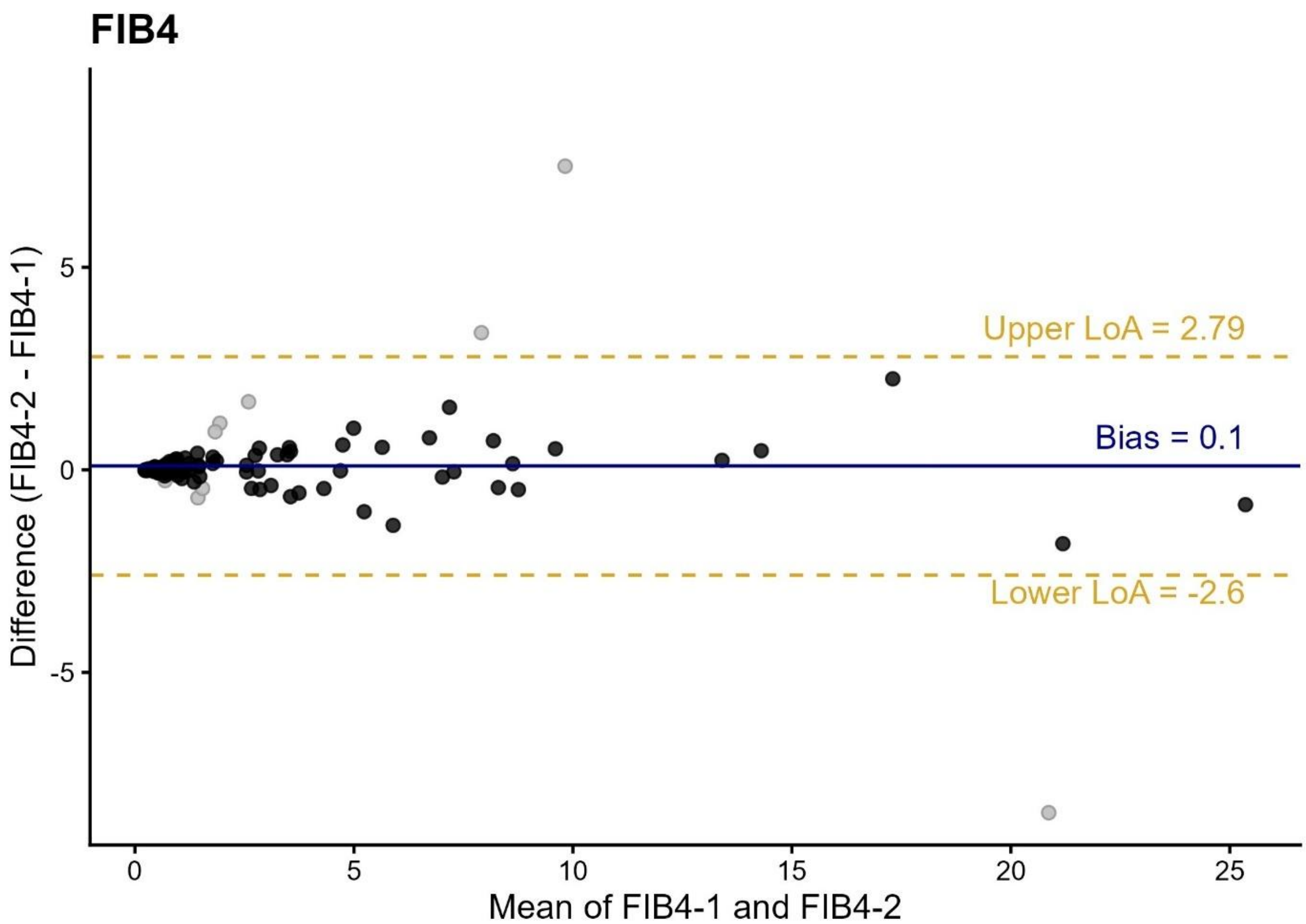

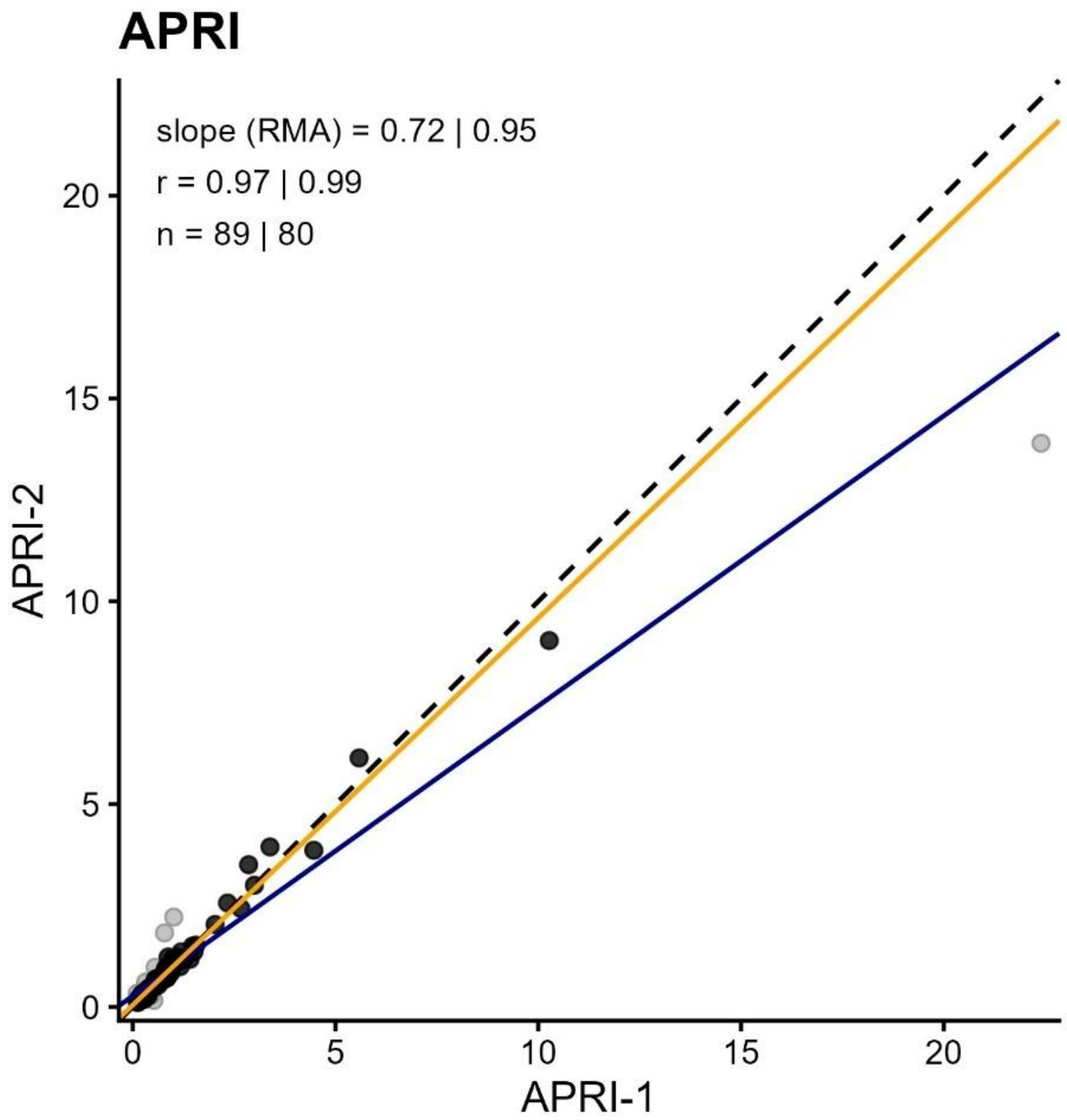
APRI
slope (RMA) = 0.72 | 0.95
r = 0.97 | 0.99
n = 89 | 80
APRI-2
APRI-1
0
5
10
15
20

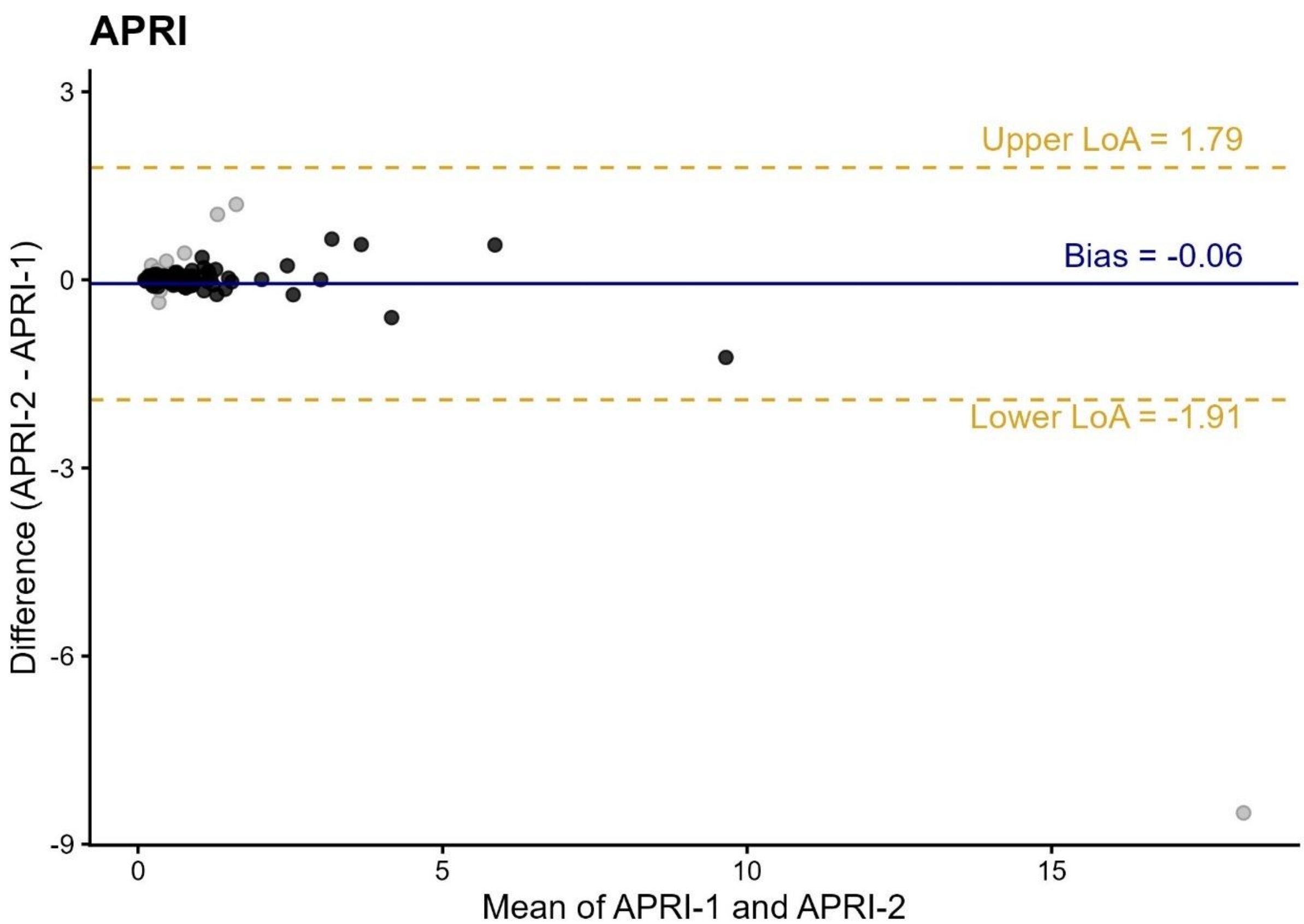
APRI
Upper LoA = 1.79
Bias = -0.06
Lower LoA = -1.91
Difference (APRI-2 - APRI-1)
Mean of APRI-1 and APRI-2
3
0
-3
-6
-9
5
10
15

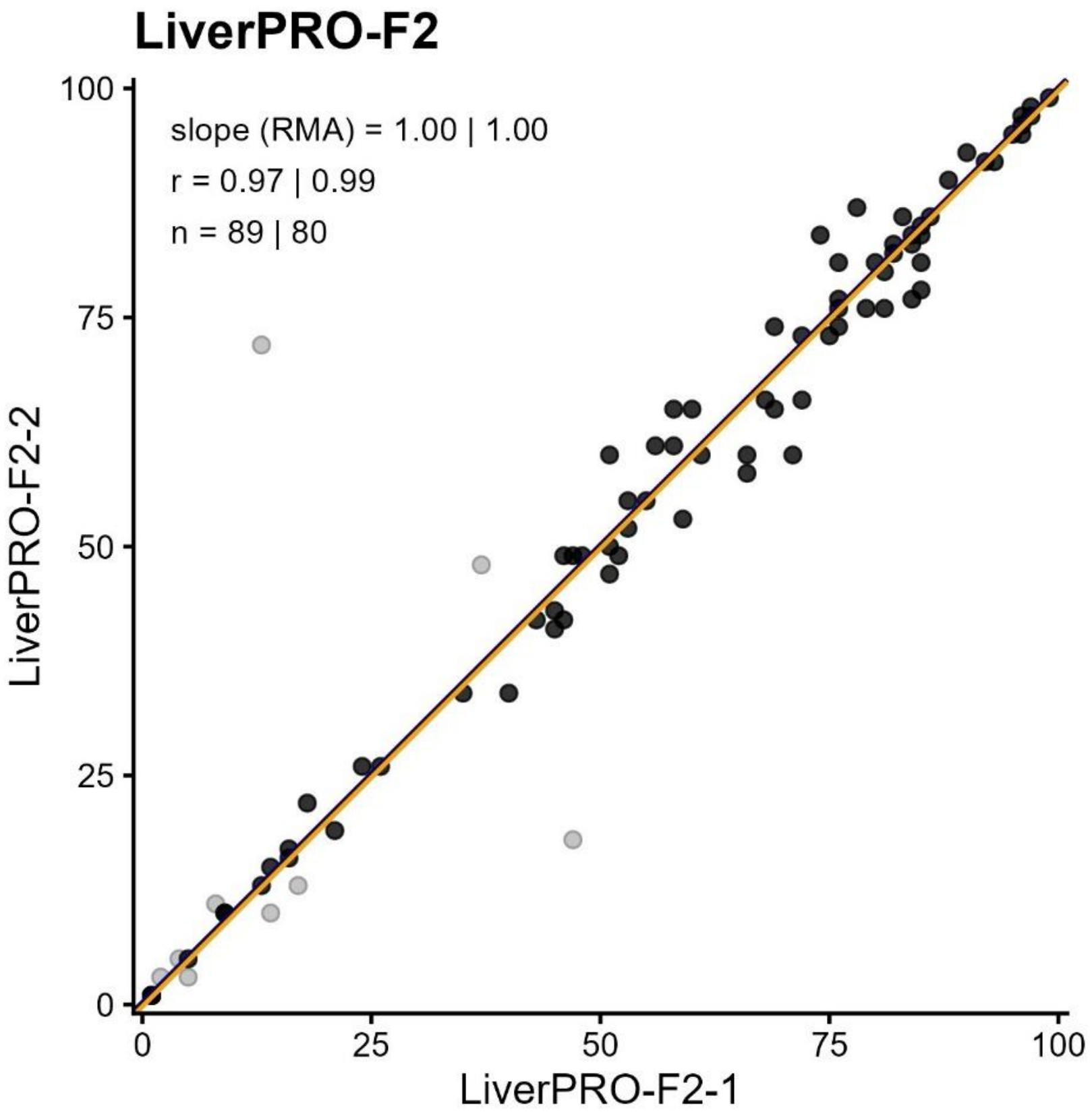
LiverPRO-F2
slope (RMA) = 1.00 | 1.00
r = 0.97 | 0.99
n = 89 | 80
LiverPRO-F2-2
LiverPRO-F2-1
0
25
50
75
100

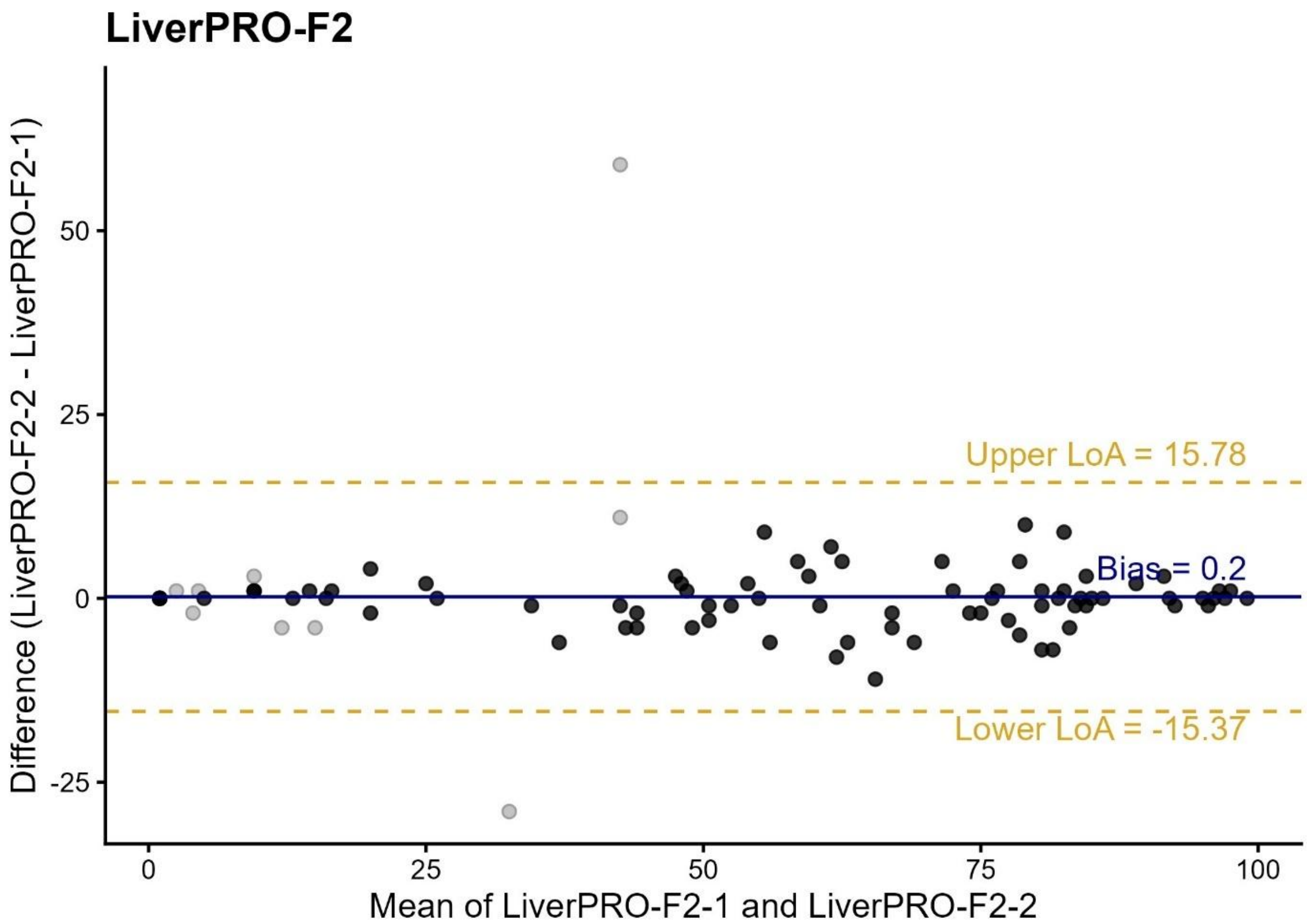
LiverPRO-F2
Upper LoA = 15.78
Bias = 0.2
Lower LoA = -15.37
Difference (LiverPRO-F2-2 - LiverPRO-F2-1)
Mean of LiverPRO-F2-1 and LiverPRO-F2-2
50
25
0
-25
0
25
50
75
100

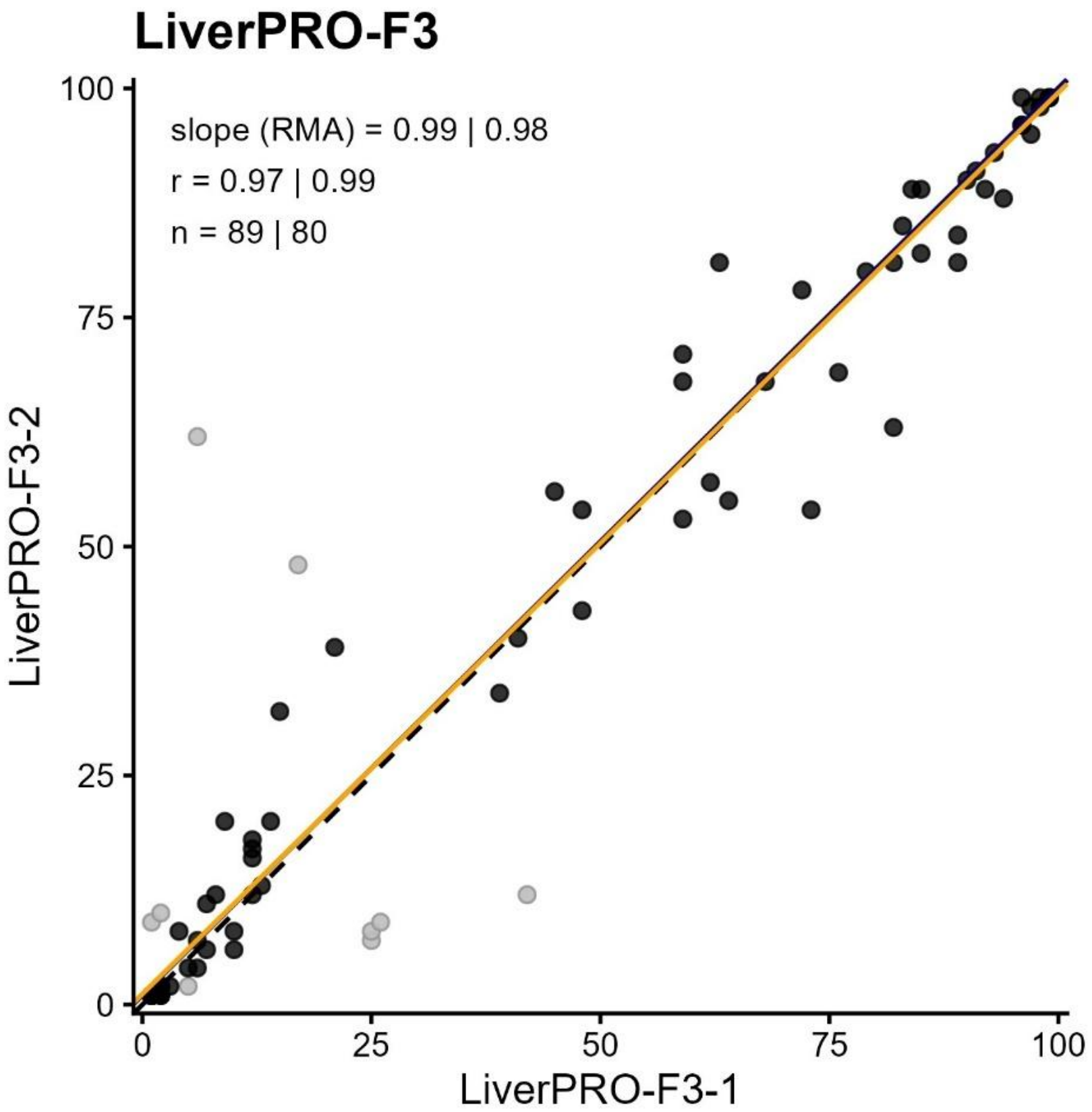
LiverPRO-F3
slope (RMA) = 0.99 | 0.98
r = 0.97 | 0.99
n = 89 | 80
LiverPRO-F3-2
LiverPRO-F3-1
0
25
50
75
100

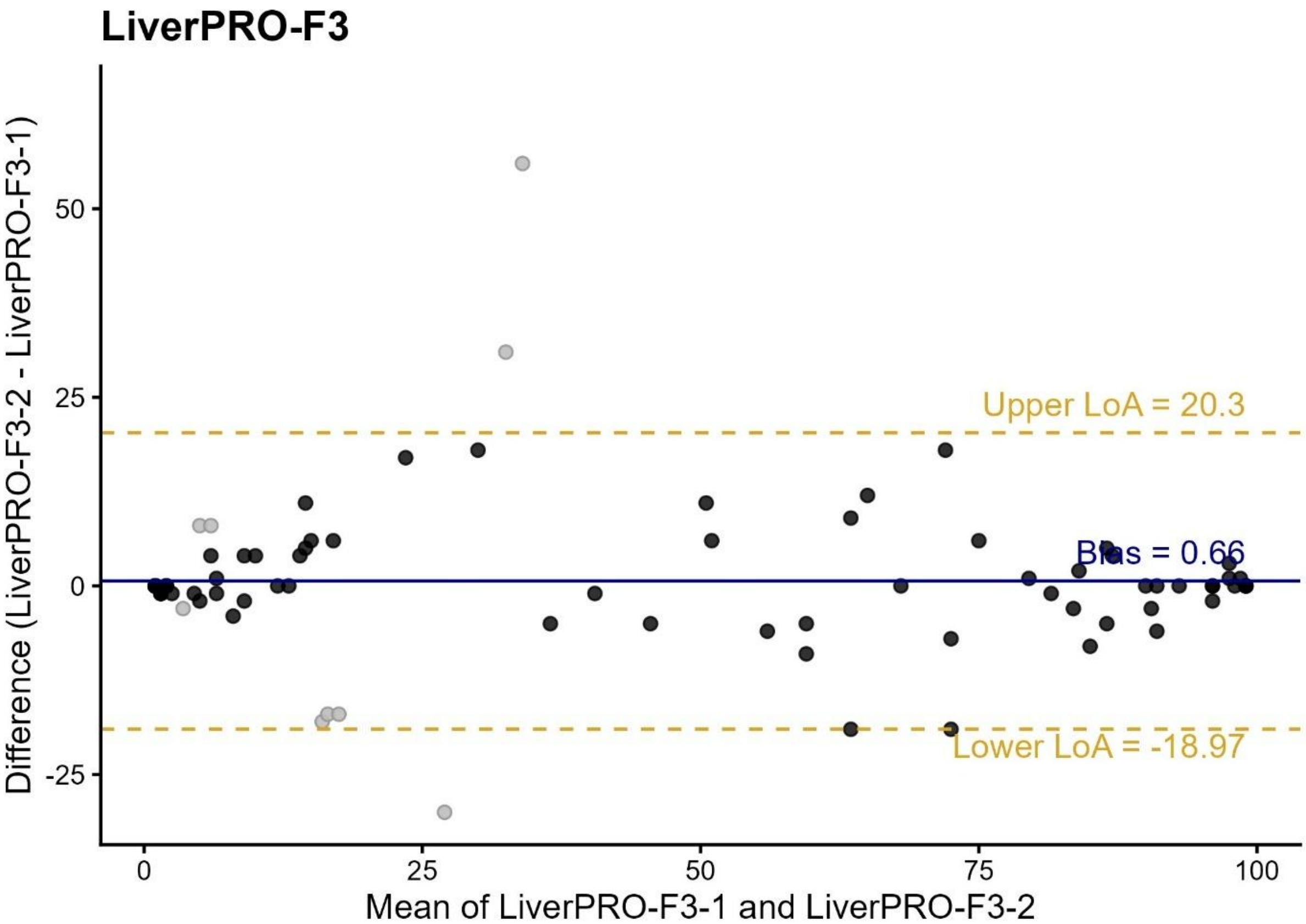
LiverPRO-F3
Upper LoA = 20.3
Bias = 0.66
Lower LoA = -18.97
Difference (LiverPRO-F3-2 - LiverPRO-F3-1)
Mean of LiverPRO-F3-1 and LiverPRO-F3-2
-25
0
25
50
75
100

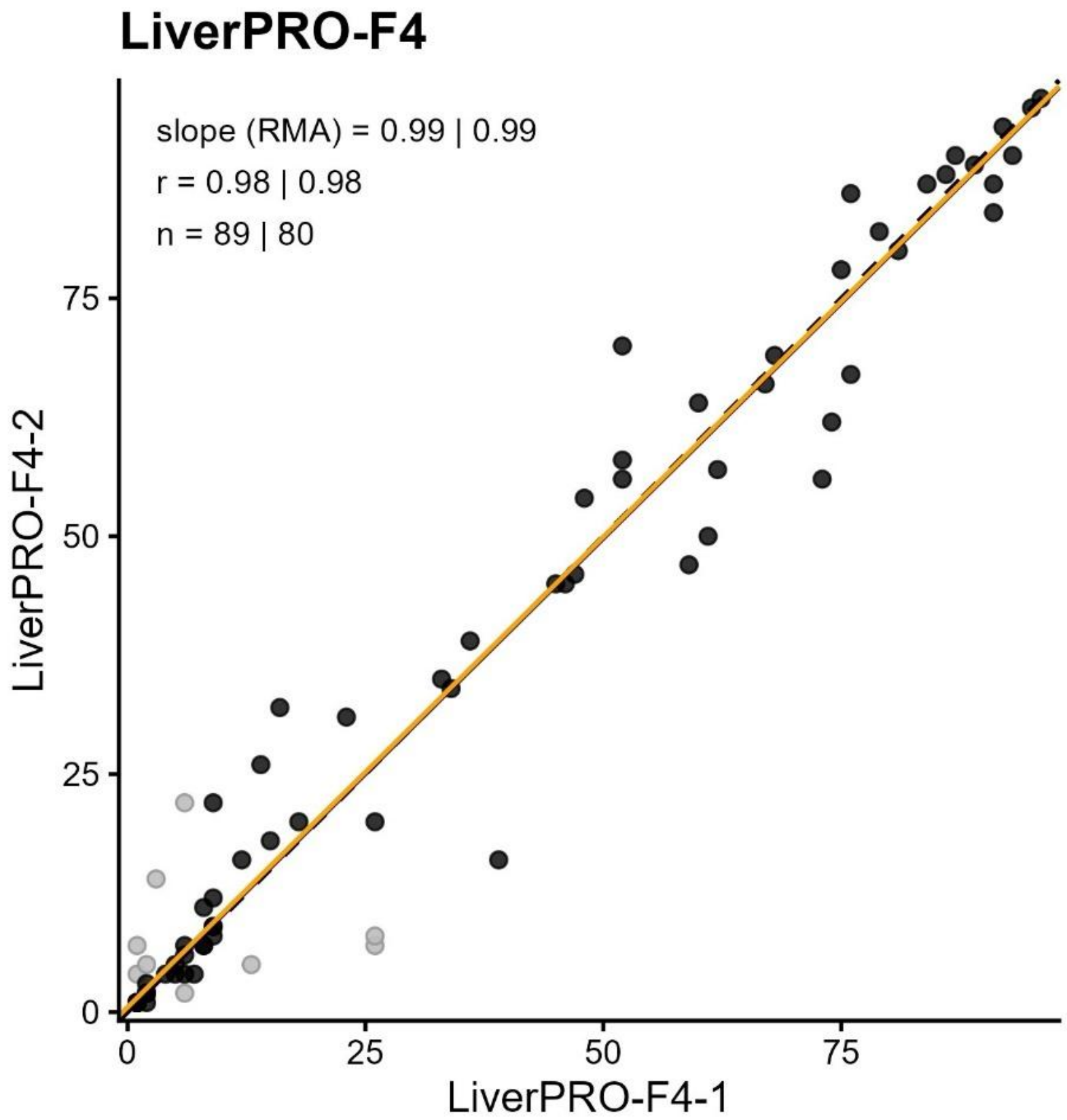
LiverPRO-F4
slope (RMA) = 0.99 | 0.99
r = 0.98 | 0.98
n = 89 | 80
LiverPRO-F4-2
LiverPRO-F4-1

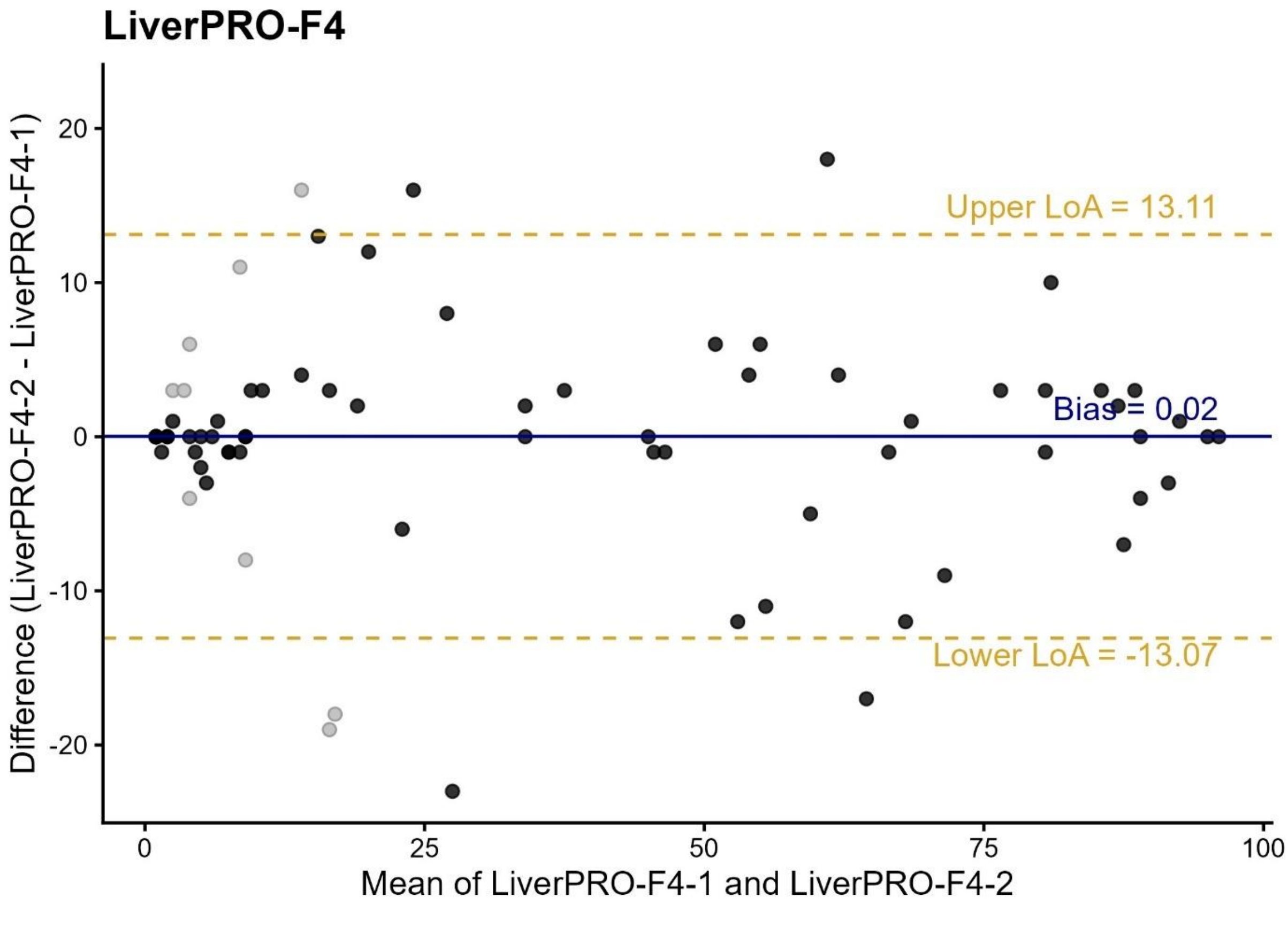
LiverPRO-F4
Upper LoA = 13.11
Bias = 0.02
Lower LoA = -13.07
Difference (LiverPRO-F4-2 - LiverPRO-F4-1)
Mean of LiverPRO-F4-1 and LiverPRO-F4-2

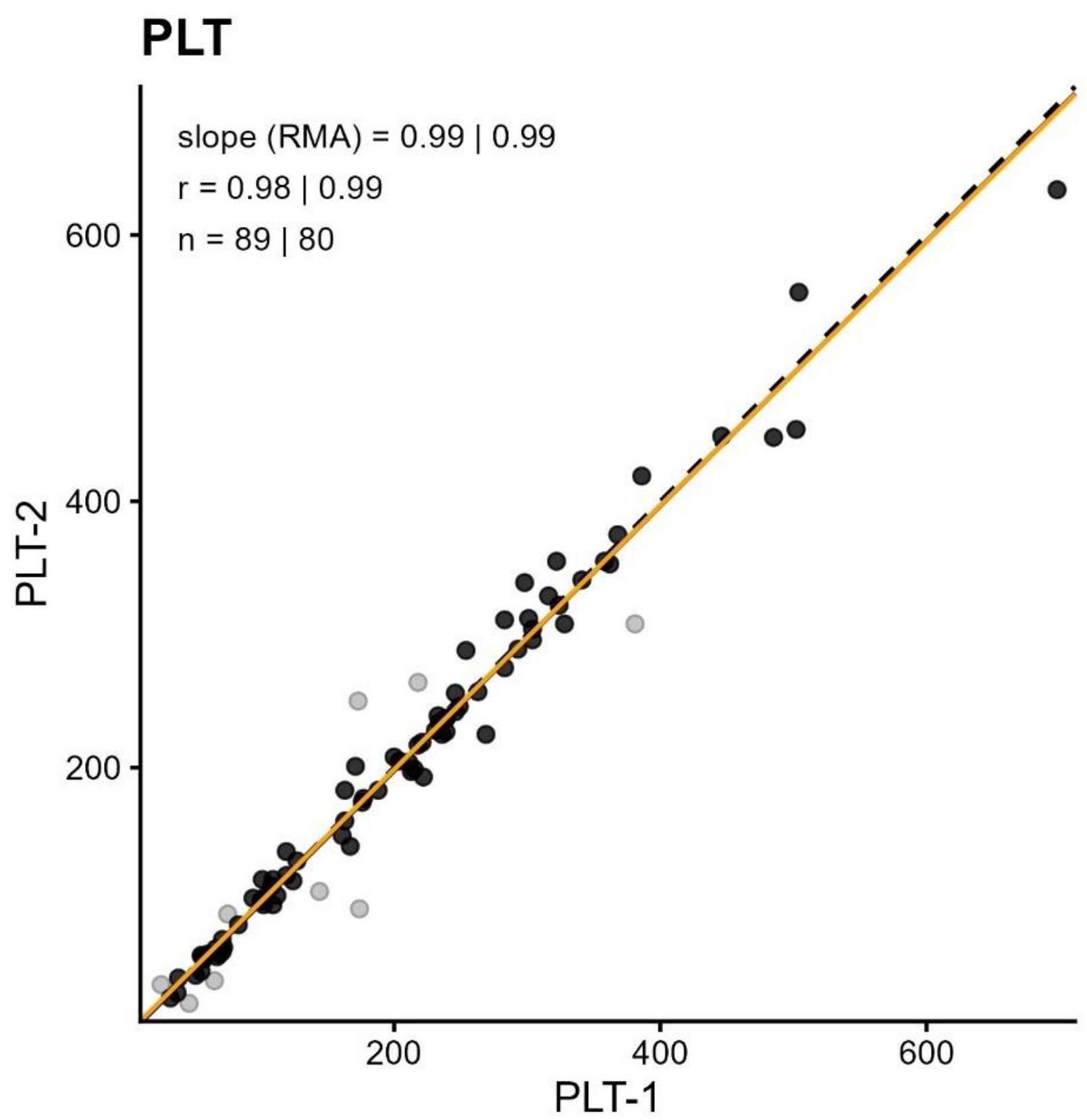
PLT
slope (RMA) = 0.99 | 0.99
r = 0.98 | 0.99
n = 89 | 80
PLT-2
PLT-1
200
400
600

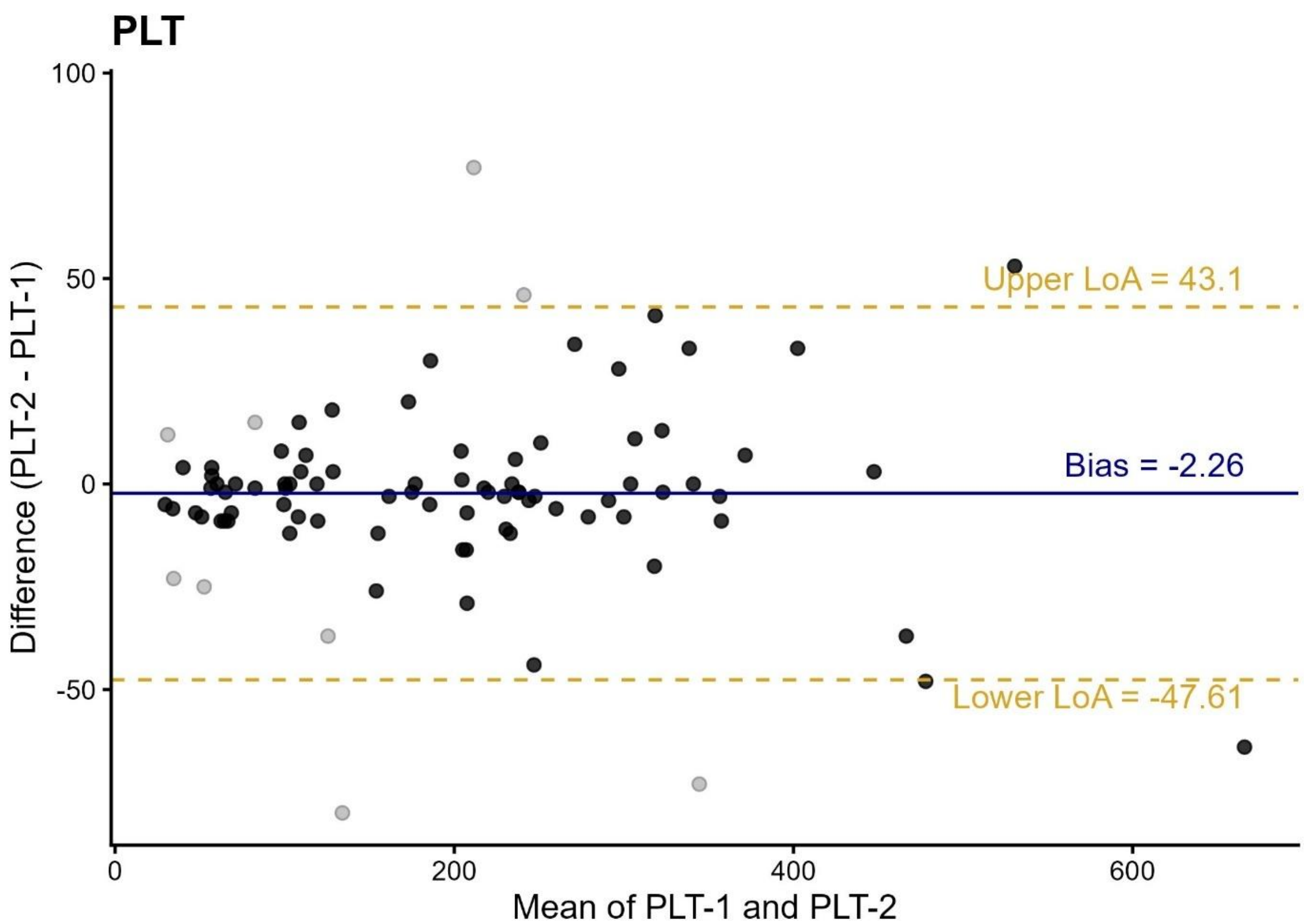
PLT
Upper LoA = 43.1
Bias = -2.26
Lower LoA = -47.61
Difference (PLT-2 - PLT-1)
Mean of PLT-1 and PLT-2
100
50
0
-50
200
400
600

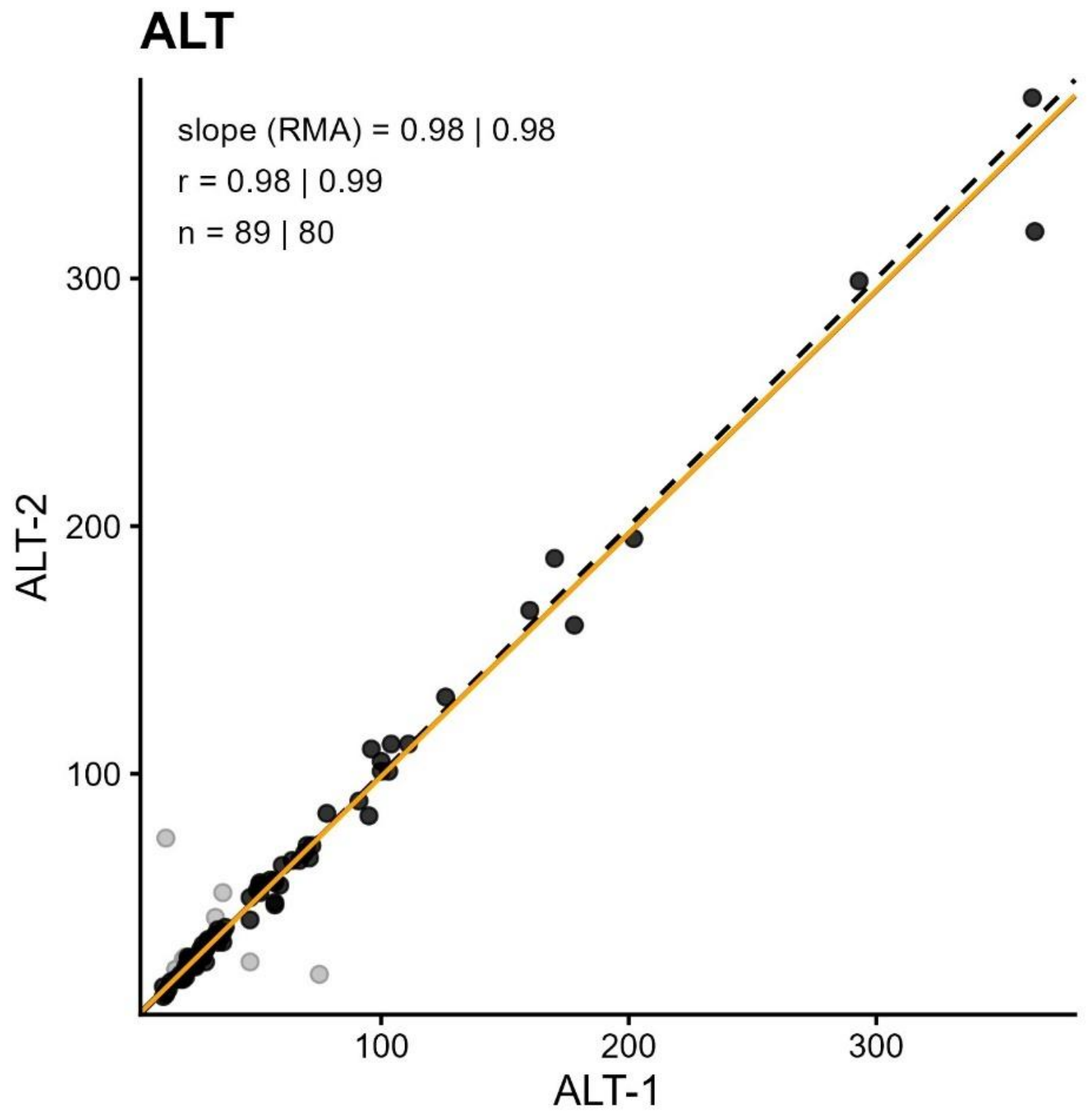
ALT
slope (RMA) = 0.98 | 0.98
r = 0.98 | 0.99
n = 89 | 80
ALT-2
300
200
100
100
200
300
ALT-1

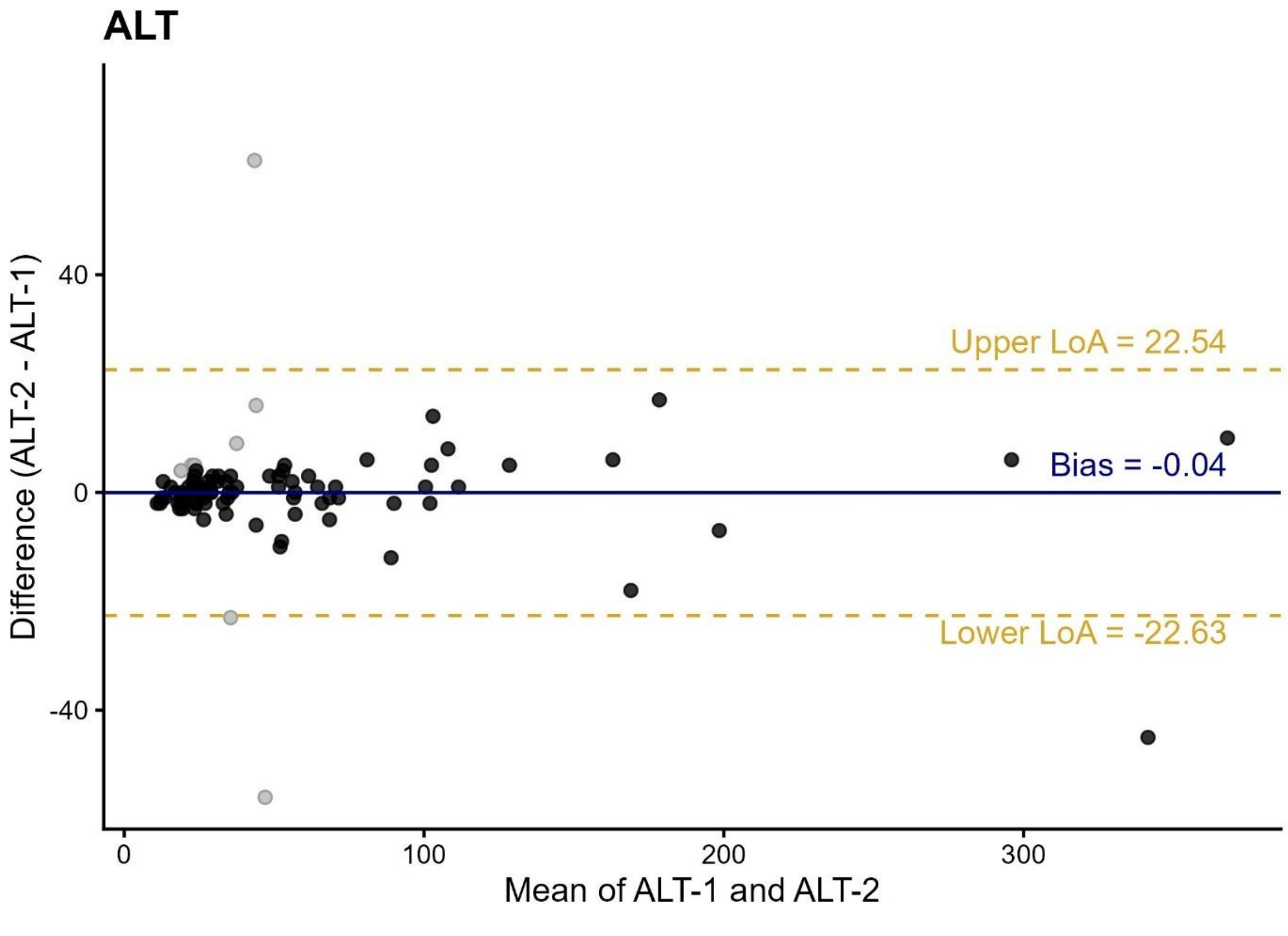
ALT
Difference (ALT-2 - ALT-1)
40
0
-40
Upper LoA = 22.54
Bias = -0.04
Lower LoA = -22.63
0
100
200
300
Mean of ALT-1 and ALT-2

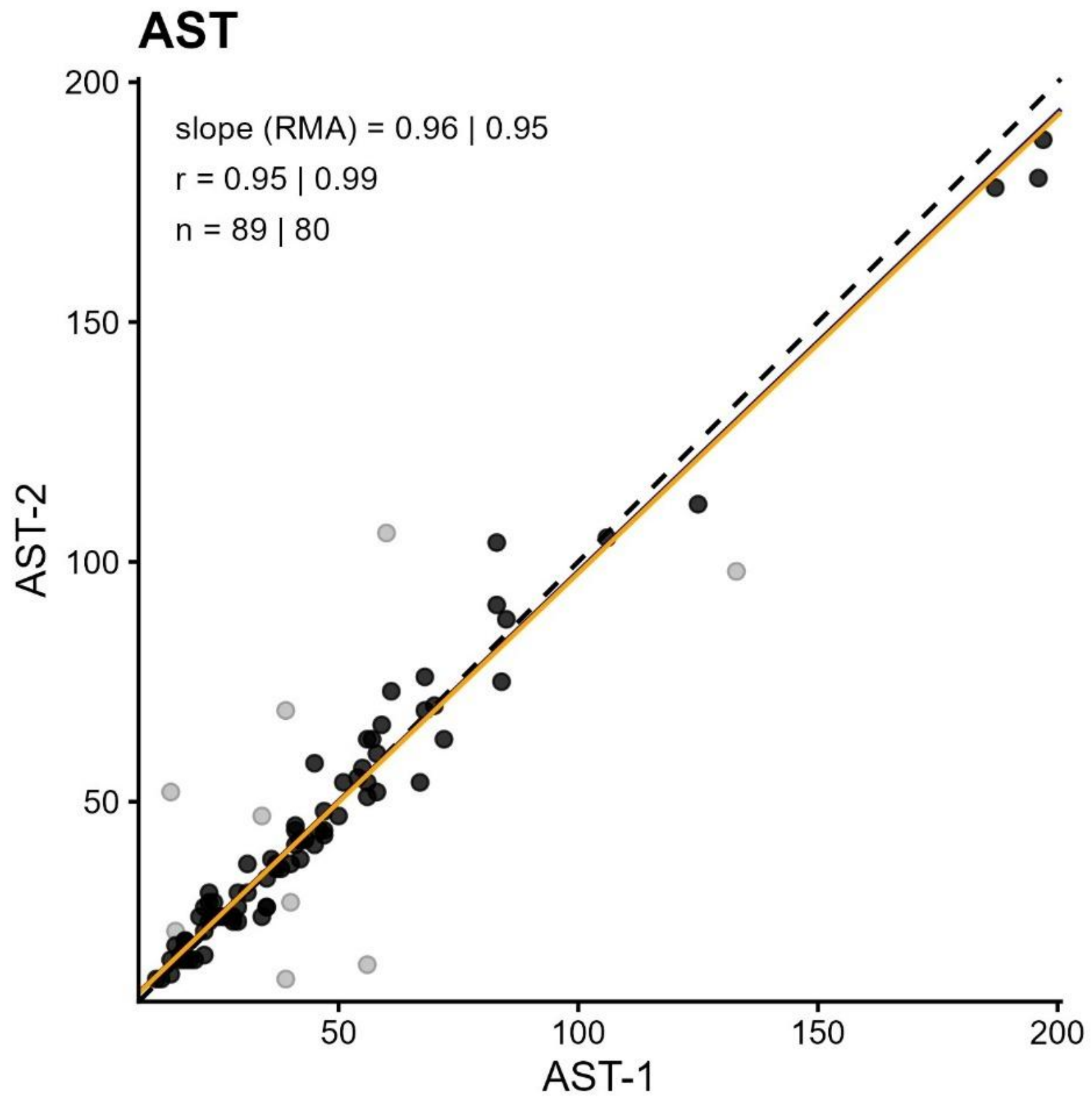
AST
slope (RMA) = 0.96 | 0.95
r = 0.95 | 0.99
n = 89 | 80
AST-2
AST-1
200
150
100
50
50
100
150
200

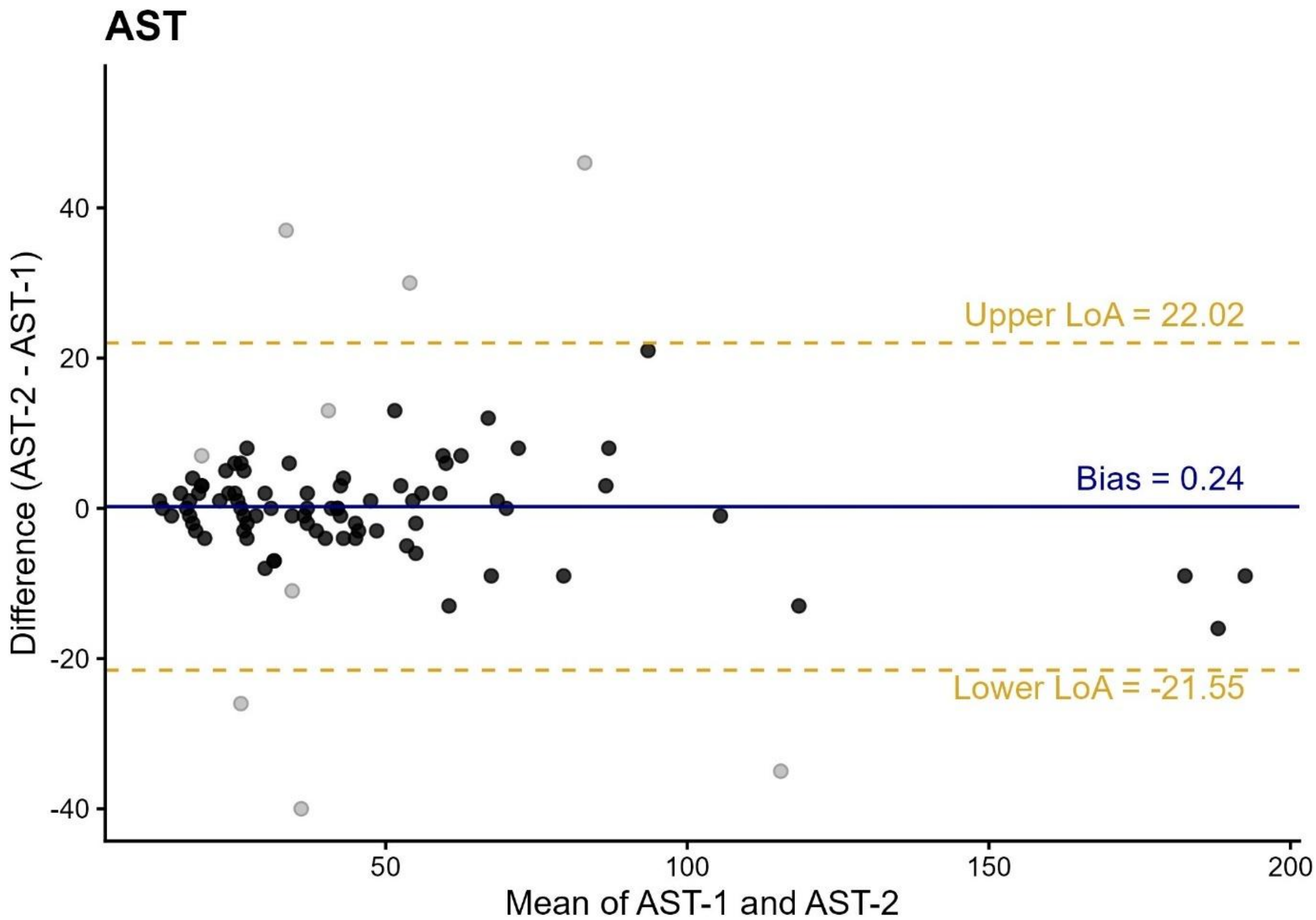
AST
Upper LoA = 22.02
Bias = 0.24
Lower LoA = -21.55
Difference (AST-2 - AST-1)
Mean of AST-1 and AST-2
40
20
0
-20
-40
50
100
150
200

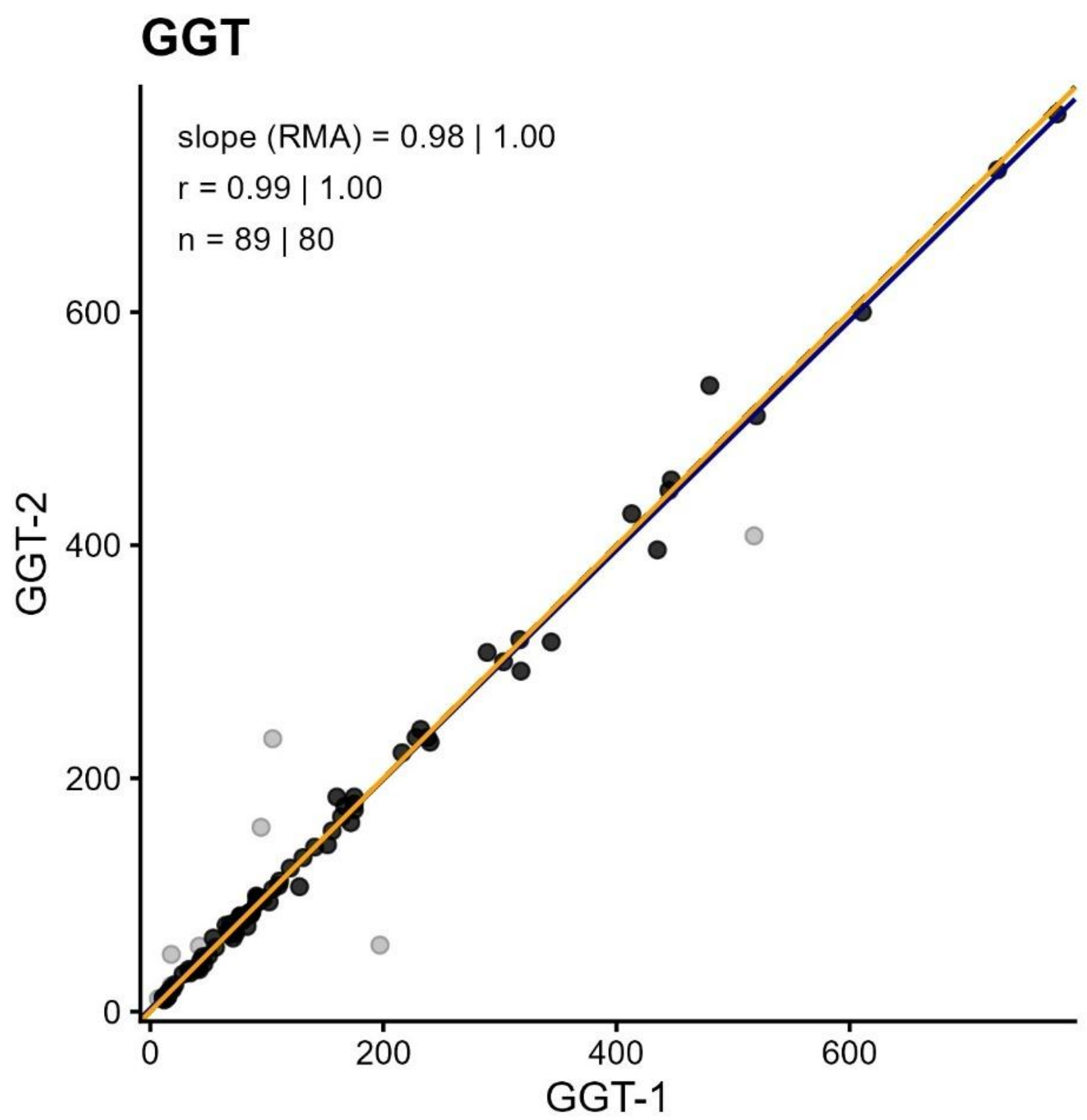
GGT
slope (RMA) = 0.98 | 1.00
r = 0.99 | 1.00
n = 89 | 80
GGT-2
GGT-1
0
200
400
600

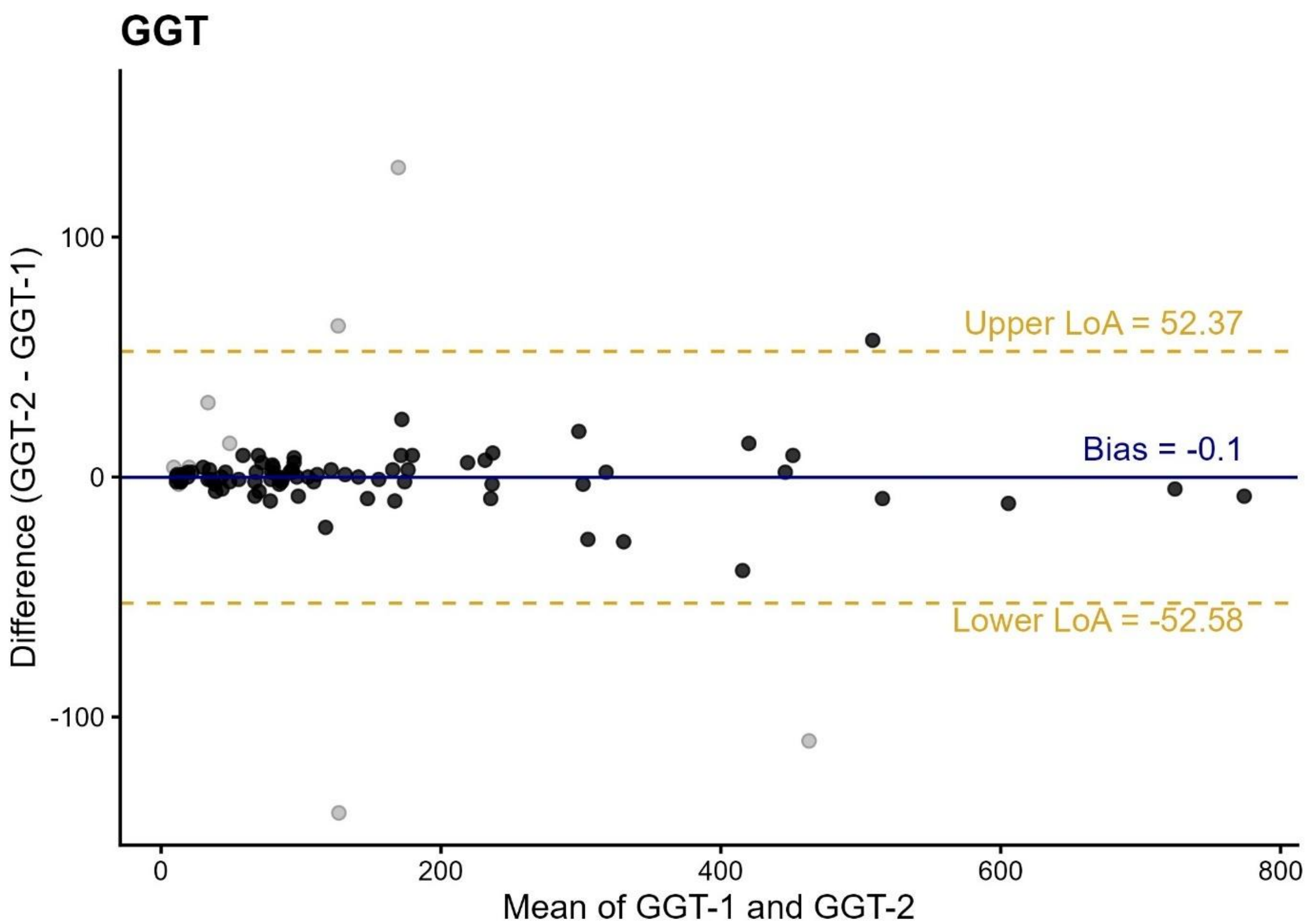
GGT
Upper LoA = 52.37
Bias = -0.1
Lower LoA = -52.58
Difference (GGT-2 - GGT-1)
Mean of GGT-1 and GGT-2
100
0
-100
0
200
400
600
800

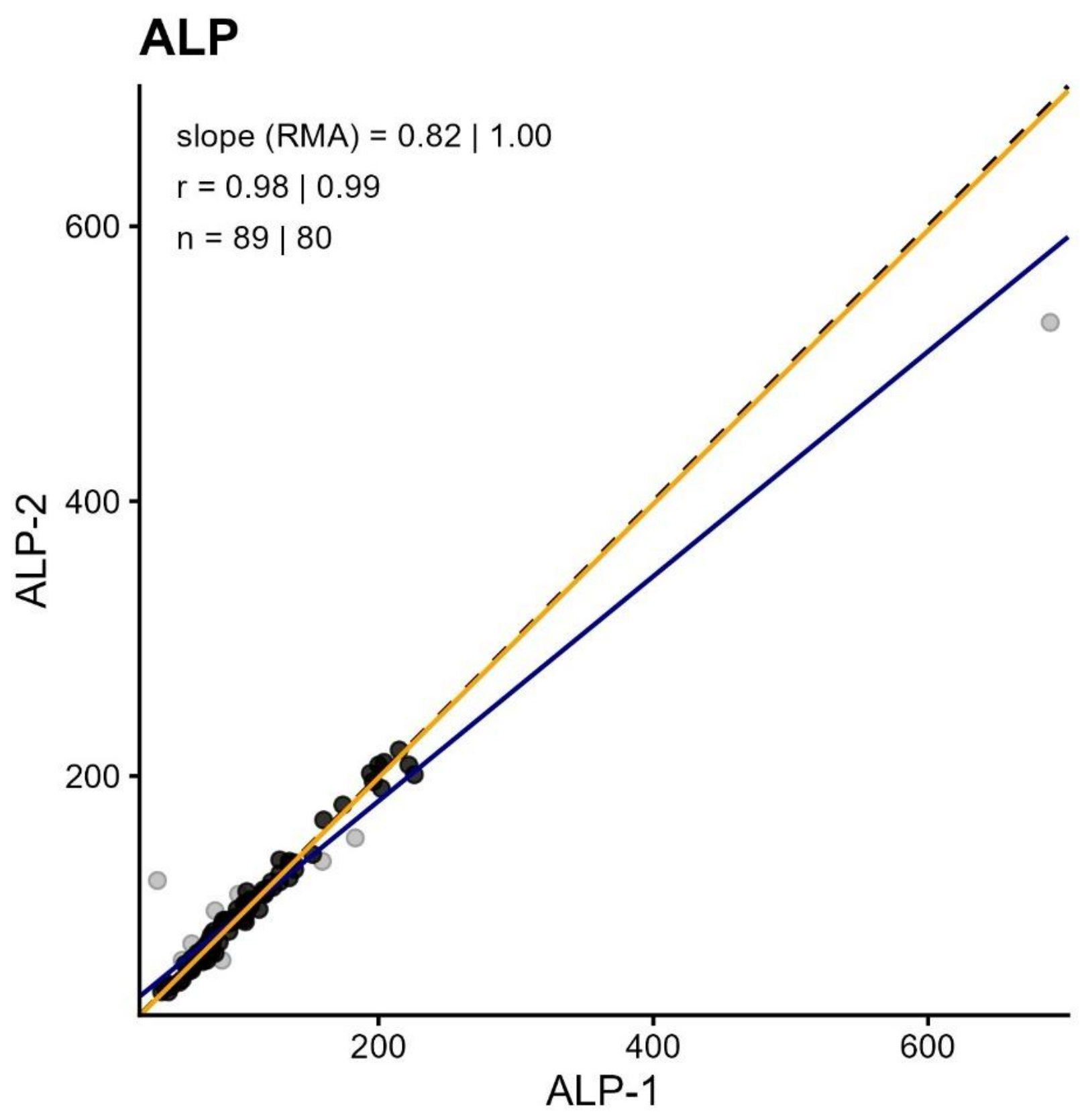

ALP
slope (RMA) = 0.82 | 1.00
r = 0.98 | 0.99
n = 89 | 80
600
400
200
ALP-2
200
400
600
ALP-1

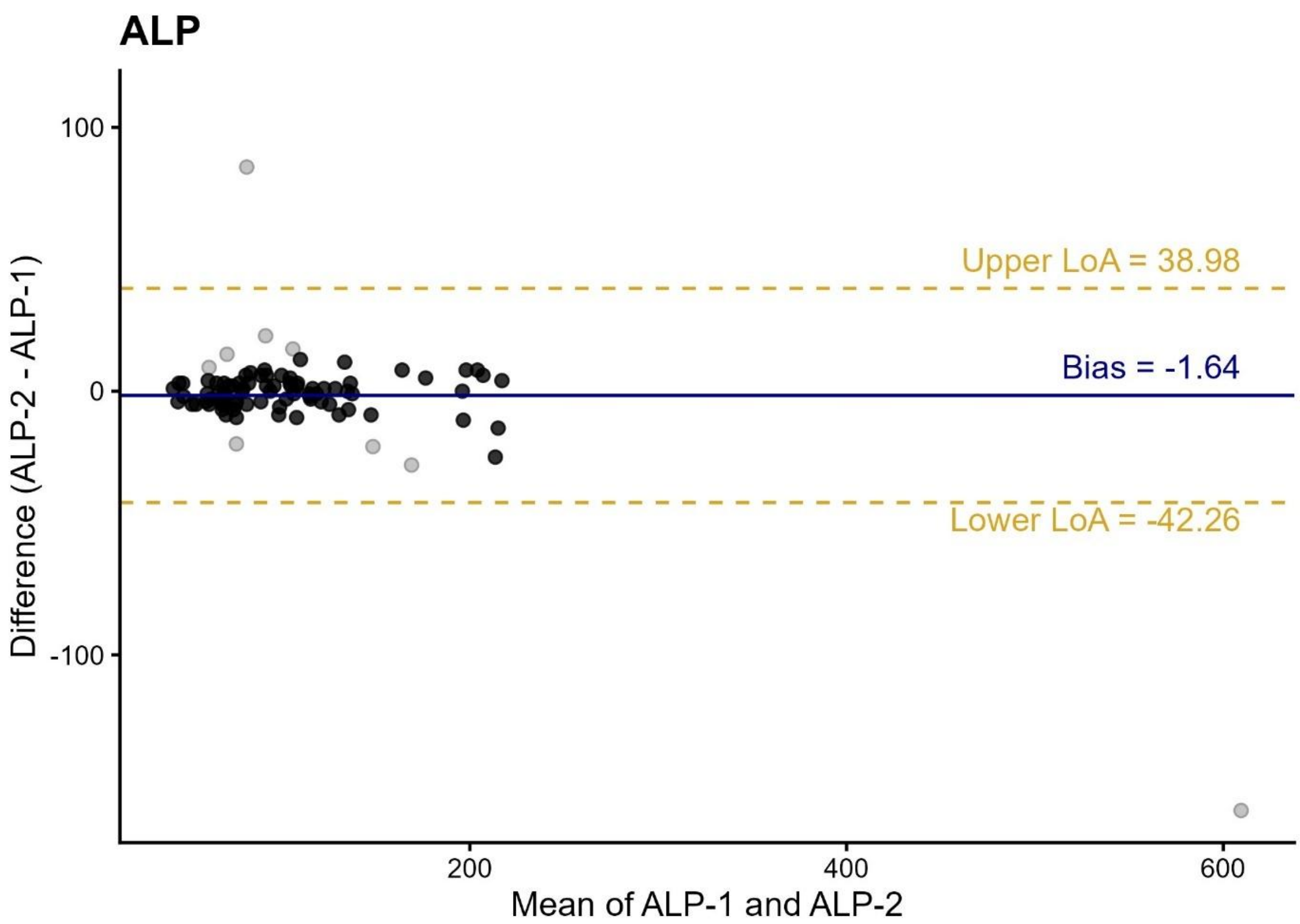

ALP
100
0
-100
Upper LoA = 38.98
Bias = -1.64
Lower LoA = -42.26
Difference (ALP-2 - ALP-1)
200
400
600
Mean of ALP-1 and ALP-2

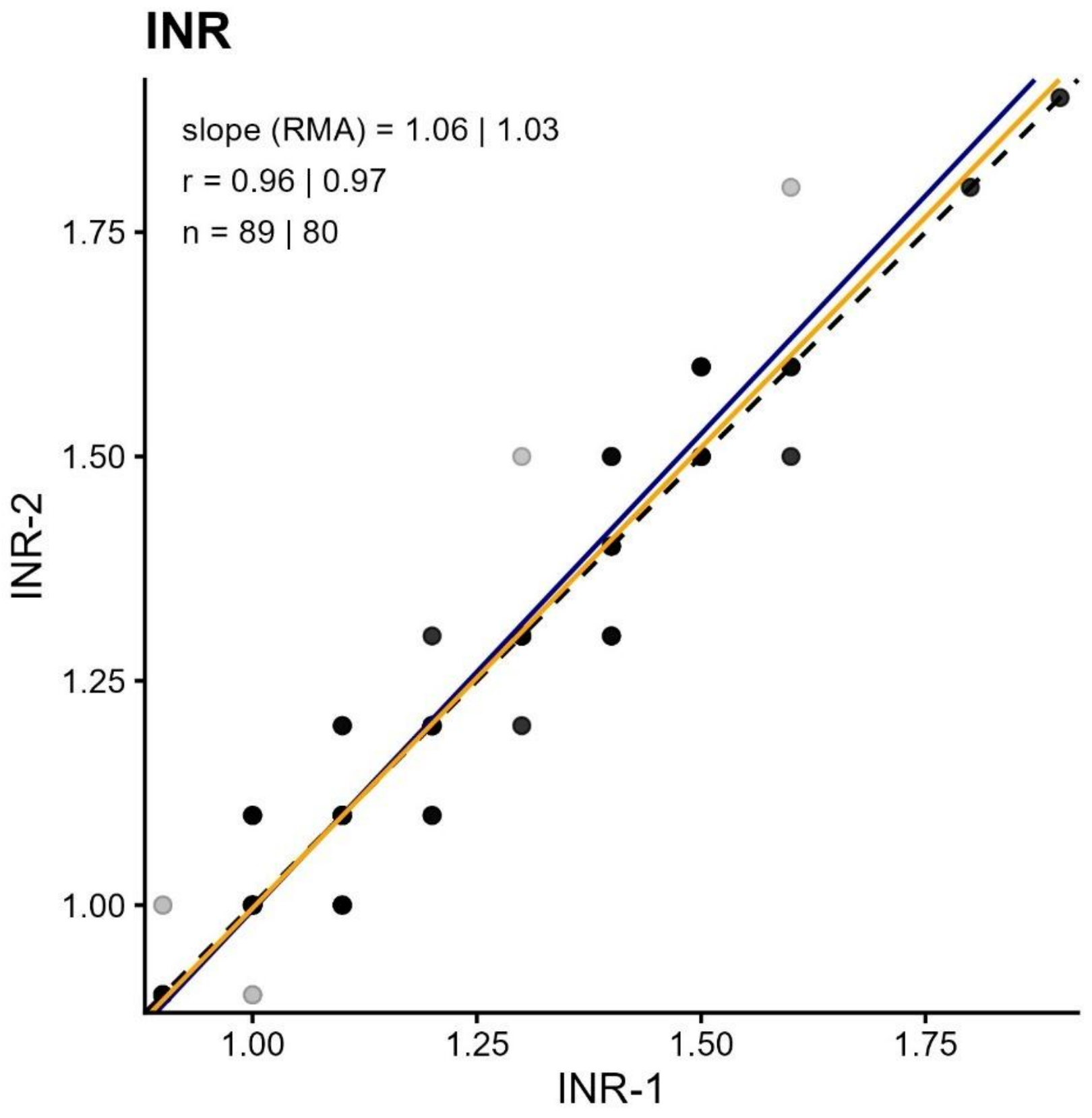
INR
slope (RMA) = 1.06 | 1.03
r = 0.96 | 0.97
n = 89 | 80
1.75
1.50
1.25
1.00
INR-2
1.00
1.25
1.50
1.75
INR-1

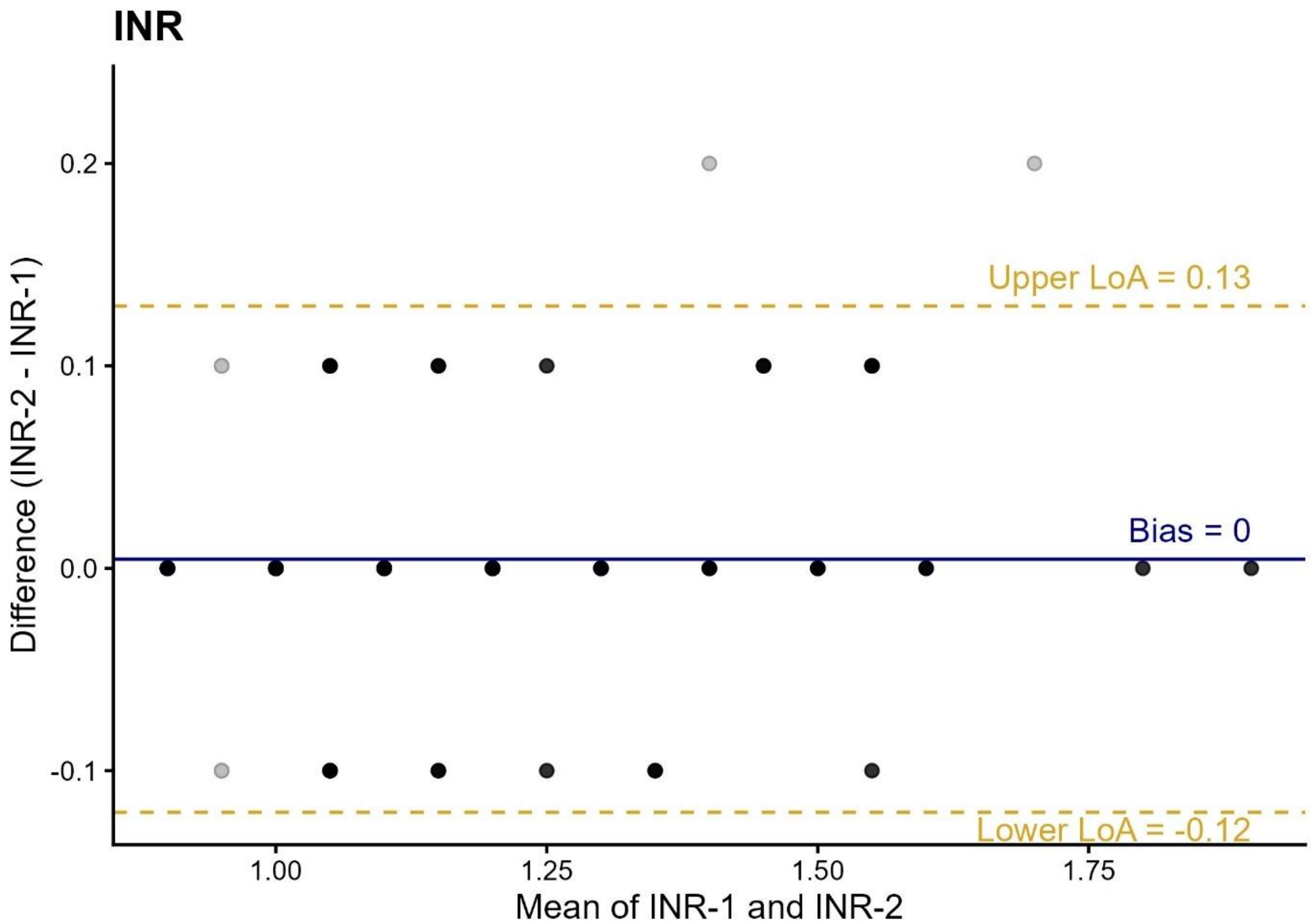
INR
0.2
0.1
0.0
-0.1
Upper LoA = 0.13
Bias = 0
Lower LoA = -0.12
Difference (INR-2 - INR-1)
1.00
1.25
1.50
1.75
Mean of INR-1 and INR-2

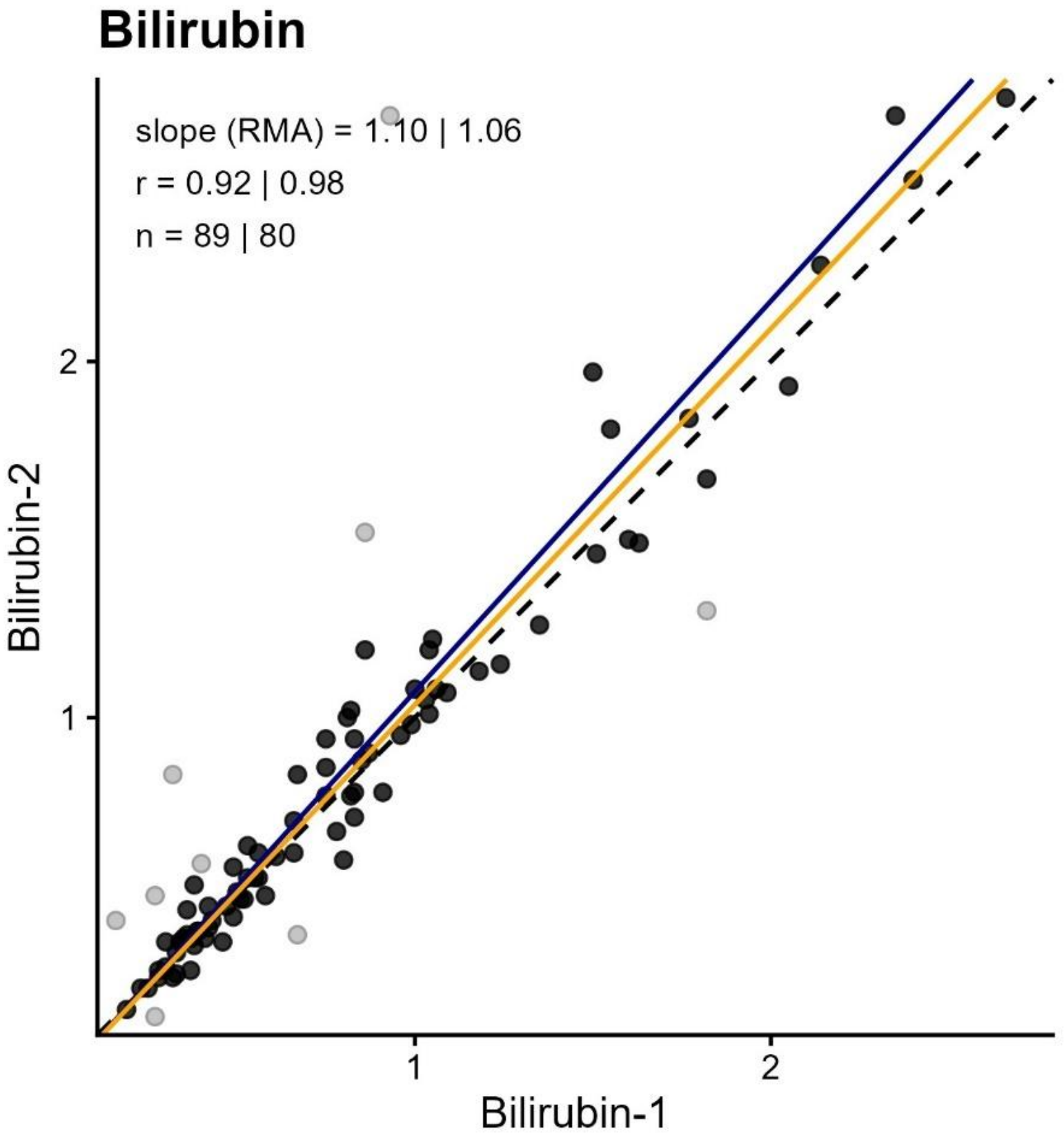
Bilirubin
slope (RMA) = 1.10 | 1.06
r = 0.92 | 0.98
n = 89 | 80
Bilirubin-2
Bilirubin-1
1
2
1
2

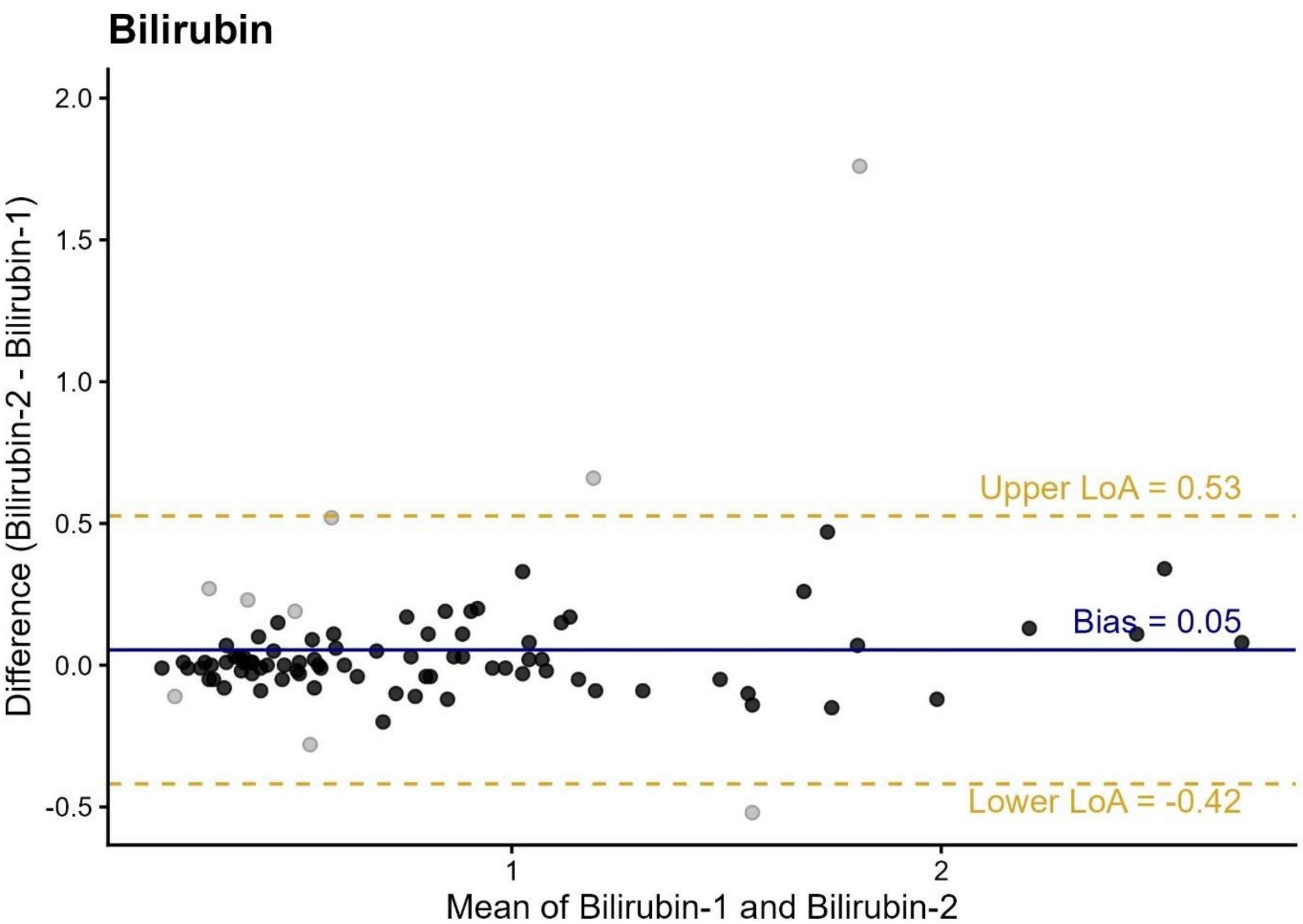
Bilirubin
Difference (Bilirubin-2 - Bilirubin-1)
Mean of Bilirubin-1 and Bilirubin-2
Upper LoA = 0.53
Bias = 0.05
Lower LoA = -0.42
2.0
1.5
1.0
0.5
0.0
-0.5
1
2

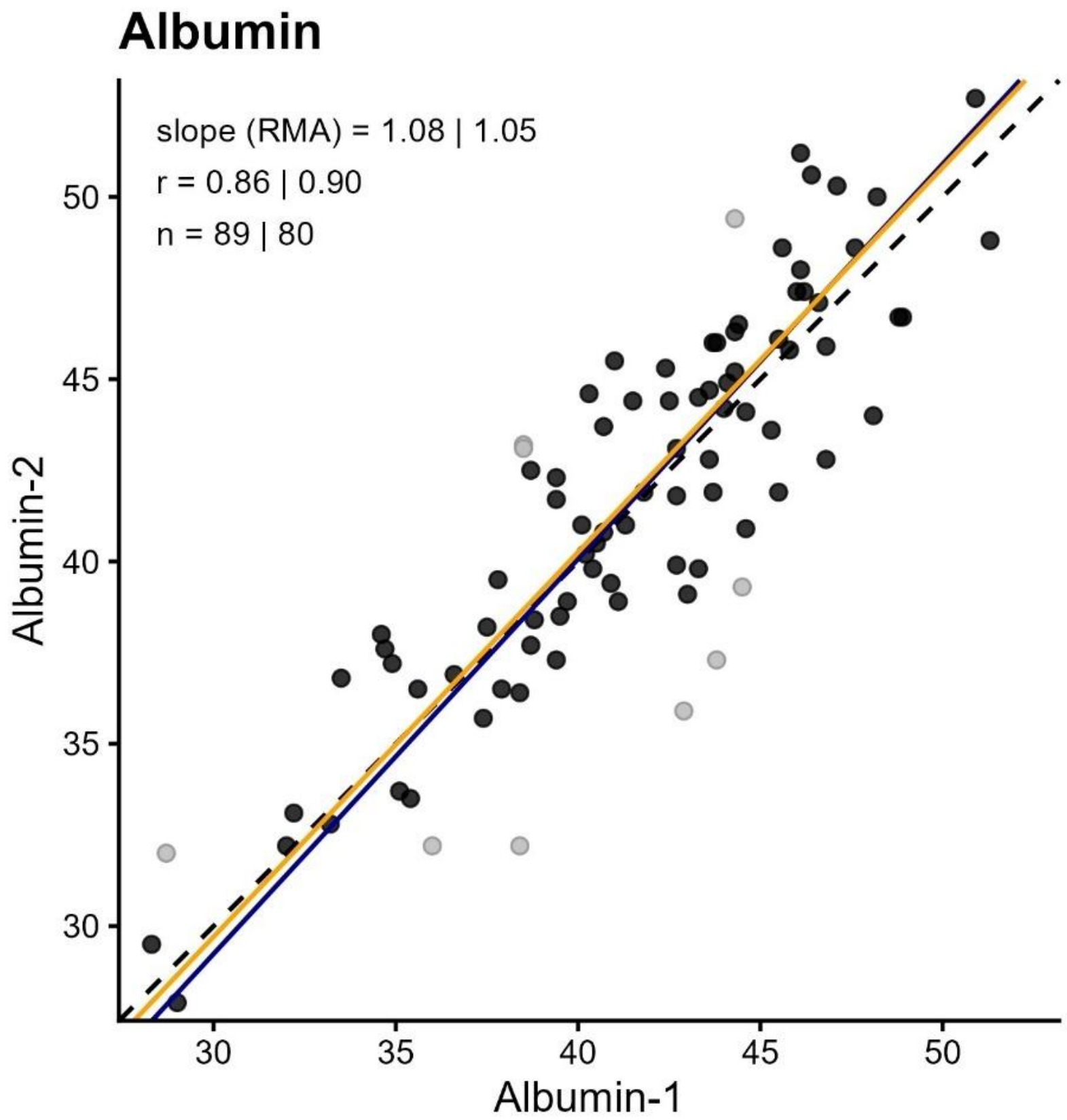

Albumin
slope (RMA) = 1.08 | 1.05
r = 0.86 | 0.90
n = 89 | 80
Albumin-2
Albumin-1
30
35
40
45
50

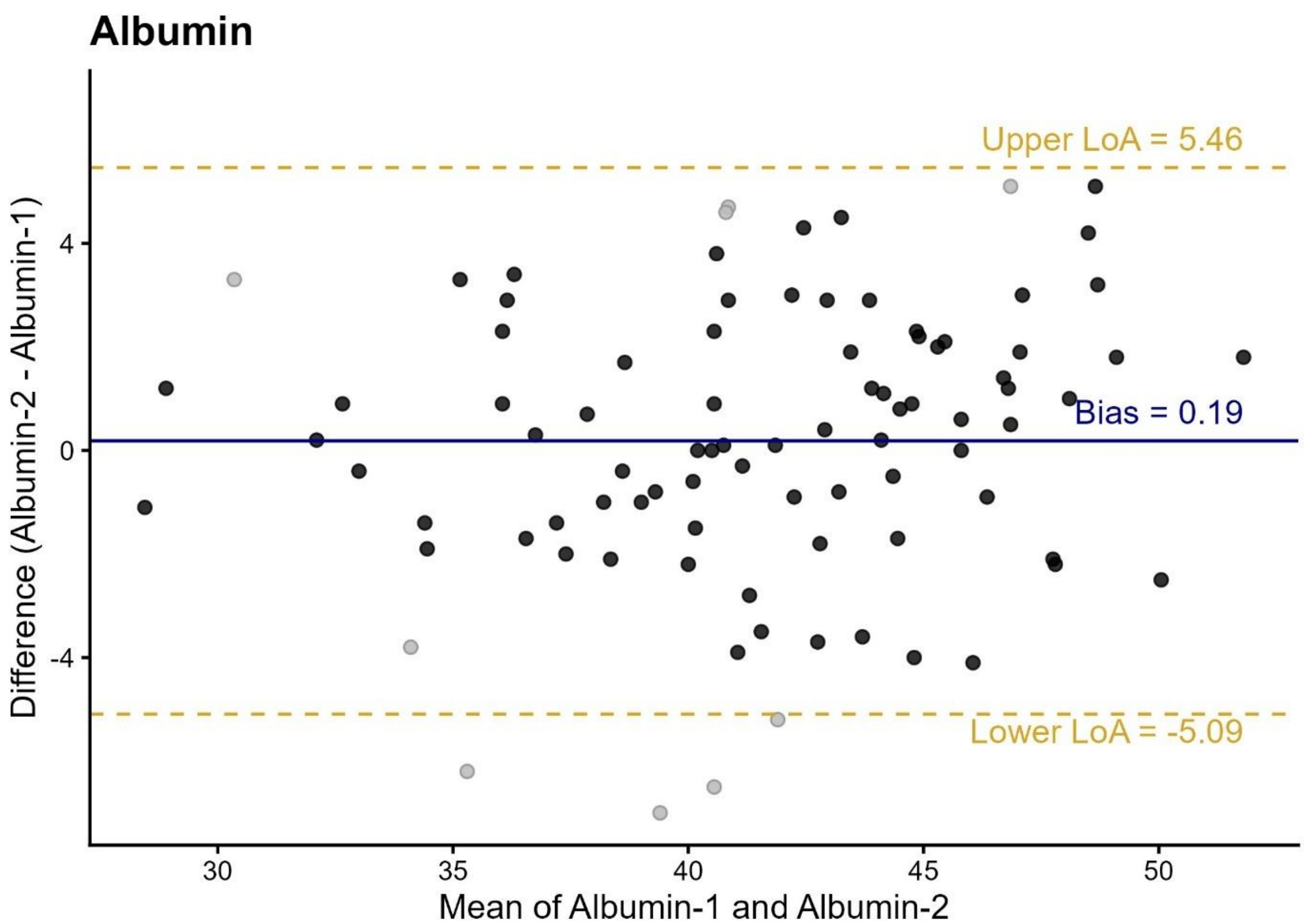

Albumin
Upper LoA = 5.46
Bias = 0.19
Lower LoA = -5.09
Difference (Albumin-2 - Albumin-1)
Mean of Albumin-1 and Albumin-2
4
0
-4
30
35
40
45
50

Reproducibility cohort I - suspected MASLD

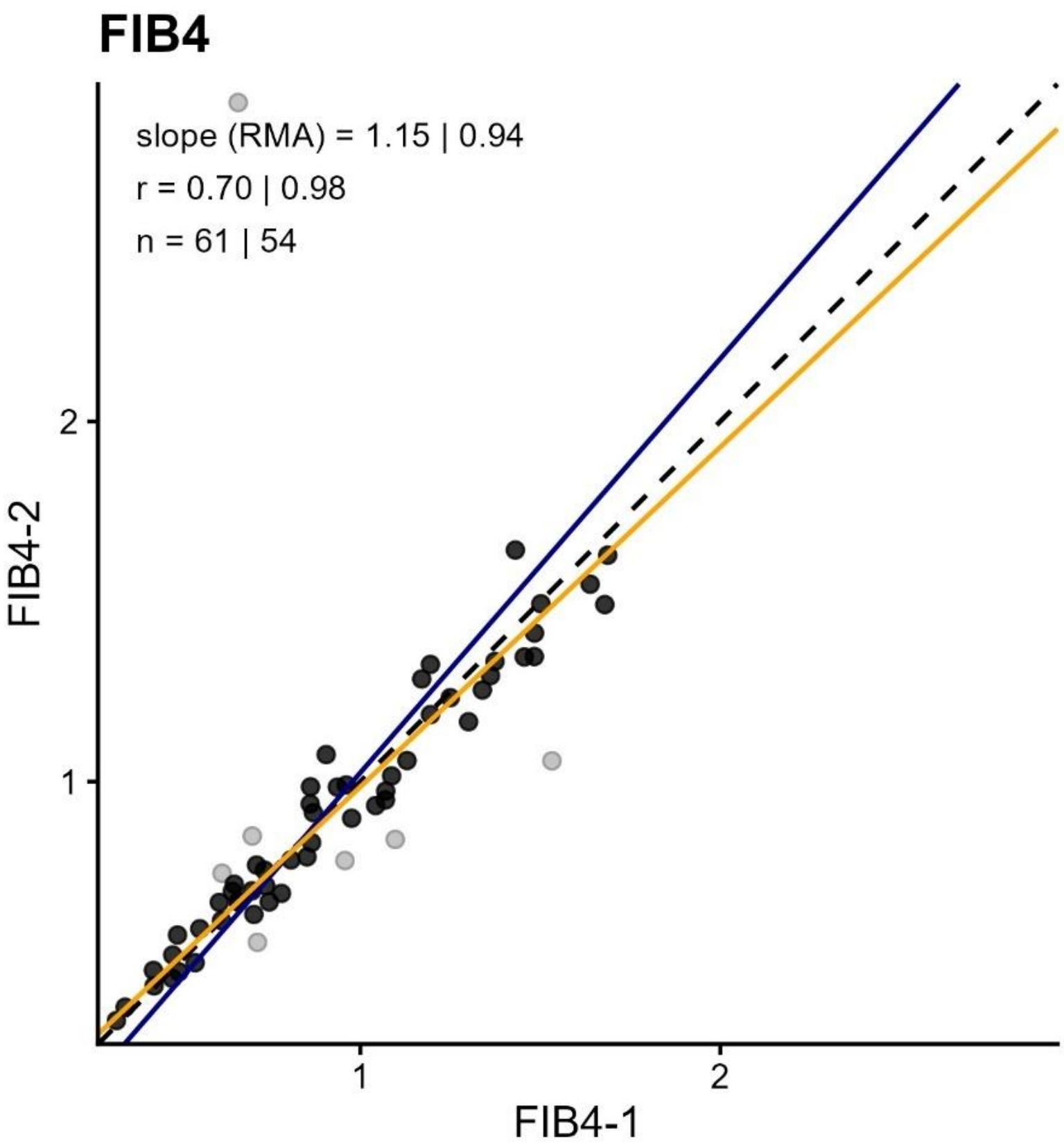

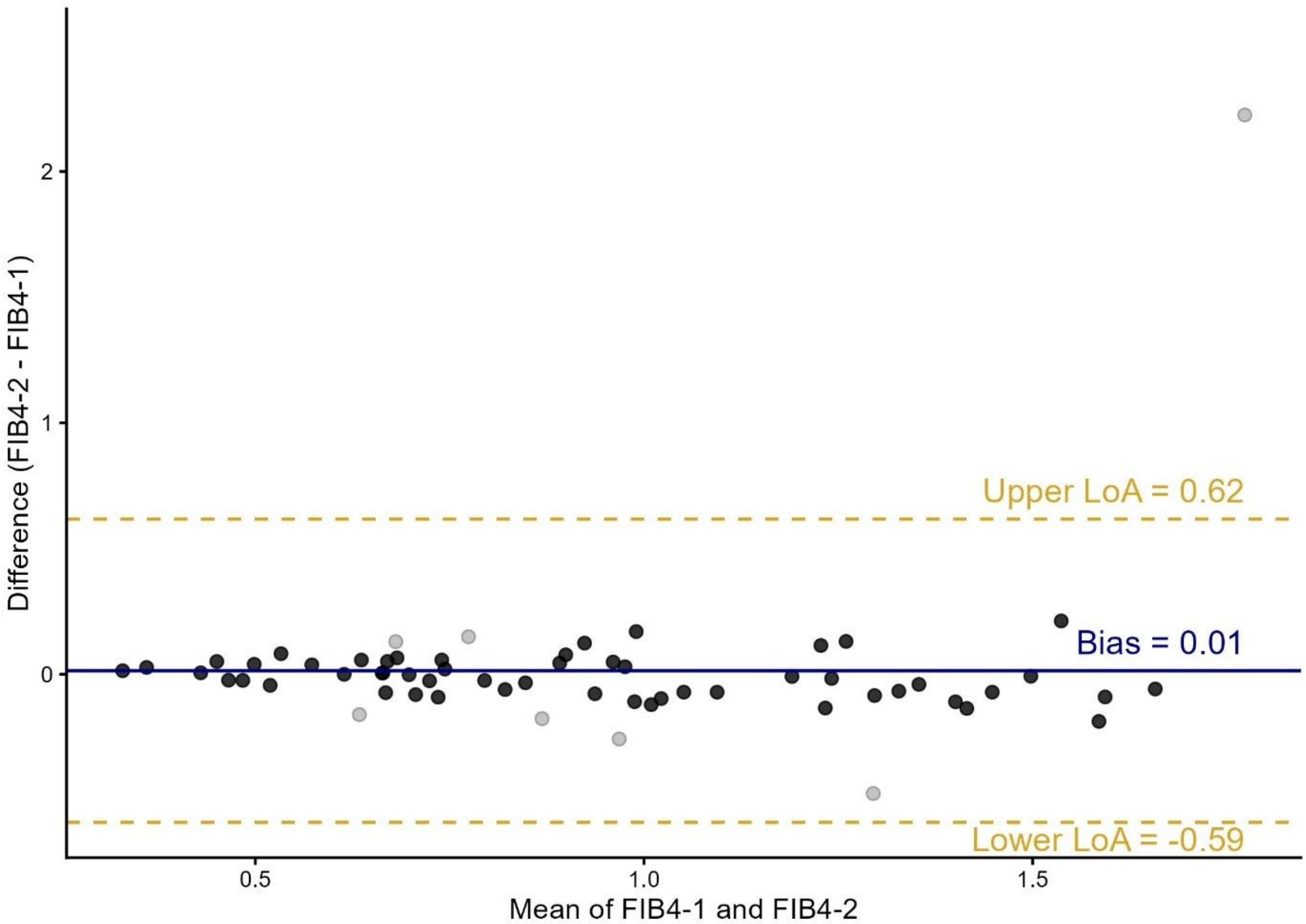

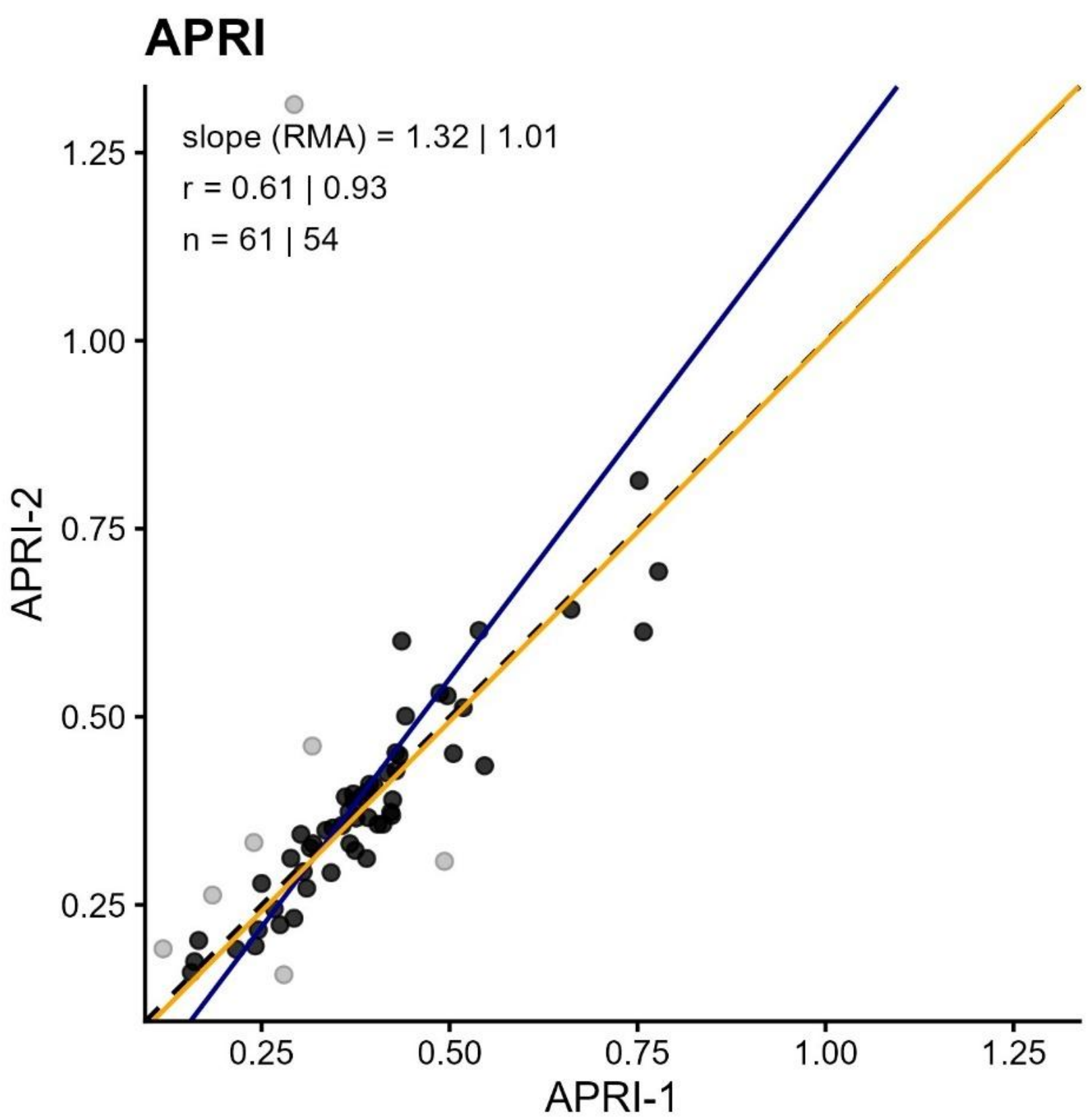
APRI
slope (RMA) = 1.32 | 1.01
r = 0.61 | 0.93
n = 61 | 54
APRI-2
APRI-1
0.25
0.50
0.75
1.00
1.25

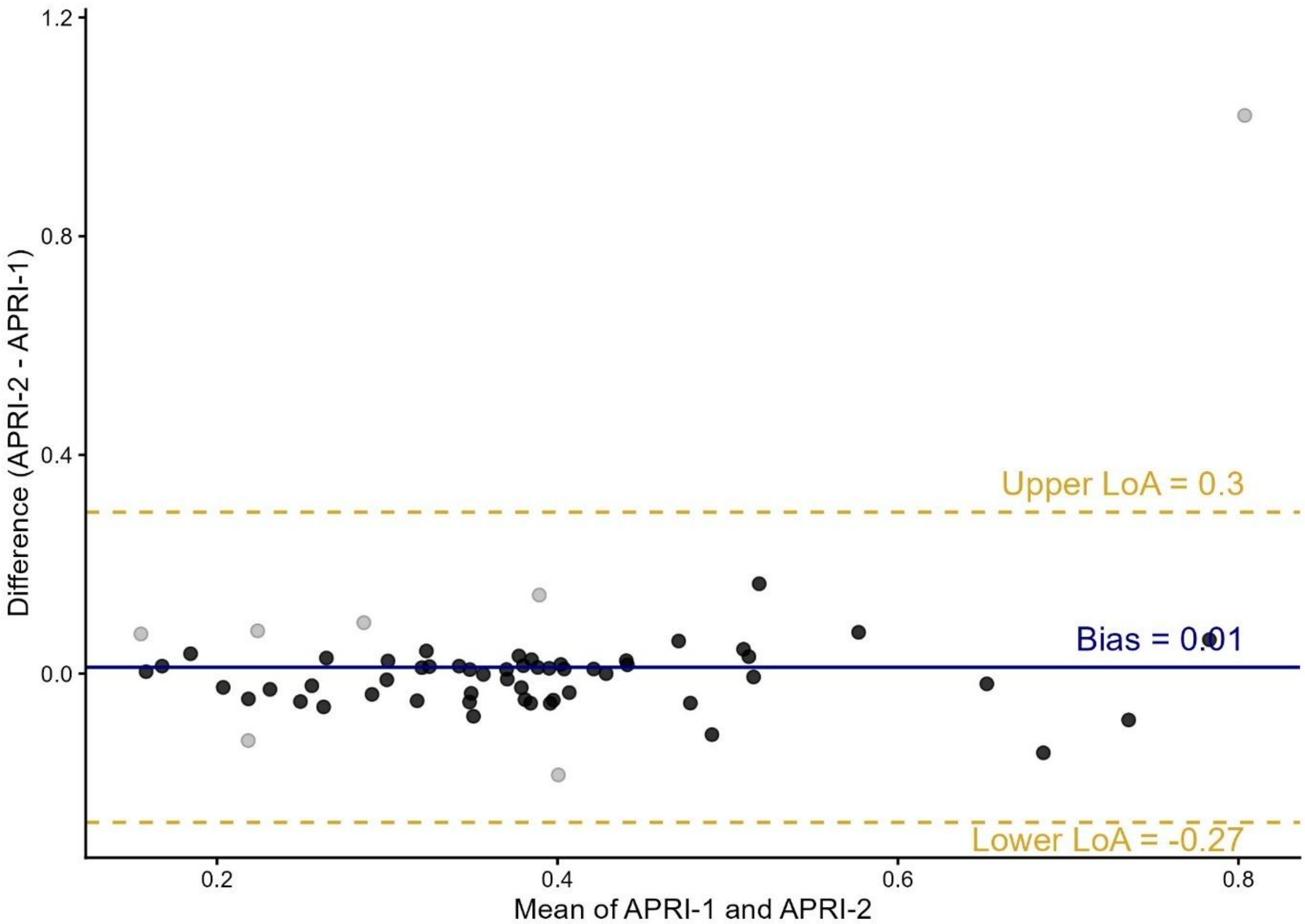
Difference (APRI-2 - APRI-1)
Mean of APRI-1 and APRI-2
Upper LoA = 0.3
Bias = 0.01
Lower LoA = -0.27
0.0
0.4
0.8
1.2
0.2
0.4
0.6
0.8

LiverPRO-F2

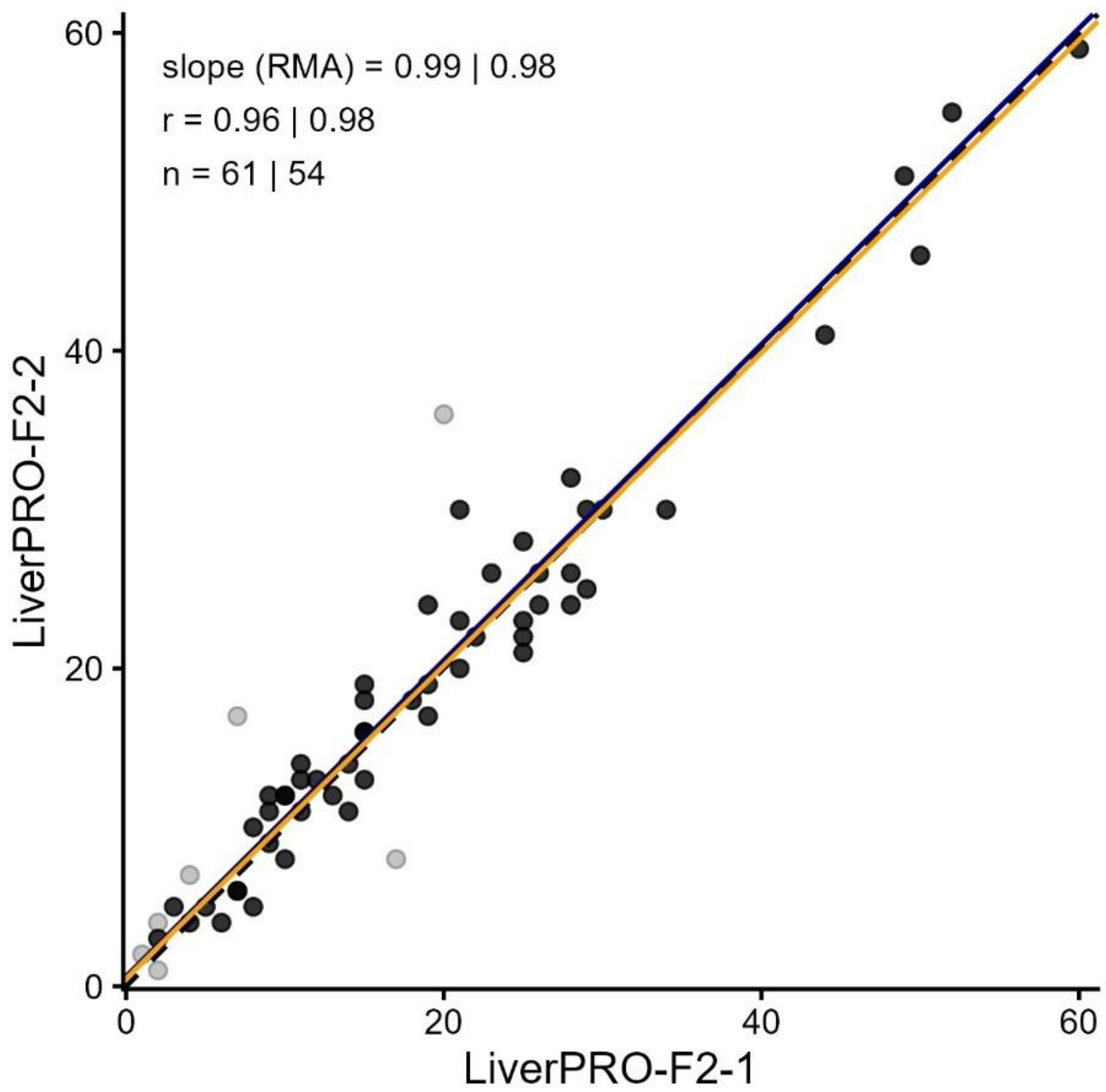
slope (RMA) = 0.99 | 0.98
r = 0.96 | 0.98
n = 61 | 54
LiverPRO-F2-2
LiverPRO-F2-1
0
20
40
60

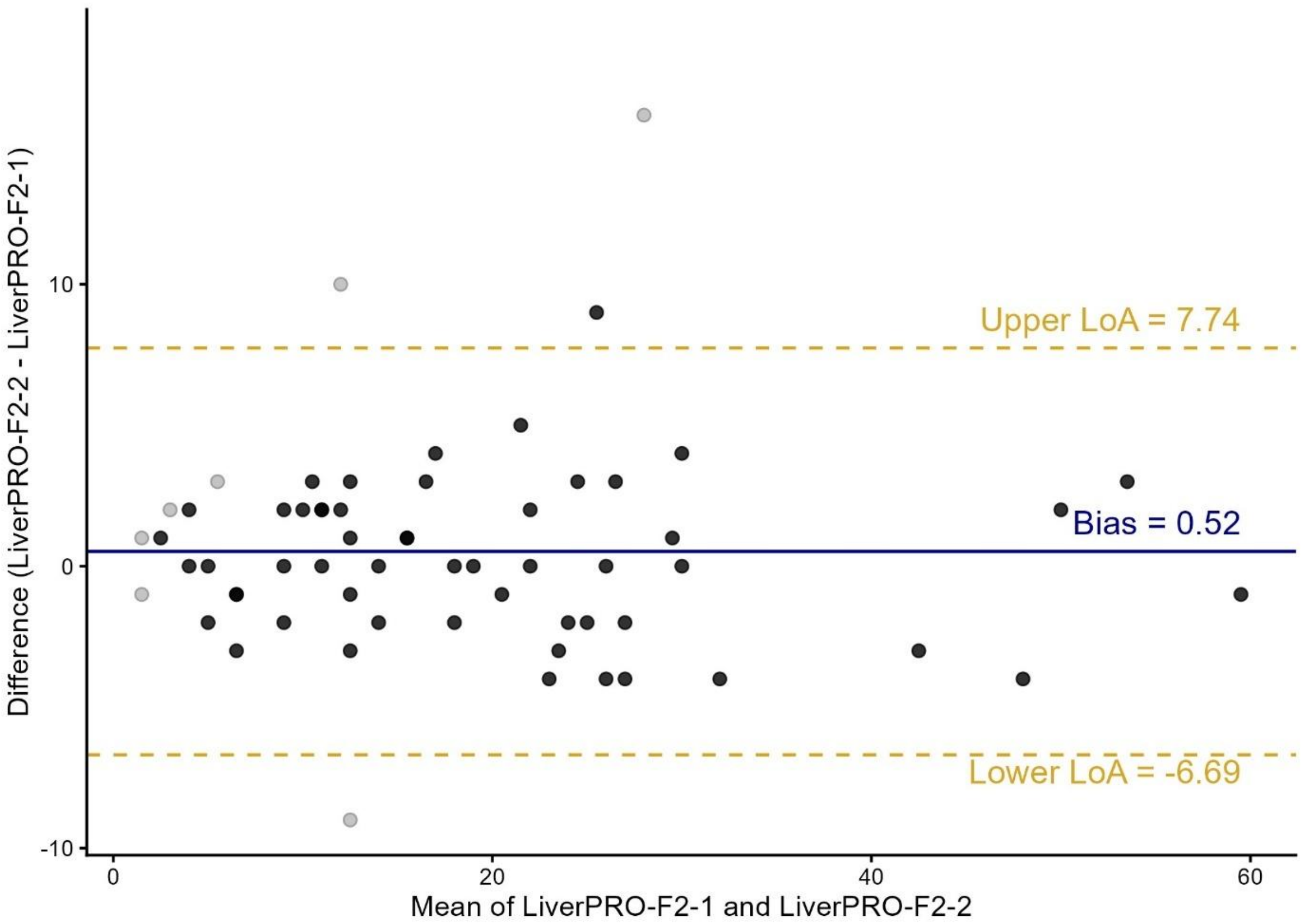
Upper LoA = 7.74
Bias = 0.52
Lower LoA = -6.69
Difference (LiverPRO-F2-2 - LiverPRO-F2-1)
Mean of LiverPRO-F2-1 and LiverPRO-F2-2
10
0
-10
0
20
40
60

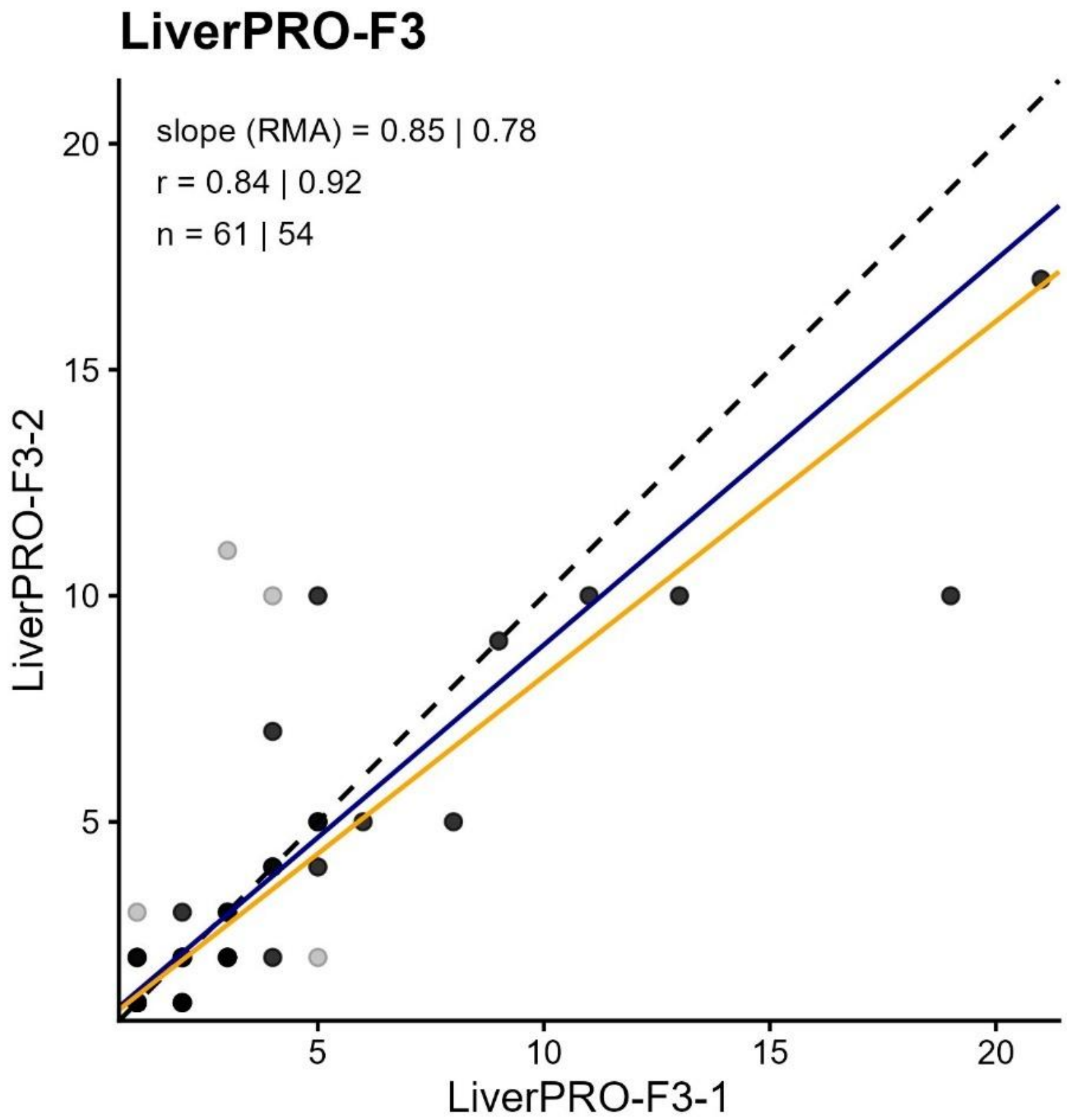
LiverPRO-F3
slope (RMA) = 0.85 | 0.78
r = 0.84 | 0.92
n = 61 | 54
LiverPRO-F3-2
LiverPRO-F3-1
20
15
10
5

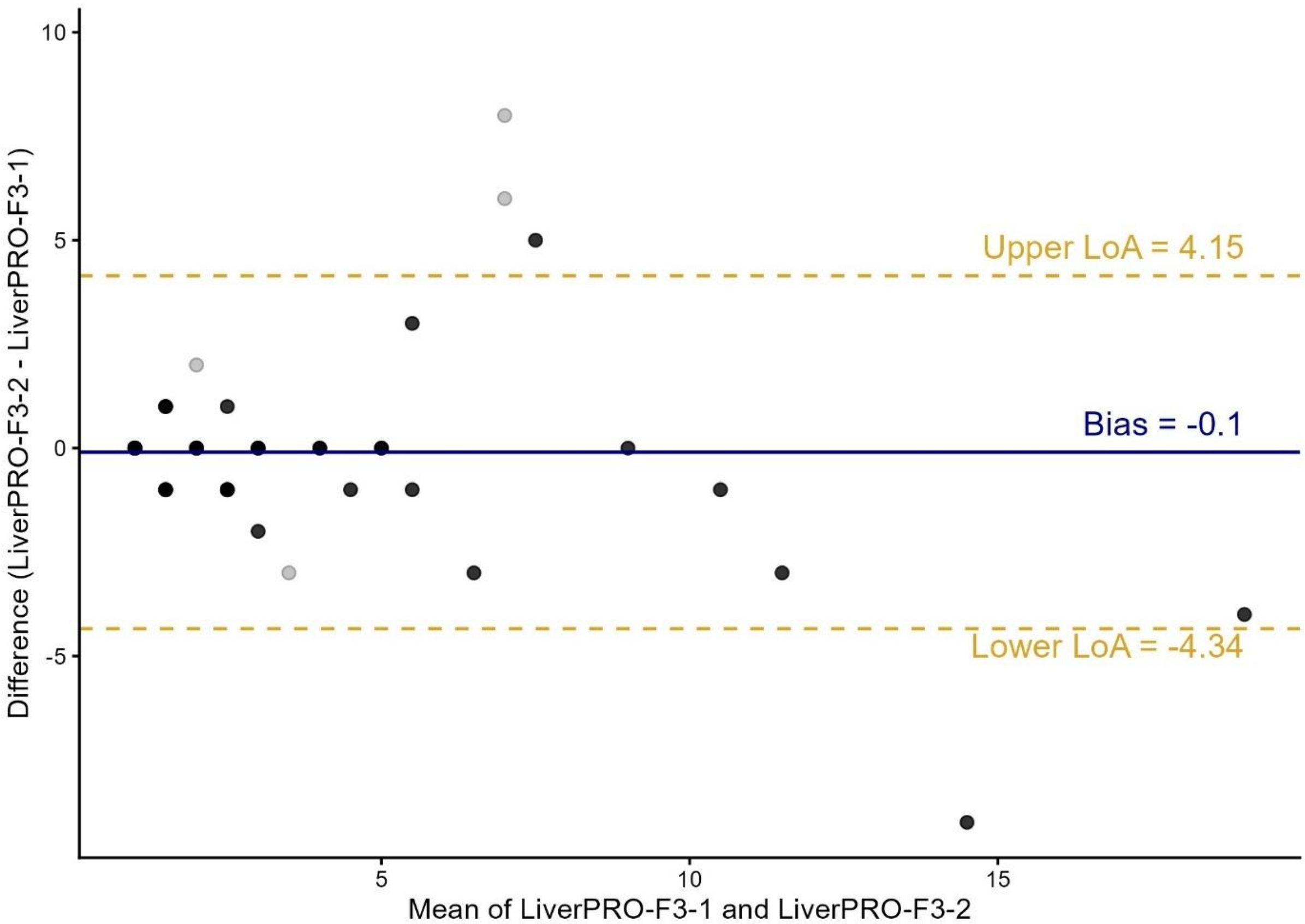
Upper LoA = 4.15
Bias = -0.1
Lower LoA = -4.34
Difference (LiverPRO-F3-2 - LiverPRO-F3-1)
Mean of LiverPRO-F3-1 and LiverPRO-F3-2
10
5
0
-5
15

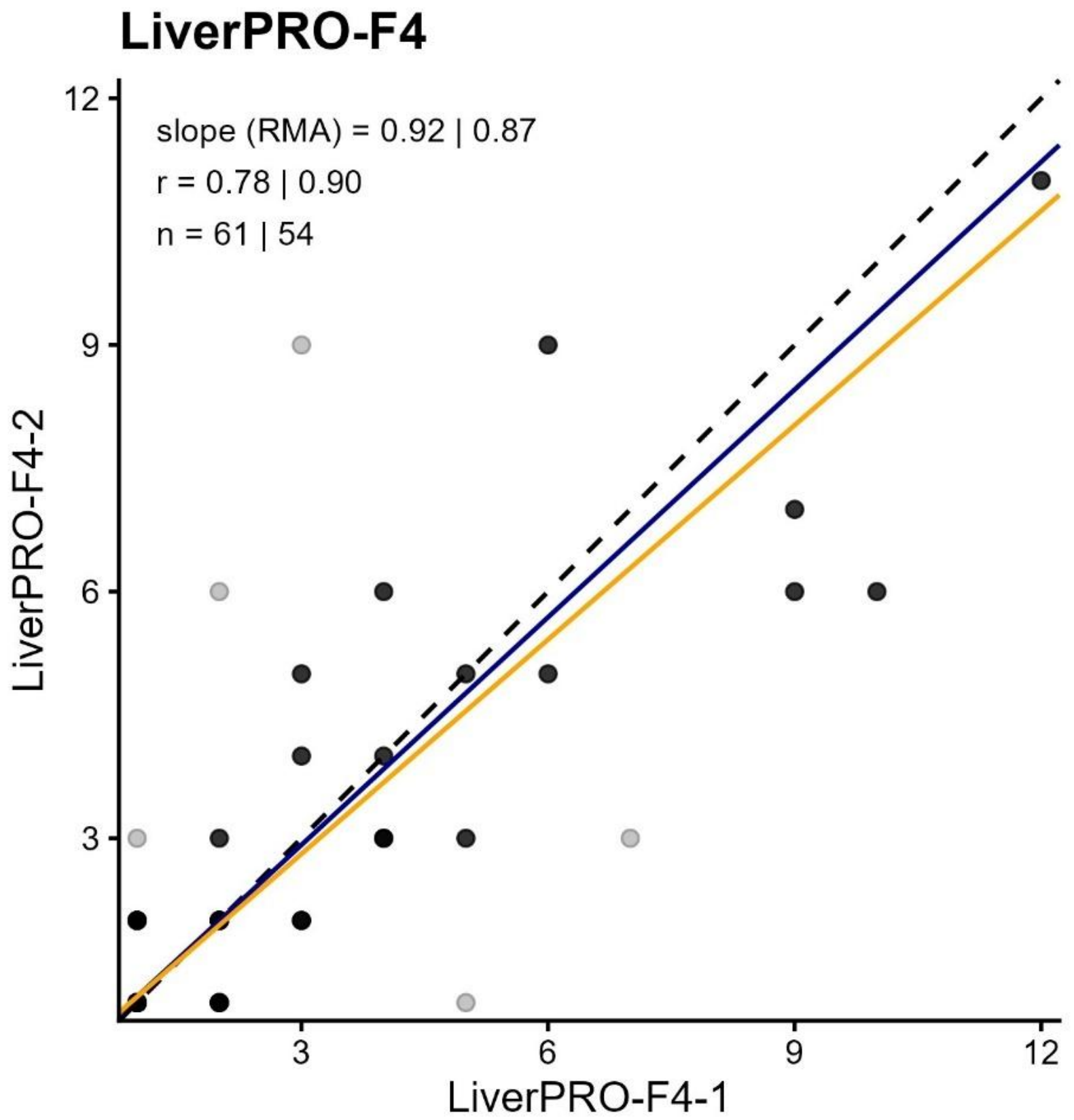

LiverPRO-F4
slope (RMA) = 0.92 | 0.87
r = 0.78 | 0.90
n = 61 | 54
LiverPRO-F4-2
LiverPRO-F4-1

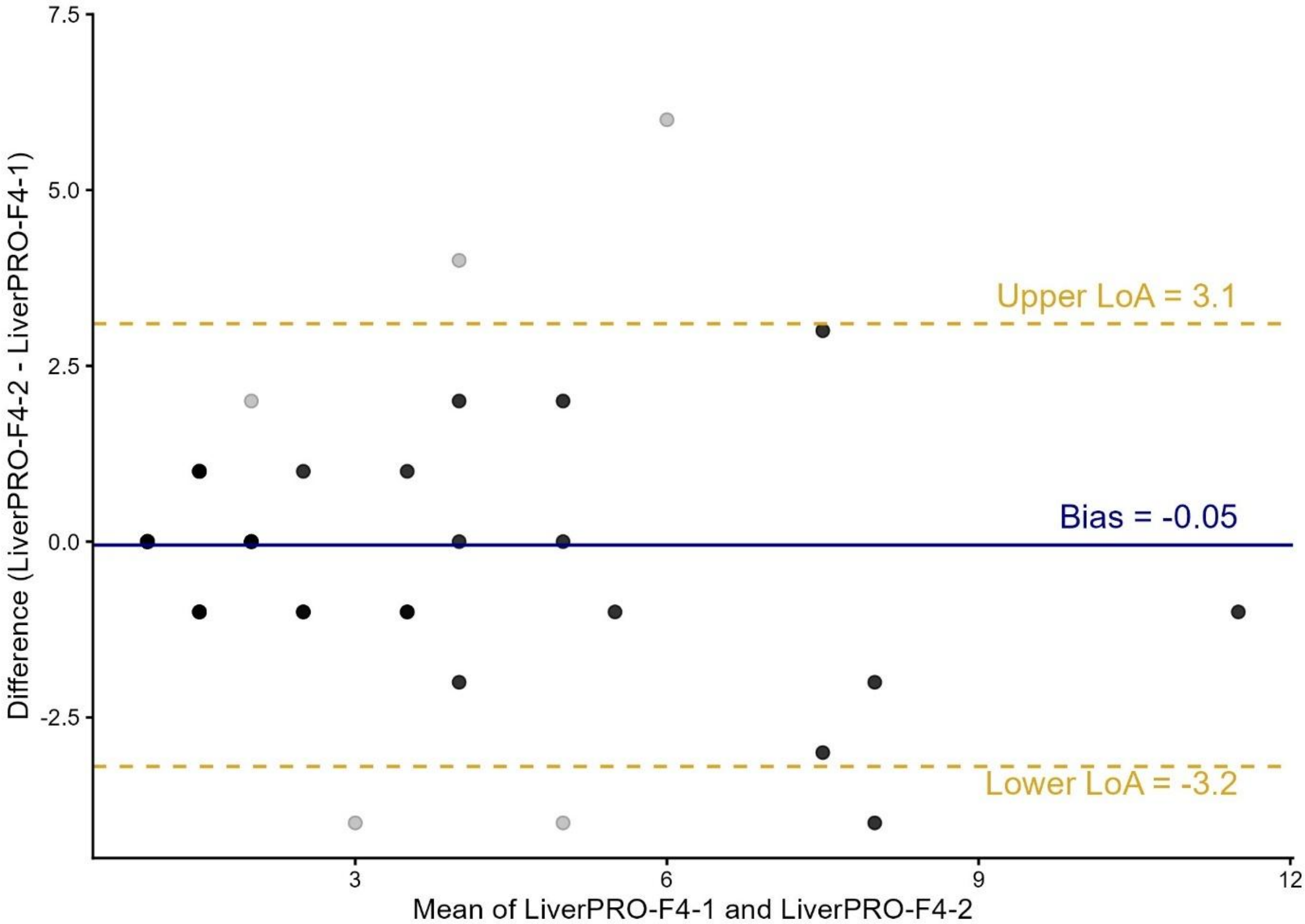

Upper LoA = 3.1
Bias = -0.05
Lower LoA = -3.2
Difference (LiverPRO-F4-2 - LiverPRO-F4-1)
Mean of LiverPRO-F4-1 and LiverPRO-F4-2

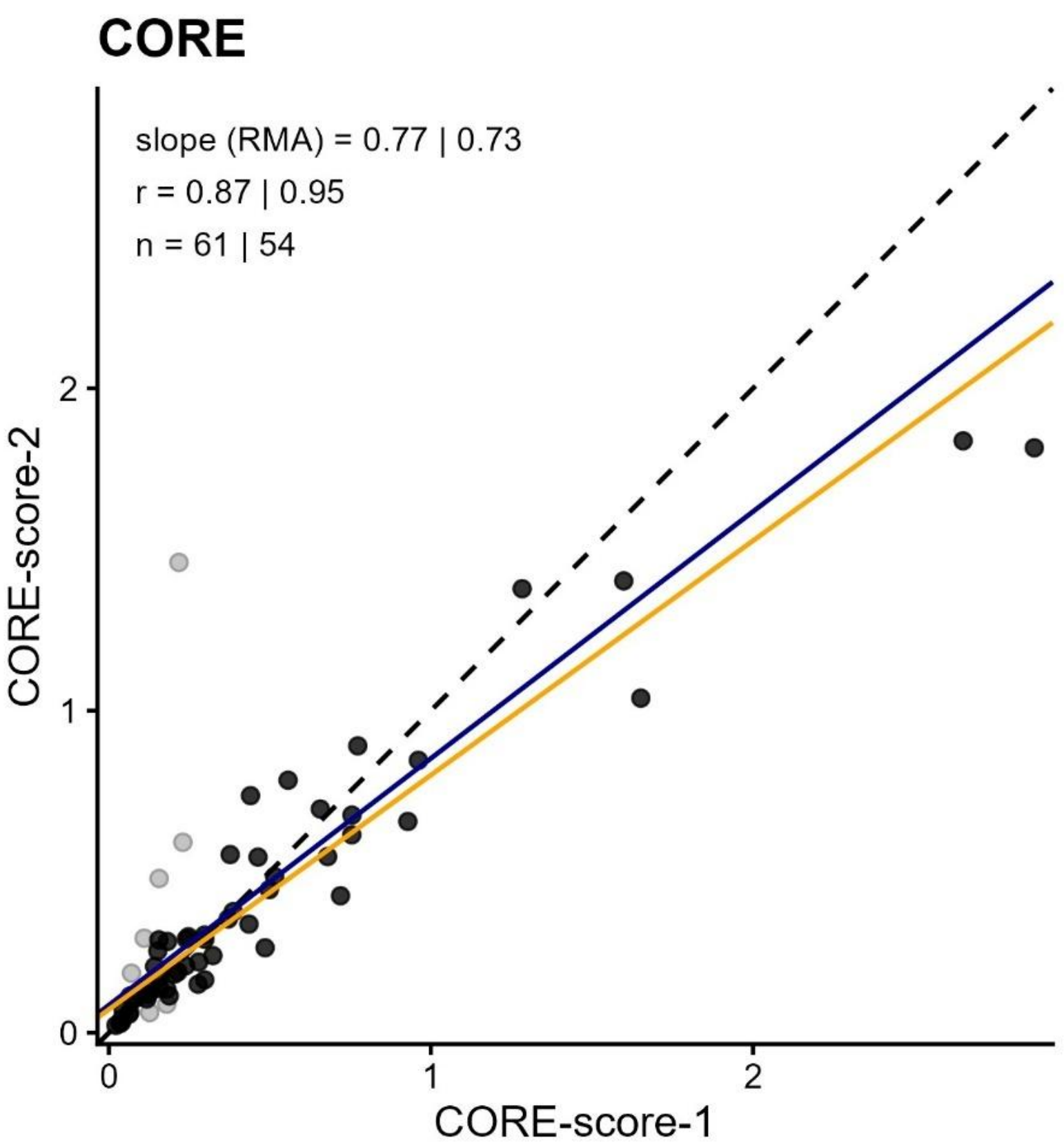
CORE
slope (RMA) = 0.77 | 0.73
r = 0.87 | 0.95
n = 61 | 54
CORE-score-2
CORE-score-1
0
1
2

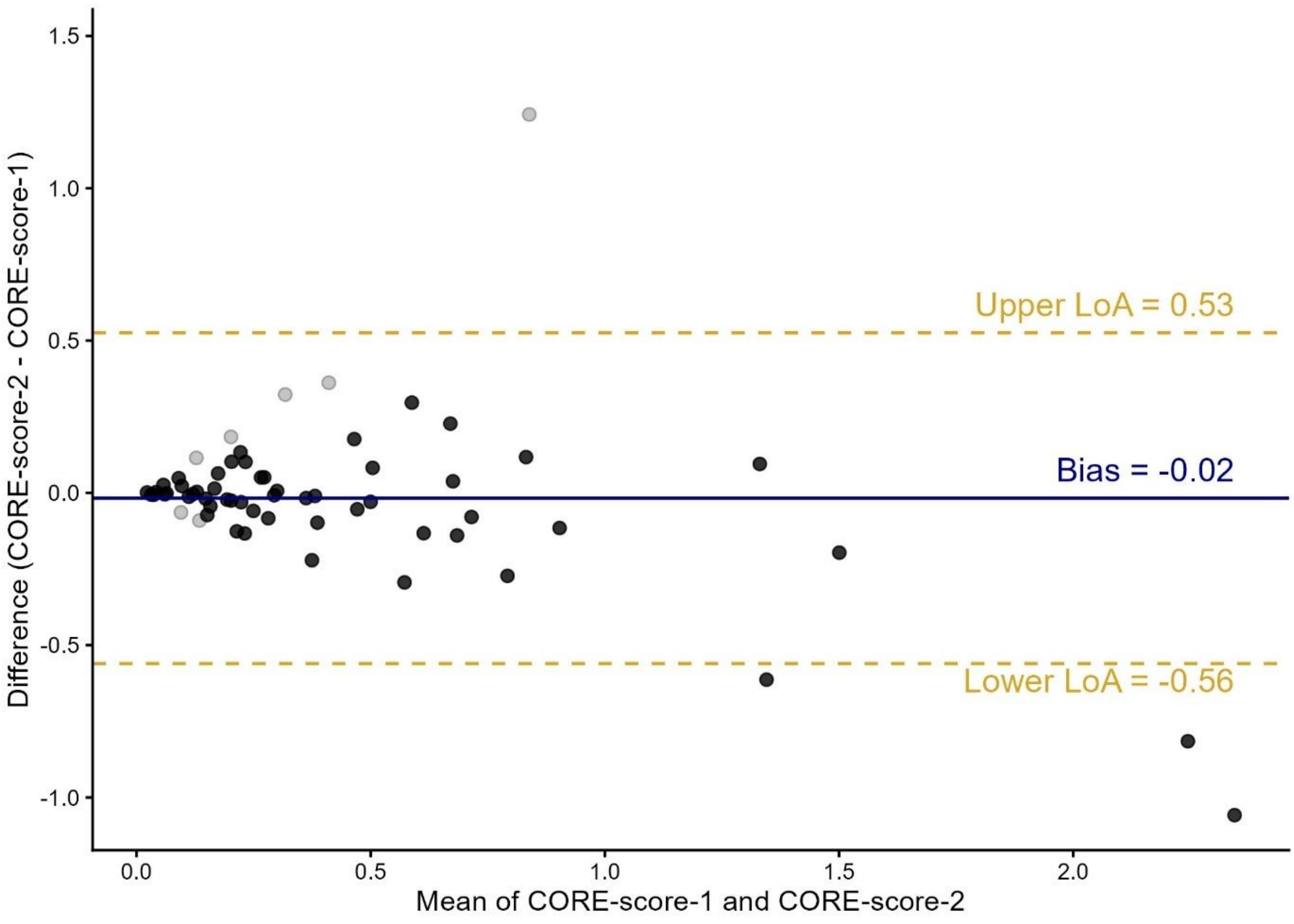
Upper LoA = 0.53
Bias = -0.02
Lower LoA = -0.56
Difference (CORE-score-2 - CORE-score-1)
Mean of CORE-score-1 and CORE-score-2
1.5
1.0
0.5
0.0
-0.5
-1.0
0.0
0.5
1.0
1.5
2.0

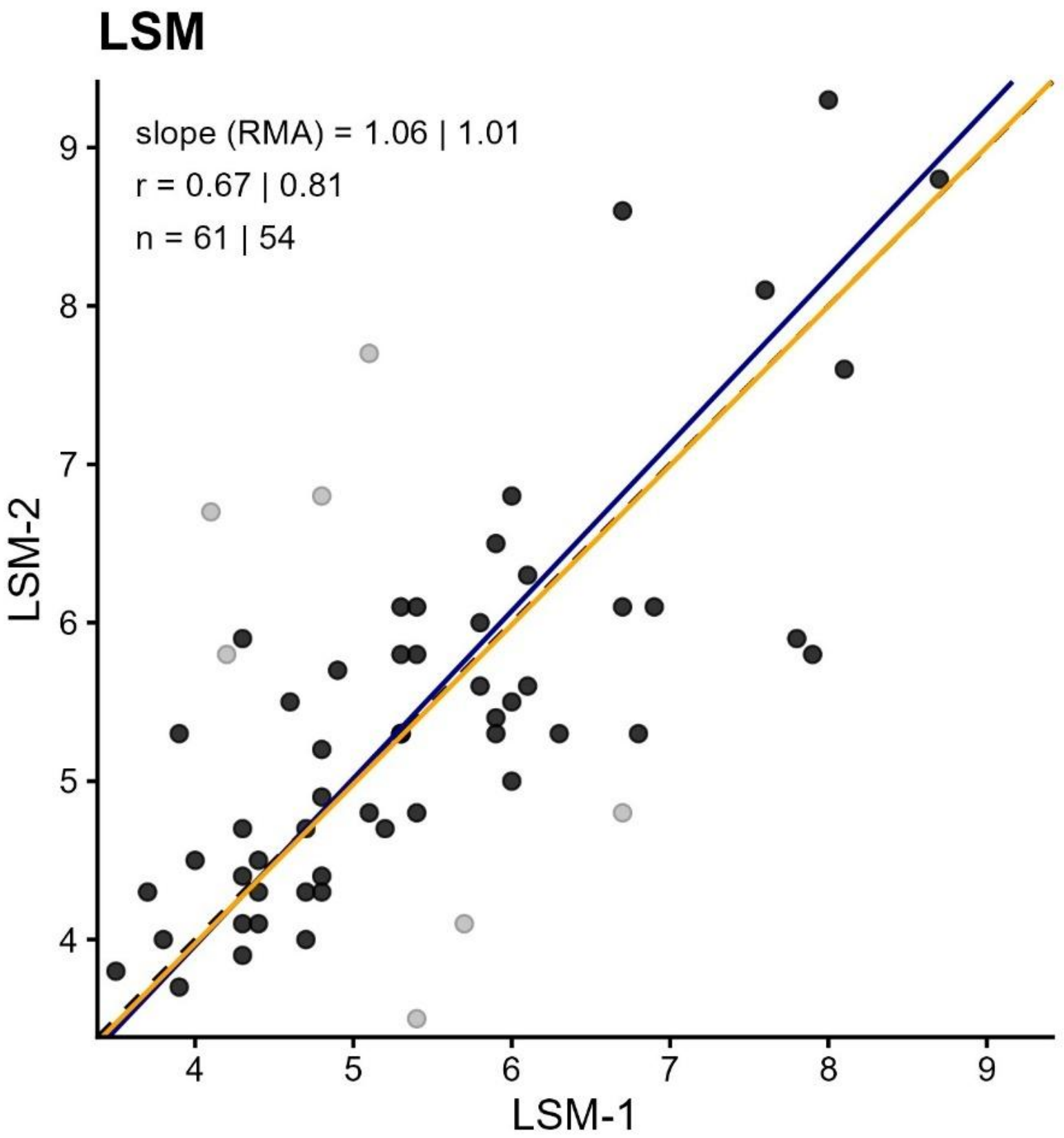
LSM
slope (RMA) = 1.06 | 1.01
r = 0.67 | 0.81
n = 61 | 54
LSM-2
LSM-1

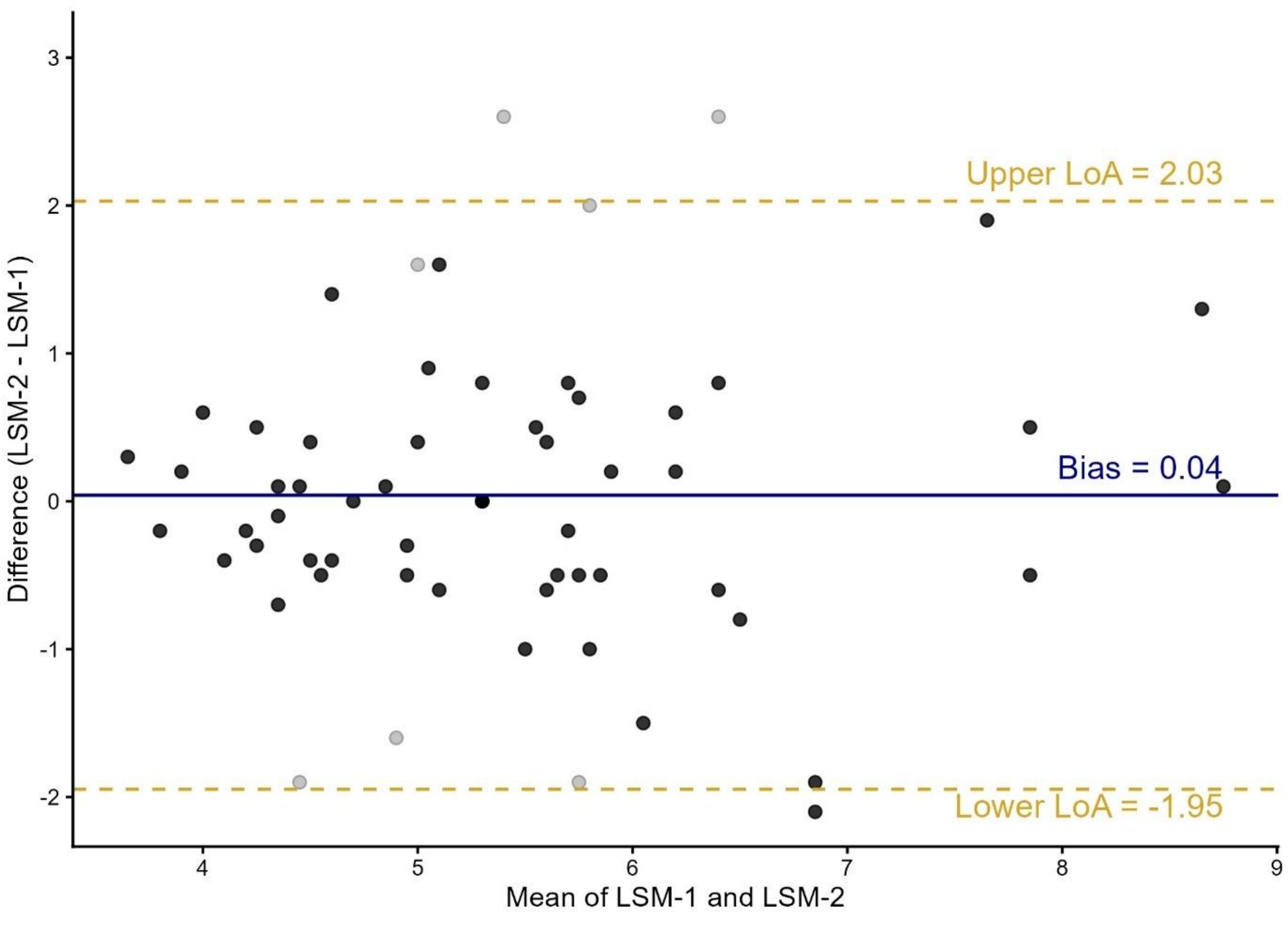
Upper LoA = 2.03
Bias = 0.04
Lower LoA = -1.95
Difference (LSM-2 - LSM-1)
Mean of LSM-1 and LSM-2

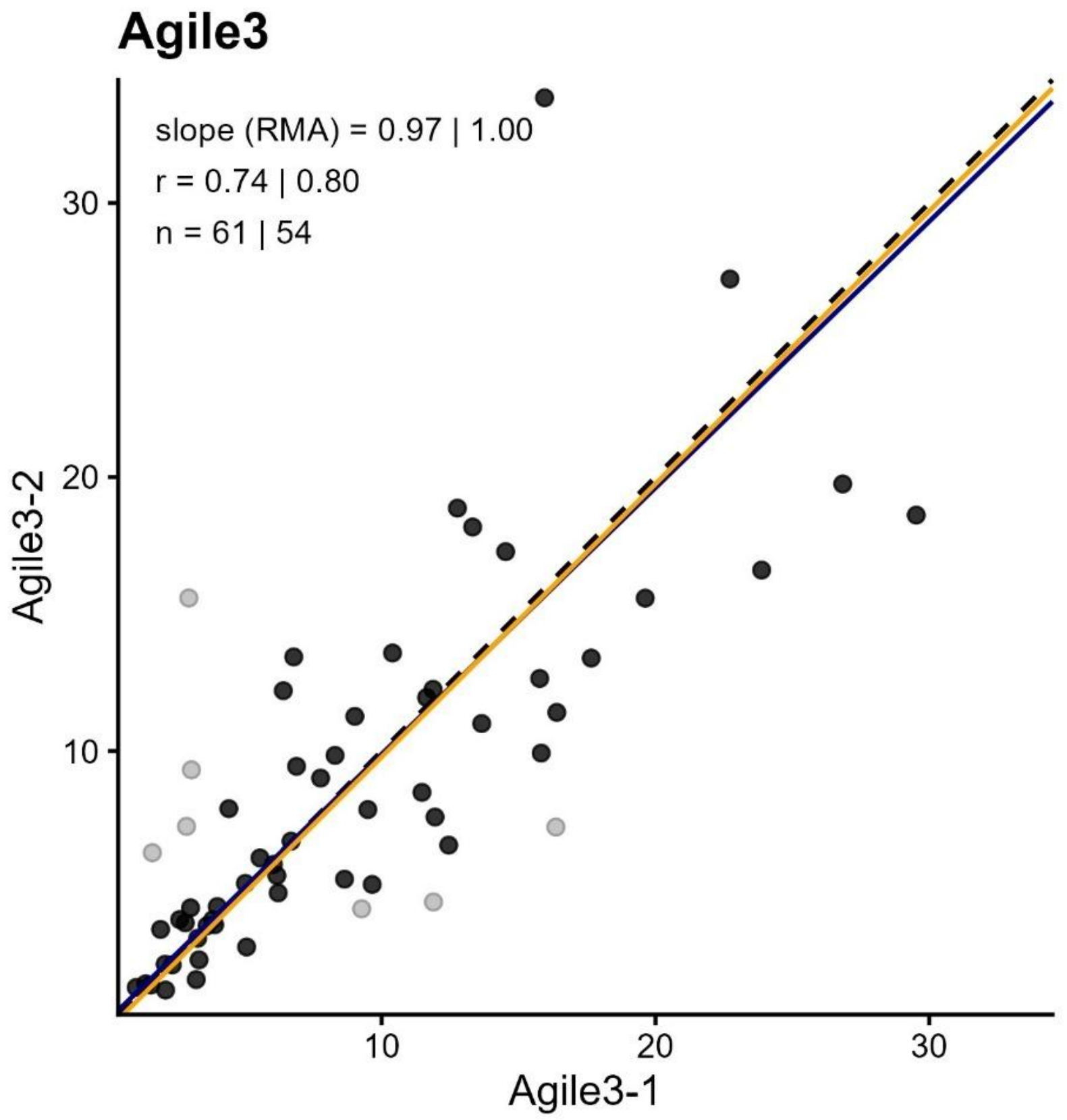
Agile3
slope (RMA) = 0.97 | 1.00
r = 0.74 | 0.80
n = 61 | 54
Agile3-2
Agile3-1
10
20
30

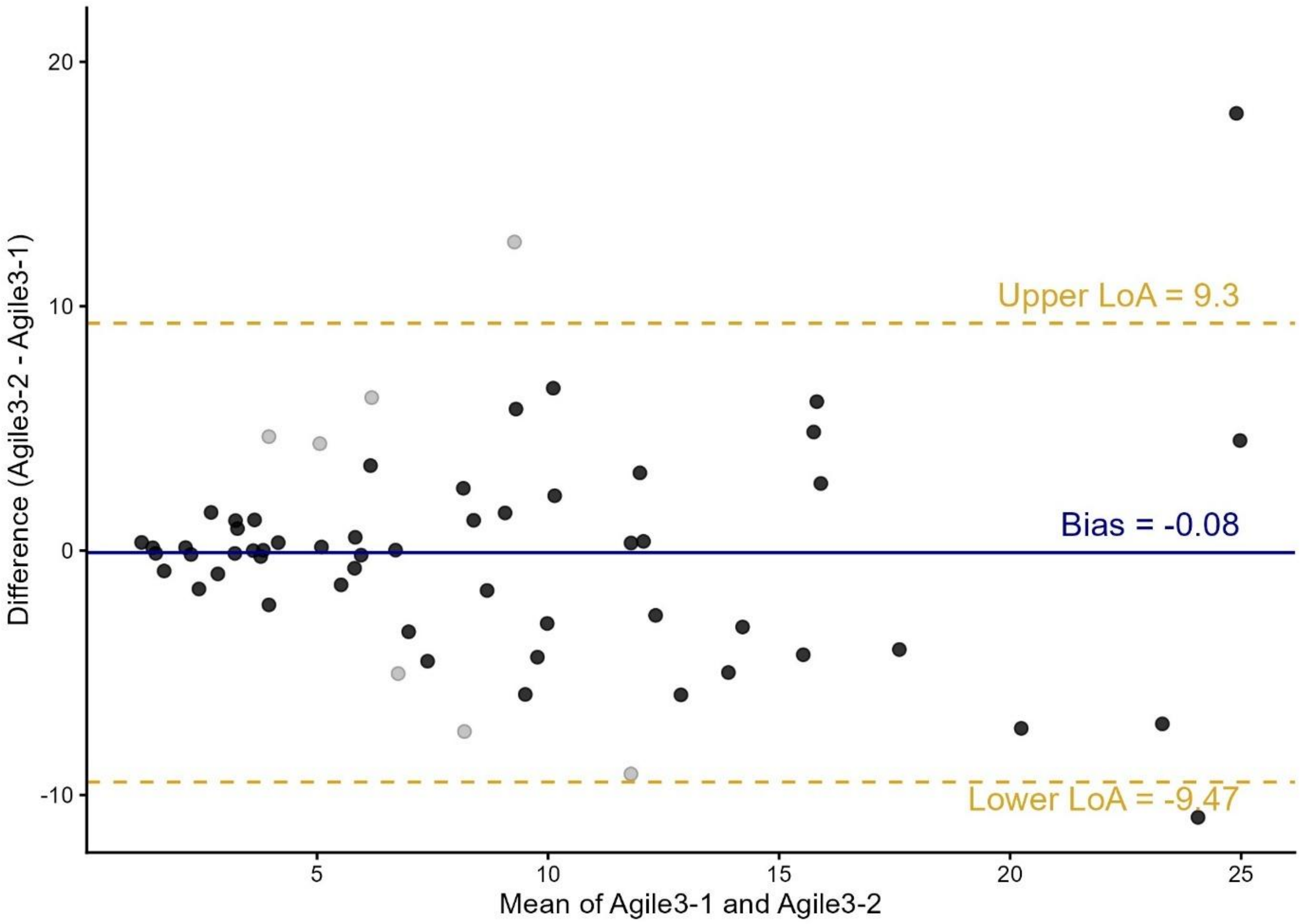
Upper LoA = 9.3
Bias = -0.08
Lower LoA = -9.47
Difference (Agile3-2 - Agile3-1)
Mean of Agile3-1 and Agile3-2
20
10
0
-10
5
10
15
20
25

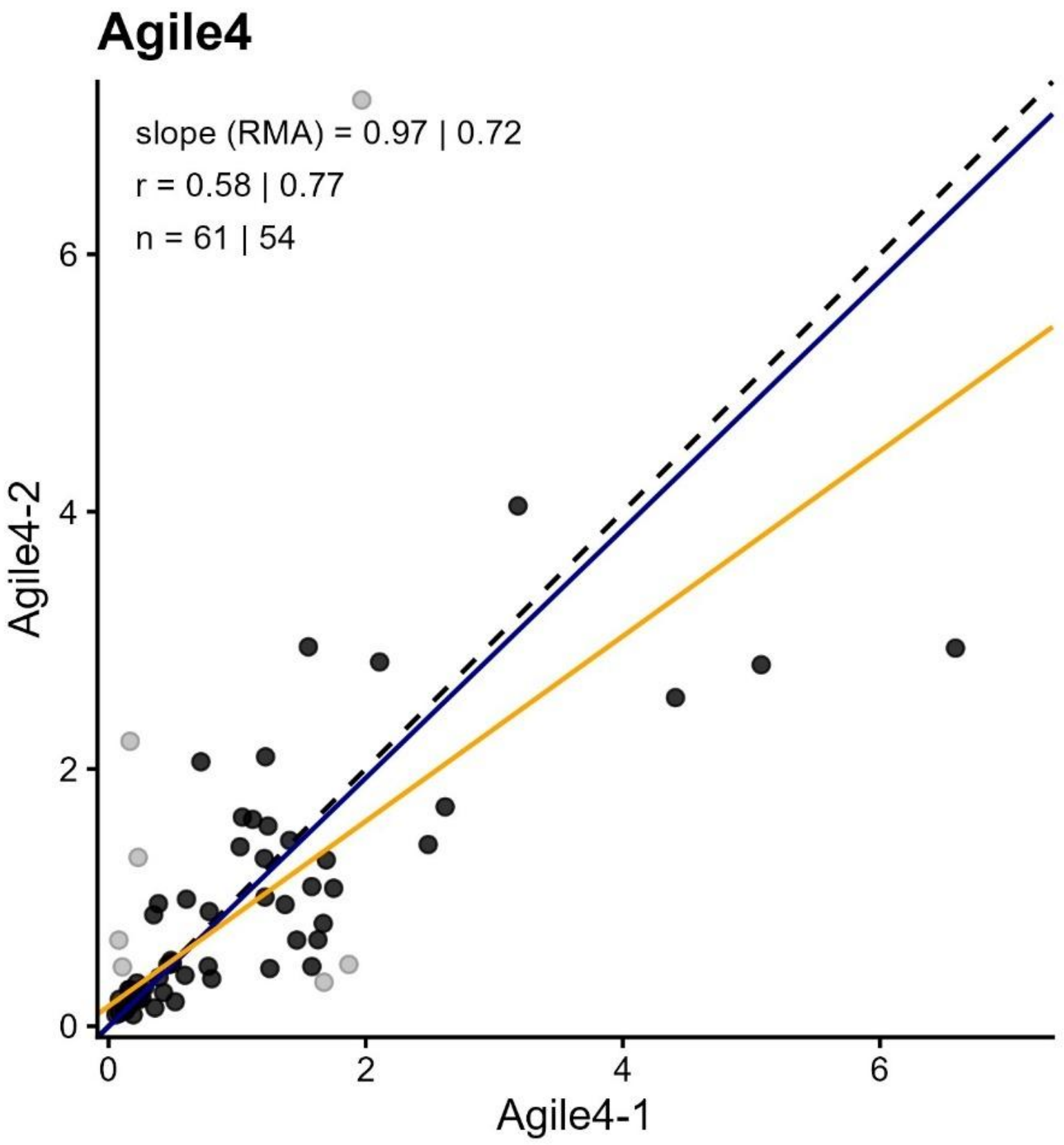
Agile4
slope (RMA) = 0.97 | 0.72
r = 0.58 | 0.77
n = 61 | 54
Agile4-2
Agile4-1
0
2
4
6

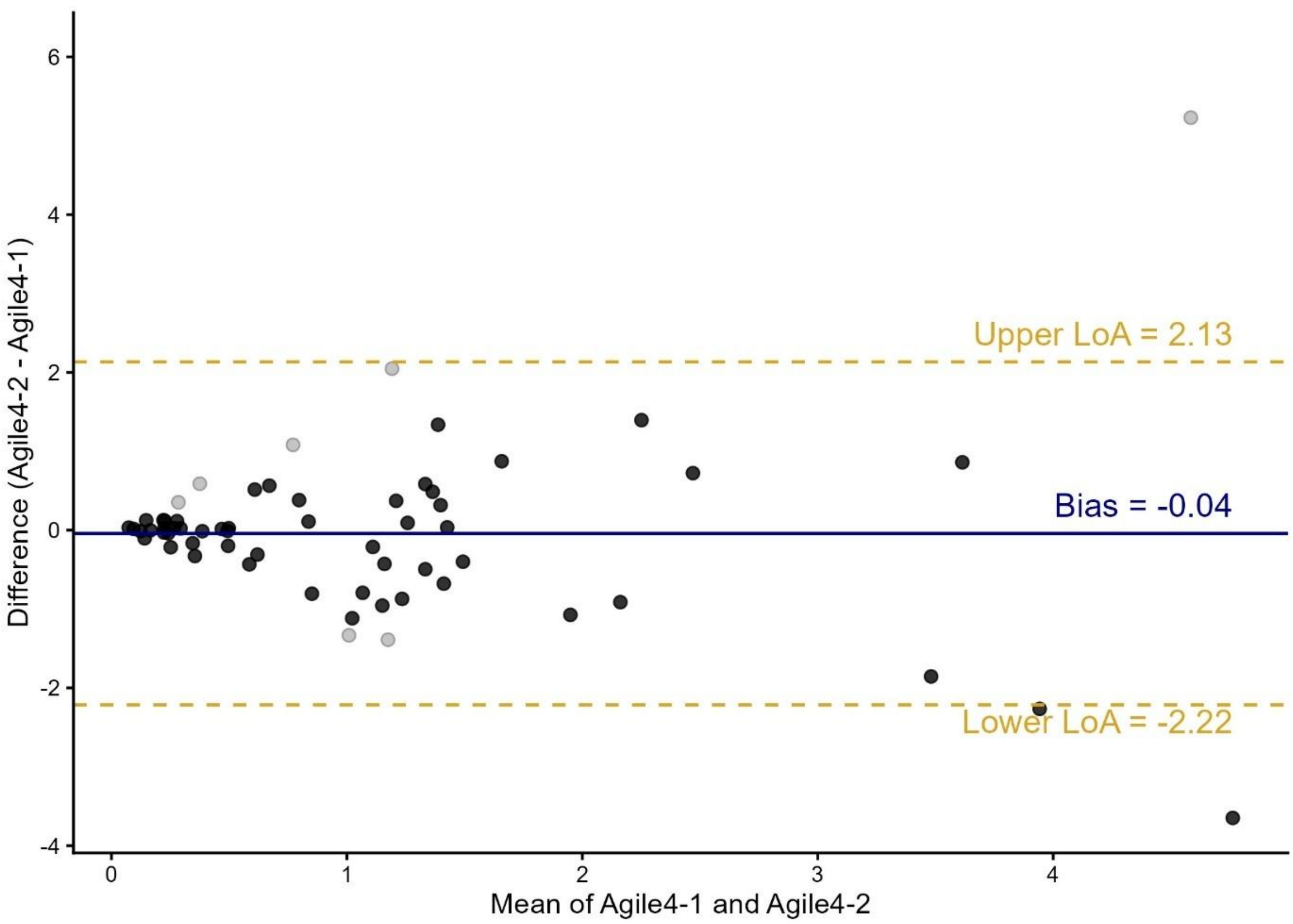
Upper LoA = 2.13
Bias = -0.04
Lower LoA = -2.22
Difference (Agile4-2 - Agile4-1)
Mean of Agile4-1 and Agile4-2
6
4
2
0
-2
-4
0
1
2
3
4

Reproducibility cohort II - suspected fibrosis

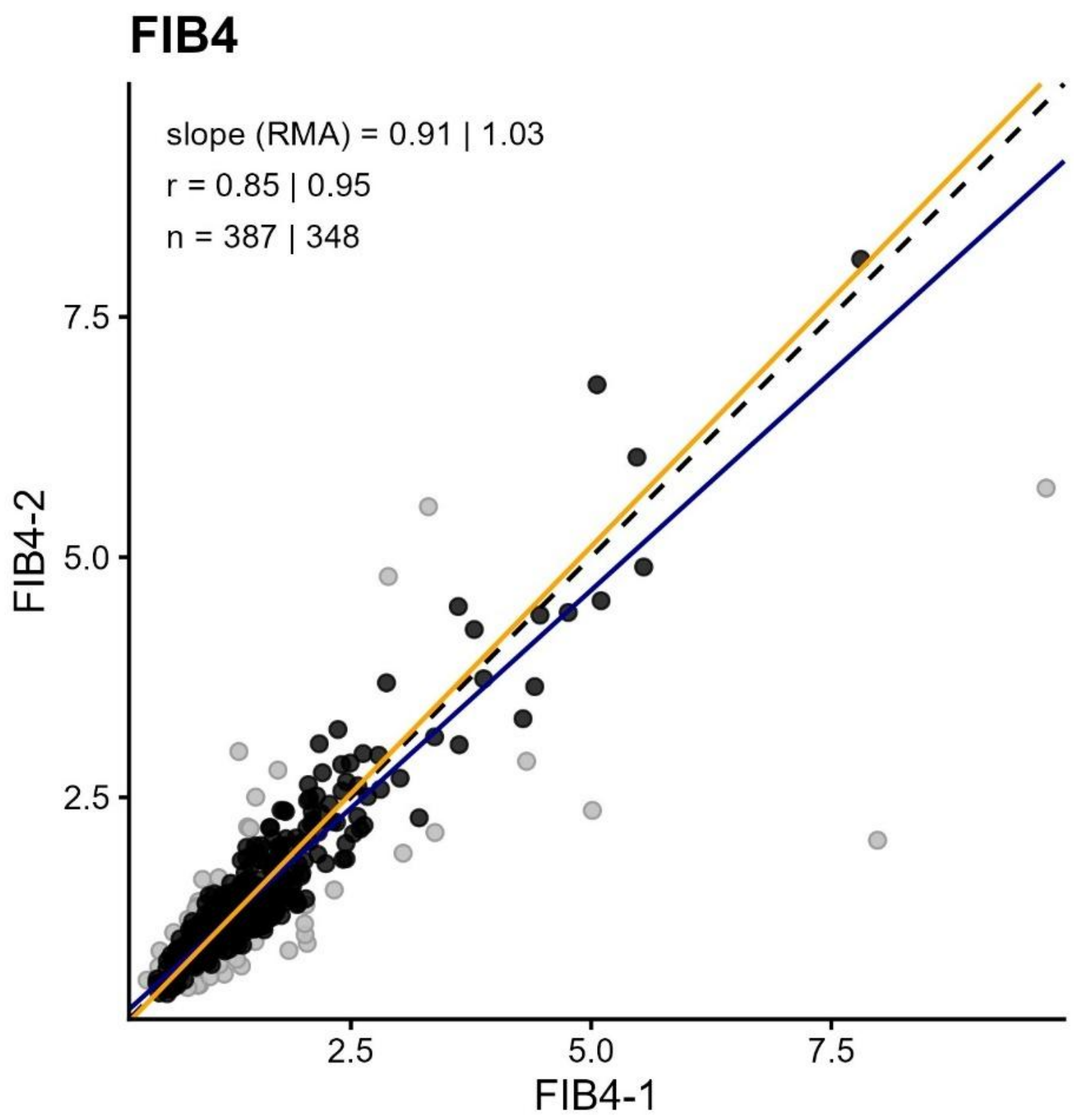

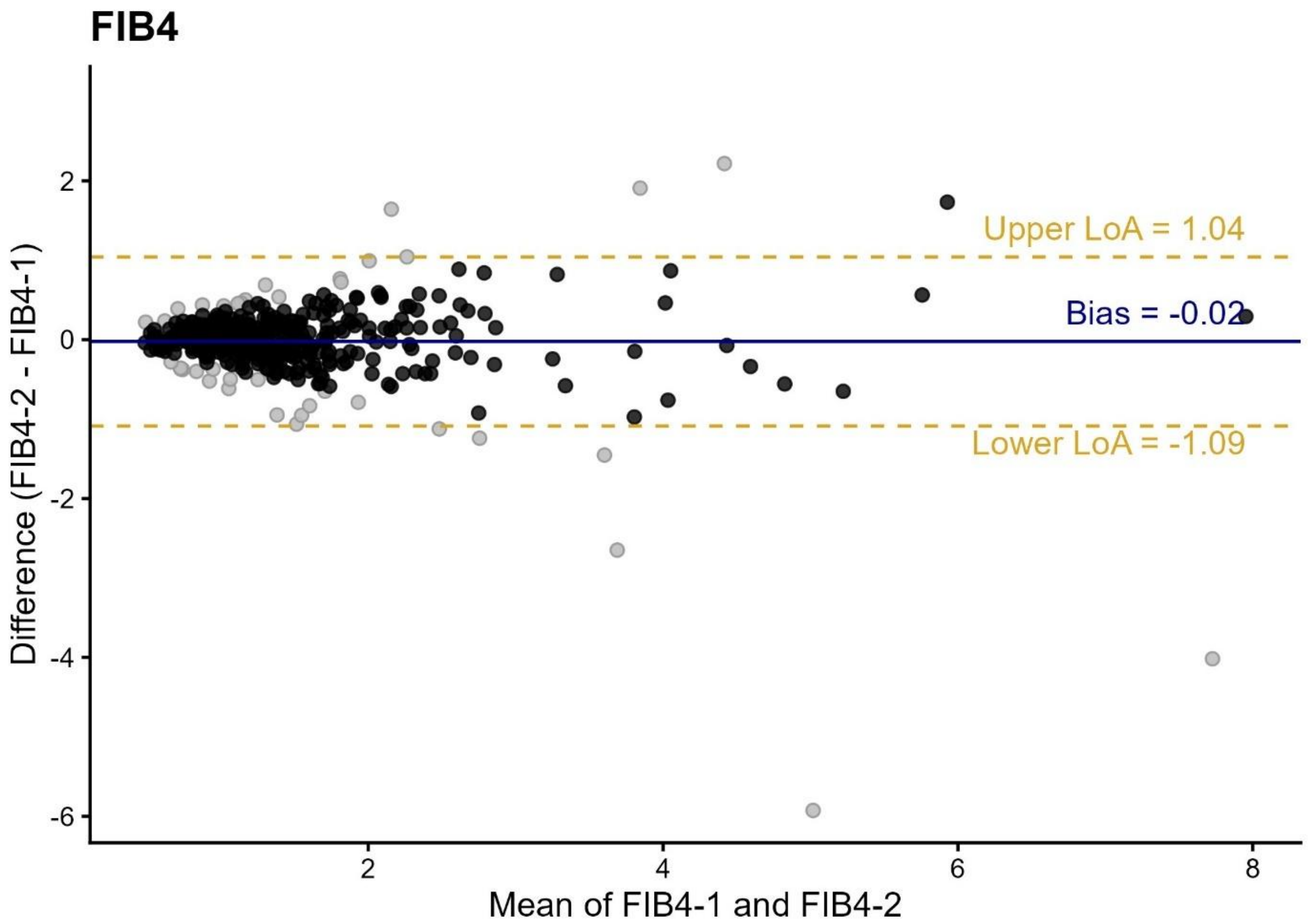

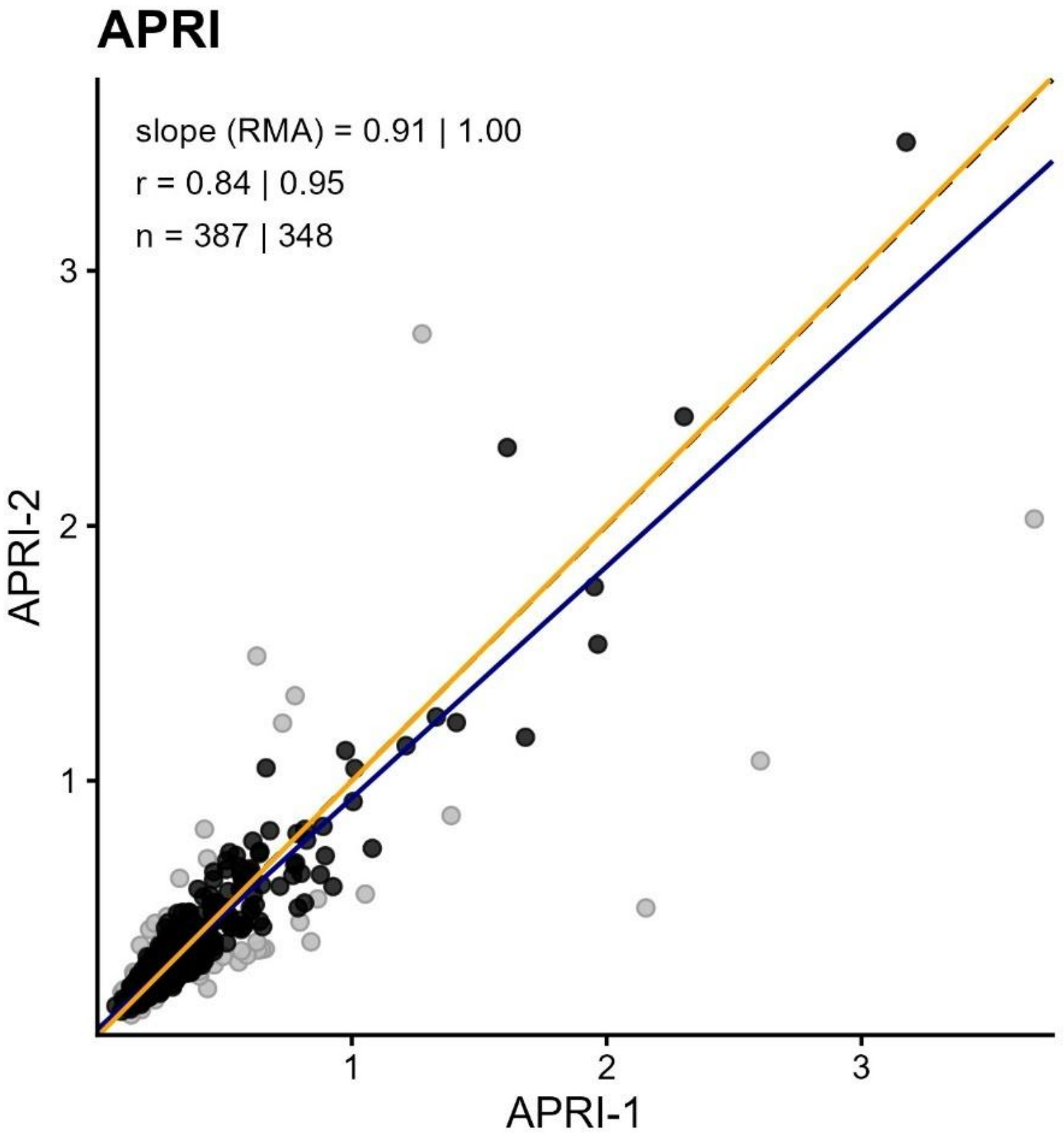
APRI
slope (RMA) = 0.91 | 1.00
r = 0.84 | 0.95
n = 387 | 348
APRI-2
APRI-1
1
2
3

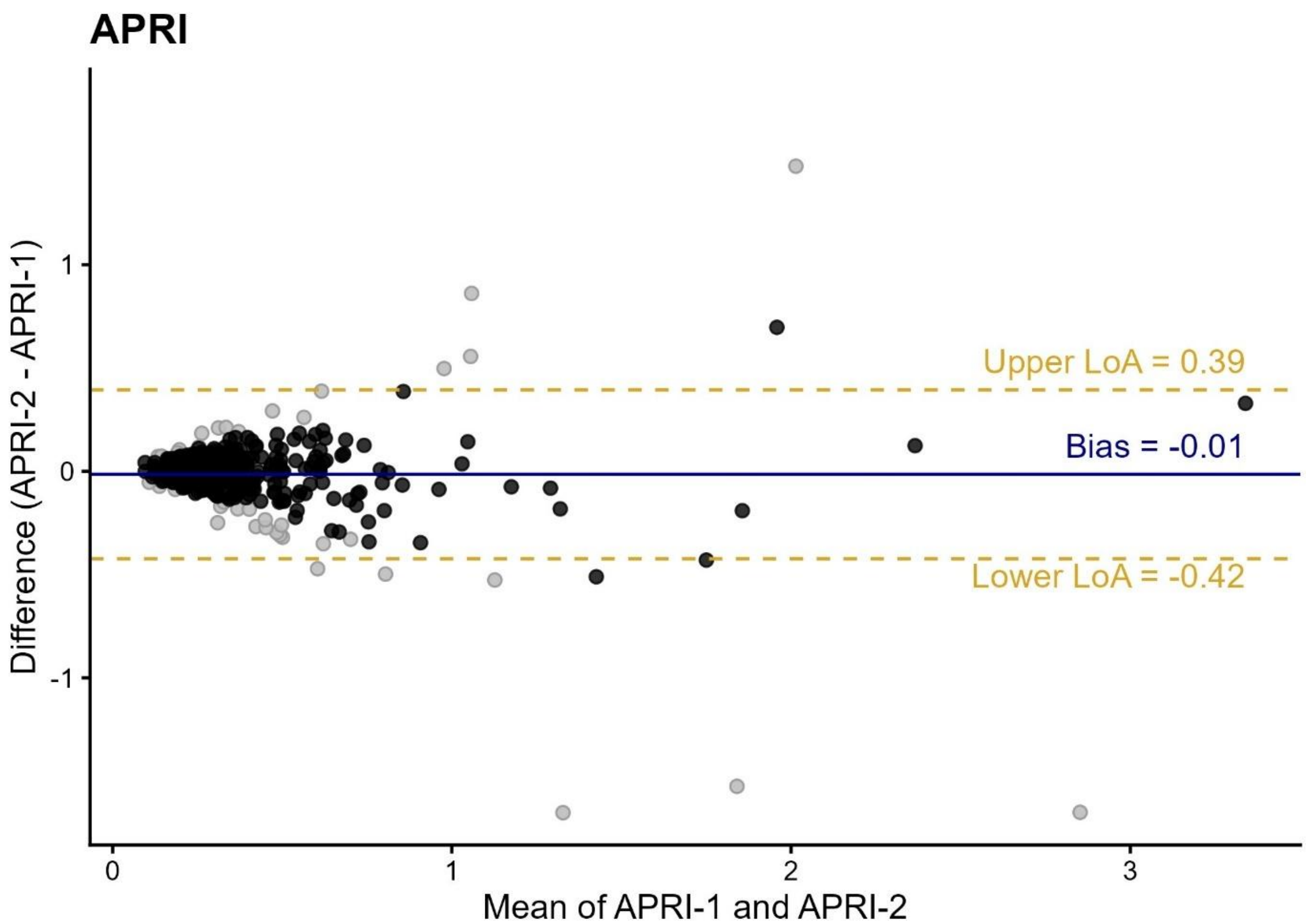
APRI
Upper LoA = 0.39
Bias = -0.01
Lower LoA = -0.42
Difference (APRI-2 - APRI-1)
Mean of APRI-1 and APRI-2
1
0
-1
2
3

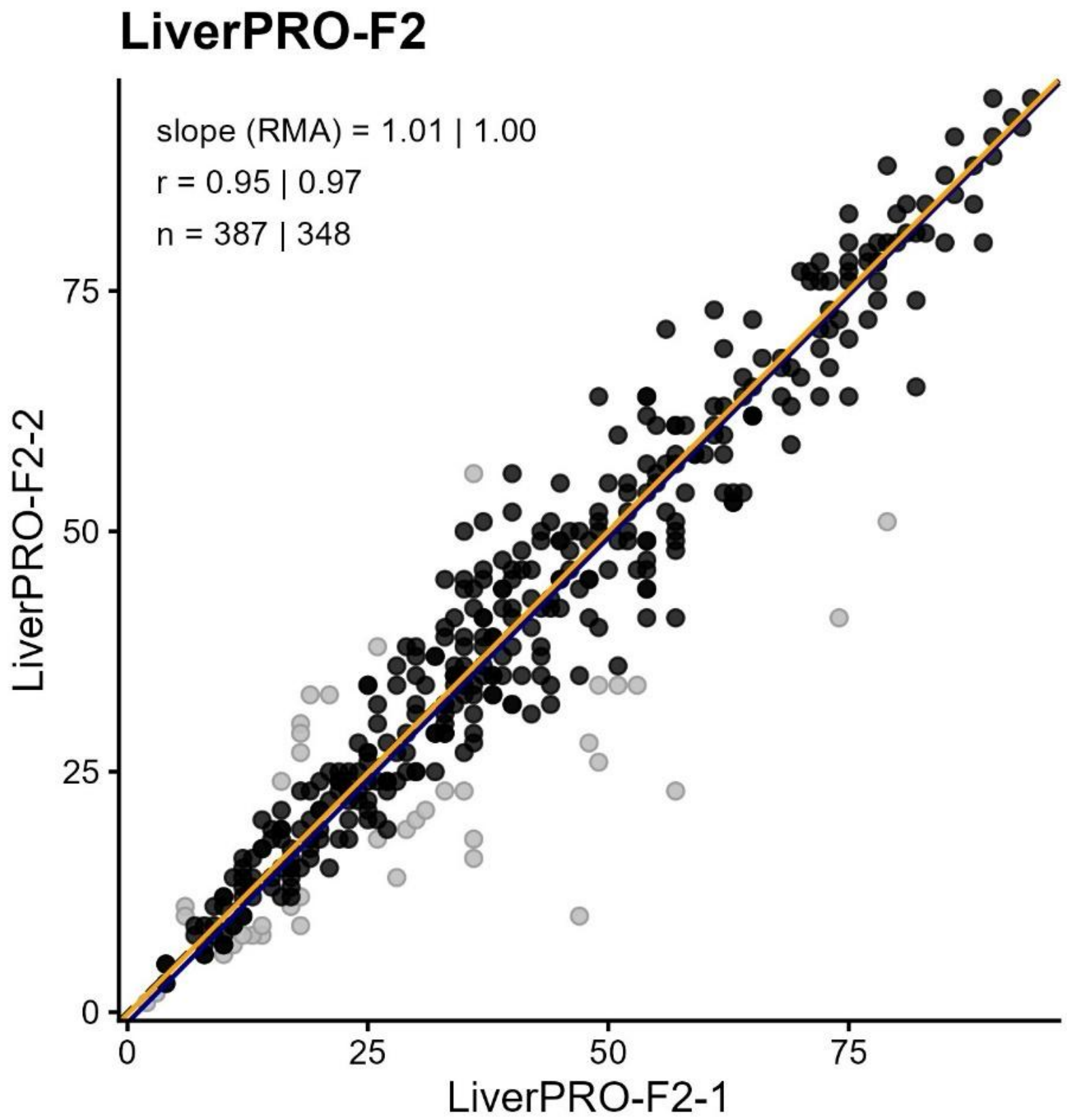
LiverPRO-F2
slope (RMA) = 1.01 | 1.00
r = 0.95 | 0.97
n = 387 | 348
LiverPRO-F2-2
LiverPRO-F2-1
0
25
50
75

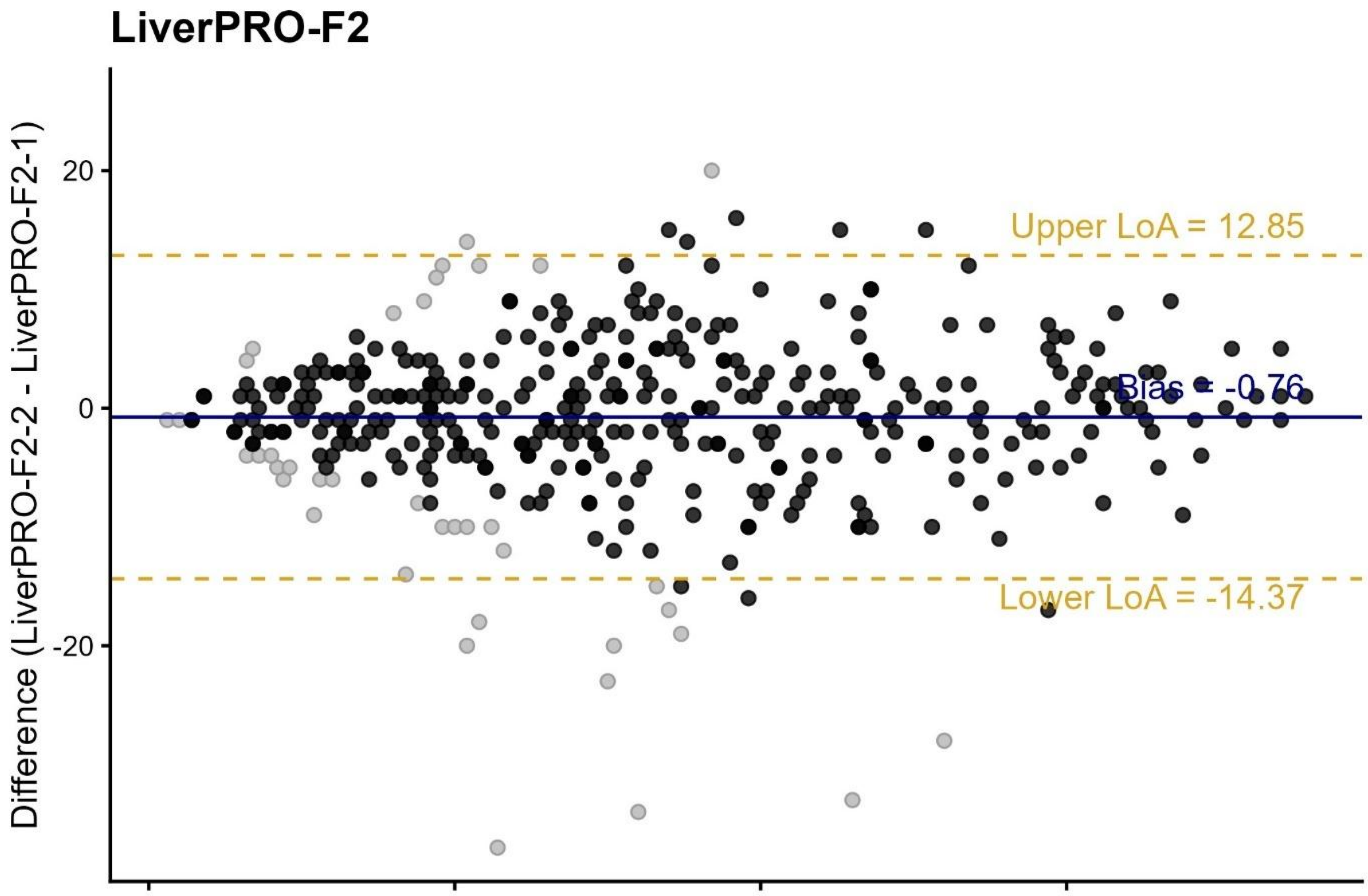
LiverPRO-F2
Upper LoA = 12.85
Bias = -0.76
Lower LoA = -14.37
Difference (LiverPRO-F2-2 - LiverPRO-F2-1)
Mean of LiverPRO-F2-1 and LiverPRO-F2-2
20
0
-20
0
25
50
75

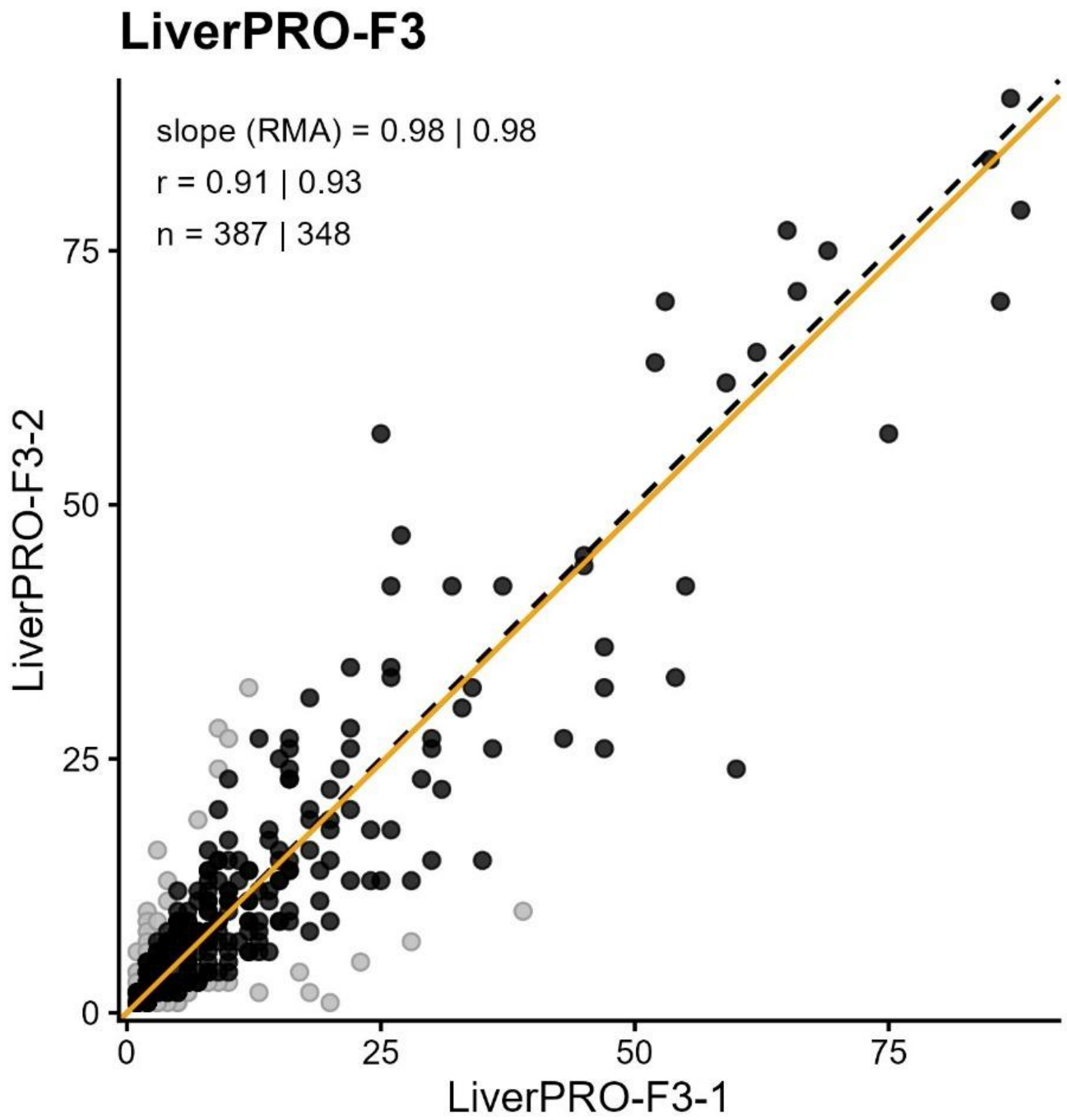
LiverPRO-F3
slope (RMA) = 0.98 | 0.98
r = 0.91 | 0.93
n = 387 | 348
LiverPRO-F3-2
LiverPRO-F3-1
0
25
50
75

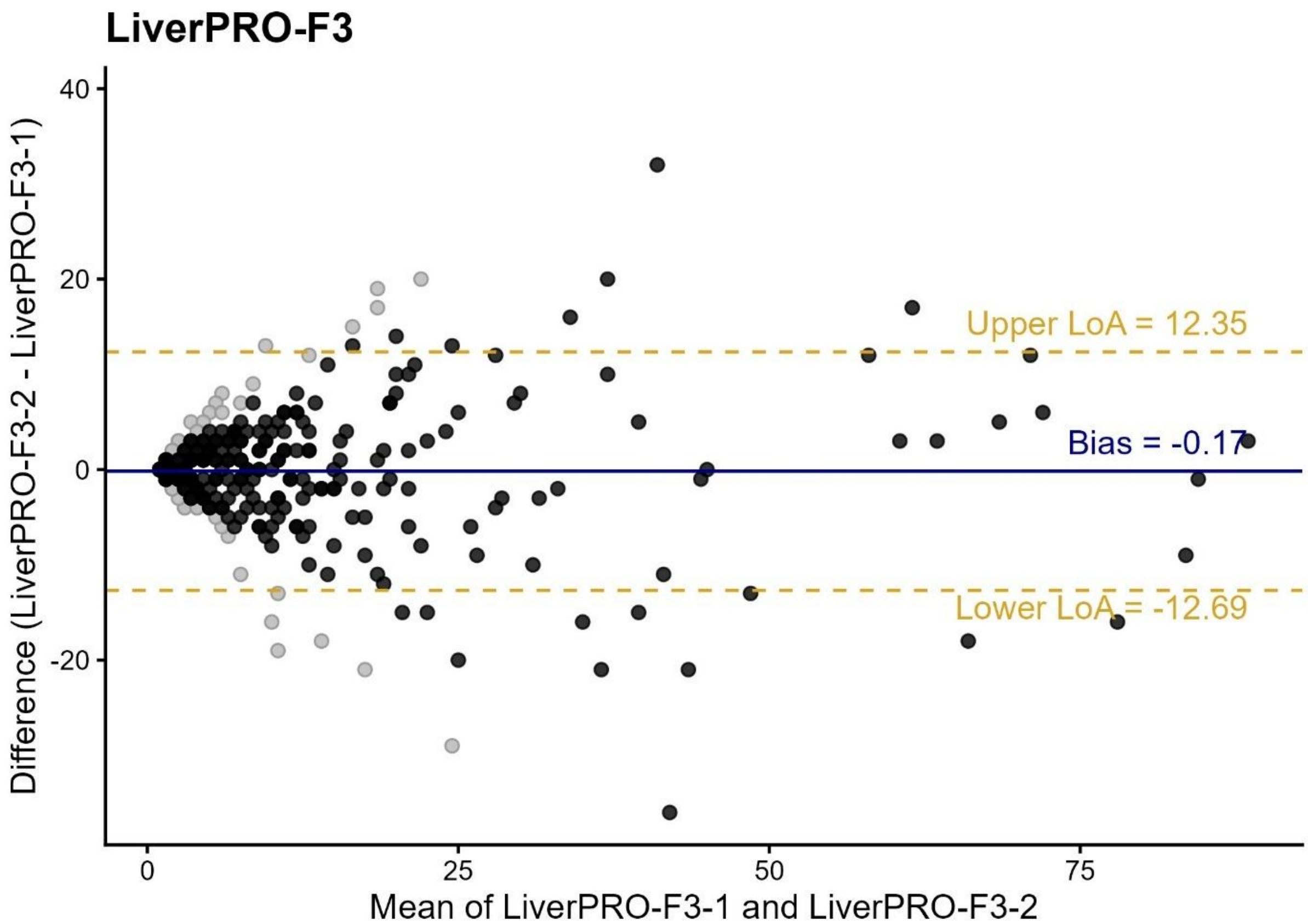
LiverPRO-F3
Upper LoA = 12.35
Bias = -0.17
Lower LoA = -12.69
Difference (LiverPRO-F3-2 - LiverPRO-F3-1)
Mean of LiverPRO-F3-1 and LiverPRO-F3-2
40
20
0
-20
0
25
50
75

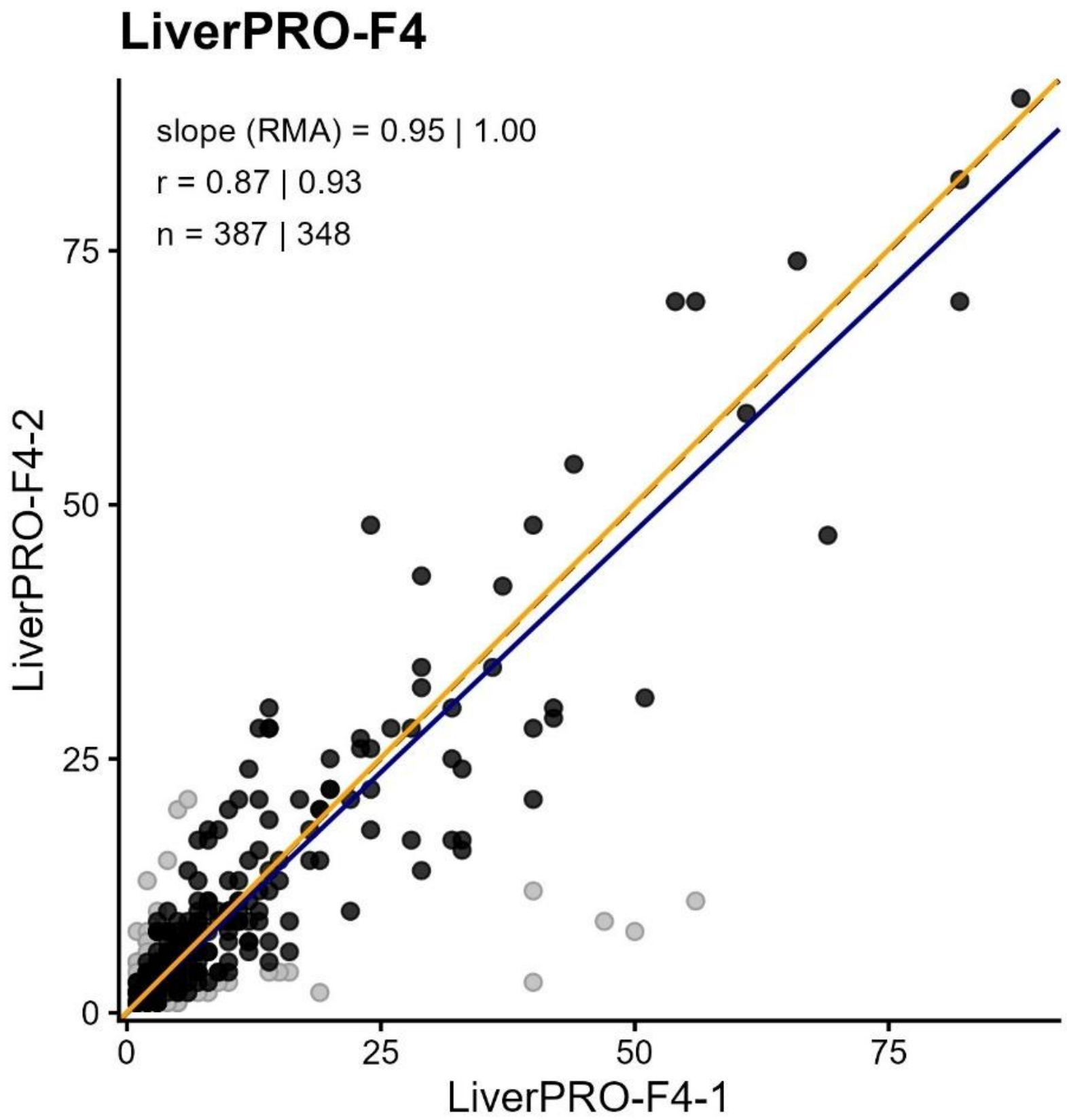
LiverPRO-F4
slope (RMA) = 0.95 | 1.00
r = 0.87 | 0.93
n = 387 | 348
LiverPRO-F4-2
LiverPRO-F4-1
0
25
50
75

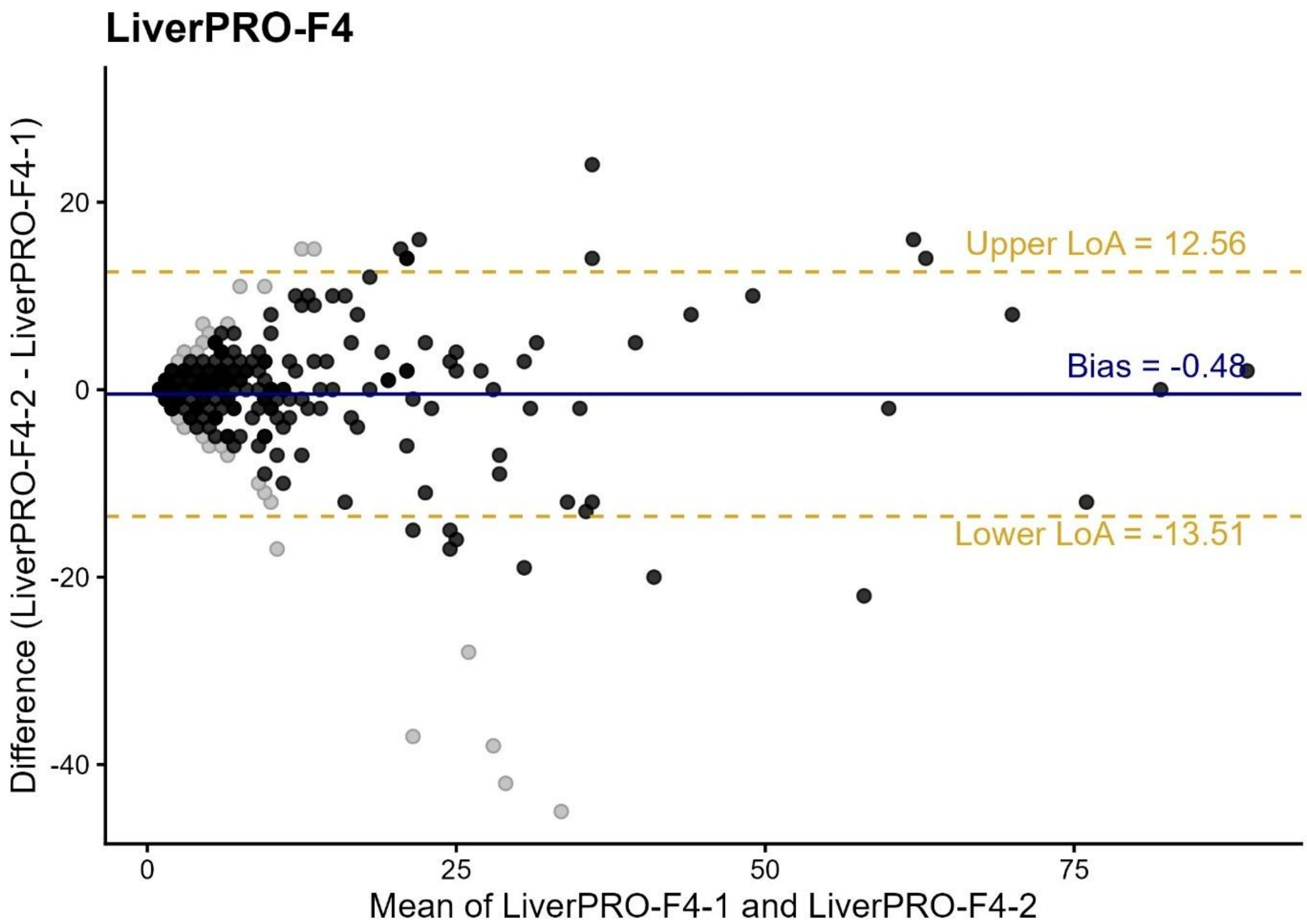
LiverPRO-F4
Upper LoA = 12.56
Bias = -0.48
Lower LoA = -13.51
Difference (LiverPRO-F4-2 - LiverPRO-F4-1)
Mean of LiverPRO-F4-1 and LiverPRO-F4-2
20
0
-20
-40
0
25
50
75

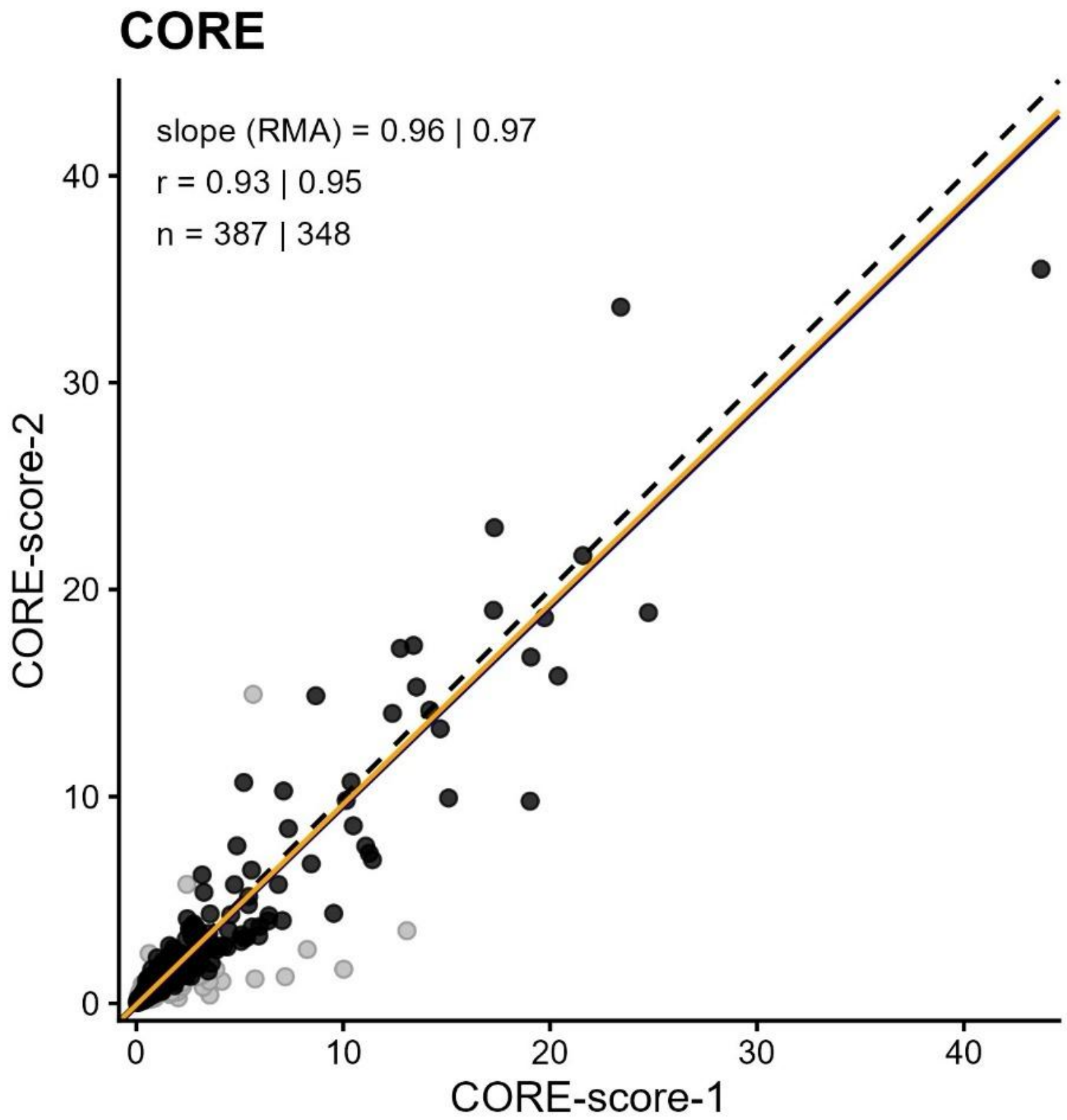
CORE
slope (RMA) = 0.96 | 0.97
r = 0.93 | 0.95
n = 387 | 348
CORE-score-2
CORE-score-1
0
10
20
30
40

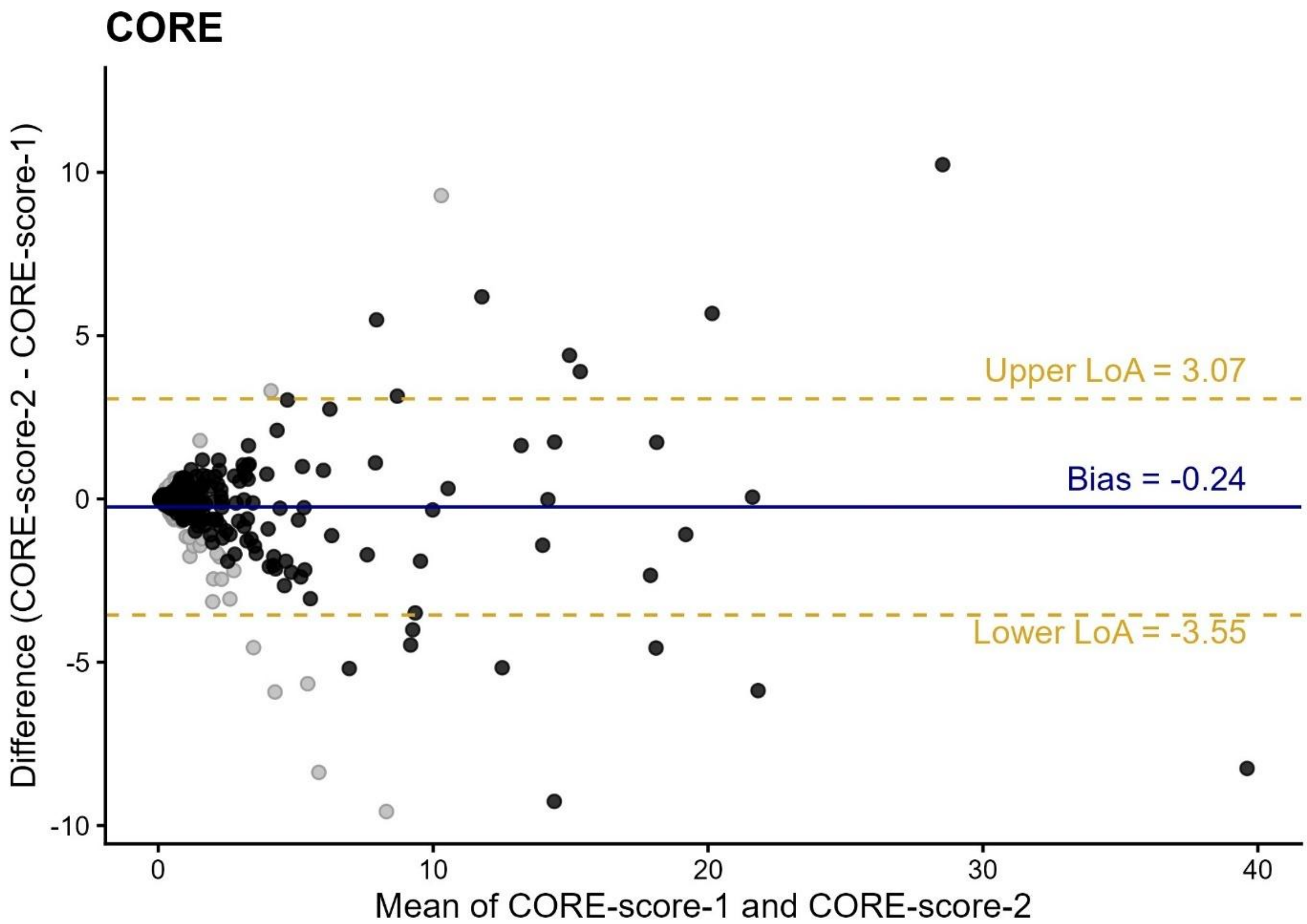
CORE
Upper LoA = 3.07
Bias = -0.24
Lower LoA = -3.55
Difference (CORE-score-2 - CORE-score-1)
Mean of CORE-score-1 and CORE-score-2
10
5
0
-5
-10
20
30
40

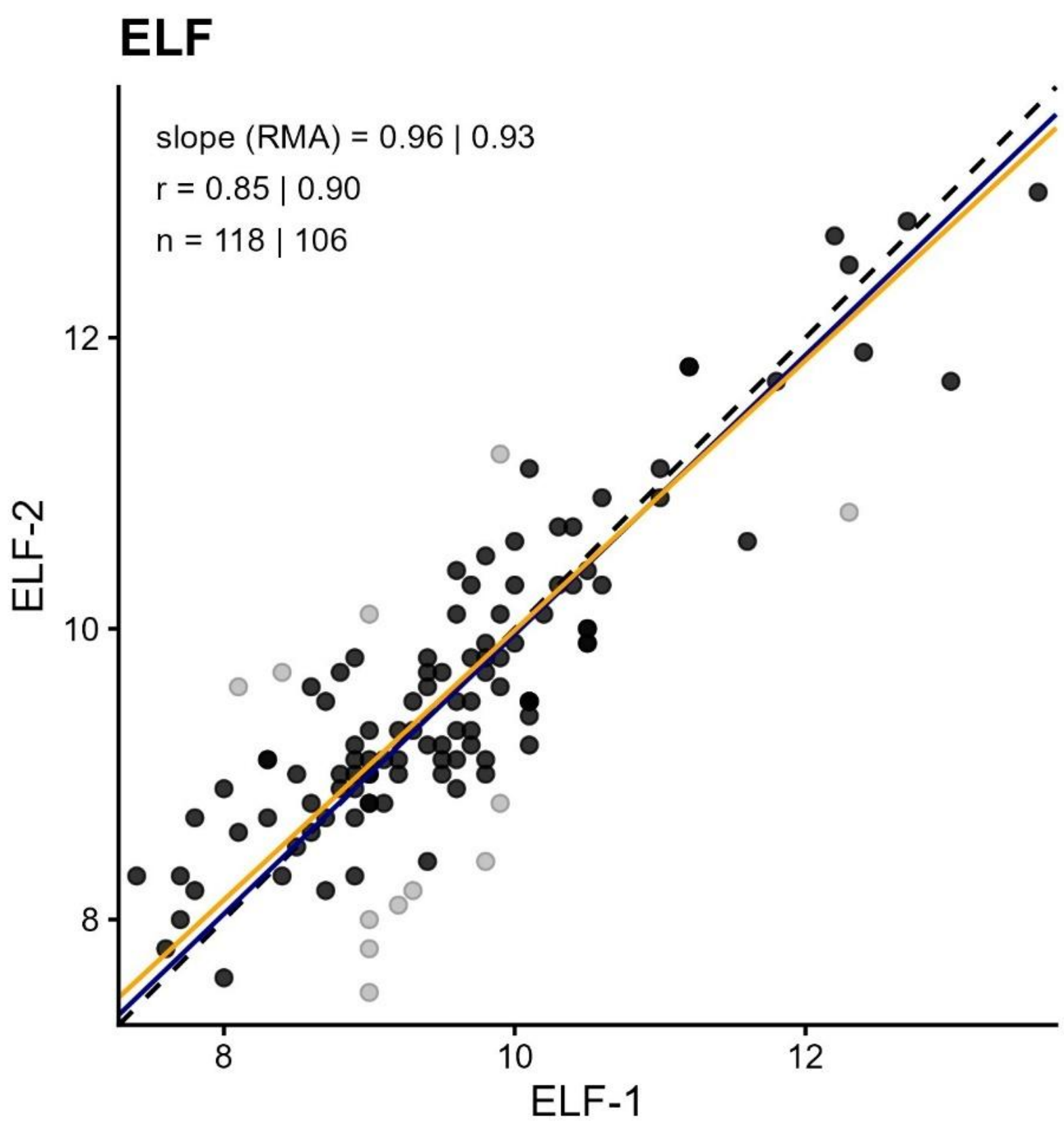
ELF
slope (RMA) = 0.96 | 0.93
r = 0.85 | 0.90
n = 118 | 106
ELF-2
ELF-1
8
10
12

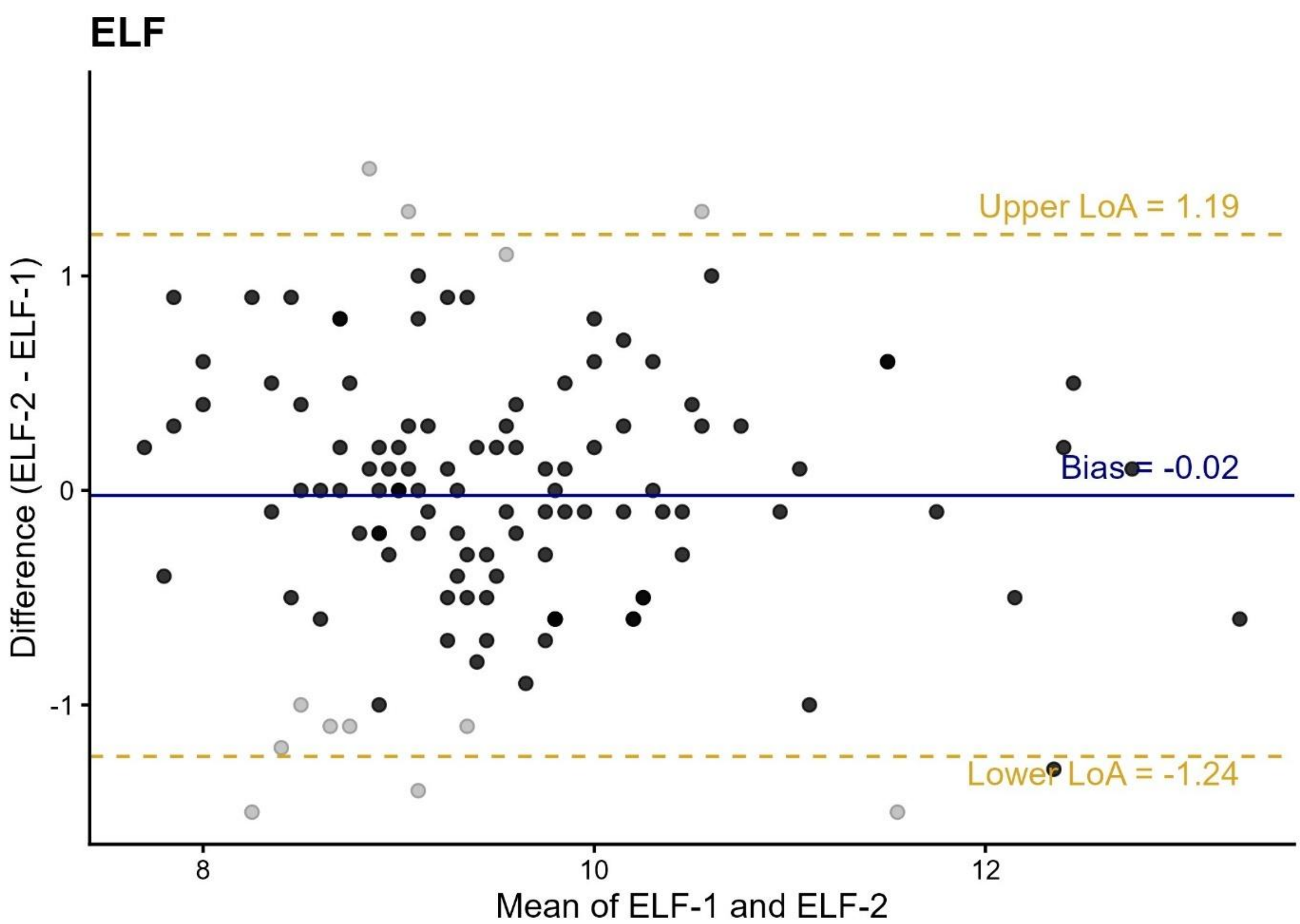
ELF
Upper LoA = 1.19
Bias = -0.02
Lower LoA = -1.24
Difference (ELF-2 - ELF-1)
Mean of ELF-1 and ELF-2
1
0
-1
8
10
12

Reproducibility cohort III - suspected portal hypertension

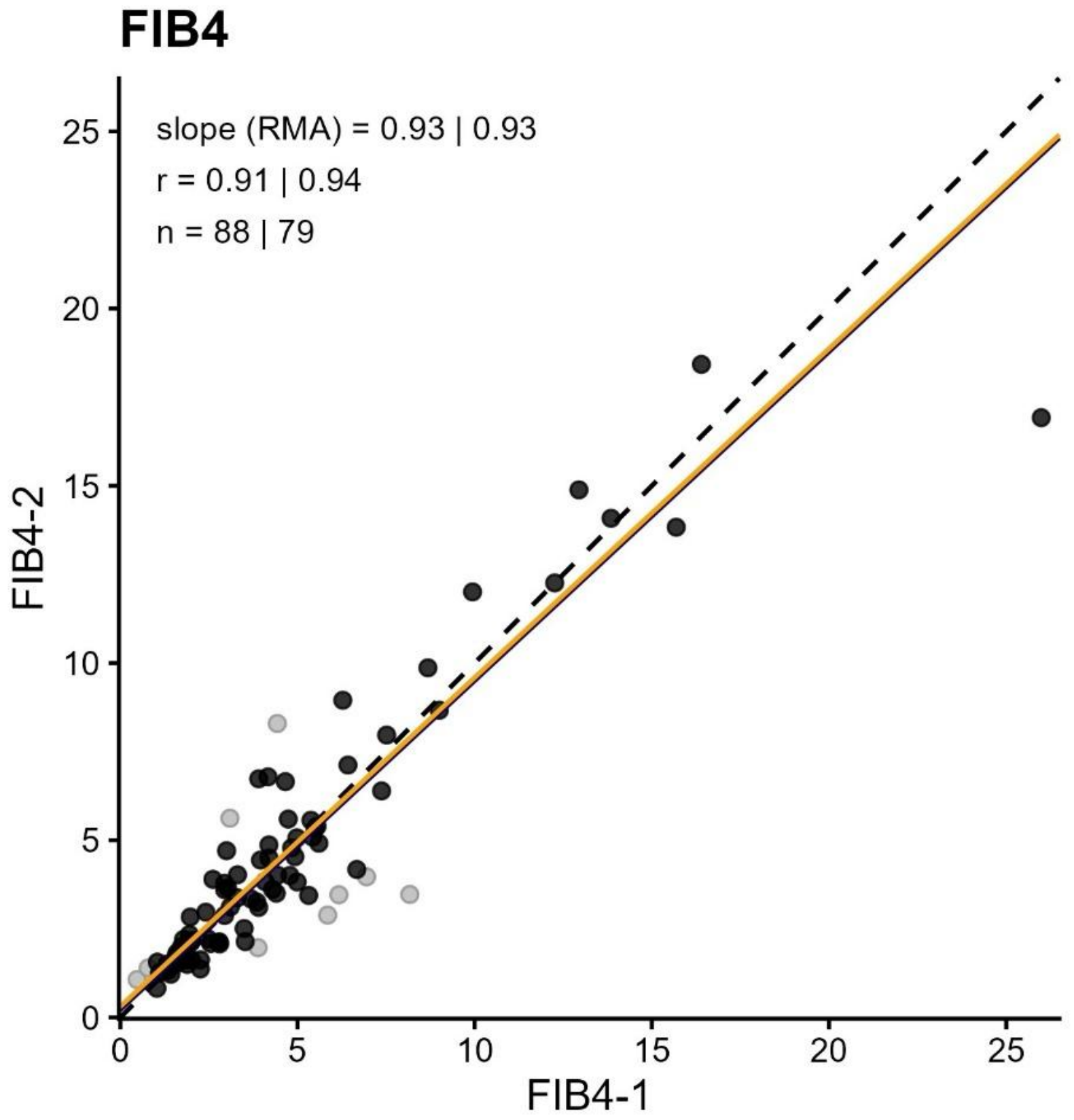

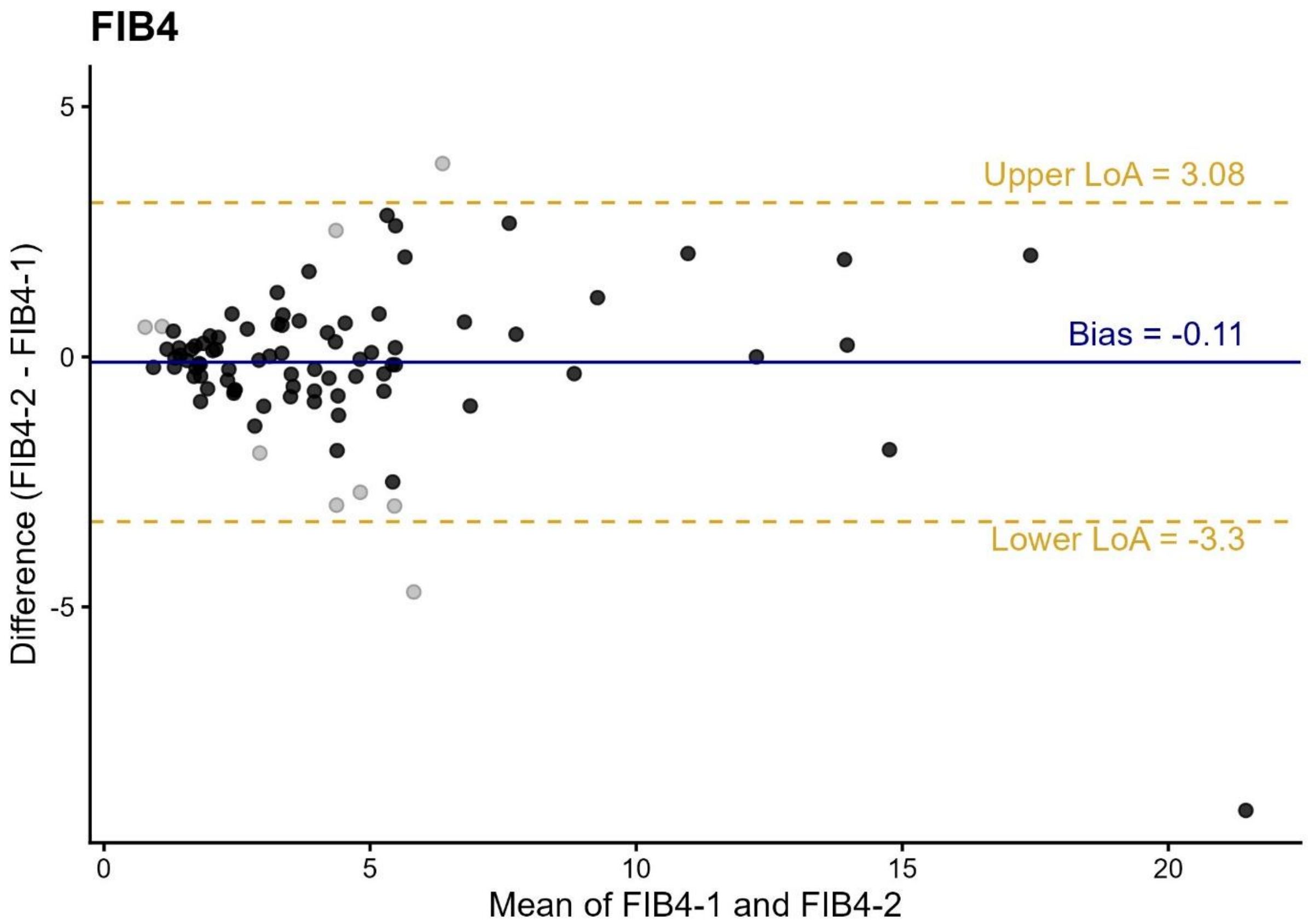

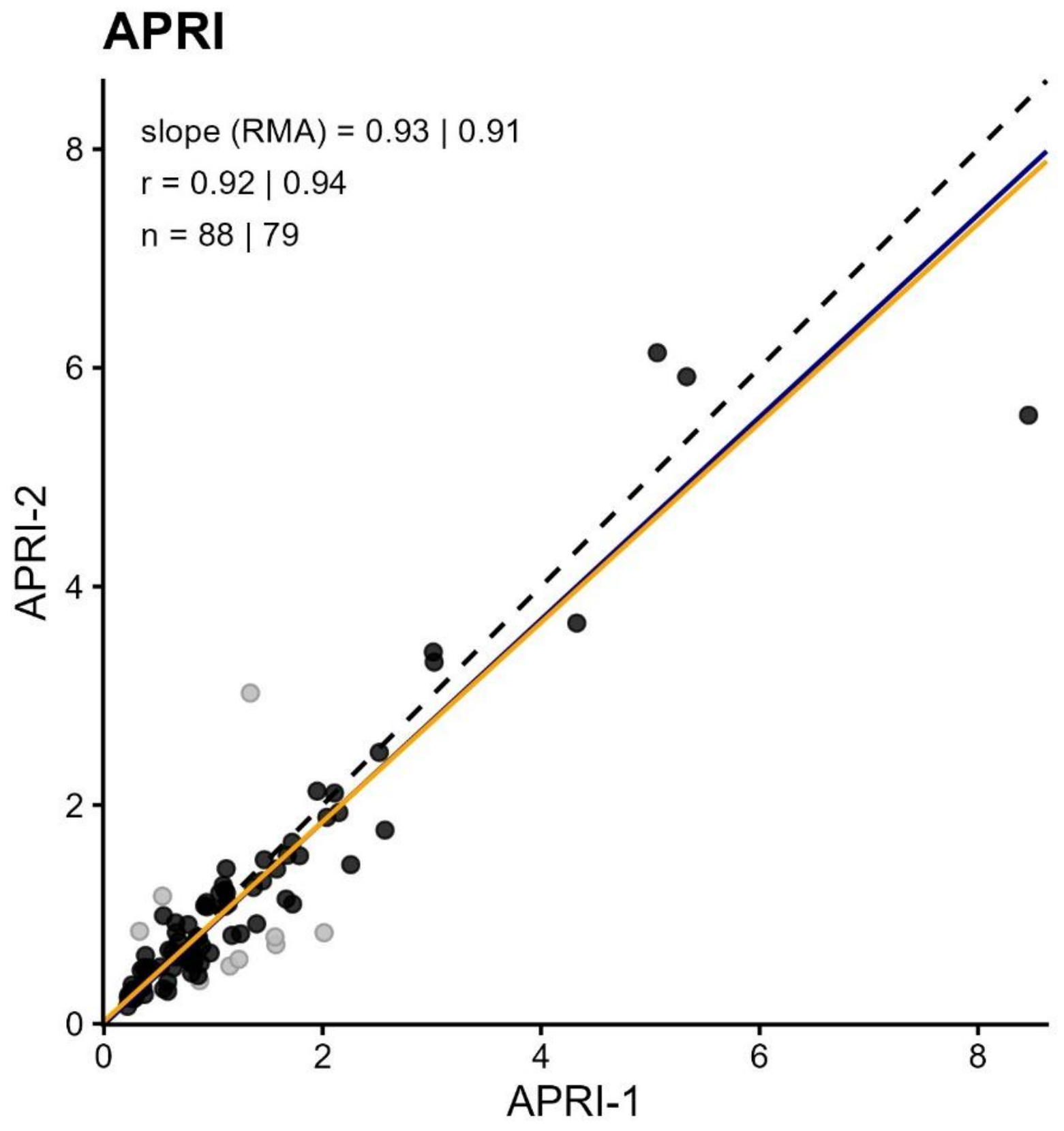

APRI
slope (RMA) = 0.93 | 0.91
r = 0.92 | 0.94
n = 88 | 79
APRI-2
APRI-1
0
2
4
6
8

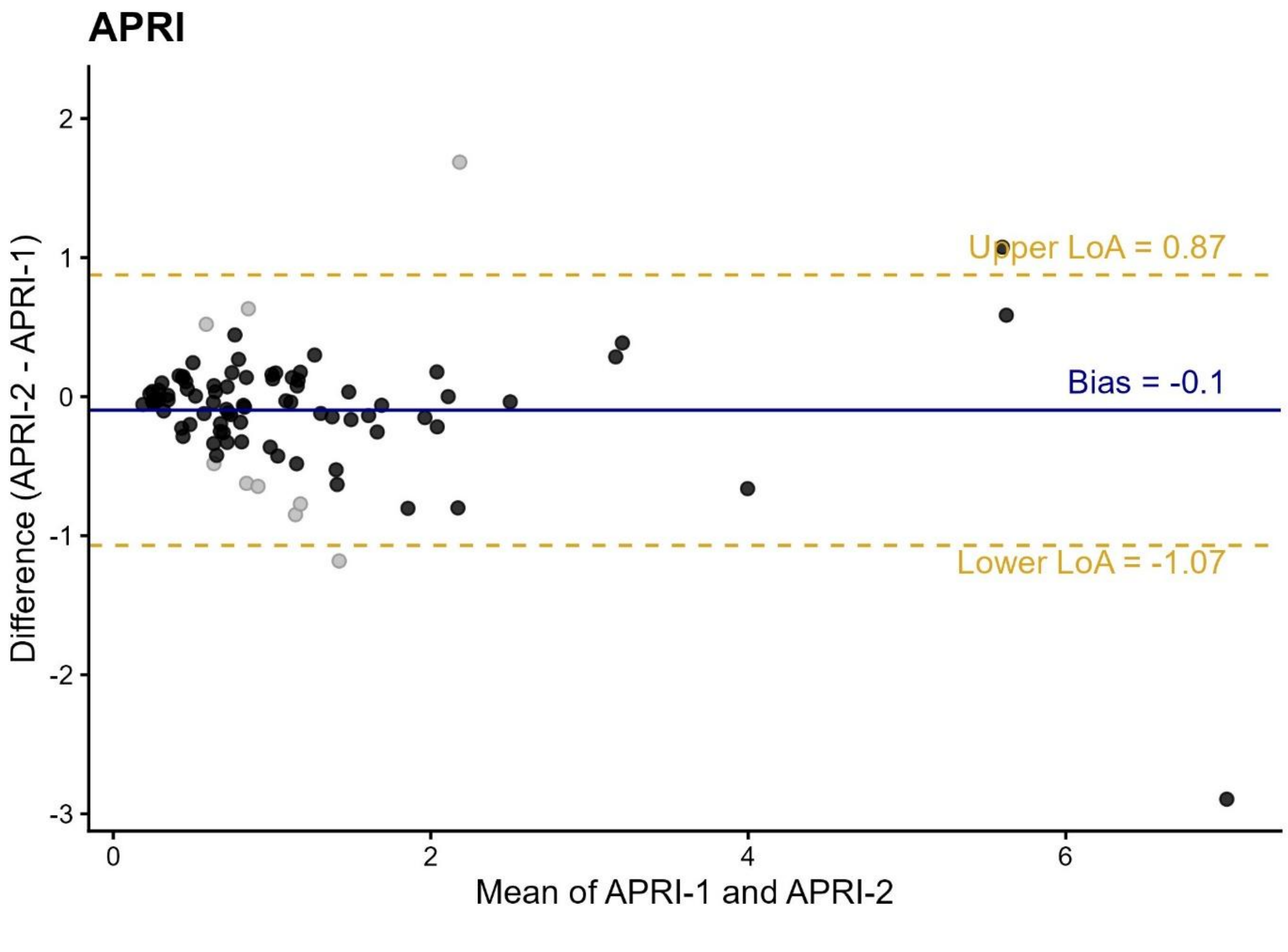

APRI
Upper LoA = 0.87
Bias = -0.1
Lower LoA = -1.07
Difference (APRI-2 - APRI-1)
Mean of APRI-1 and APRI-2
2
1
0
-1
-2
-3
0
2
4
6

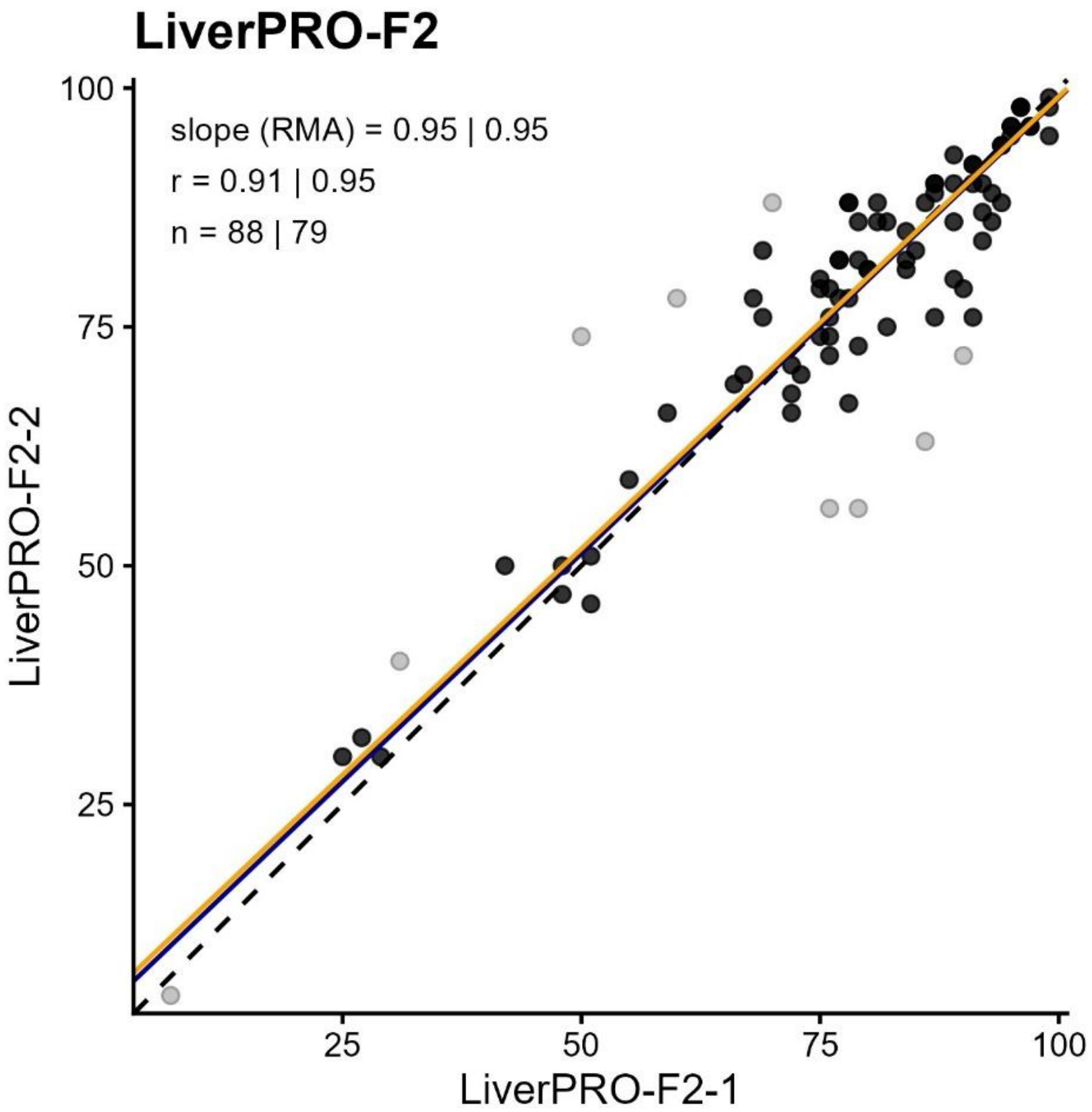
LiverPRO-F2
slope (RMA) = 0.95 | 0.95
r = 0.91 | 0.95
n = 88 | 79
100
75
50
25
LiverPRO-F2-2
25
50
75
100
LiverPRO-F2-1

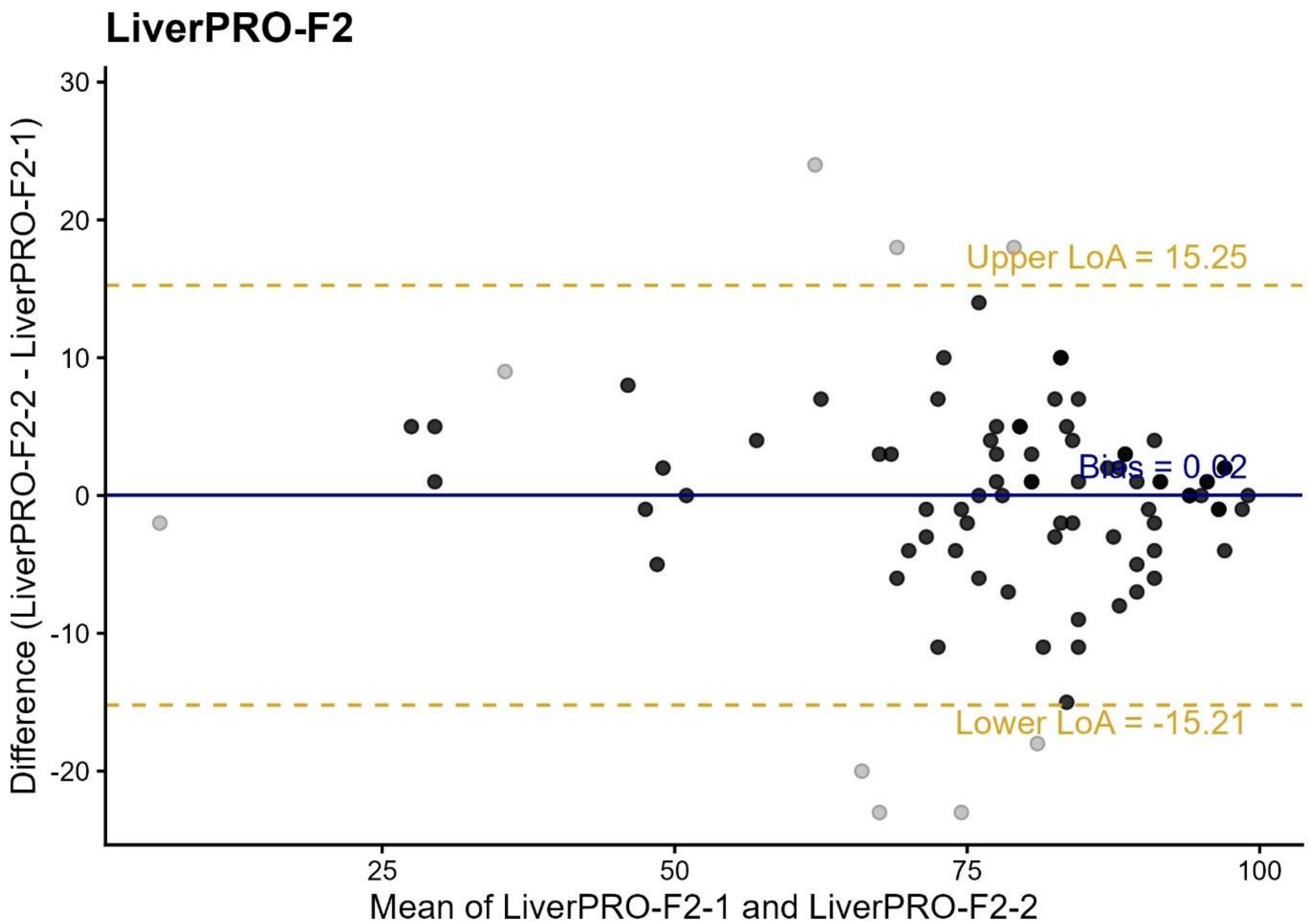
LiverPRO-F2
30
20
10
0
-10
-20
Difference (LiverPRO-F2-2 - LiverPRO-F2-1)
Upper LoA = 15.25
Bias = 0.02
Lower LoA = -15.21
25
50
75
100
Mean of LiverPRO-F2-1 and LiverPRO-F2-2

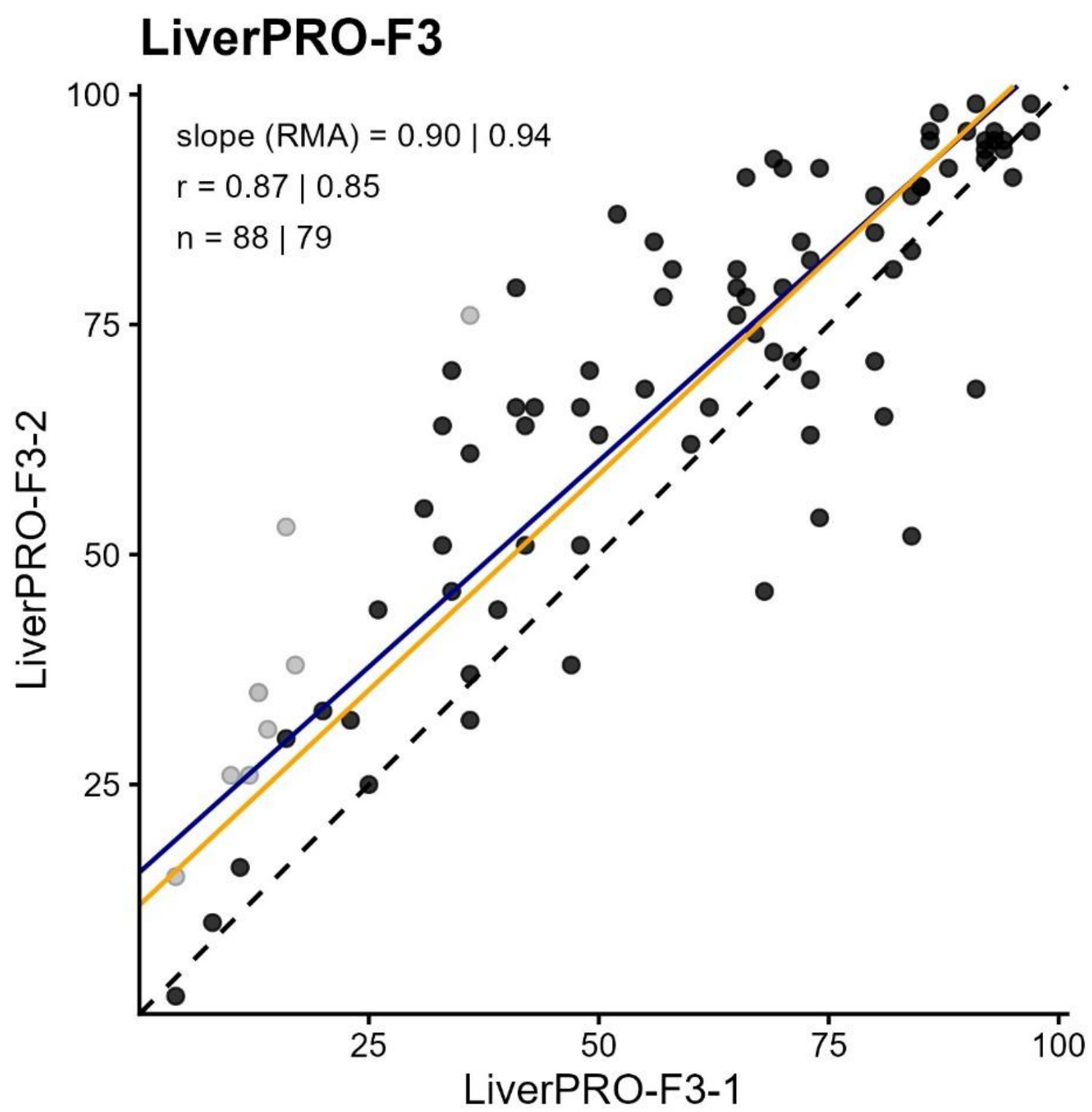
LiverPRO-F3
slope (RMA) = 0.90 | 0.94
r = 0.87 | 0.85
n = 88 | 79
LiverPRO-F3-2
LiverPRO-F3-1
100
75
50
25
25
50
75
100

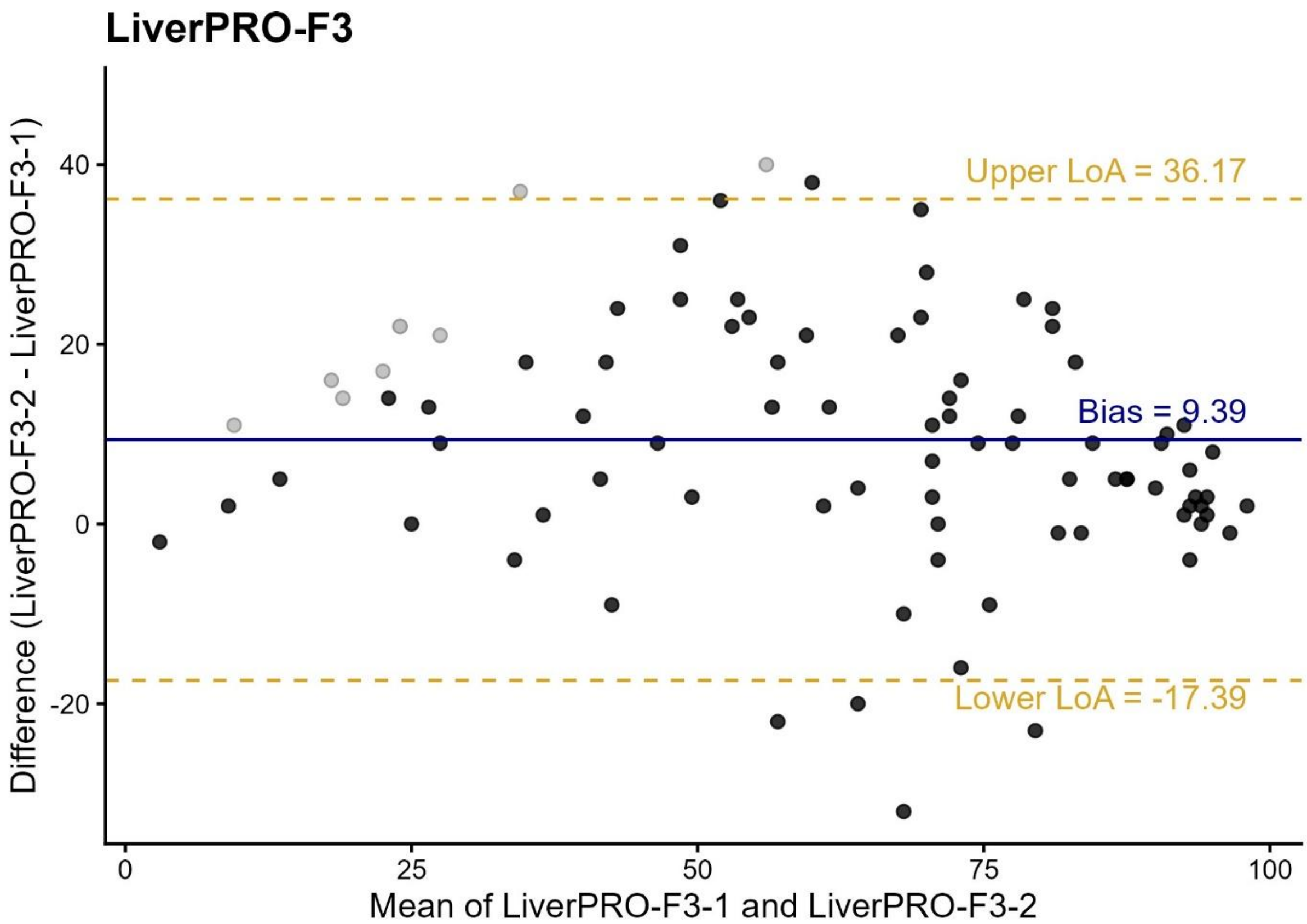
LiverPRO-F3
Upper LoA = 36.17
Bias = 9.39
Lower LoA = -17.39
Difference (LiverPRO-F3-2 - LiverPRO-F3-1)
Mean of LiverPRO-F3-1 and LiverPRO-F3-2
40
20
0
-20
0
25
50
75
100

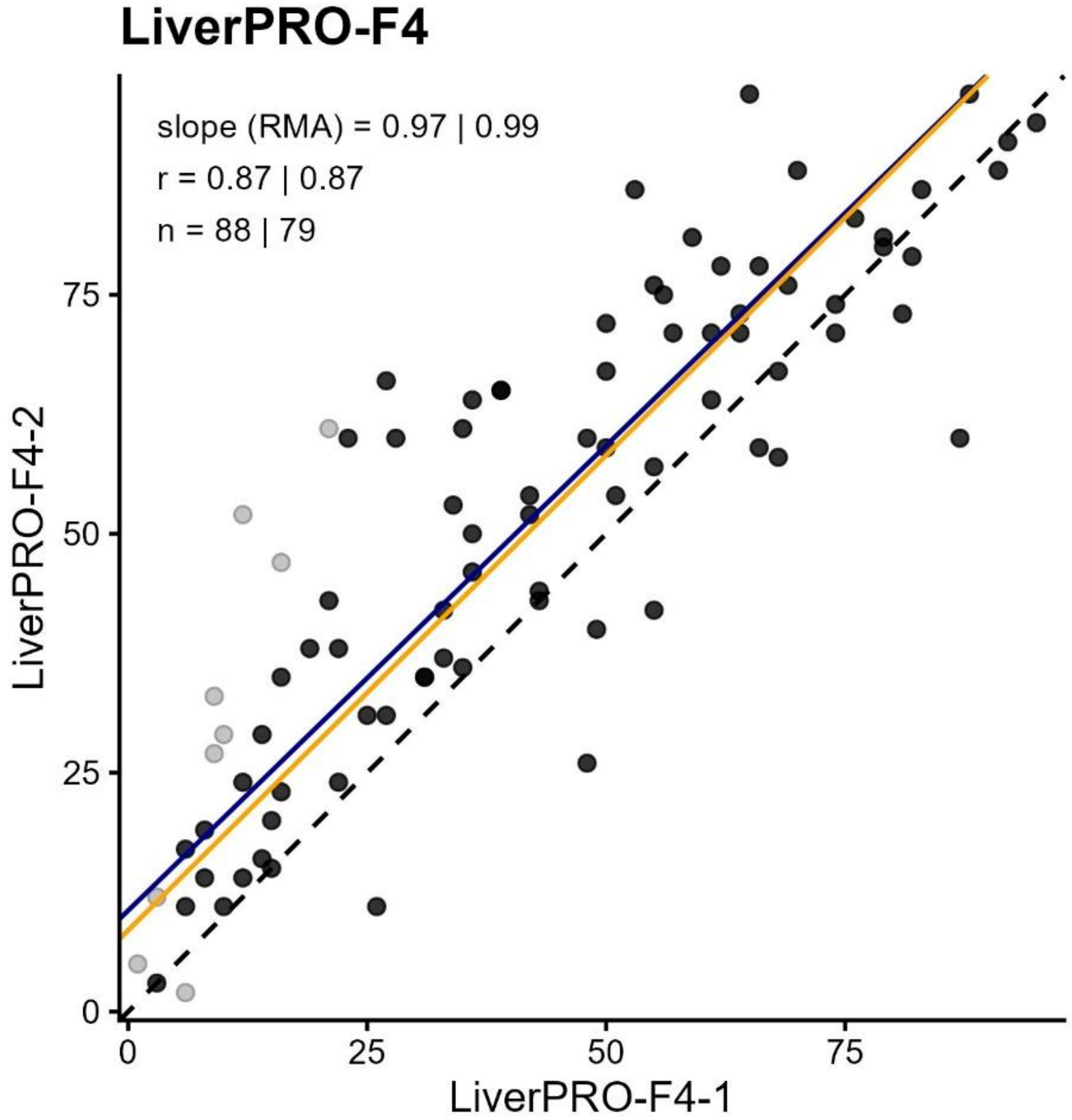
LiverPRO-F4
slope (RMA) = 0.97 | 0.99
r = 0.87 | 0.87
n = 88 | 79
75
50
25
0
LiverPRO-F4-2
0
25
50
75
LiverPRO-F4-1

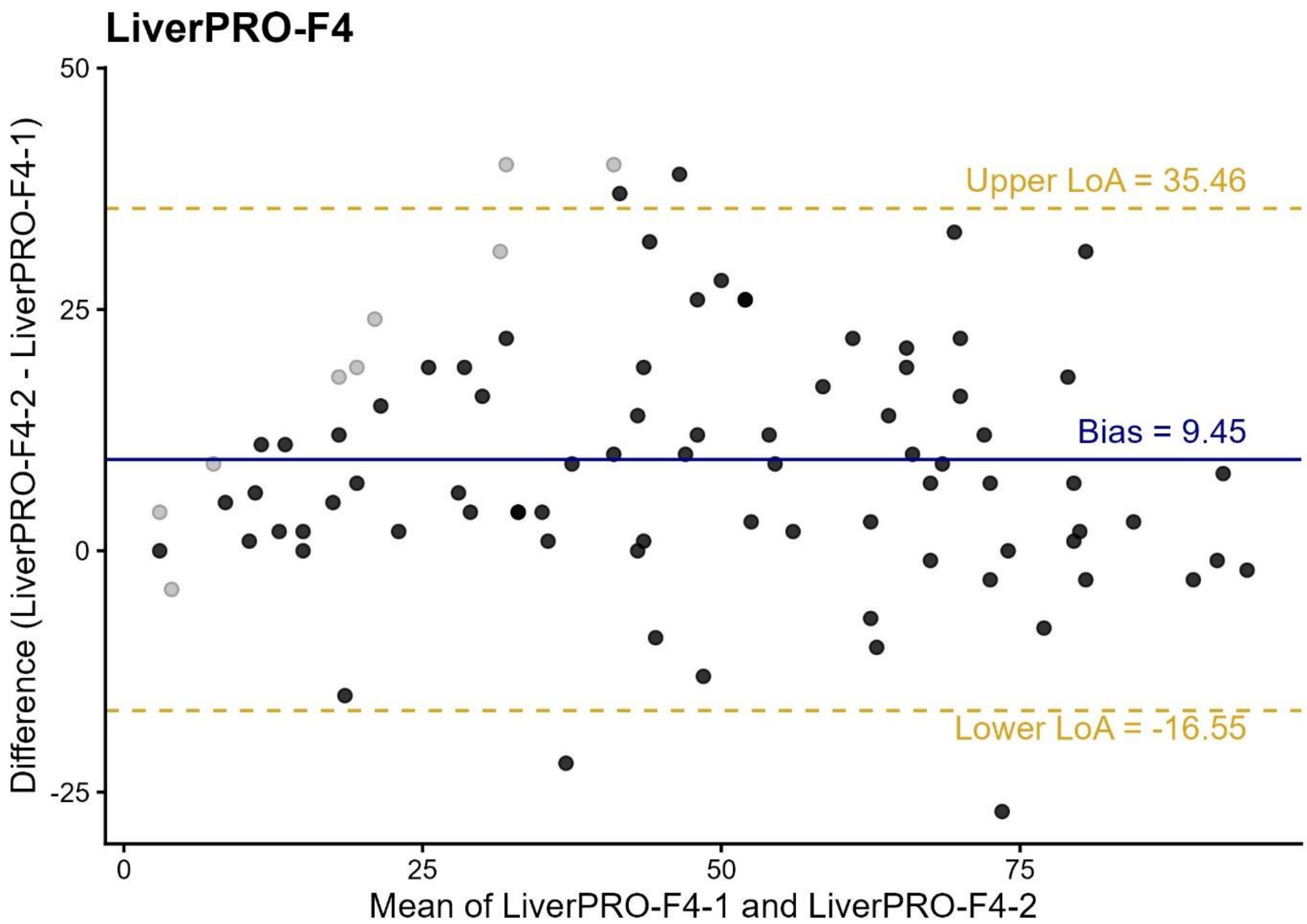
LiverPRO-F4
50
25
0
-25
Difference (LiverPRO-F4-2 - LiverPRO-F4-1)
Upper LoA = 35.46
Bias = 9.45
Lower LoA = -16.55
0
25
50
75
Mean of LiverPRO-F4-1 and LiverPRO-F4-2
